# Diastereoselective, Catalytic Access to Cross‐Aldol Products Directly from Esters and Lactones

**DOI:** 10.1002/anie.202209584

**Published:** 2022-08-19

**Authors:** Adrián Moreno González, Kieran Nicholson, Natalia Llopis, Gary S. Nichol, Thomas Langer, Alejandro Baeza, Stephen P. Thomas

**Affiliations:** ^1^ EaStCHEM School of Chemistry University of Edinburgh David Brewster Road Edinburgh EH9 3FJ UK; ^2^ AstraZeneca Pharmaceutical Technology & Development Chemical Development UK Silk Road Macclesfield SK10 2NA UK; ^3^ Instituto de Síntesis Orgánica and Dpto. de Química Orgánica Universidad de Alicante Apdo. 99 03080 Alicante Spain

**Keywords:** Aldol, Boron, C−C Bond, Main-Group Catalysis, Transborylation

## Abstract

High oxidation‐state carbonyl coupling partners including esters and lactones were reacted with enones to give aldol‐type products directly using two‐fold organoborane catalysis. This new retrosynthetic disconnection to aldol‐type products is compatible with enolisable coupling partners, without self‐condensation, and couples the high reactivity of secondary dialkylboranes with the stability of pinacolboronic esters. Excellent chemoselectivity, substrate scope (including those containing reducible functionalities and free alcohols) and diastereocontrol were achieved to access both the *syn*‐ and *anti*‐aldol‐type products. Mechanistic studies confirmed the two‐fold catalytic role of the single secondary borane catalyst for boron enolate formation and formation of an aldehyde surrogate from the ester or lactone coupling partner.

## Graphical abstract

A unique, catalytic method has been developed to directly access aldol‐type products from esters and lactones. Using borane catalysis and transborylation as the turnover mechanism, *syn*‐ and *anti*‐aldol‐type products were selectively obtained in excellent yield and stereoselectivity across a range of functionalised substrates, including bioactive natural and unnatural products.

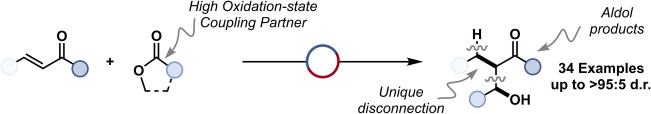

Synthetic chemistry has continually been inspired by and sought to mimic Nature.^[1]^ Cell membrane formation, which relies upon the synthesis of β‐hydroxy‐carbonyl compounds by type I and type II fatty acid synthases, is one of the most essential biological functions (Figure 1a).^[2]^ Biologically, this is a two‐step process: carbon‐carbon bond formation (coupling) with a high oxidation‐state carbonyl functionality to give a 1,3‐dicarbonyl and subsequent chemoselective reduction to a β‐hydroxy‐carbonyl. The synthetic equivalent requires a Claisen condensation^[3]^ to give the 1,3‐dicarbonyl followed by chemoselective reduction to a β‐hydroxy‐carbonyl. One‐step access to β‐hydroxy‐carbonyls is possible chemically but requires the use of, low oxidation‐state, aldehyde coupling partners (Figure 1b).^[4]^ Chemical aldol reactions have been elegantly used to recreate the reactivity of polyketide synthases (PKS I and II) and offer synthetic alternatives to biological reactivity.^[5]^ The high reactivity of aldehydes has necessitated the development and use of control strategies to direct cross‐aldol reactivity over self‐condensation, e.g. the use of silyl enol ethers.^[6]^ One‐step access to β‐hydroxy ketones using higher oxidation‐state coupling partners is unknown and such a coupling would provide a unique retrosynthetic disconnection without chemical or biological precedence.

**Figure 1:**
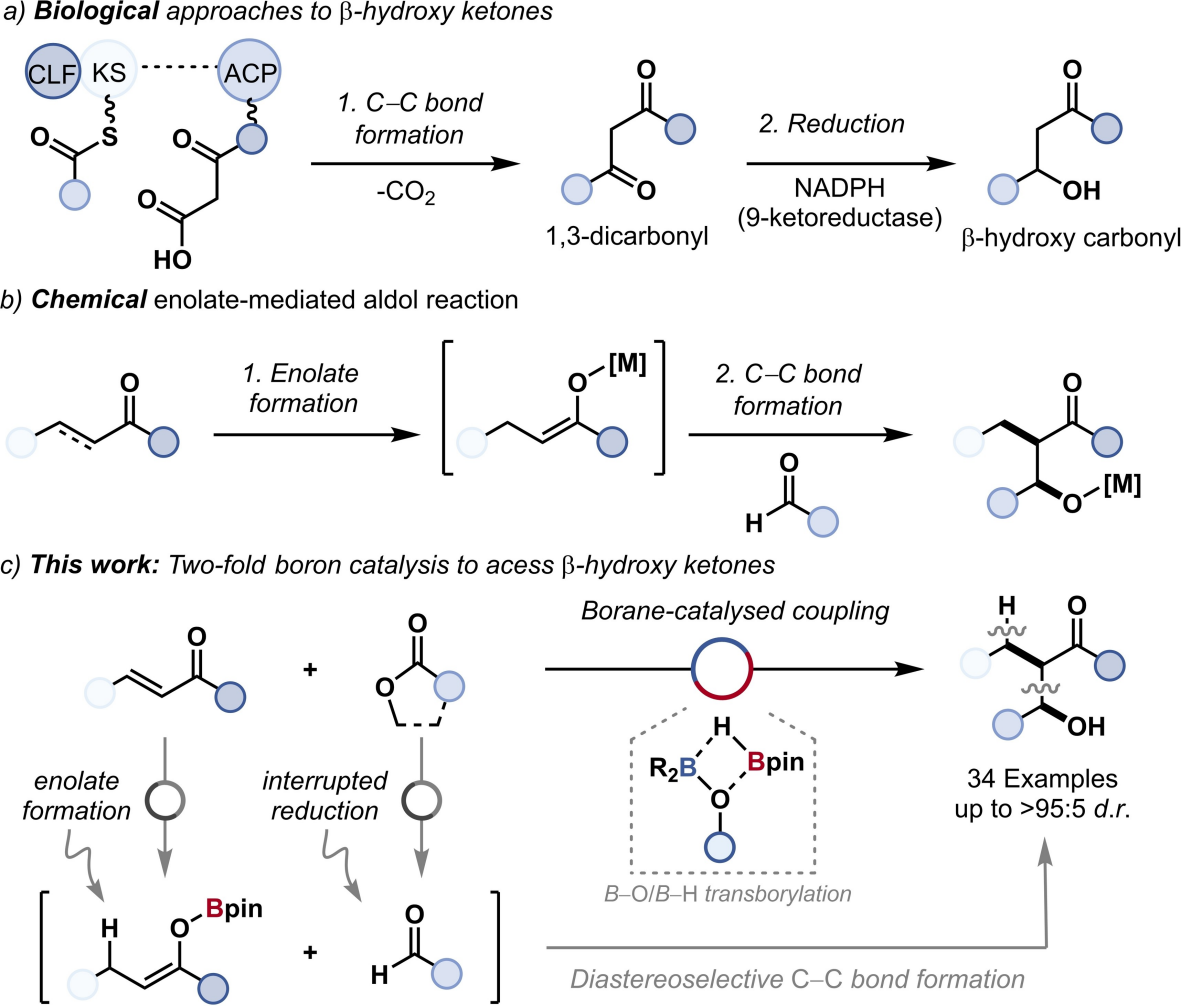
a) Biological approaches to β‐hydroxy carbonyls. b) Two‐step aldol reaction. c) Borane‐catalysed coupling of high‐oxidation‐state carbonyls.

The control of enolate chemistry is key to aldol methodologies. Enolates are conventionally generated by deprotonation of a carbonyl compound using base,^[7]^ however, rhodium‐,^[8]^ copper‐,^[9]^ and boron^[10]^ hydrides can react with α,β‐unsaturated carbonyls to give enolates (Figure 1b), including catalytic variants.[^8^, ^9^, ^11^] In all cases the formation of a β‐hydroxy carbonyl product requires an aldehyde or ketone coupling partner. Dialkylboranes (HBR_2_) have been used for the stoichiometric reduction of enones to boron enolates[^10a^, ^10b^, ^10c^, ^10d^] and the stoichiometric reduction of esters and lactones to alcohols.[^12^, ^13^] Although never isolated or intercepted at this stage, the reduction of an ester to an alcohol by a borane proceeds through an aldehyde intermediate. Thus, the potential for the catalytic generation of both the nucleophilic (boron enolate) and electrophilic (aldehyde) coupling partners of an aldol‐type reaction using a single borane catalyst was postulated, and for the first time the reaction of high oxidation‐state carbonyl reagents could be used to give aldol‐type products (Figure 1c). Crucially, aldehyde self‐condensation would be avoided by the lack of base‐mediated enolate formation, and the use of lactone coupling partners would give access to substrates unamenable to conventional aldol strategies without protecting groups.

Transborylation has enabled the stoichiometric reactions of common organoboranes to be rendered catalytic,^[14]^ however, this turnover strategy has not been applied to C−C bond formation or diastereoselective coupling reactions. It was hypothesised that the rates of both enone reduction to the boron enolate and ester reduction to the aldehyde surrogate could be controlled using transborylation as both require a borane catalyst and a dioxaborolane as the terminal reductant. Therefore, control of transborylation (boron‐boron exchange) would control the overall reaction kinetics and enable a chemically unknown, catalytic, aldol‐type reaction using high oxidation‐state carbonyl coupling partners using a single catalyst (Figure 1c). Significantly, this application of transborylation would be outwith established stoichiometric and catalytic coupling, including organoboron, chemistries.^[15]^

Using chalcone **1 a** and ethyl acetate **2 a**, the two‐fold catalytic coupling was optimised across secondary boranes (catalyst) and dioxaborolanes (terminal reductant) (See Supporting Information section 2 for details). 9‐Borabicyclo[3.3.1]nonane, [H‐*B*‐9‐BBN]_2_, (15 mol%) and pinacol borane (HBpin) (5 equiv) were found to be the optimal catalytic system giving the β‐hydroxy ketone **3 a** with high *syn*‐selectivity, presumably through generation and coupling of a (*Z*)‐boron enolate. No aldehyde self‐condensation product was observed. Other dialkylboranes including dicyclohexylborane and diisocamphenylborane were found to be unreactive for the ester reduction. Control reactions in the absence of the secondary borane (H‐*B*‐9‐BBN) catalyst showed no coupling reaction or reduction of the ketone or enone using HBpin alone. Ethyl esters gave the best yields and diastereoselectivity. The use of phenyl and *tert*‐butyl esters resulted in reduced yields and increased side reactions, including reduction of the enone to a saturated ketone. Replacing ethyl acetate with acetic anhydride or acetic acid also gave the β‐hydroxy ketone **3 a**, albeit in reduced yield (40 % and 12 %, respectively).

The scope and limitations of this enone‐ester coupling were then investigated (Table 1). Chalcone **1 a** and ethyl acetate **2 a** were reacted in 94 % isolated yield and 90 : 10 *d.r*. to give the corresponding *syn*‐β‐hydroxy ketone **3 a**. Several esters including ethyl propionate **3 b** (86 % yield, 90 : 10 *d.r*.), ethyl butanoate **3 c** (58 %, 91 : 9 *d.r*.), ethyl isovalerate **3 d** (82 %, 93 : 7 *d.r*.), ethyl 3,3‐dimethylbutanoate **3 e** (44 %, 86 : 14 *d.r*.) ethyl cyclobutylcarboxylate **3 f** (85 %, 82 : 18 *d.r*.), and ethyl cyclopropylcarboxylate **3 g** (50 %, 89 : 11 *d.r*.) were all successfully reductively‐coupled with chalcone **1 a** to give aldol‐type products, in good yields and diastereoselectivities. The *syn*‐selectivity of the coupling was confirmed by single crystal X‐ray analysis (**3 e**). The Lewis acidic catalyst was tolerant of substrates bearing Lewis basic and electron‐donating functionalities such as methoxy **3 h** (76 %, 93 : 7 *d.r*.), **3 i** (79 %, 90 : 10 *d.r*.), dimethoxy **3 j** (92 %, 87 : 13 *d.r*.), **3 k** (76 %, 85 : 15 *d.r*.), **3 l** (77 %, 92 : 8 *d.r*.), **3 m** (88 %, 87 : 13 *d.r*.), thioether **3 n** (74 %, 91 : 9 *d.r*.), **3 o** (78 %, 90 : 10 *d.r*.), and dimethylamino **3 p** (78 %, 89 : 11 *d.r*.) groups. Substrates bearing other electron‐donating, non‐coordinating functionalities such as a 4‐*tert*‐butyl substituent were reacted in moderate yield and excellent diastereoselectivity **3 q** (42 %, >95 : 5 *d.r*.).

**Table 1:**
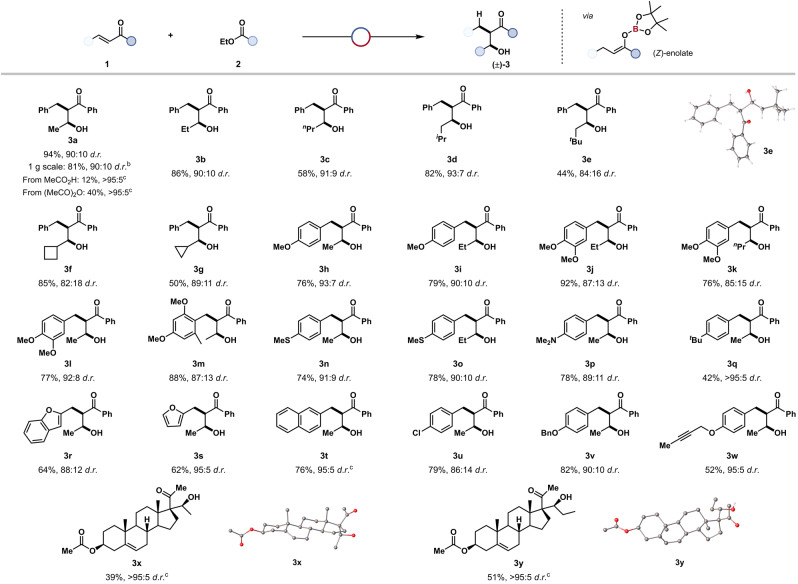
Substrate scope for borane‐catalysed enone‐ester coupling.

[a] Reaction conditions unless stated otherwise: [H‐*B*‐9‐BBN]_2_ (15 mol%), HBpin (5 equiv), substrate **3 a**–**y** (0.5 mmol, 1 equiv), ester (5 mL), 16 h, 40 °C; then 2‐aminoethanol. Diastereoselectivity determined by ^1^H NMR spectroscopy of the crude reaction mixture. [b] Reaction conditions: [H‐*B*‐9‐BBN]_2_ (15 mol%), HBpin (5 equiv), substrate **3 a** (5 mmol, 1 equiv), ester (50 mL), 16 h, 40 °C; then 2‐aminoethanol. Diastereoselectivity determined by ^1^H NMR spectroscopy of the crude reaction mixture. [c] Isolated as a single diastereomer. Thermal ellipsoids for crystal structures of **3 e**, **3 x** and **3 y** shown at 50 % probability level, red=oxygen, grey=carbon, white=hydrogen.

Substrates bearing alternative arene substituents including benzofuran **3 r** (64 %, 88 : 12 *d.r*.), furan **3 s** (62 %, 95 : 5 *d.r*.), and naphthalene **3 t** (76 %, isolated as single diastereomer) all underwent reductive coupling. 4‐Chlorochalcone **1 w** was coupled with ethyl acetate in good yield and diastereoselectivity **3 u** (79 %, 86 : 14 *d.r*.). The coupling was next applied to substrates bearing functionalities which has been observed to deleteriously react with metal hydrides,^[16]^ including benzyloxy **3 v** (82 %, 90 : 10 *d.r*.) and alkyne **3 w** (52 %, 95 : 5 *d.r*.) to give the substituted aldol‐type products in good yields and diastereoselectivity without any observed side‐reactions such as *O*‐deprotection and hydroboration of carbonyl or alkyne groups. Finally, 16‐dihydropregnenolone acetate, a precursor to four pharmaceuticals on the WHO List of Essential Medicines,^[17]^ was successfully coupled to give β‐hydroxy ketones **3 x** (39 %) and **3 y** (51 %) which were each isolated as a single diastereomer with stereochemical assignment confirmed by single crystal X‐ray analysis.

The use of lactones as coupling partners was next explored as this would highlight a significant advantage over traditional aldol strategies for the use of substrates bearing free alcohol groups (Scheme 1a). Generally, an aldol reaction with a hydroxyaldehyde as the coupling partner would require protection of the alcohol to prevent deactivation by the substrate oligimerisation.^[18]^ Lactones are widely commercially available, inexpensive, and offer greater stability than aldehydes. Due to the greater reactivity with respect to esters, only 5 equivalents of lactone were required. A dialkylborane catalyst was still required for the reduction of the lactone. Chalcone **1 a** was reacted with five‐membered lactone, γ‐butyrolactone, to give the aldol‐type product with a free pendant alcohol **4 a** in 77 % isolated yield and 90 : 10 *d.r*. The seven‐member lactone, ϵ‐caprolactone, was also reacted to give dihydroxy ketone **4 b** in 70 % yield and 86 : 14 *d.r*. Scale‐up of this reaction to 1 gram scale gave the product **4 b** in 66 % yield which recrystallised on standing to give the *syn*‐diastereoisomer only (23 %). The presence of substituents about the lactone did not hinder reactivity with (±)‐γ‐nonanoic lactone and it was successfully coupled to the *syn*‐aldol‐type product **4 c** (69 %, 90 : 10 *syn*:*anti*), as a mixture of alcohol epimers reflective of the use of racemic lactone. Lactone coupling could be further applied to alkyl‐enones, 4‐hexen‐2‐one **4 d** (52 %, isolated as single diastereomer) and 4‐octen‐2‐one **4 e** (56 %, isolated as single diastereomer).

**Scheme 1:**
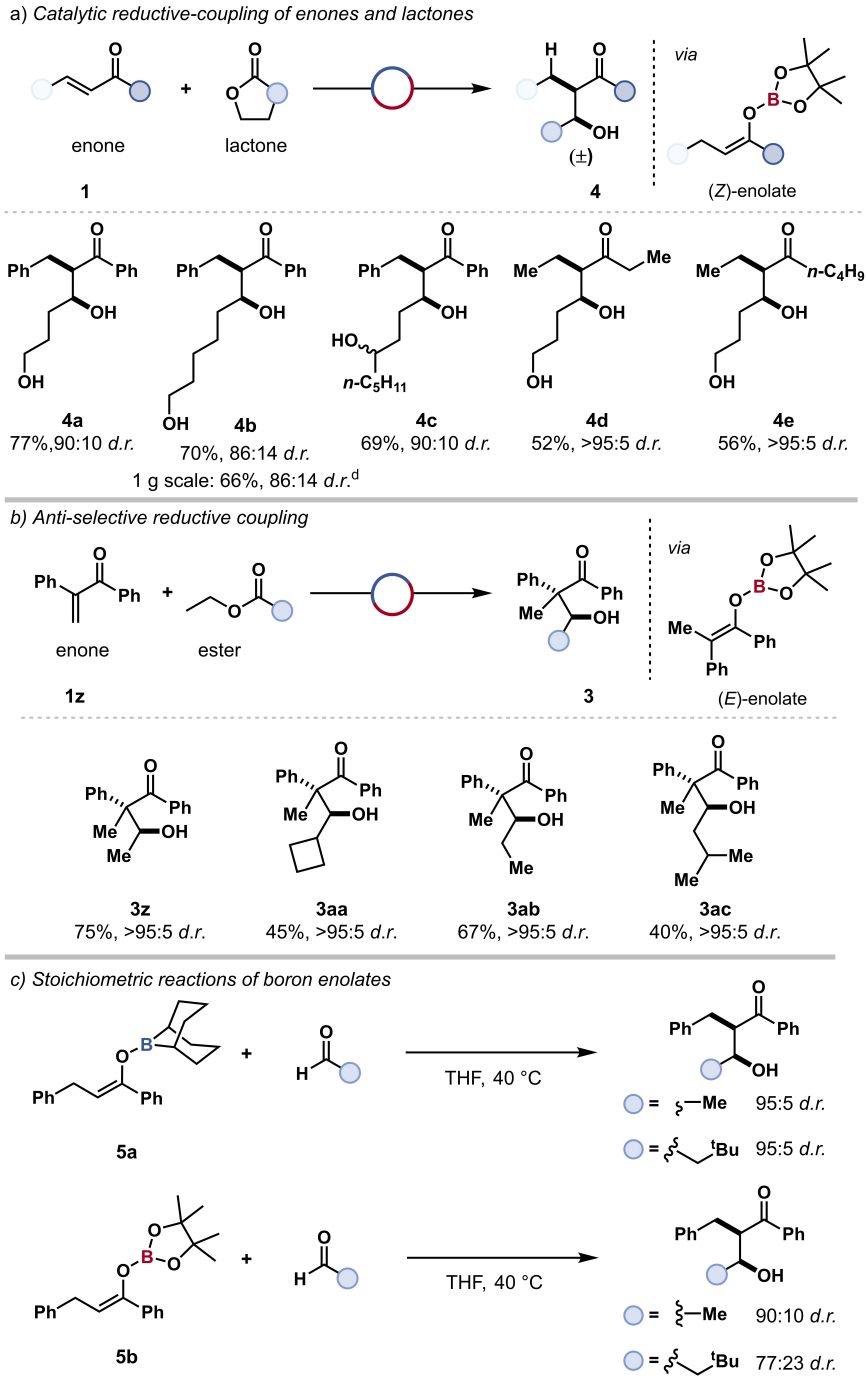
a) Borane‐catalysed enone‐lactone coupling. Reaction conditions unless stated otherwise: [H‐*B*‐9‐BBN]_2_ (15 mol%), HBpin (5 equiv), substrate **4 a**–**e** (0.5 mmol, 1 equiv), lactone (5 equiv), 16 h, 40 °C; then 2‐aminoethanol. Diastereoselectivity determined by ^1^H NMR spectroscopy of the crude reaction mixture. b) *Anti*‐selective borane‐catalysed enone‐ester coupling. Reaction conditions unless stated otherwise: [H‐*B*‐9‐BBN]_2_ (15 mol%), HBpin (5 equiv), substrate **3 z**–**ac** (0.5 mmol, 1 equiv), ester (5 mL), 16 h, 40 °C. c) Stoichiometric aldol reaction. Reaction conditions unless stated otherwise: boron enolate **5 a**–**b** (0.5 mmol, 1 equiv), aldehyde (0.5 mmol, 1 equiv), 0.1 M THF, 16 h, 40 °C; then 2‐aminoethanol. Diastereoselectivity determined by ^1^H NMR spectroscopy of the crude reaction mixture. d) Reaction conditions: [H‐*B*‐9‐BBN]_2_ (25 mol%), HBpin (5 equiv), substrate **3 a** (5 mmol, 1 equiv), ϵ‐caprolactone (5 equiv), 16 h, 40 °C; then 2‐aminoethanol. Yield and diastereoselectivity determined by ^1^H NMR spectroscopy of the crude reaction mixture, isolated as a single diastereomer following recrystallisation.

The enone‐ester coupling was found to be *syn*‐selective, presumably due to the generation of the (*Z*)‐enolate by 1,4‐hydroboration of the enone^[19]^ and coupling proceeding through a Zimmerman−Traxler‐type transition‐state structure.^[5]^ Appropriate choice of enone could therefore be used to access the (*E*)‐enolate and thus *anti*‐β‐hydroxy ketone. Application of the coupling protocol to 1,2‐diphenylprop‐2‐en‐1‐one **1 z** with different esters gave several *anti*‐β‐hydroxy ketones with moderate yields and excellent diastereoselectivity. Enone **1 z** was successfully reacted with ethyl acetate **3 z** (75 %), ethyl cyclobutylcarboxylate **3 aa** (75 %), ethyl propionate **3 ab** (67 %) and ethyl isovalerate **3 ac** (40 %), each was isolated as a single diastereomer (Scheme 1b).

The diastereoselectivity of the aldol reaction has been widely studied with the nature of the enolate having a direct influence on the diastereoselectivity of the β‐hydroxy carbonyl product.^[5]^ Two organoboron species are present in the reaction, H‐*B*‐9‐BBN and HBpin, and both could form the reacting enolate, O‐*B*‐9‐BBN enolate **5 a** and *O*‐Bpin enolate **5 b**, respectively (Scheme 1c). To investigate which enolate was active, and the inherent stereoselectivity of this enolate, both enolates were independently prepared and reacted with an aldehyde under reaction conditions. In the reaction with acetaldehyde, the O‐*B*‐9‐BBN enolate gave the β‐hydroxy ketone in higher diastereoselectivity (>95 : 5 *d.r*.) than the *O*‐Bpin enolate (90 : 10 *d.r*.). The latter diastereoselectivity matched that of the catalytic reaction, thus it was proposed that the enone‐ester coupling proceeds through an *O*‐Bpin enolate **5 b**. Using 3,3‐dimethylbutyraldehyde, the O‐*B*‐9‐BBN enolate, **5 a**, gave excellent diastereoselectivity (>95 : 5 *d.r*.), however, the *O*‐Bpin enolate, **5 b** gave a lower diastereoselectivity (77 : 23 *d.r*.) than that of the catalytic reaction (84 : 16 *d.r*.), indicating a role for both boron enolates in some cases.

To investigate whether both enolates were contributing to the catalytic reaction, the reaction of chalcone **1 a** with ethyl acetate **2 a** was tracked over time (see Supporting Information Figure S11). The ^1^H and ^11^B NMR spectra studies revealed that the enone was fully converted to the corresponding Bpin enolate **5 b** before any significant amount of aldol‐type product **3** could be observed to form, and that the rate of enone reduction greatly exceeded that of ester reduction (See Supporting Information section 10). Moreover, at each time point the *d.r*. matched that of the stoichiometric reaction of the *O*‐Bpin enolate (see Supporting Information Figure S4).

The stoichiometric reduction of esters and lactones to alcohols is well established^13^ and using disiamylborane Brown showed the reduction of γ‐butyrolactone to 4‐hydroxybutyraldehyde.^[13b]^ However, a catalytic variant is unknown and the semi‐reduction of esters or lactones has not been harnessed to enable aldehyde‐type reactivity. The mechanism of this catalytic, semi‐reduction was therefore investigated. Stoichiometric reaction of γ‐butyrolactone with HBpin (2 equiv) gave no reduction to the lactol, 2‐hydroxytetrahydrofuran, or alcohol, 1,4‐butanediol, as observed by ^1^H and ^11^B NMR spectroscopy (See Supporting Information section 8). Upon addition of [H‐*B*‐9‐BBN]_2_ (15 mol%) complete reduction to the alcohol was observed (Scheme 2a). The hemiacetal derived from γ‐butyrolactone, 2‐hydroxytetrahydrofuran, was independently reacted with stoichiometric H‐*B*‐9‐BBN (2 equiv) and HBpin (2 equiv). In both cases the formation of the corresponding aldehyde, 4‐hydroxybutyraldehyde, was observed. Control experiments in the absence of the boron species showed no observed ring‐opening (Scheme 2b).

**Scheme 2:**
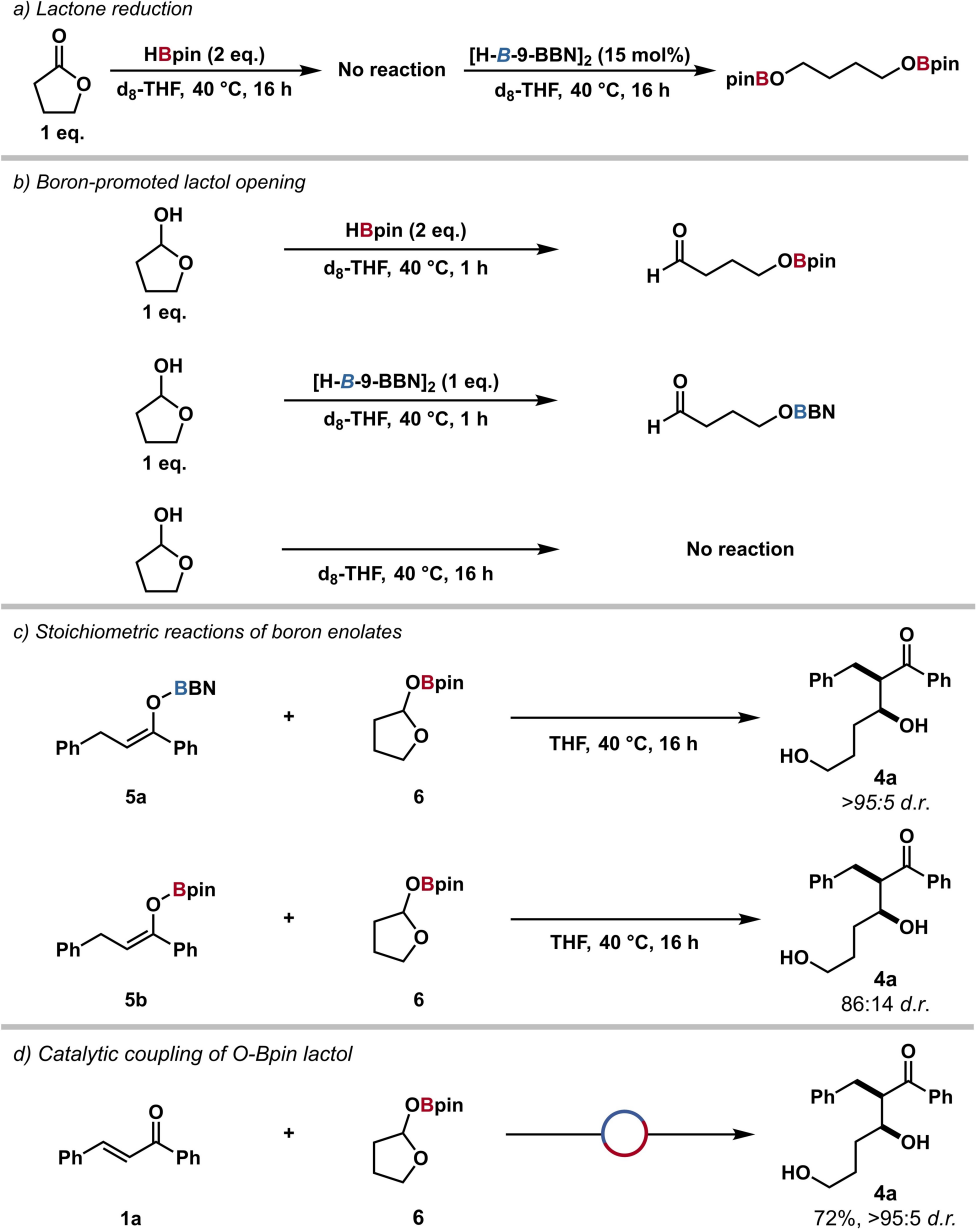
a) Borane‐catalysed reduction of γ‐butyrolactone. Reaction conditions: γ‐butyrolactone (1 equiv), HBpin (2 equiv), 16 h, 40 °C; then [H‐*B*‐9‐BBN]_2_ (15 mol%), 16 h, 40 °C. b) Boron‐promoted lactone opening. c) Stoichiometric reactions of boron‐enolates with *O*‐Bpin lactol. Reaction conditions: boron enolate **5 a**–**b** (0.5 mmol, 1 equiv), *O*‐Bpin lactol **6** (1.2 equiv), 16 h, 40 °C; then 2‐aminoethanol. d) Borane‐catalysed enone‐hemiacetal coupling. Reaction conditions: substrate **1 a** (0.5 mmol, 1 equiv), [H‐*B*‐9‐BBN]_2_ (15 mol%), HBpin (5 equiv), lactol‐Bpin **6** (5 equiv), 16 h, 40 °C; then 2‐aminoethanol. Diastereoselectivity determined by ^1^H NMR spectroscopy of the crude reaction mixture.

Stoichiometric reactions of O‐*B*‐9‐BBN and *O*‐Bpin enolates with lactol‐Bpin **6** were next investigated. The O‐*B*‐9‐BBN enolate gave the aldol‐type product with excellent diastereoselectivity (>95 : 5 *d.r*.) whereas the *O*‐Bpin enolate gave modest diastereoselectivity (86 : 14 *d.r*.), the latter matching the diastereoselectivity observed during catalysis (Scheme 2c). Finally, a catalytic reaction using *O*‐Bpin lactol **6** as the coupling partner, gave the β‐hydroxy ketone **4 a** in good yield and excellent diastereoselectivity (72 %, >95 : 5 *d.r*.) (Scheme 2d). Indicating that under catalytic conditions the O‐*B*‐9‐BBN enolate is reactive when lactols were used as coupling partners. The lower diastereoselectivity observed when lactones were used presumably arose due to higher concentrations of the *O*‐Bpin enolate in solution due to the relatively low rate of lactone to lactol reduction compared to transborylation of the enolate.

A mechanism for the boron‐catalysed reductive enone‐ester coupling was proposed where the catalyst, (H‐*B*‐9‐BBN)_,_ reacts with the enone to give an O‐*B*‐9‐BBN enolate, **I**. This enolate react with HBpin to give an *O*‐Bpin enolate, **II**, and regenerate the catalyst, H‐*B*‐9‐BBN. Concurrently the catalytic system acts to reduce the ester to a hemiacetal intermediate, **III**, which can be converted into the aldehyde and reacts with the, previously generated, *O*‐Bpin enolate to give the product β‐hydroxy ketone **3**, with the *O*‐Bpin bond hydrolysed on work‐up (Figure 2). In cases where highly reactive aldehyde or lactol substrates were used, aldol‐type reaction proceeded through the O‐*B*‐9‐BBN enolate **I**, i.e. the rate of C−C bond formation was equal or faster than transborylation of the enolate.

**Figure 2:**
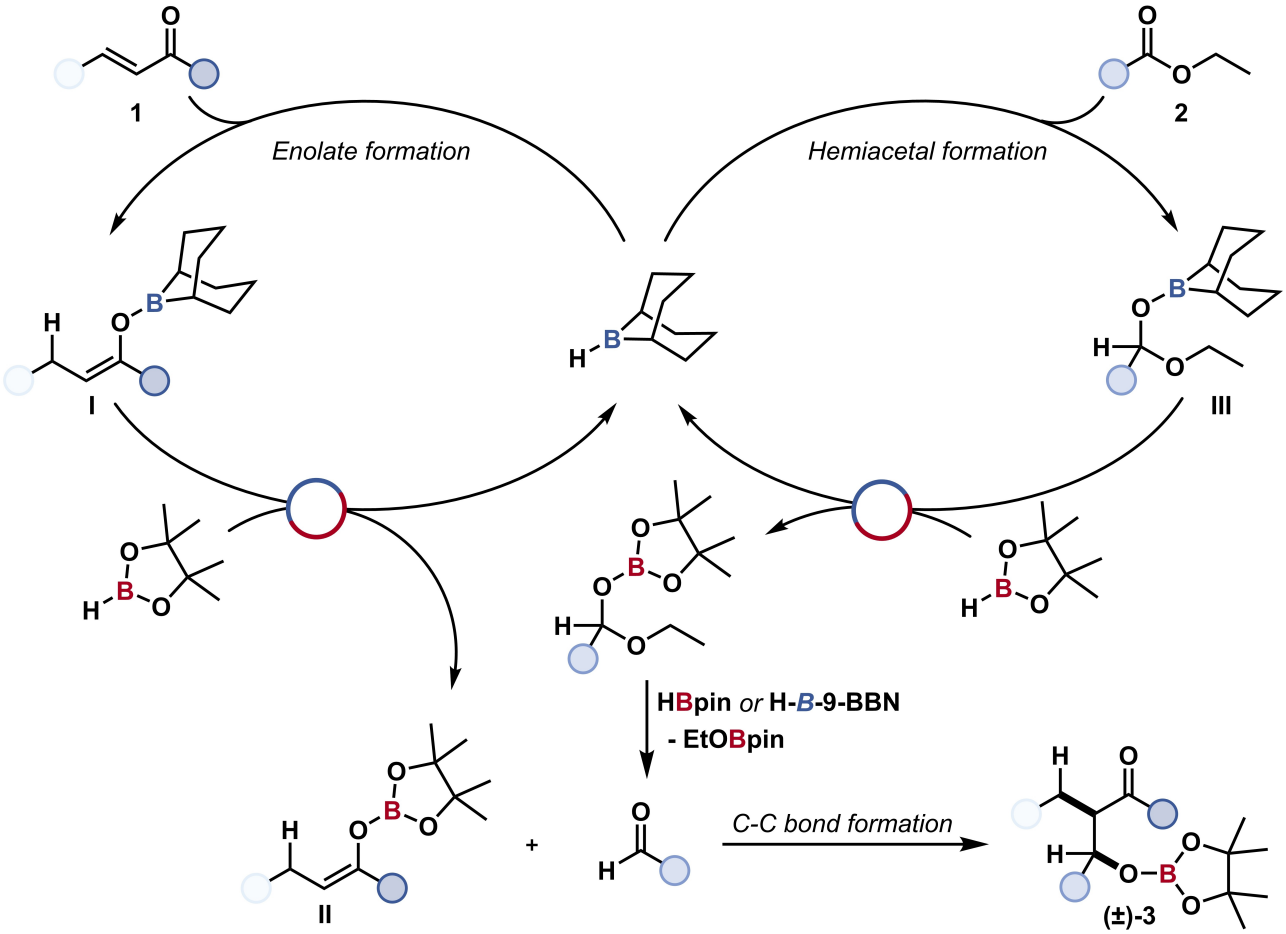
Proposed mechanism for the boron‐catalysed reductive enone‐ester coupling.

In summary, two‐fold boron catalysis has been developed to enable the preparation of aldol‐type products using a novel retrosynthetic disconnection. β‐Hydroxy ketones were prepared directly from high oxidation‐state carbonyl reagents with both *syn*‐ and *anti*‐selectivity and proceeded without the observation of any self‐condensation products. This application of organoborane catalysis to enone‐ester and enone‐lactone coupling reactions provides the first example of *B−O* transborylation in two‐fold catalysis and for diastereoselective C−C bond formation. H‐*B*‐9‐BBN and HBpin were used as the catalyst and turnover reagent for the reaction which was applied to a broad substrate scope showing tolerance to reducible functionalities and biological derivatives. Mechanistic studies identified a boron enolate and aldehyde surrogate as potential on cycle species, both of which were generated by boron catalysis enabled by *B−O* transborylation.

## Conflict of interest

The authors declare no conflict of interest.

## Supporting information

As a service to our authors and readers, this journal provides supporting information supplied by the authors. Such materials are peer reviewed and may be re‐organized for online delivery, but are not copy‐edited or typeset. Technical support issues arising from supporting information (other than missing files) should be addressed to the authors.

Supporting Information

Supporting Information

Supporting Information

Supporting Information

## Data Availability

The data that support the findings of this study are openly available in Cambridge Crystallographic Data Centre and Fachinformationszentrum Karlsruhe Access Structures at http://www.ccdc.cam.ac.uk/structures reference number 2143222.
