# Supplementary material for: Diastereoselective, Catalytic Access to Cross‐Aldol Products Directly from Esters and Lactones
Source: Angew Chem Int Ed Engl. 2022 Aug 19;61(39):e202209584. doi: 10.1002/anie.202209584 (PMC9804986; doi:10.1002/anie.202209584)
Supplement: Supplementary file 1 — Supporting Information [file ANIE-61-0-s003.pdf]

Angewandte Chemie
Eine Zeitschrift der Gesellschaft Deutscher Chemiker

Supporting Information

Diastereoselective, Catalytic Access to Cross-Aldol Products Directly from Esters and Lactones

A. Moreno González, K. Nicholson, N. Llopis, G. S. Nichol, T. Langer, A. Baeza, S. P. Thomas**

Supporting Information

Diastereoselective, Catalytic Access to Cross-Aldol Products Directly from Esters and Lactones

Adrián Moreno González[a], Kieran Nicholson[a]*, Natalia Llopis[a], Thomas Langer[b], Alejandro Baeza[c], Stephen P. Thomas[a]*

[a]EaStCHEM School of Chemistry, University of Edinburgh, David Brewster Road, Edinburgh, EH9 3FJ.

[b]Dr Thomas Langer, Chemical Development, Pharmaceutical Development &Technology, Operations, AstraZeneca, Macclesfield, UK

[c]Prof. Alejandro Baeza, Instituto de Síntesis Orgánica and Dpto. de Química Orgánica, Universidad de Alicante, Apdo. 99, 03080 Alicante, Spain.

k.nicholson-3@sms.ed.ac.uk and stephen.thomas@ed.ac.uk

Contents

S1 General Experimental Information 3

S2 Reaction Optimisation 5

S3 Ester reduction catalysed by dialkylboranes 6

S4 Substrate synthesis 7

S5 General Procedures 8

S6 Substrate Scope 9

S7 Gram-scale Aldol-type Reactions 45

S8 Mechanistic studies 47

S9 Incompatible substrates 58

S10 Study of diastereoselectivity over time 59

S11 Spectroscopic data 62

S12 Crystal Data and Experimental 156

S12 References 180

S1 General Experimental Information

Reaction Setup: All reactions were carried out in oven (185 °C) dried glassware, which had been cleaned using base (KOH in propan-2-ol) and acid (HCl in H_2O) baths. All air- and moisture sensitive reactions were carried out using an argon atmosphere glovebox or a Schleck line (nitrogen). All reported reaction temperatures corresponded to external bath temperatures. Room temperature was approximately 20 °C. "Brine" refers to a saturated solution of sodium chloride in H_2O. All reductive-coupling reactions were carried out in round bottom screw thread vial (13 x 100 mm) sealed with a 13 mm vial screw thread cap.

NMR Spectroscopy: ^{1}H, ^{13}C, and ^{11}B NMR Spectrum were recorded on Bruker Avance III 400, 500 MHz, and 600 MHz; Bruker Avance III-HD 500 MHz spectrometers. Chemical shifts are reported in parts per million (ppm). ^{1}H NMR Spectrum were referenced to the residual proteosolvent peak ($CHCl_3$: 7.26 ppm, CH_2Cl_2: 5.32 ppm, THF: 1.73 ppm). ^{13}C NMR Spectrum were referenced to the solvent peak ($CDCl_3$: 77.00 ppm). Multiplicities are shown as s (singlet), d (doublet), t (triplet), q (quartet), quin. (quintet), sext. (sextet), sept. (septet), non. (nonet), app. (apparent), br. (broad), obsc (obscure). Coupling constants, *J*, are reported in Hertz, rounded to the nearest 0.1 Hz and as observed. Integration of peaks is provided with the assignments indicated where appropriate.

Infrared Spectroscopy: Infra-red (IR) Spectrum were recorded on a Perkin-Elmer Spectrum One FT-IR, or Shimadzu IRAffinity-1 spectrometer (serial no. A213749) spectrometer. Peaks are reported in cm^{-1} with indicated relative intensities: s (strong, 0-33% T), m (medium, 34-66% T), w (weak, 67-100% T), and br (broad).

Chromatography: Column chromatography was carried out on a Teledyne ISCO CombiFlash NextGen 300+ using RediSep R_f normal phase silica flash columns (12, 25, 40, or 80 g; 20-40 microns). Substrates were purified using 40/60 petroleum ether and EtOAc on a gradient of 100:0 to 0:100 with flow rates of 10-110 mL min^{-1} depending on the size of column and ΔR_f.

***Mass Spectrometry*:** Mass spectrometry (MS) was performed by the University of Edinburgh, School of Chemistry, Mass Spectrometry Laboratory. High resolution mass Spectrum were recorded on a VG autospec, or Thermo/Finnigan MAT 900, mass spectrometer. Electron Impact (EI^+) Spectrum were performed at 70 eV using methane as the carrier gas, with either a double focusing sector field (DFSF) or time-of-flight (TOF) mass analyzer. Chemical Ionization (CI^+) Spectrum were performed with methane reagent gas, with either a double focusing sector field (DFSF) or time-of-flight (TOF) mass analyzer. Electrospray Ionization (ESI^+) Spectrum were performed using a time-of-flight (TOF) mass analyzer. Data are reported in the form of m/z (intensity relative to the base peak = 100).

Melting Points: Melting points were determined using a Stuart Scientific SMP10, or Griffin Gallankamp and are uncorrected.

***Chemicals**:* All reagents were purchased from Sigma Aldrich, Acros Organics or Alfa Aesar, or were synthesised in the laboratory.

Solvents: All solvents for air- and moisture sensitive techniques were obtained from an anhydrous solvent system (Innovative Technology). Reaction solvents tetrahydrofuran (THF) (Fisher, HPLC grade), ether (Et_2O) (Fisher, BHT stabilized ACS grade), and dichloromethane (CH_2Cl_2) (Fisher, unstabilised HPLC grade) were dried by percolation through two columns packed with neutral alumina under a positive pressure of argon. Toluene (ACS grade) was dried by percolation through a column packed with neutral alumina and a column packed with Q5 reactant (supported copper catalyst for scavenging oxygen) under a positive pressure of argon. Solvents for filtration, transfers, chromatography, and recrystallization were dichloromethane (CH_2Cl_2) (ACS grade), ether (Et_2O) (Fisher, BHT stabilised ACS grade), ethyl acetate (EtOAc) (Fisher, ACS grade), *n*-hexane (Optima), methanol (MeOH) (ACS grade), pentane (ACS grade), and petroleum ether (40–60°C, ACS grade).

Diastereoselectivity: Diastereoselectivity was determined from ^{1}H NMR of the crude reaction mixture. In each case an expansion of the ^{1}H NMR spectrum of the crude reaction mixture is shown with the integration of the *syn* and *anti* products to confirm the assigned diastereoselectivity.

S2 Reaction Optimisation

Table S1: Optimisation of reaction conditions

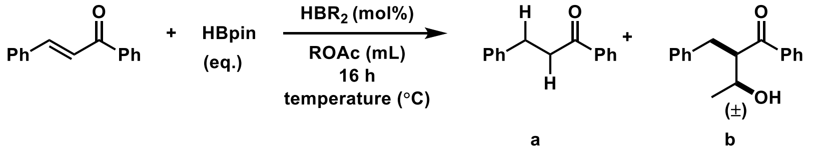

Entry	HBpin (eq.)	HBR_2	HBR_2 (mol%)	ROAc (ml)	R	Temp. (°C)	Conversion (%)	a:b	*d.r.* (*syn*:*anti*)
1	1.2	[H-*B*-9-BBN]$_2$	4	0.25	Et	40	>95	88:12	-
2	1.2	[H-*B*-9-BBN]$_2$	4	0.50	Et	40	>95	100:0	-
3	1.2	[H-*B*-9-BBN]$_2$	4	0.75	Et	40	>95	100:0	-
4	1.2	[H-*B*-9-BBN]$_2$	4	1	Et	40	>95	80:20	-
5	1.5	[H-*B*-9-BBN]$_2$	15	1	Et	20	>95	99:1	-
6	1.5	[H-*B*-9-BBN]$_2$	15	1	Et	40	>95	34:66	85:15
7	1.5	[H-*B*-9-BBN]$_2$	15	1	Et	60	>95	21:79	86:14
8	1.5	[H-*B*-9-BBN]$_2$	15	1	^{t}Bu	60	>95	99:1	-
9	1.5	[H-*B*-9-BBN]$_2$	15	1	Ph	60	>95	72:28	71:29
10	1	[H-*B*-9-BBN]$_2$	15	1	Et	60	>95	56:44	86:14
11	1.2	[H-*B*-9-BBN]$_2$	15	1	Et	60	>95	43:57	86:14
12	2	[H-*B*-9-BBN]$_2$	15	1	Et	60	>95	31:69	86:14
13	3	[H-*B*-9-BBN]$_2$	15	1	Et	60	>95	22:78	86:14
14	5	[H-*B*-9-BBN]$_2$	15	1	Et	60	>95	17:83	86:14
15	5	[H-*B*-9-BBN]$_2$	15	1	Et	40	>95	20:80	86:14
16	5	[H-*B*-9-BBN]$_2$	15	5	Et	40	>95	0:100	90:10
17	5	Cy_2BH	15	5	Et	40	>95	100:0	-
18	5	Ipc_2BH	15	5	Et	40	>55	100:0	-
19	1.2[a]	[H-*B*-9-BBN]$_2$	4	1	Et	40	14	100:0	-

Chalcone (1.00 eq.), 9-borabicyclo(3.3.1)nonane [H-*B*-9-BBN]$_2$ (0.15 eq.) and 4,4,5,5-tetramethyl-1,3,2-dioxaborolane (HBpin) (5 eq.) were stirred solution in ester (0.1 M) at 40 °C for 16 hours. The reactions were quenched with ethanolamine and the yield and diastereoselectivity were determined by analysis of the crude reaction mixture by ^{1}H NMR spectroscopy. Ipc_2BH (di*iso*pinocamphenylborane), Cy_2BH (dicyclohexylborane) [a]Reaction with HBdan (1,2,3-trihydro-1,3,2-diazaborinonaphtene) in place of HBpin.

S3 Ester reduction catalysed by dialkylboranes

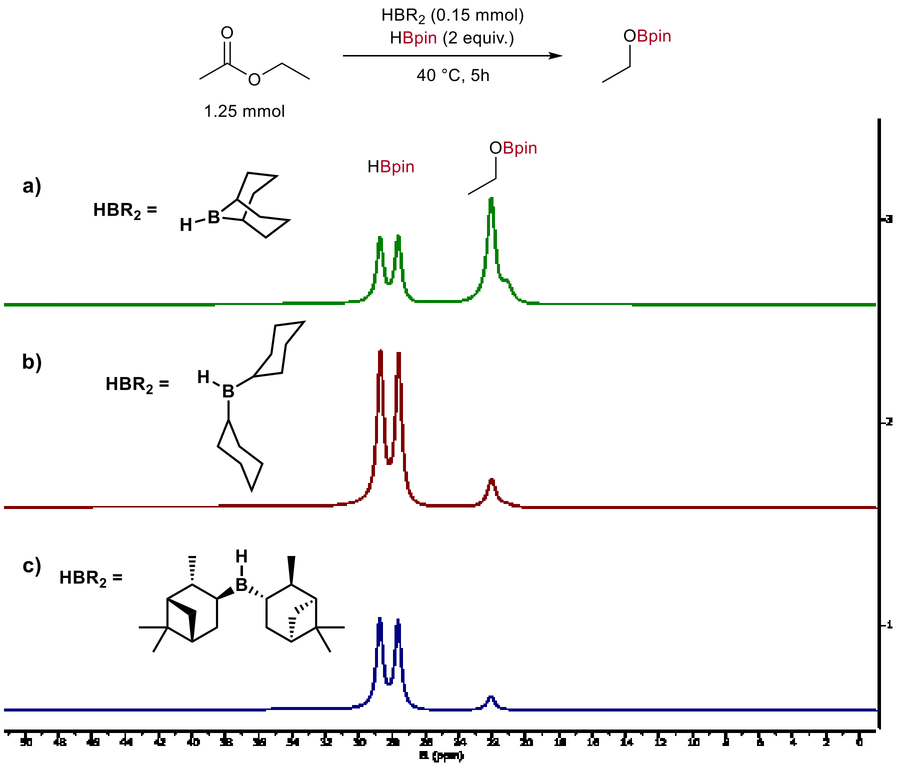

Figure S1: Reduction of ethyl acetate with dialkylboranes. a) H-*B*-9-BBN, b) $(Cy)_2BH$ and c) $(Ipc)_2BH$. Reactions monitored by ^{11}B NMR spectroscopy.

S4 Substrate synthesis

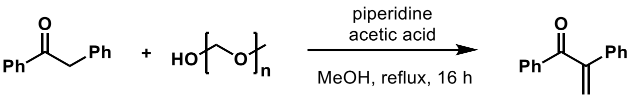

According to the procedure of Laudenschlager[1], to a solution of 2-phenylacetophenone (3.0 g, 15 mmol) in methanol (30 mL), was added formaldehyde (4.8 g, 37% solution of water), piperidine (0.3 mL), and acetic acid (0.25 mL). The mixture was heated under reflux for 16 hours. Upon cooling to room temperature, HCl (25 mL, 1 M) was added and a white precipitate formed. The crude reaction mixture was extracted with EtOAc (3 x 30 mL), the organic phases were washed with saturated $NaHCO_3$ (30 mL) and brine (30 mL), dried ($MgSO_4$), and concentrated under reduced pressure. The crude product was purified by flash column chromatography (CombiFlash Isco NextGen300+, 12 g SiO_2, 50 mm Ø, petroleum ether/ diethyl ether 5:1) to give 1,2-diphenylprop-2-en-1-one as a pale yellow oil (1.4 g, 45%).

^{1}H NMR (500 MHz, $CDCl_3$) 7.98 – 7.91 (m, 2H), 7.62 – 7.53 (m, 1H), 7.49 – 7.43 (m, 4H), 7.41 – 7.32 (m, 3H), 6.10 (s, 1H), 5.68 (s, 1H).

^{13}C NMR (126 MHz, $CDCl_3$) 197.6, 148.3, 137.1, 137.1, 133.1, 130.0, 128.7, 128.5, 128.4, 127.1, 120.9.

All data were in accordance with those previously reported.[1]

S5 General Procedures

General procedure A

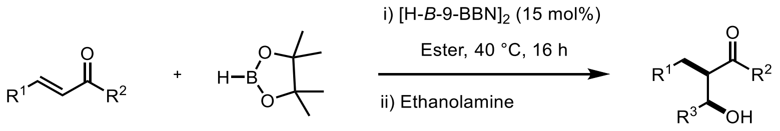

α,β-Unsaturated ketone (1.00 eq.), 9-borabicyclo(3.3.1)nonane [H-*B*-9-BBN]$_2$ (15 mol%) and 4,4,5,5-tetramethyl-1,3,2-dioxaborolane (HBpin) (5 eq.) were stirred in solution of ester (0.1 M) at 40 °C for 16 hours. The reactions were quenched with ethanolamine (0.1 mL, 16.4 mmol) and the diastereoselectivity was determined by analysis of the crude reaction mixture by ^{1}H NMR spectroscopy. The product was purified by flash column chromatography.

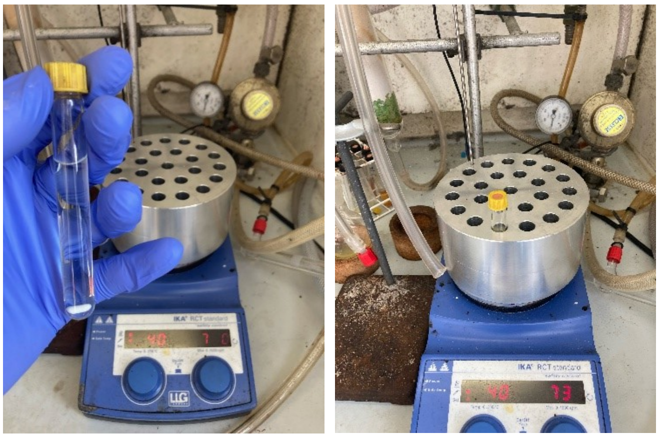

Reaction set-up for the reductive-coupling reaction.

General procedure B

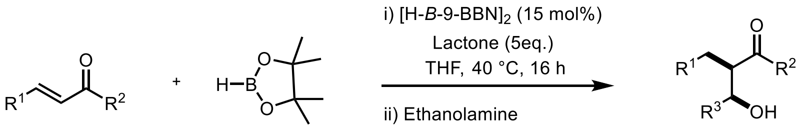

α,β-Unsaturated ketone (1.00 eq.), 9-borabicyclo(3.3.1)nonane [H-*B*-9-BBN]$_2$ (15 mol%), 4,4,5,5-tetramethyl-1,3,2-dioxaborolane (HBpin) (5 eq.) and lactone (5 eq.) were reacted in THF (0.17 M) at 40 °C for 16 hours. The reactions were quenched with ethanolamine (0.1 mL, 16.4 mmol) and the diastereoselectivity was determined by analysis of the crude reaction mixture by ^{1}H NMR spectroscopy. The product was purified by flash column chromatography.

S6 Substrate Scope

(*2RS,3RS*)-2-Benzyl-3-hydroxy-1-phenyl-1-butanone, 3a

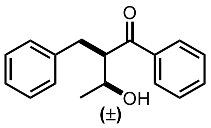

According to general procedure A, chalcone (100 mg, 0.50 mmol), [H-*B*-9-BBN]$_2$ (18 mg, 0.074 mmol) and HBpin (363 µL, 2.5 mmol) were reacted in ethyl acetate (5 mL). After 16 hours ethanolamine was added (0.3 mL, excess). Once effervescence had stopped, the mixture was filtered and concentrated *in vacuo*. The diastereoselectivity was determined by analysis of ^{1}H NMR of the crude reaction mixture using the peaks at 7.72 (*syn*) and 7.86 (*anti*) ppm (*syn*:*anti* 90:10). The crude product was purified by flash column chromatography (CombiFlash Isco NextGen300+, 12 g SiO_2, 50 mm Ø, petroleum ether/ethyl acetate 4:1) to give the alcohol, as a colourless oil (120 mg, 0.47 mmol, 94%, *syn*:*anti* 90:10).

Major diastereomer (*syn*):

^{1}H NMR (500 MHz, $CDCl_3$) 7.69 (dd, *J* = 8.4, 1.3 Hz, 2H), 7.50-7.45 (m, 1H), 7.36-7.31 (m, 2H), 7.17-7.12 (m, 4H), 7.11-7.07 (m, 1H), 4.15 (qd, *J* = 6.3, 4.3 Hz, 1H), 3.77 (app. td, *J* = 7.1, 4.4 Hz, 1H), 3.14 (app. d, *J* = 7.1 Hz, 2H), 1.28 (d, *J* = 6.5 Hz, 3H).

^{13}C NMR (126 MHz, $CDCl_3$) 204.9, 139.5, 137.5, 133.2, 129.0, 128.5, 128.4, 128.4, 126.2, 68.4, 54.5, 33.9, 21.1.

Minor Disatereomer (*anti*):

^{1}H NMR (500 MHz, $CDCl_3$) 7.83 (dd, *J* = 8.4, 1.3 Hz, 2H), 7.56-7.52 (m, 1H), 7.43-7.40 (m, 2H), 7.23-7.20 (m, 5H), 4.00-3.97 (m, 1H), 3.74-3.70 (m, 1H), 3.07 (app. d, *J* = 7.3 Hz, 2H), 1.23 (d, *J* = 6.5 Hz, 3H).

^{13}C NMR (126 MHz, $CDCl_3$) 205.8, 139.0, 137.6, 133.4, 129.1, 128.7, 128.5, 128.3, 126.5, 68.4, 53.8, 36.1, 22.1.

Data were in accordance with those previously reported.[2]

(*2RS,3RS*)-2-Benzyl-3-hydroxy-1-phenyl-1-butanone, 3a

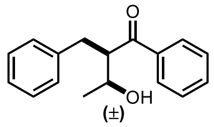

According to general procedure B, chalcone (100 mg, 0.50 mmol), [H-*B*-9-BBN]$_2$ (18 mg, 0.074 mmol), HBpin (363 µL, 2.5 mmol) and acetic anhydride (237 µL, 2.5 mmol) were reacted in THF (3 mL). After 16 hours ethanolamine was added (0.3 mL, excess). Once effervescence had stopped, the mixture was filtered and concentrated *in vacuo*. The crude product was purified by flash column chromatography (CombiFlash Isco NextGen300+, 12 g SiO_2, 50 mm Ø, petroleum ether/ethyl acetate 4:1) to give the alcohol, as a colourless oil (51 mg, 0.20 mmol, 40%, single diastereomer).

^{1}H NMR (500 MHz, $CDCl_3$) 7.69 (dd, J = 8.4, 1.3 Hz, 2H), 7.50-7.45 (m, 1H), 7.36-7.31 (m, 2H), 7.17-7.12 (m, 4H), 7.11-7.07 (m, 1H), 4.15 (qd, J = 6.3, 4.3 Hz, 1H), 3.77 (app. td, J = 7.1, 4.4 Hz, 1H), 3.14 (app. d, J = 7.1 Hz, 2H), 1.28 (d, J = 6.5 Hz, 3H).

^{13}C NMR (126 MHz, $CDCl_3$) 204.9, 139.5, 137.5, 133.2, 129.0, 128.5, 128.4, 128.4, 126.2, 68.4, 54.5, 33.9, 21.1.

(*2RS,3RS*)-2-Benzyl-3-hydroxy-1-phenyl-1-butanone, 3a

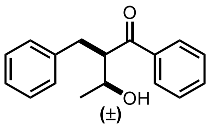

According to general procedure B, chalcone (100 mg, 0.50 mmol), [H-*B*-9-BBN]$_2$ (18 mg, 0.074 mmol), HBpin (363 µL, 2.5 mmol) and carboxylic acid (726 µL, 2.5 mmol) were reacted in THF (3 mL). After 16 hours ethanolamine was added (0.3 mL, excess). Once effervescence had stopped, the mixture was filtered and concentrated *in vacuo*. The crude product was purified by flash column chromatography (CombiFlash Isco NextGen300+, 12 g SiO_2, 50 mm Ø, petroleum ether/ethyl acetate 4:1) to give the alcohol, as a colourless oil (15 mg, 0.06 mmol, 12%, single diastereomer).

^{1}H NMR (500 MHz, $CDCl_3$) 7.69 (dd, *J* = 8.4, 1.3 Hz, 2H), 7.50-7.45 (m, 1H), 7.36-7.31 (m, 2H), 7.17-7.12 (m, 4H), 7.11-7.07 (m, 1H), 4.15 (qd, *J* = 6.3, 4.3 Hz, 1H), 3.77 (app. td, *J* = 7.1, 4.4 Hz, 1H), 3.14 (app. d, *J* = 7.1 Hz, 2H), 1.28 (d, *J* = 6.5 Hz, 3H).

^{13}C NMR (126 MHz, $CDCl_3$) 204.9, 139.5, 137.5, 133.2, 129.0, 128.5, 128.4, 128.4, 126.2, 68.4, 54.5, 33.9, 21.1.

(*2RS,3RS*)-2-Benzyl-3-hydroxy-1-phenyl-1-pentanone, 3b

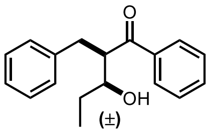

According to general procedure A, chalcone (100 mg, 0.50 mmol), [H-*B*-9-BBN]$_2$ (18 mg, 0.074 mmol) and HBpin (363 µL, 2.5 mmol) were reacted in ethyl propionate (5 mL). After 16 hours ethanolamine was added (0.3 mL, excess). Once effervescence had stopped, the mixture was filtered and concentrated *in vacuo*. The diastereoselectivity was determined by analysis of ^{1}H NMR of the crude reaction mixture using the peaks at 7.69 (*syn*) and 7.84 (*anti*) ppm (*syn*:*anti* 90:10). The crude product was purified by flash column chromatography (CombiFlash Isco NextGen300+, 12 g SiO_2, 50 mm Ø, petroleum ether/ethyl acetate 4:1) to give the *alcohol*, as a colourless oil (115 mg, 0.43 mmol, 86%, single diastereomer).

^{1}H NMR (500 MHz, $CDCl_3$) 7.68 (dd, *J* = 8.4, 1.3 Hz, 2H), 7.53-7.45 (m, 1H), 7.38-7.30 (m, 2H), 7.21-7.11 (m, 4H), 7.11-7.06 (m, 1H), 3.88-3.79 (m, 2H), 3.19 (dd, *J* = 13.7, 9.3 Hz, 1H), 3.14 (dd, *J* = 13.7, 4.4 Hz, 1H), 2.65 (d, *J* = 3.1 Hz, 1H), 1.68-1.52 (m, 2H), 1.00 (t, *J* = 7.4 Hz, 3H).

^{13}C NMR (126 MHz, $CDCl_3$) 204.9, 139.7, 137.4, 133.2, 129.0, 128.5, 128.4, 128.3, 126.2, 73.7, 52.9, 33.5, 27.8, 10.5.

HRMS (EI^+) Calcd for $C_{18}H_{20}O_2$ 268.1458; Found 268.1470.

IR ν_{max} (neat) 3452 (w, br), 3028 (w), 2962 (w), 2932 (w), 1670 (s).

(*2RS,3RS*)-2-Benzyl-3-hydroxy-1-phenyl-1-hexanone, 3c

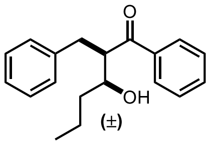

According to general procedure A, chalcone (100 mg, 0.50 mmol), [H-*B*-9-BBN]$_2$ (18 mg, 0.074 mmol) and HBpin (363 µL, 2.5 mmol) were reacted in ethyl butyrate (5 mL). After 16 hours ethanolamine was added (0.3 mL, excess). Once effervescence had stopped, the mixture was filtered and concentrated *in vacuo*. The diastereoselectivity was determined by analysis of ^{1}H NMR of the crude reaction mixture using the peaks at 7.68 (*syn*) and 7.82 (*anti*) ppm (*syn*:*anti* 91:9). The crude product was purified by flash column chromatography (CombiFlash Isco NextGen300+, 12 g SiO_2, 50 mm Ø, petroleum ether/ethyl acetate 4:1) to give the alcohol, as a colourless oil (82 mg, 0.29 mmol, 58%, single diastereomer).

^{1}H NMR (500 MHz, $CDCl_3$) 7.70-7.66 (m, 2H), 7.50-7.45 (m, 1H), 7.33 (dd, *J* = 8.3, 7.3 Hz, 2H), 7.18-7.11 (m, 4H), 7.10-7.06 (m, 1H), 3.94 (m, 1H), 3.80 (app. dt, *J* = 9.6, 4.3 Hz, 1H), 3.16 (dd, *J* = 13.7, 9.7 Hz, 1H), 3.11 (dd, *J* = 13.7, 4.6 Hz, 1H), 2.63 (br. s, 1H), 1.67-1.35 (m, 4H), 0.92 (t, *J* = 7.1 Hz, 3H).

^{13}C NMR (126 MHz, $CDCl_3$) 205.0, 139.7, 137.4, 133.2, 129.0, 128.5, 128.4, 128.3, 126.2, 71.9, 53.3, 37.0, 33.5, 19.3, 14.0.

HRMS (EI^+) Calcd for $C_{19}H_{22}O_2$ 282.1614; Found 282.1613.

IR ν_{max} (neat) 3438 (w, br), 2958 (w), 2932 (w), 2872 (w), 1672 (s).
Data were in accordance with those previously reported.[3]

(*2RS,3RS*)-2-Benzyl-3-hydroxy-5-methyl-1-phenyl-1-hexanone, 3d

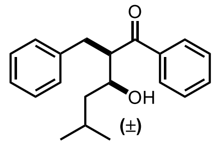

According to general procedure A, chalcone (100 mg, 0.50 mmol), [H-*B*-9-BBN]$_2$ (18 mg, 0.074 mmol) and HBpin (363 μL, 2.5 mmol) were reacted in ethyl isovalerate (5 mL). After 16 hours ethanolamine was added (0.3 mL, excess). Once effervescence had stopped, the mixture was filtered and concentrated *in vacuo*. The diastereoselectivity was determined by analysis of ^{1}H NMR of the crude reaction mixture using the peaks at 7.81 (*syn*) and 7.68 (*anti*) ppm (*syn*:*anti* 93:7).The crude product was purified by flash column chromatography (CombiFlash Isco NextGen300+, 12 g SiO_2, 50 mm Ø, petroleum ether/ethyl acetate 4:1) to give the alcohol, as colourless needles (127 mg, 0.43 mmol, 82%, single diastereomer).

^{1}H NMR (500 MHz, $CDCl_3$) 7.68 (dd, *J* = 8.4, 1.3 Hz, 2H), 7.50-7.46 (m, 1H), 7.36-7.30 (m, 2H), 7.18-7.11 (m, 4H), 7.10-7.06 (m, 1H), 4.05-4.00 (m, 1H), 3.77 (ddd, *J* = 9.6, 4.7, 3.7 Hz, 1H), 3.18 (dd, *J* = 13.7, 9.6 Hz, 1H), 3.13 (dd, *J* = 13.7, 4.7 Hz, 1H), 2.65-2.60 (m, 1H), 1.88-1.79 (m, 1H), 1.64-1.56 (m, 1H), 1.31-1.24 (m, 1H) 0.93 (d, *J* = 6.7 Hz, 3H), 0.91 (d, *J* = 6.6 Hz, 3H).

^{13}C NMR (126 MHz, $CDCl_3$) 205.1, 139.7, 137.4, 133.2, 129.0, 128.5, 128.4, 128.4, 126.2, 70.1, 53.6, 43.9, 33.6, 24.8, 23.5, 21.9.

m.p. 84-86 °C (CH_2Cl_2), Lit.; 64–66 °C (diethyl ether)

Data were in accordance with those previously reported.[4]

(*2RS,3RS*)-2-Benzyl-3-hydroxy-5,5-dimethyl-1-phenyl-1-hexanone, 3e

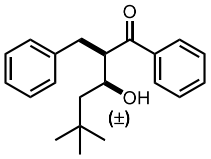

According to general procedure A, chalcone (100 mg, 0.50 mmol), [H-*B*-9-BBN]$_2$ (18 mg, 0.074 mmol) and HBpin (363 μL, 2.5 mmol) were reacted in ethyl tert-butylacetate (5 mL). After 16 hours ethanolamine was added (0.3 mL, excess). Once effervescence had stopped, the mixture was filtered and concentrated *in vacuo*. The diastereoselectivity was determined by analysis of ^{1}H NMR of the crude reaction mixture using the peaks at 7.68 (*syn*) and 7.81 (*anti*) ppm (*syn*:*anti* 84:16).The crude product was purified by flash column chromatography (CombiFlash Isco NextGen300+, 12 g SiO_2, 50 mm Ø, petroleum ether/ethyl acetate 4:1) to give the *alcohol*, as colourless microcrystalline (68 mg, 0.22 mmol, 44%, single diastereomer).

^{1}H NMR (500 MHz, $CDCl_3$) 7.67 (dd, *J* = 8.5, 1.3 Hz, 2H), 7.52-7.42 (m, 1H), 7.38-7.29 (m, 2H), 7.17-7.10 (m, 4H), 7.09-7.05 (m, 1H), 4.09 (m, 1H), 3.73 (app. dt, *J* = 10.0, 4.0 Hz, 1H), 3.17 (dd, *J* = 13.7, 10.0 Hz, 1H), 3.09 (dd, *J* = 13.7, 4.2 Hz, 1H), 2.55 (br. s, 1H), 1.58 (dd, *J* = 14.4, 8.6 Hz, 1H), 1.41 (dd, *J* = 14.4, 2.0 Hz, 1H), 0.96 (s, 9H).

^{13}C NMR (126 MHz, $CDCl_3$) 205.0, 139.7, 137.5, 133.2, 129.0, 128.5, 128.4, 128.4, 126.2, 69.7, 55.0, 49.1, 33.6, 30.4, 30.0.

HRMS (EI^+) Calcd for $C_{21}H_{26}O_2$ 310.1927; Found 310.1930.

IR ν_{max} (neat) 3511 (w, br), 2954 (w), 2934 (w), 2904 (w), 1657 (s).

m.p. 101-103 °C (CH_2Cl_2)

(*2RS,3RS*)-2-Benzyl-3-cyclobutyl-3-hydroxy-1-phenyl-1-propanone, 3f

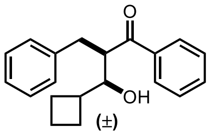

According to general procedure A, chalcone (100 mg, 0.50 mmol), [H-*B*-9-BBN]$_2$ (18 mg, 0.074 mmol) and HBpin (363 μL, 2.5 mmol) were reacted in ethyl cyclobutylcarboxylate (5 mL). After 16 hours ethanolamine was added (0.3 mL, excess). Once effervescence had stopped, the mixture was filtered and concentrated *in vacuo*. The diastereoselectivity was determined by analysis of ^{1}H NMR of the crude reaction mixture using the peaks at 7.66 (*syn*) and 7.77 (*anti*) ppm (*syn*:*anti* 82:18). The crude product was purified by flash column chromatography (CombiFlash Isco NextGen300+, 12 g SiO_2, 50 mm Ø, petroleum ether/ethyl acetate 4:1) to give the *alcohol*, as a colourless oil (125 mg, 0.43 mmol, 85%, single diastereomer).

^{1}H NMR (500 MHz, $CDCl_3$) 7.68 (dd, J = 8.4, 1.3 Hz, 2H), 7.50-7.46 (m, 1H), 7.37-7.31 (m, 2H), 7.18-7.10 (m, 4H), 7.10-7.05 (m, 1H), 3.86 (dd, J = 8.1, 3.7 Hz, 1H), 3.78 (app. dt, J = 10.0, 3.9 Hz, 1H), 3.15 (dd, J = 13.7, 10 Hz, 1H), 3.06 (dd, J = 13.7, 4.1 Hz, 1H), 2.65 (s, 1H), 2.57 (m, 1H), 2.09-2.01 (m, 1H), 1.98-1.74 (m, 5H).

^{13}C NMR (126 MHz, $CDCl_3$) 204.7, 139.8, 137.2, 133.2, 129.0, 128.5, 128.4, 128.3, 126.1, 76.3, 50.8, 39.0, 33.3, 25.3, 24.7, 18.0.

HRMS (EI$^+$) Calcd for $C_{20}H_{22}O_2$ 294.1614; Found 294.1602.

IR ν_{max} (neat) 3392 (w, br), 2982 (w), 2932 (w), 2920 (w), 2860 (w), 1673 (s).

(*2RS,3RS*)-2-Benzyl-3-cyclopropyl-3-hydroxy-1-phenyl-1-propanone, 3g

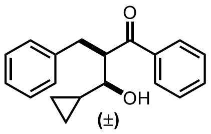

According to general procedure A, chalcone (100 mg, 0.50 mmol), [H-*B*-9-BBN]$_2$ (18 mg, 0.074 mmol) and HBpin (363 µL, 2.5 mmol) were reacted in ethyl cyclobutylcarboxylate (5 mL). After 16 hours ethanolamine was added (0.3 mL, excess). Once effervescence had stopped, the mixture was filtered and concentrated *in vacuo*. The diastereoselectivity was determined by analysis of ^{1}H NMR of the crude reaction mixture using the peaks at 7.71 (*syn*) and 7.84 (*anti*) ppm (*syn*:*anti* 89:11). The crude product was purified by flash column chromatography (CombiFlash Isco NextGen300+, 12 g SiO_2, 50 mm Ø, petroleum ether/ethyl acetate 4:1) to give the *alcohol*, as a colourless oil (70 mg, 0.25 mmol, 50%, single diastereomer).

^{1}H NMR (500 MHz, $CDCl_3$) 7.71 (dd, *J* = 8.2, 1.4 Hz, 2H), 7.49-7.44 (m, 1H), 7.33 (dd, *J* = 8.3, 7.3 Hz, 2H), 7.19-7.15 (m, 4H), 7.11-7.05 (m, 1H), 4.05-3.97 (m, 1H), 3.26 (dd, *J* = 13.6, 4.4 Hz, 1H), 3.24-3.14 (m, 2H), 2.44 (br. s, 1H), 1.04-0.94 (m, 1H), 0.56-0.49 (m, 1H), 0.46-0.31 (m, 2H), 0.27-0.19 (m, 1H).

^{13}C NMR (126 MHz, $CDCl_3$) 204.1, 139.8, 138.0, 133.0z, 129.0, 128.4, 128.4, 128.3, 126.1, 54.2, 34.6, 16.2, 3.7, 3.5.

HRMS (EI^+) Calcd for $C_{19}H_{20}O_2$ 280.1458; Found 280.1469.

IR ν_{max} (neat) 3414 (w), 2931 (w), 1654 (s).

(*2RS,3RS*)-3-Hydroxy-2-(4-methoxybenzyl)-1-phenyl-1-butanone, 3h

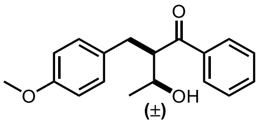

According to general procedure A, 4-methoxychalcone (119 mg, 0.50 mmol), [H-*B*-9-BBN]$_2$ (18 mg, 0.074 mmol) and HBpin (363 µL, 2.5 mmol) were reacted in ethyl acetate (5 mL). After 16 hours ethanolamine was added (0.3 mL, excess). Once effervescence had stopped, the mixture was filtered and concentrated *in vacuo*. The diastereoselectivity was determined by analysis of ^{1}H NMR of the crude reaction mixture using the peaks at 7.71 (*syn*) and 7.84 (*anti*) ppm (*syn*:*anti* 93:7). The crude product was purified by flash column chromatography (CombiFlash Isco NextGen300+, 12 g SiO_2, 50 mm Ø, petroleum ether/ethyl acetate 4:1) to give the *alcohol*, as a pale yellow oil (108 mg, 0.38 mmol, 76%, *syn:anti* 91:9).

Major diastereomer (*syn*):

^{1}H NMR (500 MHz, $CDCl_3$) 7.70 (dd, *J* = 8.4, 1.3 Hz, 2H), 7.51-7.46 (m, 1H), 7.38-7.32 (m, 2H), 7.04 (d, *J* = 8.6, 2H), 6.71 (d, *J* = 8.6 Hz, 2H), 4.13 (qd, *J* = 6.4, 4.4 Hz, 1H), 3.76-3.72 (m, 1H), 3.71 (s, 3H), 3.10-3.06 (m, 2H), 1.26 (d, *J* = 6.4 Hz, 3H).

^{13}C NMR (126 MHz, $CDCl_3$) 205.0, 158.0, 137.6, 133.1, 131.5, 129.4, 128.5, 128.4, 113.9, 68.4, 55.2, 54.7, 33.0, 21.1.

Minor diastereomer (*anti*):

^{1}H NMR (500 MHz, $CDCl_3$) 7.85-7.83 (m, 2H), 7.58-7.52 (m, 1H), 7.45-7.41 (m, 2H), 7.12 (d, *J* = 8.6, 2H), 6.77 (d, *J* = 8.6, 2H), 4.02-3.96 (m, 1H), 3.71-3.66 (obsc., 1H), 3.75 (s, 3H), 3.03-2.99 (m, 2H), 1.22 (d, *J* = 6.4, 3H).

^{13}C NMR (126 MHz, $CDCl_3$) 205.9, 158.2, 137.6 (obsc.), 133.4, 131.1, 130.1, 128.7, 128.3, 113.9, 68.3, 55.2, 54.1, 35.2, 22.1.

HRMS (EI^+) Calcd for $C_{18}H_{20}O_3$ 284.1407; Found 284.1406.

IR ν_{max} (neat) 3444 (w, br), 2968 (w), 2932 (w), 2911 (w), 2835 (w), 1672 (s), 1245 (s).

(*2RS,3RS*)-3-Hydroxy-2-(4-methoxybenzyl)-1-phenyl-1-pentanone, 3i

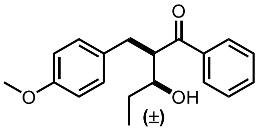

According to general procedure A, 4-methoxychalcone (119 mg, 0.50 mmol), [H-*B*-9-BBN]$_2$ (18 mg, 0.074 mmol) and HBpin (363 µL, 2.5 mmol) were reacted in ethyl propionate (5 mL). After 16 hours ethanolamine was added (0.3 mL, excess). Once effervescence had stopped, the mixture was filtered and concentrated *in vacuo*. The diastereoselectivity was determined by analysis of ^{1}H NMR of the crude reaction mixture using the peaks at 7.70 (*syn*) and 7.84 (*anti*) ppm (*syn*:*anti* 90:10). The crude product was purified by flash column chromatography (CombiFlash Isco NextGen300+, 12 g SiO_2, 50 mm Ø, petroleum ether/ethyl acetate 4:1) to give the *alcohol*, as a colourless oil (118 mg, 0.40 mmol, 79%, *syn*:*anti* >20:1).

^{1}H NMR (500 MHz, $CDCl_3$) 7.70 (dd, *J* = 8.4, 1.3 Hz, 2H), 7.50-7.45 (m, 1H), 7.34 (dd, *J* = 8.3, 7.3 Hz, 2H), 7.04 (d, *J* = 8.6 Hz, 2H), 6.70 (d, *J* = 8.7 Hz, 2H), 3.86-3.78 (m, 2H), 3.70 (s, 3H), 3.11 (dd, 13.8, 9.4 Hz, 1H), 3.06 (dd, *J* = 13.8, 4.4 Hz, 1H), 2.71 (br. s, 1H), 1.64-1.50 (m, 2H), 0.99 (t, *J* = 7.4 Hz, 3H).

^{13}C NMR (126 MHz, $CDCl_3$) 205.1, 158.0, 137.5, 133.1, 131.6, 130.0, 128.5, 128.4, 113.9, 73.7, 55.2, 53.2, 32.8, 27.9, 10.5.

HRMS (EI$^+$) Calcd for $C_{19}H_{22}O_3$ 298.1564; Found 298.1563.

IR ν_{max} (neat) 3502 (w, br), 2962 (w), 2934 (w), 2877 (w), 2835 (w), 1672 (s), 1245 (s).

(*2RS,3RS*)-3-Hydroxy-2-(3,4-dimethoxybenzyl)-1-phenyl-1-pentanone, 3j

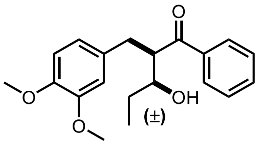

According to general procedure A, 3,4-dimethoxychalcone (134 mg, 0.50 mmol), [H-*B*-9-BBN]$_2$ (18 mg, 0.074 mmol) and HBpin (363 µL, 2.5 mmol) were reacted in ethyl propionate (5 mL). After 16 hours ethanolamine was added (0.3 mL, excess). Once effervescence had stopped, the mixture was filtered and concentrated *in vacuo*. The diastereoselectivity was determined by analysis of ^{1}H NMR of the crude reaction mixture using the peaks at 7.70 (*syn*) and 7.82 (*anti*) ppm (*syn*:*anti* 87:13). The crude product was purified by flash column chromatography (CombiFlash Isco NextGen300+, 12 g SiO_2, 50 mm Ø, petroleum ether/ethyl acetate 4:1) to give the *alcohol*, as a yellow oil (151 mg, 0.46 mmol, 92%, *syn*:*anti* 88:12).

Major diastereomer (*syn*):

^{1}H NMR (500 MHz, $CDCl_3$) 7.70 (dd, *J* = 8.4, 1.3 Hz, 2H), 7.51-7.46 (m, 1H), 7.35 (dd, *J* = 8.3, 7.4 Hz, 2H), 6.68-6.66 (m, 2H), 6.59 (s, 1H), 3.86-3.81 (m, 1H), 3.81-3.78 (m, 1H) 3.78 (s, 3H), 3.71 (s, 3H), 3.11 (dd, *J* = 13.8, 9.5 Hz, 1H), 3.07 (dd, *J* = 13.8, 4.6 Hz, 1H), 1.65-1.54 (m, 2H), 1.00 (t, *J* = 7.4 Hz, 3H).

^{13}C NMR (126 MHz, $CDCl_3$) 205.2, 148.7, 147.4, 137.5, 133.2, 132.2, 128.5, 128.3, 120.9, 112.4, 111.3, 73.8z, 55.9, 55.7, 53.0, 33.4, 27.8, 10.5.

Minor diastereomer (*anti*):

^{1}H NMR (500 MHz, $CDCl_3$) 7.80-7.77 (m, 2H), 7.53-7.48 (m, 1H), 7.40-7.36 (m, 2H), 6.75-6.69 (m, 2H), 6.64-6.61 (obsc., 1H), 3.85-3.65 (m, 8H), 3.05-3.00 (m, 2H), 1.50-1.43 (m, 2H), 0.91 (t, *J* = 7.4 Hz, 3H).

^{13}C NMR (126 MHz, $CDCl_3$) 206.4, 148.8, 147.6, 137.8, 133.5, 131.6, 128.7, 128.2, 121.1, 112.5, 111.4, 74.3, 55.9, 55.8 (obsc.), 52.2, 36.1, 29.0, 10.6.

HRMS (EI^+) Calcd for $C_{20}H_{24}O_4$ 328.1669; Found 328.1666.

IR ν_{max} (neat) 3484 (w, br), 2961 (w), 2935 (w), 2875 (w), 2835 (w), 1672 (s), 1261 (s), 1235 (s).

(*2RS,3RS*)-3-Hydroxy-2-(3,4-dimethoxybenzyl)-1-phenyl-1-hexanone, 3k

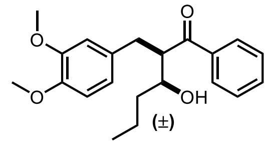

According to general procedure A, 3,4-dimethoxychalcone (134 mg, 0.50 mmol), [H-*B*-9-BBN]$_2$ (18 mg, 0.074 mmol) and HBpin (363 µL, 2.5 mmol) were reacted in ethyl butyrate (5 mL). After 16 hours ethanolamine was added (0.3 mL, excess). Once effervescence had stopped, the mixture was filtered and concentrated *in vacuo*. The diastereoselectivity was determined by analysis of ^{1}H NMR of the crude reaction mixture using the peaks at 7.71 (*syn*) and 7.83 (*anti*) ppm (*syn*:*anti* 85:15). The crude product was purified by flash column chromatography (CombiFlash Isco NextGen300+, 12 g SiO_2, 50 mm Ø, petroleum ether/ethyl acetate 4:1) to give the *alcohol*, as a brown oil (0.130 g, 0.38 mmol, 76%, *syn*:*anti* 85:15).

Major diastereomer (*syn*):

^{1}H NMR (500 MHz, $CDCl_3$) 7.69 (dd, *J* = 8.4, 1.3 Hz, 2H), 7.51-7.45 (m, 1H), 7.35 (m, 2H), 6.68-6.66 (m, 2H), 6.59 (s, 1H), 3.96-3.90 (m, 1H), 3.86-3.83 (m, 1H) 3.77 (s, 3H), 3.71 (s, 3H), 3.14-3.06 (m, 2H), 2.71 (br. s, 1H), 1.64-1.35 (m, 4H), 0.92 (t, *J* = 7.1 Hz, 3H).

^{13}C NMR (126 MHz, $CDCl_3$) 205.2, 148.7, 147.4, 137.6, 133.2, 132.2, 128.5, 128.3, 121.0, 112.5, 111.4, 72.0, 55.9, 55.8, 53.5, 37.1, 33.5, 19.3, 14.0.

Minor diastereomer (*anti*):

^{1}H NMR (500 MHz, $CDCl_3$) 7.81 (dd, *J* = 8.3, 1.3 Hz, 2H), 7.55-7.51 (m, 1H), 7.42-7.38 (m, 2H), 6.75-6.72 (m, 2H), 6.68-6.64 (obsc., 1H), 3.96-3.75 (m, 5H), 3.81 (s, 3H), 3.06-3.02 (m, 2H), 1.63-1.34 (obsc., 4H), 0.84 (t, *J* = 7.2 Hz, 3H).

^{13}C NMR (126 MHz, $CDCl_3$) 206.5, 148.8, 147.6, 137.7, 133.5, 131.6, 128.7, 128.2, 121.1, 112.5, 111.4, 72.6, 55.9, 55.9, 52.4, 38.3, 36.2, 19.4, 13.9.

HRMS (EI^+) Calcd for $C_{21}H_{26}O_4$ 342.1826; Found 342.1830.

IR ν_{max} (neat) 3504 (w, br), 2957 (w), 2934 (w), 2871 (w), 2835 (w), 1672 (s), 1261 (s), 1235 (s).

(*2RS,3RS*)-3-Hydroxy-2-(3,4-(dimethoxy)benzyl)-1-phenyl-1-butanone, 3l

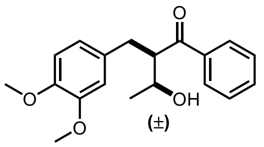

According to general procedure A, 3,4-(dimethoxy)chalcone (134 mg, 0.50 mmol), [H-*B*-9-BBN]$_2$ (18 mg, 0.074 mmol) and HBpin (363 µL, 2.5 mmol) were reacted in ethyl acetate (5 mL). After 16 hours ethanolamine was added (0.3 mL, excess). Once effervescence had stopped, the mixture was filtered and concentrated *in vacuo*. The diastereoselectivity was determined by analysis of ^{1}H NMR of the crude reaction mixture using the peaks at 7.73 (*syn*) and 7.84 (*anti*) ppm (*syn*:*anti* 92:8). The crude product was purified by flash column chromatography (CombiFlash Isco NextGen300+, 12 g SiO_2, 50 mm Ø, petroleum ether/ethyl acetate 4:1) to give the *alcohol*, as a yellow oil (121 mg, 0.39 mmol, 77%, *syn:anti* 92:8).

Major diastereomer (*syn*):

^{1}H NMR (500 MHz, $CDCl_3$) 7.69 (dd, J = 8.4, 1.3 Hz, 2H), 7.48-7.44 (m, 1H), 7.34-7.32 (m, 2H), 6.66-6.64 (m, 2H), 6.58 (d, J = 1.6 Hz, 2H), 4.15-4.09 (m, 1H), 3.75 (s, 3H), 3.74-3.70 (m, 1H), 3.69 (s, 3H), 3.10-3.07 (m, 2H), 1.24 (d, J = 6.3 Hz, 3H).

^{13}C NMR (126 MHz, $CDCl_3$) 205.0, 148.7, 147.4, 137.7, 133.2, 132.1, 128.7, 128.5, 128.3, 120.9, 112.4, 111.3, 68.5, 55.8, 55.7, 54.8, 34.0, 24.8, 21.3.

Minor diastereomer (*anti*):

^{1}H NMR (500 MHz, $CDCl_3$) 7.82-7.79 (m, 2H), 7.53-7.49 (m, 1H), 7.41-7.36 (m, 2H), 6.72-6.70 (m, 2H), 6.67 (d, J = 1.6, 2H), 4.06-4.01 (m, 1H), 3.78 (s, 3H), 3.74-3.70 (obsc., 1H), 3.70 (s, 3H), 3.02-2.98 (m, 2H), 1.24 (obsc., 3H).

^{13}C NMR (126 MHz, $CDCl_3$) 205.9zz, 148.8, 147.6, 137.8, 133.4, 131.5, 128.7, 128.3, 121.1, 112.5, 111.4, 68.7, 55.9, 55.8, 54.1, 35.7, 24.7, 22.0.

HRMS (EI$^+$) Calcd for $C_{19}H_{22}O_4$ 314.1513; Found 314.1526.

IR ν_{max} (neat) 3507 (w, br), 2962 (w), 2955 (w), 2935 (w), 1657 (s).

(*2RS,3RS*)-2-Hydroxy-2-(2,4-dimethoxy-6-methyl)benzyl)-1-phenyl-1-butanone, 3m

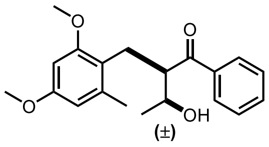

According to general procedure A, (*E*)-2,4-dimethoxy-6-methyl-chalcone (141 mg, 0.50 mmol), [H-*B*-9-BBN]$_2$ (18 mg, 0.074 mmol) and HBpin (363 µL, 2.5 mmol) were reacted in ethyl acetate (5 mL). After 16 hours ethanolamine was added (0.3 mL, excess). Once effervescence had stopped, the mixture was filtered and concentrated *in vacuo*. The diastereoselectivity was determined by analysis of ^{1}H NMR of the crude reaction mixture using the peaks at 7.65 (*syn*) and 7.87 (*anti*) ppm (*syn*:*anti* 87:13). The crude product was purified by flash column chromatography (CombiFlash Isco NextGen300+, 12 g SiO_2, 50 mm Ø, petroleum ether/ethyl acetate 4:1) to give the *alcohol*, as a yellow oil (145 mg, 0.44 mmol, 88%, single diastereomer).

^{1}H NMR (500 MHz, $CDCl_3$) 7.66 (dd, *J* = 8.4, 1.3 Hz, 2H), 7.48-7.43 (m, 1H), 7.30 (dd, *J* = 8.3, 7.3 Hz, 2H), 6.21 (d, *J* = 2.5 Hz, 1H), 6.14 (d, *J* = 2.4 Hz, 1H), 4.20 (qd, *J* = 6.3, 4.0 Hz, 1H), 3.76-3.70 (m, 4H), 3.62 (s, 3H), 3.13 (dd, *J* = 13.9, 4.7 Hz, 1H), 3.07 (dd, *J* = 13.9, 9.0 Hz, 1H), 2.23 (s, 3H), 1.29 (d, *J* = 6.4 Hz, 3H).

^{13}C NMR (126 MHz, $CDCl_3$) 206.5, 158.7, 158.4, 138.2, 137.5, 132.9, 128.2, 128.2, 118.8, 106.7, 95.9, 68.7, 55.2, 55.0, 51.2, 24.4, 20.8, 20.2.

HRMS (EI^+) Calcd for $C_{20}H_{24}O_4$ 328.1669; Found 328.1655.

IR ν_{max} (neat) 3460 (w, br), 2937 (w), 2837 (w), 1672 (s), 1201 (s), 1147 (s).

(*2RS,3RS*)-3-Hydroxy-2-(4-thiomethoxybenzyl)-1-phenyl-1-butanone, 3n

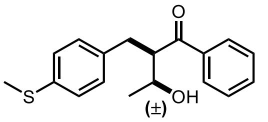

According to general procedure A, 4-thiomethoxychalcone (127gm, 0.50 mmol), [H-*B*-9-BBN]$_2$ (18 mg, 0.074 mmol) and HBpin (363 µL, 2.5 mmol) were reacted in ethyl acetate (5 mL). After 16 hours ethanolamine was added (0.3 mL, excess). Once effervescence had stopped, the mixture was filtered and concentrated *in vacuo*. The diastereoselectivity was determined by analysis of ^{1}H NMR of the crude reaction mixture using the peaks at 7.72 (*syn*) and 7.85 (*anti*) ppm (*syn*:*anti* 91:9). The crude product was purified by flash column chromatography (CombiFlash Isco NextGen300+, 12 g SiO_2, 50 mm Ø, petroleum ether/ethyl acetate 4:1) to give the *alcohol*, as a colourless oil (111 mg, 0.37 mmol, 74%, *syn:anti* 92:8).

Major diastereomer (*syn*):

^{1}H NMR (500 MHz, $CDCl_3$) 7.71 (dd, *J* = 8.4, 1.3 Hz, 2H), 7.50-7.46 (m, 1H), 7.36-7.32 (m, 2H), 7.08-7.03 (m, 4H), 4.16-4.09 (m, 1H), 3.78-3.73 (m, 1H), 3.13-3.08 (m, 2H), 2.64 (br. s, 1H), 2.38 (s, 3H), 1.25 (d, *J* = 6.4 Hz, 3H).

^{13}C NMR (126 MHz, $CDCl_3$) 204.6, 137.5, 136.6, 135.9, 133.3, 129.5, 128.5, 128.4, 127.1, 68.4, 54.5, 33.3, 21.2, 16.2.

Minor diastereomer (*anti*):

^{1}H NMR (500 MHz, $CDCl_3$) 7.84 (dd, *J* = 8.4, 1.3 Hz, 2H), 7.56-7.52 (m, 1H), 7.44-7.39 (m, 2H), 7.13 (s, 4H), 4.03-3.96 (m, 1H), 3.73-3.69 (m, 1H), 3.04-2.99 (m, 2H), 2.64 (br. s, 1H), 2.41 (s, 3H), 1.22 (d, *J* = 6.4 Hz, 3H).

^{13}C NMR (126 MHz, $CDCl_3$) 205.5, 137.6 (obsc.), 136.2, 136.0, 133.5, 129.7, 128.7, 128.3, 127.1 (obsc.), 68.3, 53.8, 35.3, 22.0, 16.1.

HRMS (EI^+) Calcd for $C_{18}H_{20}O_2S$ 300.1179; Found 300.1190.

IR ν_{max} (neat) 3417 (w, br), 2971 (w), 2921 (w), 1672 (s).

(*2RS,3RS*)-3-Hydroxy-2-(4-thiomethoxybenzyl)-1-phenyl-1-pentanone, 3o

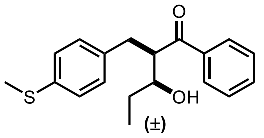

According to general procedure A, 4-thiomethoxychalcone (127 mg, 0.50 mmol), [H-*B*-9-BBN]$_2$ (18 mg, 0.074 mmol) and HBpin (363 µL, 2.5 mmol) were reacted in ethyl propionate (5 mL). After 16 hours ethanolamine was added (0.3 mL, excess). Once effervescence had stopped, the mixture was filtered and concentrated *in vacuo*. The diastereoselectivity was determined by analysis of ^{1}H NMR of the crude reaction mixture using the peaks at 7.71 (*syn*) and 7.84 (*anti*) ppm (*syn*:*anti* 90:10). The crude product was purified by flash column chromatography (CombiFlash Isco NextGen300+, 12 g SiO_2, 50 mm Ø, petroleum ether/ethyl acetate 4:1) to give the *alcohol*, as a yellow oil (123 mg, 0.39 mmol, 78%, single diastereomer).

^{1}H NMR (500 MHz, $CDCl_3$) 7.70 (dd, *J* = 8.4, 1.4 Hz, 2H), 7.51-7.47 (m, 1H), 7.37-7.33 (m, 2H), 7.09-7.03 (m, 4H), 6.70 (d, *J* = 8.7 Hz, 2H), 3.86-3.78 (m, 2H), 3.13 (dd, *J* = 13.8, 9.6 Hz, 1H), 3.06 (dd, *J* = 13.8, 4.2, 1H), 2.61 (br. s, 1H), 2.39 (s, 3H), 1.65-1.52 (m, 2H), 0.99 (t, *J* = 7.4 Hz, 3H).

^{13}C NMR (126 MHz, $CDCl_3$) 204.6, 137.4, 136.8, 135.9, 133.3, 129.5, 128.6, 128.4, 127.1, 73.7, 52.9, 33.0, 27.9, 16.2, 10.5.

HRMS (EI^+) Calcd for $C_{19}H_{22}O_2S$ 314.1328; Found 314.1335.

IR ν_{max} (neat) 3420 (w, br), 2962 (w), 2922 (w), 2875 (w), 1670 (s).

(*2RS,3RS*)-2-(4-(Dimethylamino)benzyl)-3-hydroxy-1-phenyl-1-butanone, 3p

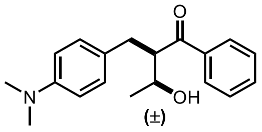

According to general procedure A, 4-(dimethylamino)chalcone (126 mg, 0.50 mmol), [H-*B*-9-BBN]$_2$ (18 mg, 0.074 mmol) and HBpin (363 μL, 2.5 mmol) were reacted in ethyl acetate (5 mL). After 16 hours ethanolamine was added (0.3 mL, excess). Once effervescence had stopped, the mixture was filtered and concentrated *in vacuo*. The diastereoselectivity was determined by analysis of ^{1}H NMR of the crude reaction mixture using the peaks at 7.75 (*syn*) and 7.88 (*anti*) ppm (*syn*:*anti* 89:11). The crude product was purified by flash column chromatography (CombiFlash Isco NextGen300+, 12 g SiO_2, 50 mm Ø, petroleum ether/ethyl acetate 4:1) to give the *alcohol*, as an orange oil (116 mg, 0.39 mmol, 78%, single diastereomer).

^{1}H NMR (500 MHz, $CDCl_3$) 7.74 (dd, *J* = 8.3, 1.3 Hz, 2H), 7.50-7.46 (m, 1H), 7.35 (dd, *J* = 8.3, 7.6 Hz, 2H), 7.01 (d, *J* = 8.7, 2H), 6.58 (d, *J* = 8.7, 2H), 4.13 (qd, *J* = 6.4, 4.5 Hz, 1H), 3.76-3.71 (m, 1H), 3.10-3.02 (m, 2H), 2.84 (s, 6H), 2.60 (br. s, 1H), 1.26 (d, *J* = 6.4 Hz, 3H).

^{13}C NMR (126 MHz, $CDCl_3$) 205.2, 149.3, 137.7, 133.0, 129.6, 128.5, 127.4, 113.1, 68.5, 54.8, 40.8, 33.0, 21.1.

2 x ^{13}C resonances were overlapping as a result 13 peaks are reported rather than the expected 14.

HRMS (EI^+) Calcd for $C_{19}H_{23}O_2N$ 297.1723; Found 297.1724.

IR ν_{max} (neat) 3380 (w, br), 2971 (w), 2932 (w), 2884 (w), 2800 (w), 1672 (s).

(*2RS,3RS*)-2-(4-*tert*-Butylbenzyl)-3-hydroxy-1-phenyl-1-butanone, 3q

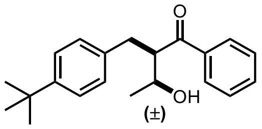

According to general procedure A, 4'-*tert*-butylchalcone (132 mg, 0.50 mmol), [H-*B*-9-BBN]$_2$ (18 mg, 0.074 mmol) and HBpin (363 µL, 2.5 mmol) were reacted in ethyl acetate (5 mL). After 16 hours ethanolamine was added (0.3 mL, excess). Once effervescence had stopped, the mixture was filtered and concentrated *in vacuo*. The diastereoselectivity was determined by analysis of ^{1}H NMR of the crude reaction mixture using the region from 7.60 to 8.00 ppm (single diastereomer). The crude product was purified by flash column chromatography (CombiFlash Isco NextGen300+, 12 g SiO_2, 50 mm Ø, petroleum ether/ethyl acetate 4:1) to give the *alcohol*, as a colourless oil (81 mg, 0.28 mmol, 52%, single diastereomer).

^{1}H NMR (500 MHz, $CDCl_3$) 7. 67 (dd, 2H), 7.48-7.44 (m, 1H), 7.34-7.28 (m, 2H), 7.17 (d, *J* = 8.3 Hz, 2H), 7.05 (d, *J* = 8.3, 2H), 4.16 (qd, *J* = 6.3, 4.4 Hz, 1H), 3.79-3.74 (m, 1H), 3.11 (m, 2H), 1.28 (d, *J* = 6.4 Hz, 3H), 1.23 (s, 9H).

^{13}C NMR (126 MHz, $CDCl_3$) 205.1, 137.7, 136.4, 133.0, 128.6, 128.4, 128.4, 125.3, 68.4, 54.6, 34.3, 33.5, 31.3, 21.1.

HRMS (EI^+) Calcd for $C_{21}H_{26}O_2$ 310.1927; Found 310.1937.

IR ν_{max} (neat) 3436 (w, br), 2962 (w), 2867 (w), 1672 (s).

(*2RS,3RS*)-2-(Benzofuran-2-ylmethyl)-3-hydroxy-1-phenyl-1-butanone, 3r

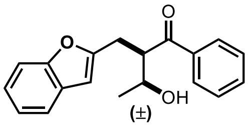

According to general procedure A, (*E*)-3-(benzofuran-2-yl)-1-phenylprop-2-en-1-one (124 mg, 0.50 mmol), [H-*B*-9-BBN]$_2$ (18 mg, 0.074 mmol) and HBpin (363 μL, 2.5 mmol) were reacted in ethyl acetate (5 mL). After 16 hours ethanolamine was added (0.3 mL, excess). Once effervescence had stopped, the mixture was filtered and concentrated *in vacuo*. The diastereoselectivity was determined by analysis of ^{1}H NMR of the crude reaction mixture using the peaks at 7.89 (*syn*) and 7.96 (*anti*) ppm (*syn*:*anti* 88:12). The crude product was purified by flash column chromatography (CombiFlash Isco NextGen300+, 12 g SiO_2, 50 mm Ø, petroleum ether/ethyl acetate 4:1) to give the *alcohol*, as a yellow oil (94 mg, 0.32 mmol, 64%, *syn*:*anti* 88:12).

Major diastereomer (*syn*):

^{1}H NMR (500 MHz, $CDCl_3$) 7.89 (dd, *J* = 8.4, 1.3 Hz, 2H), 7.51-7.47 (m, 1H), 7.39-7.35 (m, 3H), 7.34-7.31 (m, 1H), 7.18-7.10 (m, 2H), 6.34 (d, *J* = 0.9 Hz, 1H), 4.22 (qd, *J* = 6.4, 4.3 Hz, 1H), 4.06-4.00 (m, 1H), 3.33-3.30 (m, 2H), 2.80 (br. s, 1H), 1.29 (d, 6.4 Hz, 3H).

^{13}C NMR (126 MHz, $CDCl_3$) 203.8, 156.4, 154.7, 136.9, 133.5, 128.7, 128.6, 128.5, 123.4, 122.5, 120.4, 110.7, 103.7, 68.1, 50.9, 26.5, 21.1.

Minor diastereomer (*anti*):

^{1}H NMR (500 MHz, $CDCl_3$) 7.96 (dd, *J* = 8.4, 1.3 Hz, 2H), 7.56-7.52 (m, 1H), 7.45-7.40 (m, 3H), 7.22-7.15 (m, 3H), 6.42 (d, *J* = 0.9 Hz, 1H), 4.15-4.10 (m, 1H), 4.05-4.00 (obsc., 1H), 3.30-3.25 (m, 2H), 1.27 (d, *J* = 6.5 Hz, 3H).

^{13}C NMR (126 MHz, $CDCl_3$) 204.6, 155.7, 154.8, 137.2, 133.6, 128.8, 128.7 (obsc.), 128.4, 123.6, 122.6, 120.5, 110.7, 104.1, 68.7, 50.4, 28.7, 21.7.

HRMS (EI^+) Calcd for $C_{19}H_{18}O_3$ 294.1251; Found 294.1246.

IR ν_{max} (neat) 3443 (w, br), 3059 (w), 2972 (w), 2931 (w), 1672 (s).

(*2RS,3RS*)-2-(2-Methylfuryl)-3-hydroxy-1-phenyl-1-butanone, 3s

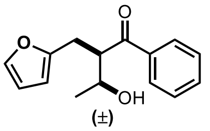

According to general procedure A, (*E*)-3-(furan-1-yl)-1-phenylprop-2-en-1-one (107 mg, 0.50 mmol), [H-*B*-9-BBN]$_2$ (18 mg, 0.074 mmol) and HBpin (363 μL, 2.5 mmol) were reacted in ethyl acetate (5 mL). After 16 hours ethanolamine was added (0.3 mL, excess). Once effervescence had stopped, the mixture was filtered and concentrated *in vacuo*. The diastereoselectivity was determined by analysis of ^{1}H NMR of the crude reaction mixture using the peaks at 7.85 (*syn*) and 7.91 (*anti*) ppm (*syn*:*anti* 19:1). The crude product was purified by flash column chromatography (CombiFlash Isco NextGen300+, 12 g SiO_2, 50 mm Ø, petroleum ether/ethyl acetate 4:1) to give the *alcohol*, as a colourless oil (90 mg, 0.28 mmol, 52%, *syn*:*anti* 92:8).

Mayor Disatereomer (*syn*):

^{1}H NMR (500 MHz, $CDCl_3$) 7.84 (dd, *J* = 8.4, 1.3 Hz, 2H), 7.56-7.52 (m, 1H), 7.44-7.40 (m, 2H), 7.21 (dd, *J* = 1.9, 0.8 Hz, 1H), 6.15 (dd, *J* = 3.2, 1.9 Hz, 1H), 5.93 (dd, *J* = 3.2, 0.8 Hz, 1H), 4.20-4.13 (m, 1H), 3.90-3.84 (m, 1H), 3.17-3.15 (m, 2H), 2.70 (s, 1H), 1.24 (d, *J* = 6.5 Hz, 3H).

^{13}C NMR (126 MHz, $CDCl_3$) 204.4, 153.1, 141.23, 137.0, 133.4, 128.6, 128.4, 110.3, 106.7, 68.0, 51.1, 26.0, 20.9.

Minor Disatereomer (*anti*):

^{1}H NMR (500 MHz, $CDCl_3$) 7.90 (dd, *J* = 8.4, 1.3 Hz, 2H), 7.58-7.56 (m, 1H), 7.47-7.43 (m, 2H), 7.25 (dd, *J* = 1.9, 0.8 Hz, 1H), 6.19 (dd, *J* = 3.2, 1.9 Hz, 1H), 6.00 (dd, *J* = 3.2, 0.8 Hz, 1H), 4.09-4.03 (m, 1H), 3.90-3.84 (obsc., 1H), 3.13-3.09 (m, 2H), 2.70 (br. s, 1H), 1.27-1.22 (obsc., 3H).

^{13}C NMR (126 MHz, $CDCl_3$) 205.1, 152.5, 141.4, 137.7, 133.5, 128.7, 128.3, 110.3 (obsc.), 107.0, 68.8, 50.9, 28.6, 21.8.

HRMS (EI$^+$) Calcd for $C_{15}H_{16}O_3$ 244.1094; Found 244.1097.

IR ν_{max} (neat) 3443 (w), 2972 (w), 2928 (w), 1672 (s).

(*2RS,3RS*)-2-Hydroxy-2-(naphthalen-2-yl)-1-phenyl-1-butanone, 3t

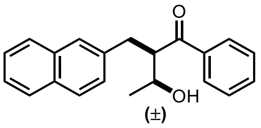

According to general procedure A, (2E)-1-phenyl-3-(2-naphthyl)-2-propen-1-one (129 mg, 0.50 mmol), [H-*B*-9-BBN]$_2$ (18 mg, 0.074 mmol) and HBpin (363 µL, 2.5 mmol) were reacted in ethyl acetate (5 mL). After 16 hours ethanolamine was added (0.3 mL, excess). Once effervescence had stopped, the mixture was filtered and concentrated *in vacuo*. The crude product was purified by flash column chromatography (CombiFlash Isco NextGen300+, 12 g SiO_2, 50 mm Ø, petroleum ether/ethyl acetate 4:1) to give the *alcohol*, as a colourless oil (116 mg, 0.38 mmol, 76%, single diastereomer).

^{1}H NMR (500 MHz, $CDCl_3$) 7.74-7.70 (m, 3H), 7.69-7.66 (m, 2H), 7.58 (s, 1H), 7.45-7.37 (m, 3H), 7.31-7.26 (m, 3H), 4.19 (qd, *J* = 6.4, 4.4 Hz, 1H), 3.90 (app. dt, *J* = 9.2, 4.8 Hz, 1H), 3.36-3.28 (m, 2H), 2.57 (br. s, 1H), 1.31 (d, *J* = 6.4 Hz, 3H).

^{13}C NMR (126 MHz, $CDCl_3$) 204.6, 137.5, 137.1 133.5, 133.2, 132.1, 128.5, 128.4, 128.1, 127.5, 127.5, 127.5, 127.4, 125.9, 125.3, 68.5, 54.4, 34.0, 21.2.

HRMS (EI^+) Calcd for $C_{21}H_{20}O_2$ 304.1458; Found 304.1464.

IR ν_{max} (neat) 3439 (w, br), 3055 (w), 2968 (w), 2928 (w), 1671.55 (s).

(*2RS,3RS*)-2-(4-Chlorobenzyl)-3-hydroxy-1-phenyl-1-butanone, 3u

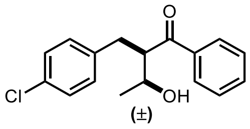

According to general procedure A, 4-chlorochalcone (121 mg, 0.50 mmol), [H-*B*-9-BBN]$_2$ (18 mg, 0.074 mmol) and HBpin (363 µL, 2.5 mmol) were reacted in ethyl acetate (5 mL). After 16 hours ethanolamine was added (0.3 mL, excess). Once effervescence had stopped, the mixture was filtered and concentrated *in vacuo*. The diastereoselectivity was determined by analysis of ^{1}H NMR of the crude reaction mixture using the peaks at 7.73 (*syn*) and 7.85 (*anti*) ppm (*syn*:*anti* 86:14). The crude product was purified by flash column chromatography (CombiFlash Isco NextGen300+, 12 g SiO_2, 50 mm Ø, petroleum ether/ethyl acetate 4:1) to give the *alcohol*, as a colourless oil (114 mg, 0.40 mmol, 79%, *syn:anti* 86:14).

Major diastereomer (*syn*):

^{1}H NMR (500 MHz, $CDCl_3$) 7.70 (dd, *J* = 8.4, 1.3 Hz, 2H), 7.53-7.48 (m, 1H), 7.39-7.34 (m, 2H), 7.14-7.10 (m, 2H), 7.08-7.04 (m, 2H), 4.13 (qd, *J* = 6.3, 4.7 Hz, 1H), 3.74 (app. dt, *J* = 9.4, 4.7 Hz, 1H), 3.16-3.06 (m, 2H), 1.27 (d, *J* = 6.3 Hz, 3H).

^{13}C NMR (126 MHz, $CDCl_3$) 204.3, 138.0, 137.4, 133.4, 132.0, 130.4, 128.6, 128.5, 128.3, 68.3, 54.4, 33.2, 21.2.

Minor diastereomer (*anti*):

^{1}H NMR (500 MHz, $CDCl_3$) 7.83 (dd, *J* = 8.4, 1.3 Hz, 2H), 7.58-7.54 (m, 1H), 7.45-7.41 (m, 2H), 7.21-7.17 (m, 2H), 7.15-7.11 (obsc., 2H), 4.02-3.96 (m, 1H), 3.72-3.68 (m, 1H), 3.09-3.01 (m, 2H), 1.23 (d, *J* = 6.3 Hz, 3H).

^{13}C NMR (126 MHz, $CDCl_3$) 205.3, 137.5, 137.4, 133.6, 132.3, 130.5, 128.8, 128.6, 128.2, 68.3, 53.6, 35.3, 22.0.

HRMS (EI^+) Calcd for $C_{17}H_{17}O_2Cl$ 288.0912; Found 288.0901.

IR ν_{max} (neat) 3440 (w, br), 2971 (w), 2932 (w), 1672 (s).

(*2RS,3RS*)-2-(4-(Benzyloxy)benzyl)-3-hydroxy-1-phenyl-1-butanone, 3v

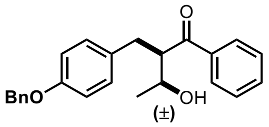

According to general procedure A, 4-(benzyloxy)chalcone (158 mg, 0.50 mmol), [H-*B*-9-BBN]$_2$ (18 mg, 0.074 mmol) and HBpin (363 µL, 2.5 mmol) were reacted in ethyl acetate (5 mL). After 16 hours ethanolamine was added (0.3 mL, excess). Once effervescence had stopped, the mixture was filtered and concentrated *in vacuo*. The diastereoselectivity was determined by analysis of ^{1}H NMR of the crude reaction mixture using the peaks at 7.69 (*syn*) and 7.82 (*anti*) ppm (*syn*:*anti* 90:10). The crude product was purified by flash column chromatography (CombiFlash Isco NextGen300+, 12 g SiO_2, 50 mm Ø, petroleum ether/ethyl acetate 4:1) to give the *alcohol*, as a pale yellow needles (148 mg, 0.41 mmol, 82%, *syn:anti* 92:8).

Major diastereomer (*syn*):

^{1}H NMR (500 MHz, $CDCl_3$) 7.70 (dd, *J* = 8.4, 1.3 Hz, 2H), 7.50-7.46 (m, 1H), 7.38-7.29 (m, 7H), 7.04 (d, *J* = 8.6 Hz, 2H), 6.78 (app. d, *J* = 8.6 Hz, 2H), 4.96 (s, 2H), 4.18-4.10 (m, 1H), 3.76-3.73 (m, 1H), 3.10 (app. d, *J* = 7.2 Hz, 2H), 2.67 (d, *J* = 3.3 Hz, 1H), 1.27 (d, *J* = 6.4 Hz, 3H).

^{13}C NMR (126 MHz, $CDCl_3$) 205.0, 157.2, 137.6, 137.1, 133.1, 131.8, 130.0, 128.5, 128.5, 128.4, 127.9, 127.4, 114.9, 70.0, 68.4, 54.7, 33.0, 22.1.

Minor diastereomer (*anti*):

^{1}H NMR (500 MHz, $CDCl_3$) 7.85 (dd, *J* = 8.4, 1.3 Hz, 2H), 7.57-7.53 (m, 1H), 7.45-7.28 (m, 7H), 7.14 (d, *J* = 8.6 Hz, 2H), 6.86 (d, *J* = 8.6 Hz, 2H), 5.00 (s, 2H), 4.05-4.00 (m, 1H), 3.73-3.69 (m, 1H), 3.03 (app. d, *J* = 7.3 Hz, 2H), 1.24 (d, *J* = 6.4, 3H).

^{13}C NMR (126 MHz, $CDCl_3$) 206.0, 157.4, 137.7 (obsc.), 137.1, 133.4, 131.3, 130.2, 128.7, 128.6, 128.3, 128.0, 127.5, 115.0, 70.0, 68.3, 54.0, 35.3, 21.1.

HRMS (EI$^+$) Calcd for $C_{24}H_{24}O_3$ 360.1720; Found 360.1719.

IR ν_{max} (neat) 3521 (w, br), 2981 (w), 2962 (w), 2928 (w), 2891 (w), 1660 (s).

m.p. 77-79 °C (CH_2Cl_2)

(*2RS,3RS*)-2-(4-(2-Butynyloxy)benzyl)-3-hydroxy-1-phenyl-1-butanone, 3w

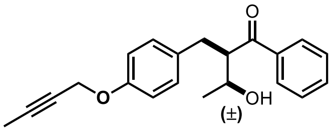

According to general procedure A, (*E*)-3-(4-(2-butynyloxy)phenyl)-1-phenylprop-2-en-1-one (138 mg, 0.50 mmol), [H-*B*-9-BBN]$_2$ (18 mg, 0.074 mmol) and HBpin (363 μL, 2.5 mmol) were reacted in ethyl acetate (5 mL). After 16 hours ethanolamine was added (0.3 mL, excess). Once effervescence had stopped, the mixture was filtered and concentrated *in vacuo*. The diastereoselectivity was determined by analysis of ^{1}H NMR of the crude reaction mixture using the peaks at 7.65 (*syn*) and 7.78 (*anti*) ppm (*syn*:*anti* 19:1). The crude product was purified by flash column chromatography (CombiFlash Isco NextGen300+, 12 g SiO_2, 50 mm Ø, petroleum ether/ethyl acetate 4:1) to give the *alcohol*, as a colourless oil (90 mg, 0.28 mmol, 52%, *syn*:*anti* 19:1).

^{1}H NMR (500 MHz, $CDCl_3$) 7.76-7.65 (m, 2H), 7.50-7.45 (m, 1H), 7.38-7.31 (m, 2H), 7.07-7.02 (m, 2H), 6.79-6.73 (m, 2H), 4.56-4.52 (m, 2H), 4.17-4.09 (m, 1H), 3.76-3.70 (m, 1H), 3.08 (app. d, J = 7.6 Hz, 2H), 2.56 (br. s, 1H), 1.85-1.81 (m, 3H), 1.26 (d, J = 6.4 Hz, 3H).

^{13}C NMR (126 MHz, $CDCl_3$) 205.0, 156.3, 137.6, 133.1, 132.1, 129.9, 128.5, 128.4, 114.9, 83.6, 74.1, 68.4, 56.5, 54.7, 33.1, 21.1, 3.7.

HRMS (EI^+) Calcd for $C_{21}H_{22}O_3$ 322.1564; Found 322.1566.

IR ν_{max} (neat) 3457 (w, br), 2969 (w), 2921 (w), 1672 (s).

3β-acetoxy-17-α-(ethan-1-ol)-5-pregnen-2-one, 3x

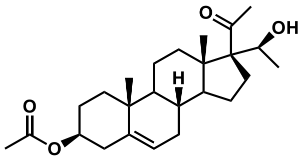

According to general procedure A, 16-dihydropregnenolone acetate (179 mg, 0.50 mmol), [H-*B*-9-BBN]$_2$ (18 mg, 0.074 mmol) and HBpin (363 µL, 2.5 mmol) were reacted in ethyl acetate (5 mL). After 16 hours ethanolamine was added (0.3 mL, excess). Once effervescence had stopped, the mixture was filtered and concentrated *in vacuo*. The crude product was purified by flash column chromatography (CombiFlash Isco NextGen300+, 12 g SiO_2, 50 mm Ø, petroleum ether/ethyl acetate 4:1) to give the *alcohol*, as a colourless microcrystalline solid (78 mg, 0.19 mmol, 39 %, single diastereomer).

^{1}H NMR (600 MHz, $CDCl_3$) 5.38-5.36 (m, 1H), 4.61 (tdd, *J* = 11.4, 5.6, 4.2 Hz, 1H), 4.19 (p, *J* = 6.4 Hz, 1H), 2.50 (ddd, *J* = 14.4, 11.2, 2.7 Hz, 1H), 2.41-2.29 (m, 2H), 2.27 (s, 3H), 2.05 (s, 3H), 2.02-1.94 (m, 2H), 1.92-1.82 (m, 3H), 1.76 -1.67 (m, 2H), 1.65-1.42 (m, 8H), 1.26 (d, *J*= 6.3 Hz, 3H), 1.15 (td, *J* = 13.9, 13.5, 4.2 Hz, 1H), 1.04 (s, 3H), 1.02-0.95 (m, 1H), 0.71 (m, 3H).

^{13}C NMR (151 MHz, $CDCl_3$) 214.4, 170.9, 140.1, 122.6, 74.2, 70.3, 70.0, 52.1, 49.9, 46.8, 38.4, 37.3, 37.0, 34.2, 32.5, 32.4, 31.7, 28.1, 26.3, 25.2, 22.3, 21.8, 21.1, 19.6, 16.8.

HRMS (EI^+) Calcd for $C_{25}H_{39}O_4$ 403.2843; Found 403.2849.

IR ν_{max} (neat) 3386 (br), 2931 (m), 1732 (s), 1440 (m), 1371(m), 1247 (s).

$[\alpha]_D^{20}$ −160 (c 0.0135, CH_2Cl_2).

m.p. 122-123 (EtOAc)

3β-Acetoxy-17 α-(propan-1-ol)-5-pregnen-2-one, 3y

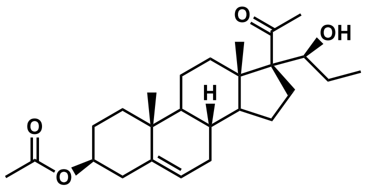

Molecular Weight: 416.60

According to general procedure A, 16-dihydropregnenolone acetate (179 mg, 0.50 mmol), [H-*B*-9-BBN]$_2$ (18 mg, 0.074 mmol) and HBpin (363 μL, 2.5 mmol) were reacted in ethy propionate (5 mL). After 16 hours ethanolamine was added (0.3 mL, excess). Once effervescence had stopped, the mixture was filtered and concentrated *in vacuo*. The crude product was purified by flash column chromatography (CombiFlash Isco NextGen300+, 12 g SiO_2, 50 mm Ø, petroleum ether/ethyl acetate 4:1) to give the *alcohol*, as a white microcrystalline solid (106 mg, 0.26 mmol, 51 %, single diastereomer).

^{1}H NMR (500 MHz, $CDCl_3$) 5.44 – 5.34 (m, 1H), 4.62 (tdd, *J* = 11.4, 5.7, 4.2 Hz, 1H), 3.72 (ddd, *J* = 10.2, 8.1, 1.8 Hz, 1H), 2.55 (d, *J* = 8.1 Hz, 1H), 2.48 (ddd, *J* = 14.7, 11.0, 2.6 Hz, 1H), 2.39 – 2.29 (m, 2H), 2.27 (s, 3H), 2.05 (s, 3H), 2.02 – 1.96 (m, 2H), 1.91 – 1.85 (m, 2H), 1.74 (dddd, *J* = 20.2, 12.1, 7.0, 2.3 Hz, 2H), 1.67 – 1.56 (m, 6H), 1.55 – 1.42 (m, 3H), 1.35 – 1.25 (m, 2H), 1.16 – 1.07 (m, 2H), 1.07 – 1.02 (m, 6H), 1.04 - 0.97 (m, 1H) 0.73 (s, 3H).

^{13}C NMR (126 MHz, $CDCl_3$) 215.6, 170.9, 140.2, 122.6, 76.6, 74.3, 69.5, 52.2, 49.9, 47.6, 38.5, 37.4, 37.0, 34.7, 32.6, 32.5, 32.3, 28.1, 28.1, 27.7, 25.4, 21.8, 21.2, 19.7, 16.9, 11.7

HRMS (EI^+) Calcd for $C_{26}H_{41}O_4$ 417.2999; Found 417.3005.

IR ν_{max} (neat) 3559 (w), 2930 (w), 1728 (s), 1695 (m), 1453 (m), 1240 (m).

$[\alpha]_D^{20}$ −171 (c 0.0215, CH_2Cl_2).

m.p. 141-142 (EtOAc)

(*2RS,3RS*)-3-Hydroxy-2-methyl-1,2-diphenyl-1-butanone, 3z

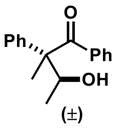

According to general procedure A, 1,2-diphenylprop-2-en-1-one (104 mg, 0.50 mmol), [H-*B*-9-BBN]$_2$ (18 mg, 0.074 mmol) and HBpin (363 µL, 2.5 mmol) were reacted in ethyl acetate (5 mL). After 16 hours ethanolamine was added (0.3 mL, excess). Once effervescence had stopped, the mixture was filtered and concentrated *in vacuo*. The crude product was purified by flash column chromatography (CombiFlash Isco NextGen300+, 12 g SiO_2, 50 mm Ø, petroleum ether/ethyl acetate 4:1) to give the *alcohol*, as a colourless oil (95 mg, 0.38 mmol, 75%, isolated as single diastereomer).

^{1}H NMR (500 MHz, $CDCl_3$) 7.47-7.44 (m, 2H), 7.40-7.34 (m, 3H), 7.32-7.28 (m, 3H), 7.24-7.19 (m, 2H), 4.58 (q, *J* = 6.5 Hz), 3.59 (br. s, 1H), 1.71 (s, 3H), 0.83 (d, *J* = 6.5 Hz).

^{13}C NMR (126 MHz, $CDCl_3$) 206.0, 140.5, 136.2, 132.2, 129.9, 129.2, 128.0, 127.5, 126.7, 72.8, 59.5, 15.8, 14.7.

HRMS (EI^+) Calcd for $C_{17}H_{18}O_2$ 254.1301; Found 254.1304.

IR ν_{max} (neat) 3569 (w), 3059 (w), 2981 (w), 2916 (w), 1662 (s), 1445 (s).

(*2RS,3RS*)-3-Cyclobutyl-3-hydroxy-2-methyl-1,2-diphenyl-1-propanone, 3aa

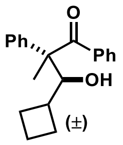

According to general procedure A, 1,2-diphenylprop-2-en-1-one (104 mg, 0.50 mmol), [H-*B*-9-BBN]$_2$ (18 mg, 0.074 mmol) and HBpin (363 µL, 2.5 mmol) were reacted in ethyl cyclobutylcarboxylate (5 mL). After 16 hours ethanolamine was added (0.3 mL, excess). Once effervescence had stopped, the mixture was filtered and concentrated *in vacuo*. The crude product was purified by flash column chromatography (CombiFlash Isco NextGen300+, 12 g SiO_2, 50 mm Ø, petroleum ether/ethyl acetate 4:1) to give the *alcohol*, as a colourless oil (66 mg, 0.23 mmol, 45%, isolated as single diastereomer).

^{1}H NMR (500 MHz, $CDCl_3$) 7.42-7.38 (m, 2H), 7.37-7.27 (m, 6H), 7.23-7.17 (m, 2H), 4.30 (d, *J* = 6.5 Hz, 1 H), 3.37 (br. s, 1H), 2.29-2.19 (m, 1H), 2.11-2.01 (m, 1H), 1.95-1.87 (m, 1H), 1.67 (s, 3H), 1.67-1.60 (m, 1H), 1.40-1.30 (m, 1H), 1.14-1.06 (m, 1H).

^{13}C NMR (126 MHz, $CDCl_3$) 205.9, 140.1, 136.3, 132.0, 129.7, 129.0, 128.0, 127.5, 127.0, 59.6, 36.0, 26.4, 25.2, 18.7, 15.7.

HRMS (EI^+) Calcd for $C_{20}H_{23}O_2$ 295.1693; Found 295.1693.

IR ν_{max} (neat) 3553 (w), 2976 (w), 2933 (w), 2859 (w), 1661 (s), 1445 (s).

(*2RS,3RS*)-3-Hydroxy-2-methyl-1,2-diphenyl-1-pentanone, 3ab

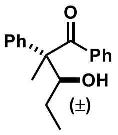

According to general procedure A, 1,2-diphenylprop-2-en-1-one (104 mg, 0.50 mmol), [H-*B*-9-BBN]$_2$ (18 mg, 0.074 mmol) and HBpin (363 µL, 2.5 mmol) were reacted in ethyl propionate (5 mL). After 16 hours ethanolamine was added (0.3 mL, excess). Once effervescence had stopped, the mixture was filtered and concentrated *in vacuo*. The crude product was purified by flash column chromatography (CombiFlash Isco NextGen300+, 12 g SiO_2, 50 mm Ø, petroleum ether/ethyl acetate 4:1) to give the *alcohol*, as a colourless oil (66 mg, 0.23 mmol, 45%, isolated as single diastereomer).

^{1}H NMR (500 MHz, $CDCl_3$) 7.42 (dd, *J* = 8.5, 1.3 Hz, 2H), 7.40-7.34 (m, 3H), 7.33-7.28 (m, 3H), 7.24-7.19 (m, 2H), 4.27-4.21 (m, 1H), 3.41 (br. s, 1H), 1.69 (s, 3H), 1.30-1.20 (m, 1H), 0.99-0.90 (m, 1H), 0.87 (t, *J* = 7.0 Hz).

^{13}C NMR (126 MHz, $CDCl_3$) 206.0, 140.3, 136.4, 132.0, 129.7, 129.2, 128.0, 127.5, 126.9, 78.6, 59.7, 22.5, 15.4, 11.5.

HRMS (EI$^+$) Calcd for $C_{18}H_{20}O_2$ 268.1458; Found 268.1461.

IR ν_{max} (neat) 3553 (w), 2978 (w), 2961 (w), 2931 (w), 2875 (w), 1659 (s), 1445 (m).

(*2RS,3RS*)-3-Hydroxy-2,5-dimethyl-1,2-diphenyl-1-hexanone, 3ac

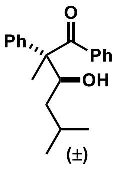

According to general procedure A, 1,2-diphenylprop-2-en-1-one (104 mg, 0.50 mmol), [H-*B*-9-BBN]$_2$ (18 mg, 0.074 mmol) and HBpin (363 µL, 2.5 mmol) were reacted in ethyl isovalerate (5 mL). After 16 hours ethanolamine was added (0.3 mL, excess). Once effervescence had stopped, the mixture was filtered and concentrated *in vacuo*. The crude product was purified by flash column chromatography (CombiFlash Isco NextGen300+, 12 g SiO_2, 50 mm Ø, petroleum ether/ethyl acetate 4:1) to give the *alcohol*, as a colourless oil (60 mg, 0.20 mmol, 40%, isolated as single diastereomer).

^{1}H NMR (500 MHz, $CDCl_3$) 7.42 (dd, *J* = 8.5, 1.3 Hz, 2H), 7.40-7.34 (m, 3H), 7.33-7.29 (m, 3H), 7.24-7.18 (m, 2H), 4.43 (d, *J* = 10.3 Hz, 1H), 3.39 (br. s, 1H), 1.78-1.69 (m, 1H), 1.69 (s, 3H), 1.29-1.23 (m, 1H), 0.95-0.85 (m, 1H), 0.80 (d, *J* = 6.8 Hz, 3H), 0.63 (d, *J* = 6.6 Hz, 3H).

^{13}C NMR (126 MHz, $CDCl_3$) 206.2, 140.1, 136.5, 132.0, 129.7, 129.1, 128.0, 127.4, 126.9, 74.8, 59.6, 38.5, 24.7, 23.9, 21.2, 15.2.

HRMS (EI^+) Calcd for $C_{20}H_{25}O_2$ 297.1849; Found 297.1839.

IR ν_{max} (neat) 3555 (w), 2953 (m), 2918 (m), 2868 (w), 2849 (w), 1661 (s), 1597 (m), 1576 (m), 1445 (m).

(*2RS,3RS*)-2-Benzyl-3,6-dihydroxy-1-phenyl-1-hexanone, 4a

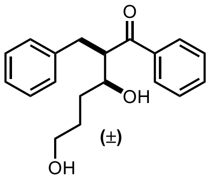

According to general procedure B, chalcone (100 mg, 0.50 mmol), [H-*B*-9-BBN]$_2$ (18 mg, 0.074 mmol), HBpin (363 µL, 2.5 mmol) and γ-butyrolactone (190 µL, 2.5 mmol) were reacted in THF (3 mL). After 16 hours ethanolamine was added (0.3 mL, excess). Once effervescence had stopped, the mixture was filtered and concentrated *in vacuo*. The diastereoselectivity was determined by analysis of ^{1}H NMR of the crude reaction mixture using the peaks at 7.67 (*syn*) and 7.81 (*anti*) ppm (*syn*:*anti* 90:10). The crude product was purified by flash column chromatography (CombiFlash Isco NextGen300+, 12 g SiO_2, 50 mm Ø, petroleum ether/ethyl acetate 4:1) to give the *alcohol*, as a colourless oil (115 mg, 0.39 mmol, 77%, *syn:anti* 88:12).

Major diastereomer (*syn*):

^{1}H NMR (500 MHz, $CDCl_3$) 7.67 (dd, *J* = 8.4, 1.3 Hz, 2H), 7.49-7.44 (m, 1H), 7.35-7.30 (m, 2H), 7.18-7.10 (m, 4H), 7.09-7.04 (m, 1H), 3.99-3.94 (m, 1H), 3.85-3.80 (m, 1H), 3.72-3.61 (m, 2H), 3.18-3.12 (m, 2H), 1.73-1.63 (m, 4H).

^{13}C NMR (126 MHz, $CDCl_3$) 205.1, 139.5, 137.5, 133.2, 129.0, 128.5, 128.4, 128.4, 126.2, 72.3, 62.7, 53.7, 34.2, 32.3, 29.6.

Minor diastereomer (*anti*):

^{1}H NMR (500 MHz, $CDCl_3$) 7.85-7.82 (m, 2H), 7.57-7.51 (m, 1H), 7.44-7.39 (m, 2H), 7.25-7.19 (m, 4H), 7.17-7.10 (obsc., 1H), 4.00-3.92 (obsc., 1H), 3.80-3.77 (m, 1H), 3.69-3.58 (obsc., 2H), 3.12-3.06 (m, 2H), 1.75-1.63 (obsc., 4H).

^{13}C NMR (126 MHz, $CDCl_3$) 206.2, 138.8, 137.4, 133.6, 129.1, 128.7, 128.6, 128.3, 126.5, 72.6, 62.8 (obsc.), 52.5, 36.3, 33.2, 29.9.

HRMS (EI^+) Calcd for $C_{19}H_{22}O_3$ 298.1564; Found 298.1554.

IR ν_{max} (neat) 3336 (br), 2935 (m), 1871 (m), 1671 (s), 1448 (s).

(*2RS,3RS*)-2-Benzyl-3,8-dihydroxy-1-phenyl-1-octanone, 4b

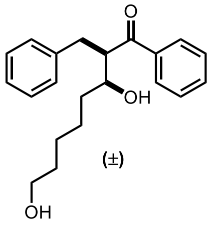

According to general procedure B, chalcone (100 mg, 0.50 mmol), [H-*B*-9-BBN]$_2$ (18 mg, 0.074 mmol), HBpin (363 µL, 2.5 mmol) and ε-caprolactone (285 µL, 2.5 mmol) were reacted in THF (3 mL). After 16 hours ethanolamine was added (0.3 mL, excess). Once effervescence had stopped, the mixture was filtered and concentrated *in vacuo*. The diastereoselectivity was determined by analysis of ^{1}H NMR of the crude reaction mixture using the peaks at 7.68 (*syn*) and 7.84 (*anti*) ppm (*syn*:*anti* 86:14).The crude product was purified by flash column chromatography (CombiFlash Isco NextGen300+, 12 g SiO_2, 50 mm Ø, petroleum ether/ethyl acetate 4:1) to give the *alcohol*, as a colourless oil (114 mg, 0.35 mmol, 70%, *syn:anti* 85:15).

Major diastereomer (*syn*):

^{1}H NMR (600 MHz, $CDCl_3$) 7.69-7.64 (m, 2H), 7.48-7.44 (m, 1H), 7.32 (dd, *J* = 8.4, 7.3 Hz, 2H), 7.17-7.10 (m, 4H), 7.09-7.04 (m, 1H), 3.96-3.90 (m, 1H), 3.81-3.77 (m, 1H), 3.61 (t, *J* = 6.5 Hz, 2H), 3.15-3.10 (m, 2H), 1.62-1.46 (m, 5H), 1.43-1.32 (m, 3H).

^{13}C NMR (151 MHz, $CDCl_3$) 205.0, 139.6, 137.5, 133.2, 129.0, 128.5, 128.4, 128.3, 126.2, 72.1, 62.7, 53.6, 34.9, 33.9, 32.5, 25.8, 25.6.

Minor diastereomer (*anti*):

^{1}H NMR (600 MHz, $CDCl_3$) 7.83-7.79 (m, 2H), 7.55-7.51 (m, 1H), 7.42-7.39 (m, 2H), 7.24-7.19 (m, 4H), 7.16-7.10 (obsc., 1H), 3.92-3.90 (obsc., 1H), 3.77-3.71 (m, 1H), 3.56 (t, *J* = 6.6 Hz, 2H), 3.10-3.06 (obsc., 2H), 1.62-1.29 (obsc., 8H).

^{13}C NMR (151 MHz, $CDCl_3$) 206.3, 138.9, 137.5, 133.5, 129.1, 128.7, 128.5, 128.3, 126.5, 72.4, 62.0, 52.3, 36.3, 36.0, 29.7, 25.9, 25.5.

HRMS (EI$^+$) Calcd for $C_{21}H_{27}O_3$ 327.1955; Found 327.1937.

IR ν_{max} (neat) 3336 (w, br), 2935 (w), 2871 (w), 1672 (s).

(*2RS,3RS,6R*)-2-Benzyl-3,6-dihydroxy-1-phenyl-1-undecanone and (*2RS,3RS,6S*)-2-benzyl-3,6-dihydroxy-1-phenyl-1-undecanone 4c

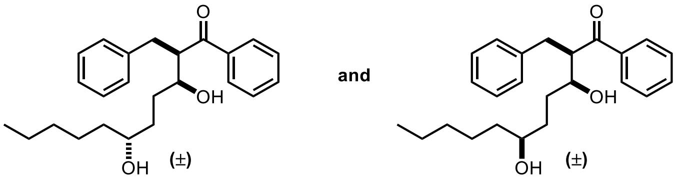

According to general procedure B, chalcone (100 mg, 0.50 mmol), [H-*B*-9-BBN]$_2$ (18 mg, 0.074 mmol), HBpin (363 µL, 2.5 mmol) and (±)coconut lactone (400 µL, 2.5 mmol) were reacted in THF (3 mL). After 16 hours ethanolamine was added (0.3 mL, excess). Once effervescence had stopped, the mixture was filtered and concentrated *in vacuo*. The crude product was purified by flash column chromatography (CombiFlash Isco NextGen300+, 12 g SiO_2, 50 mm Ø, petroleum ether/ethyl acetate 4:1) to give the *alcohol*, as a colourless oil (127 mg, 0.35 mmol, 69%, 50:50 mixture of diastereomers 1 and 2). ^{13}C NMR was characterised according to relative intensity of peaks.

^{1}H NMR (500 MHz, $CDCl_3$) 7.71-7.63 (m, 2H), 7.49-7.43 (m, 1H), 7.35-7.28 (m, 2H), 7.18-7.09 (m, 4H), 7.09-7.04 (m, 1H), 4.00-3.92 (m, 1H), 3.86-3.77 (m, 1H), 3.66-3.50 (m, 1H), 3.21-3.10 (m, 2H), 1.70-1.62 (m, 3H), 1.55-1.46 (m, 1H), 1.46-1.35 (m, 3H), 1.33-1.22 (m, 6H), 0.88 (t, J = 6.8 Hz, 3H).

Diastereomer 1:

^{13}C NMR (126 MHz, $CDCl_3$) 205.0, 139.6, 137.5, 133.2, 129.0, 128.5, 128.4, 128.4, 126.2, 72.4, 71.6, 53.8, 37.4, 34.4, 33.7, 31.9, 31.1, 25.4, 22.6, 14.0.

Diastereomer 2:

^{13}C NMR (126 MHz, $CDCl_3$) 204.9, 139.6, 137.6, 133.1, 129.0, 128.5, 128.4, 128.4, 126.2, 72.8, 72.1, 54.0, 37.8, 34.3, 33.7, 32.1, 31.9, 25.4, 22.6, 14.0.

HRMS (EI^+) Calcd for $C_{24}H_{32}O_3$ 368.2346; Found 368.2353.

IR ν_{max} (neat) 3386 (w, br), 2928 (m), 2858 (m), 1672 (s).

(*2RS,3RS*)-5-Ethyl-1,4-dihydroxy-6-octanone, 4d

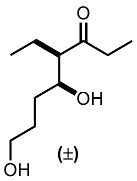

According to general procedure B, (*E*)-4-hexen-3-one (57 μL, 0.50 mmol), [H-*B*-9-BBN]$_2$ (18 mg, 0.074 mmol), HBpin (363 μL, 2.5 mmol) and γ-butyrolactone (190 μL, 2.5 mmol) were reacted in THF (3 mL). After 16 hours ethanolamine was added (0.3 mL, excess). Once effervescence had stopped, the mixture was filtered and concentrated *in vacuo*. The crude product was purified by flash column chromatography (CombiFlash Isco NextGen300+, 12 g SiO_2, 50 mm Ø, petroleum ether/ethyl acetate 4:1) to give the *alcohol*, as a colourless oil (50 mg, 0.26 mmol, 52%, single diastereomer).

^{1}H NMR (500 MHz, $CDCl_3$) 3.78 (ddd, J = 8.6, 4.8, 3.7, 3H), 3.71-3.65 (m, 1H), 3.65-3.59 (m, 1H), 2.60-2.43 (m, 3H), 1.76-1.62 (m, 4H), 1.55-1.46 (m, 2H), 1.04 (t, J = 7.2 Hz, 3H), 0.88 (t, J = 7.5 Hz, 3H).

^{13}C NMR (126 MHz, $CDCl_3$) 216.3, 71.7, 62.7, 58.3, 37.9, 31.9, 29.6, 20.2, 12.3, 7.4.

HRMS (EI$^+$) Calcd for $C_{10}H_{20}O_3$ 188.1407; Found 188.1410.

IR ν_{max} (neat) 3365 (w, br), 2962 (w), 2937 (w), 2877 (w), 1699 (s).

(*2RS,3RS*)-5-Ethyl-1,4-dihydroxy-6-decanone, 4e

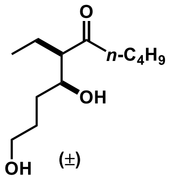

According to general procedure B, (*E*)-2-octen-4-one (63 µL, 0.50 mmol), [H-*B*-9-BBN]$_2$ (18 mg, 0.074 mmol), HBpin (363 µL, 2.5 mmol) and γ-butyrolactone (190 µL, 2.5 mmol) were reacted in THF (3 mL). After 16 hours ethanolamine was added (0.3 mL, excess). Once effervescence had stopped, the mixture was filtered and concentrated *in vacuo*. The crude product was purified by flash column chromatography (CombiFlash Isco NextGen300+, 12 g SiO_2, 50 mm Ø, petroleum ether/ethyl acetate 4:1) to give the *alcohol*, as a colourless oil (61 mg, 0.28 mmol, 56%, single diastereomer).

^{1}H NMR (600 MHz, $CDCl_3$) 3.81 (dt, J = 8.5, 4.2 Hz, 1H), 3.71 (dt, J = 11.1, 5.6 Hz, 1H), 3.65 (dt, J = 10.6, 6.2 Hz, 1H), 3.36 (s, 1H), 2.60 – 2.49 (m, 2H), 2.47 (dt, J = 17.5, 7.4 Hz, 1H), 1.81 – 1.62 (m, 4H), 1.60 – 1.47 (m, 4H), 1.39 – 1.30 (m, 2H), 1.32 – 1.22 (m, 1H), 0.92 (m, 6H).

^{13}C NMR (126 MHz, $CDCl_3$) 216.3, 72.0, 63.1, 58.7, 44.7, 32.3, 30.0, 25.7, 22.6, 20.4, 14.2, 12.7.

HRMS (EI^+) Calcd for $C_{12}H_{24}O_3Na$ 239.1618; Found 239.1618.

IR ν_{max} (neat) 2940 (w), 1745 (m), 1373 (m).

S7 Gram-scale Aldol-type Reactions

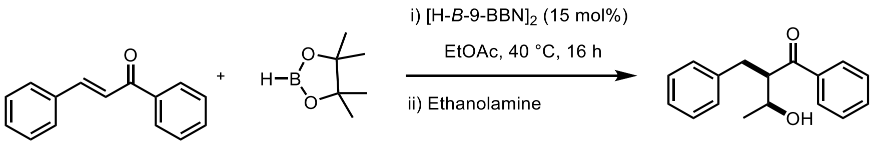

Chalcone (1.0 g, 5.0 mmol, 1.00 eq.), 9-borabicyclo(3.3.1)nonane [H-*B*-9-BBN]$_2$ (180.0 mg, 15 mol%) and 4,4,5,5-tetramethyl-1,3,2-dioxaborolane (HBpin) (3.6 mL, 5 eq.) were stirred in solution of ester (0.1 M) at 40 °C for 16 hours under a nitrogen atmosphere. The reactions was quenched with ethanolamine (0.5 mL, 82.0 mmol) and the diastereoselectivity were determined by analysis of the crude reaction mixture by ^{1}H NMR spectroscopy. The crude product was purified by flash column chromatography (CombiFlash Isco NextGen300+, 12 g SiO$_2$, 50 mm Ø, petroleum ether/ethyl acetate 4:1) to give the alcohol, as a colourless oil (0.99 g, 4.1 mmol, 81%, *syn*:*anti* 90:10).

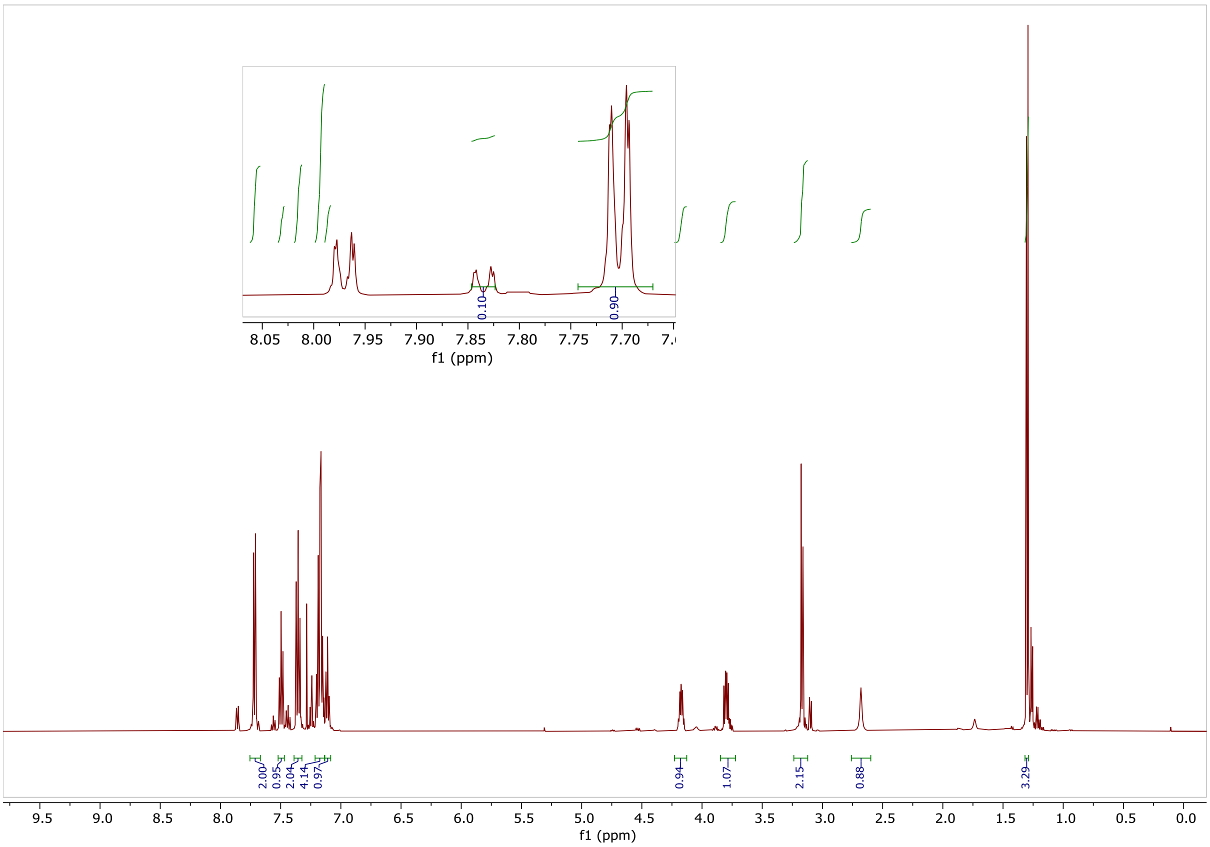

^{1}H NMR (500 MHz, CDCl$_3$) Spectrum of (*2RS,3RS*)-2-benzyl-3-hydroxy-1-phenyl-1-butanone **3a**. Expansion: diastereoselectivity determined by ^{1}H NMR analysis of the crude reaction mixture.

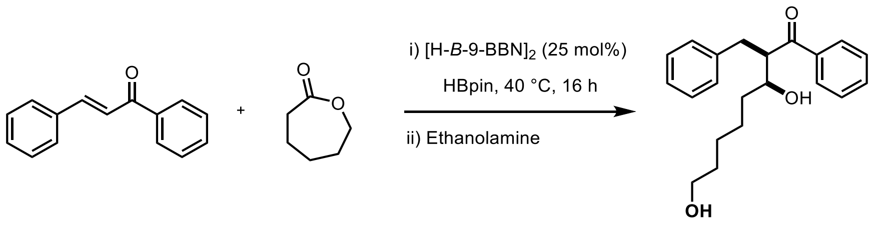

Chalcone (1.0 g, 5.0 mmol, 1.00 eq.), 9-borabicyclo(3.3.1)nonane [H-*B*-9-BBN]$_2$ (300.0 mg, 25 mol%), 4,4,5,5-tetramethyl-1,3,2-dioxaborolane (HBpin) (3.6 mL, 5 eq.) and ε-caprolactone (2.8 mL, 5 eq.) were reacted in THF (0.17 M) at 40 °C for 16 hours. The reaction was quenched with ethanolamine (0.5 mL, 82 mmol) and the diastereoselectivity were determined by analysis of the crude reaction mixture by ^{1}H NMR spectroscopy. The crude product was purified by flash column chromatography (CombiFlash Isco NextGen300+, 12 g SiO_2, 50 mm Ø, petroleum ether/ethyl acetate 4:1) to give the *alcohol* as an oil (66%, *syn*:*anti* 86:14 *d.r.*) which recrystallised on standing to give the syn diastereomer of the *alcohol*, as a colourless microcrystalline solid (0.373 g, 1.1 mmol, 23%, single diastereomer).

m.p. 85-86 °C (Et_2O)

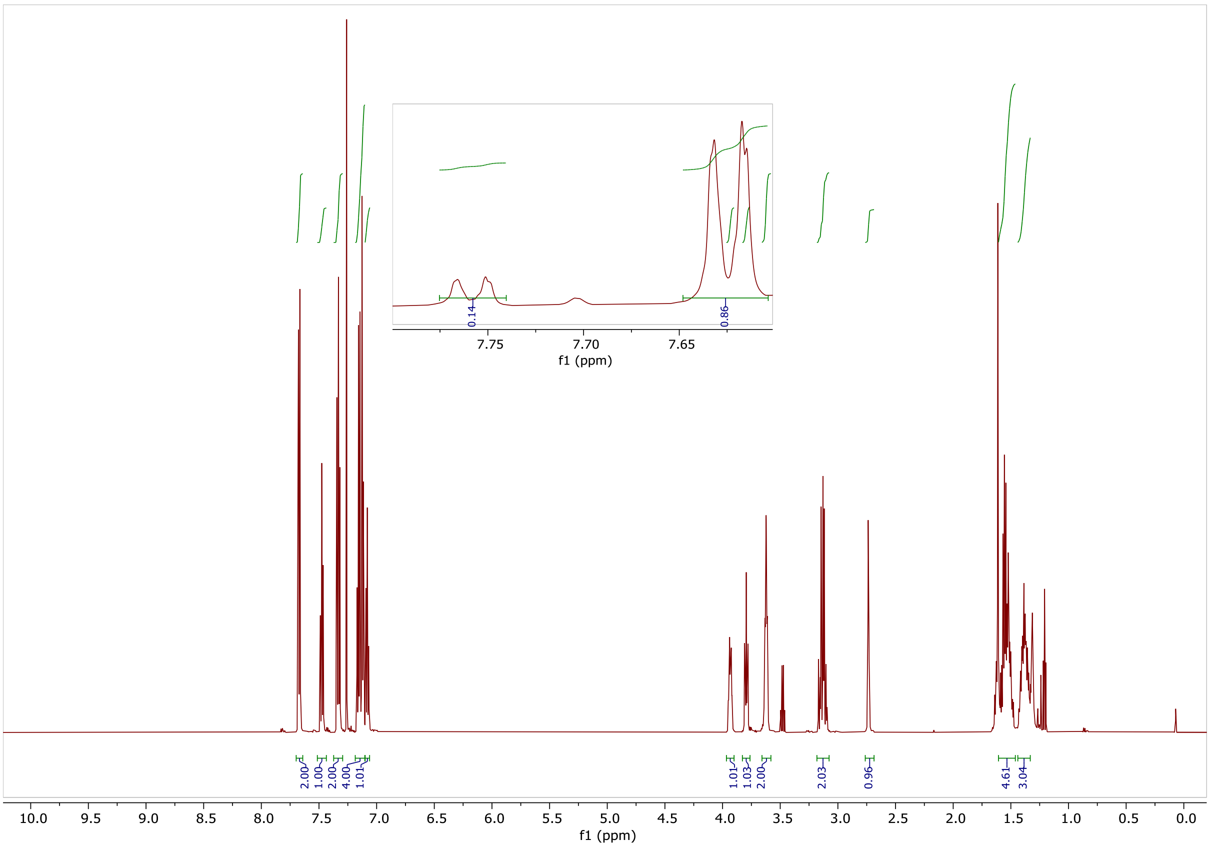

^{1}H NMR (600 MHz, $CDCl_3$) Spectrum of (2*RS*,3*RS*)-2-benzyl-3,8-dihydroxy-1-phenyl-1-octanone **4b**. Expansion: diastereoselectivity determined by ^{1}H NMR analysis of the crude reaction mixture.

S8 Mechanistic studies

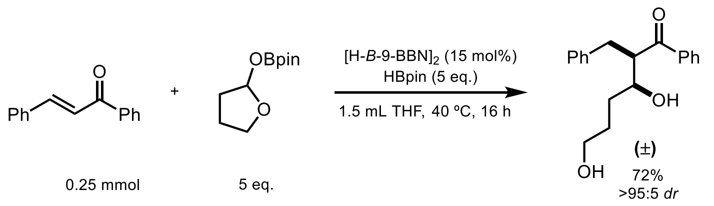

Figure S2: Catalytic reductive coupling of chalcone and lactol-Bpin.

Chalcone (1.00 eq.), 9-borabicyclo(3.3.1)nonane [H-*B*-9-BBN]$_2$ (15 mol%), pinacolborane (HBpin) (5.00 eq.) and lactol-Bpin (5.00 eq.) (prepared by reacting 2-hydroxytetrahydrofuran with 1 eq. of HBpin in THF at room temperature for 16 hours) were reacted at 40 °C for 16 hours in THF (0.17 M). The reaction was quenched with ethanolamine and the yield and diastereoselectivity was determined by analysis of the crude reaction mixture by ^{1}H NMR spectroscopy.

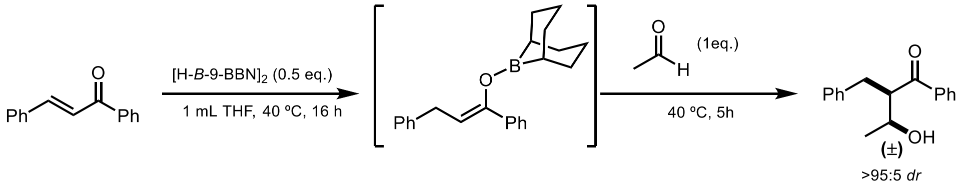

Figure S3: Aldol coupling of O-*B*-9-BBN enolate with acetaldehyde.

Chalcone (1.00 eq.) and 9-borabicyclo(3.3.1)nonane [H-*B*-9-BBN]$_2$ (0.50 eq.), were reacted in THF (0.5 M) at 40 °C for 16 hours. Then acetaldehyde (1.00 eq.) was added and the mixture was stirred at 40 °C for 5 hours. The reaction was quenched with ethanolamine and the diastereoselectivity was determined by analysis of the crude reaction mixture by ^{1}H NMR spectroscopy (>95:5 *dr*).

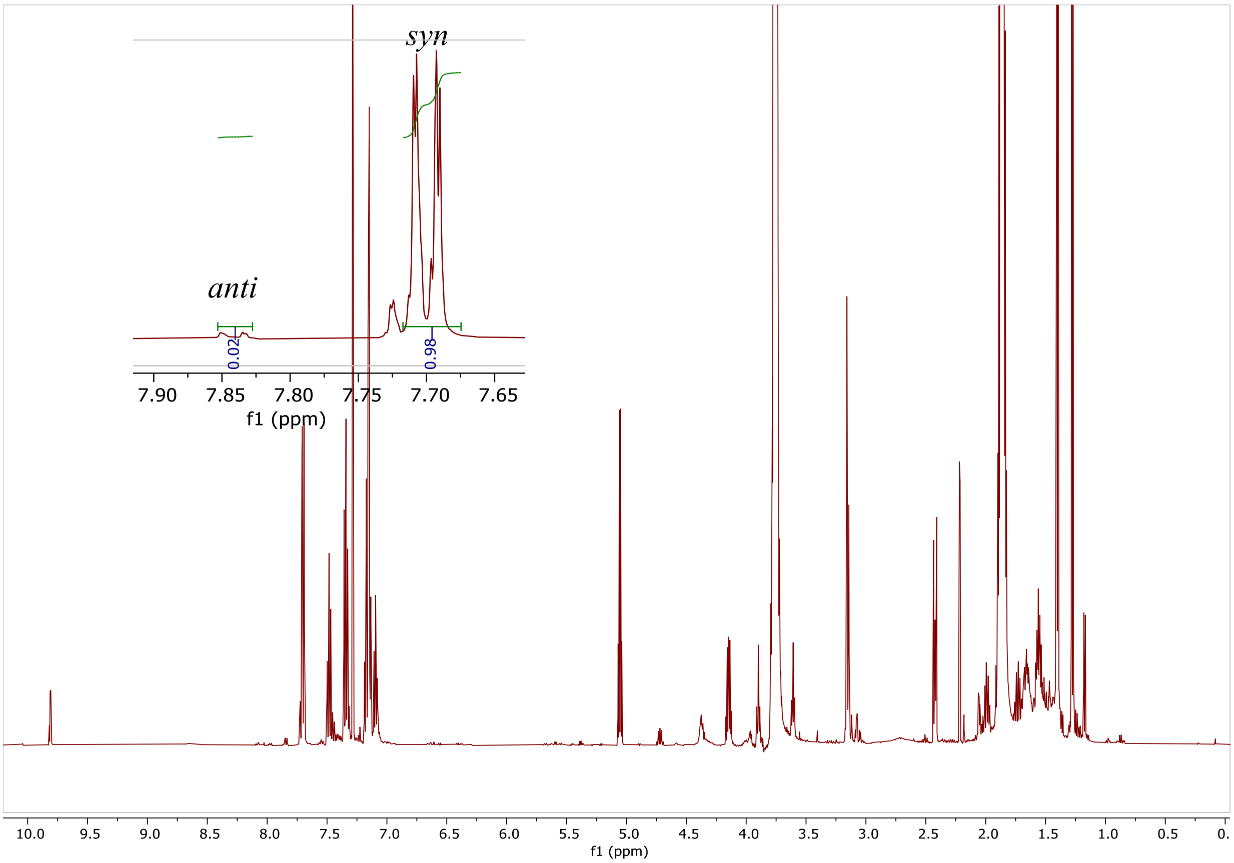

^{1}H NMR (500 MHz, $CDCl_3$) of the crude reaction mixture of the stoichiometric aldol reaction of O-*B*-9-BBN enolate with acetaldehyde.

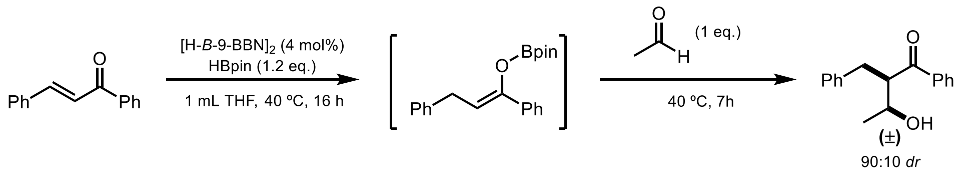

Figure S4: Aldol coupling of the O-Bpin enolate with acetaldehyde.

Chalcone (1.00 eq.), 9-borabicyclo(3.3.1)nonane [H-*B*-9-BBN]$_2$ (0.04 eq.) and 4,4,5,5-tetramethyl-1,3,2-dioxaborolane (HBpin) (1.20 eq.) were reacted in THF (0.5 M) at 40 °C for 16 hours. Then acetaldehyde (1.00 eq.) was added and the mixture was stirred at 40 °C for 7 hours. The reaction was quenched with ethanolamine and the diastereoselectivity was determined by analysis of the crude reaction mixture by ^{1}H NMR spectroscopy (90:10 *dr*).

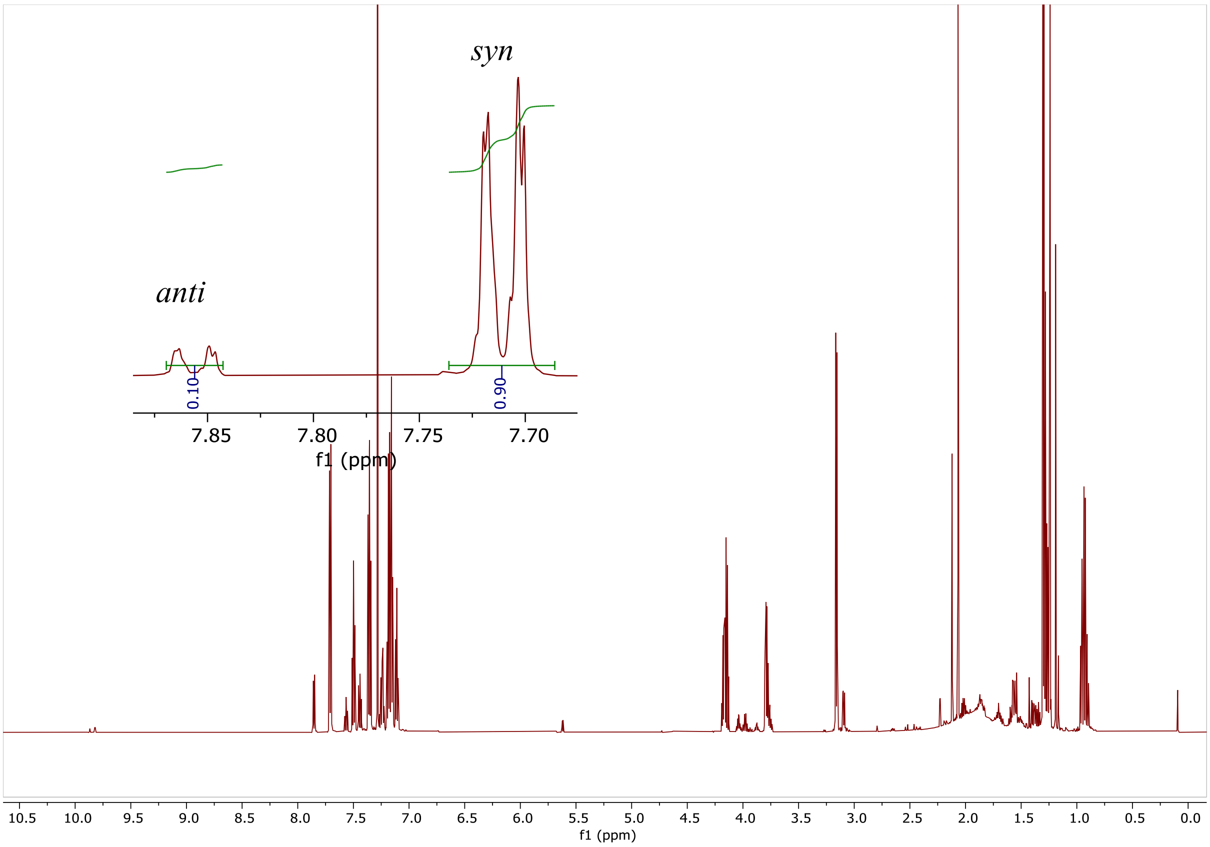

^{1}H NMR (500 MHz, $CDCl_3$) of the crude reaction mixture of the stoichiometric aldol reaction of O-Bpin enolate with acetaldehyde.

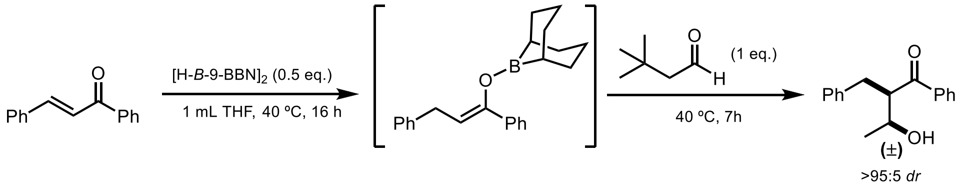

Figure S5: Aldol coupling of O-*B*-9-BBN enolate with 3,3-dimethylbutyraldehyde.

Chalcone (1.00 eq.) and 9-borabicyclo(3.3.1)nonane [H-*B*-9-BBN]$_2$ (0.50 eq.), were reacted in THF (0.5 M) at 40 °C for 16 hours. Then 3,3-dimethylbutyraldehyde (1.00 eq.) was added and the mixture was stirred at 40 °C for 5 hours. The reaction was quenched with ethanolamine and the diastereoselectivity was determined by analysis of the crude reaction mixture by ^{1}H NMR spectroscopy (>95:5 *dr*).

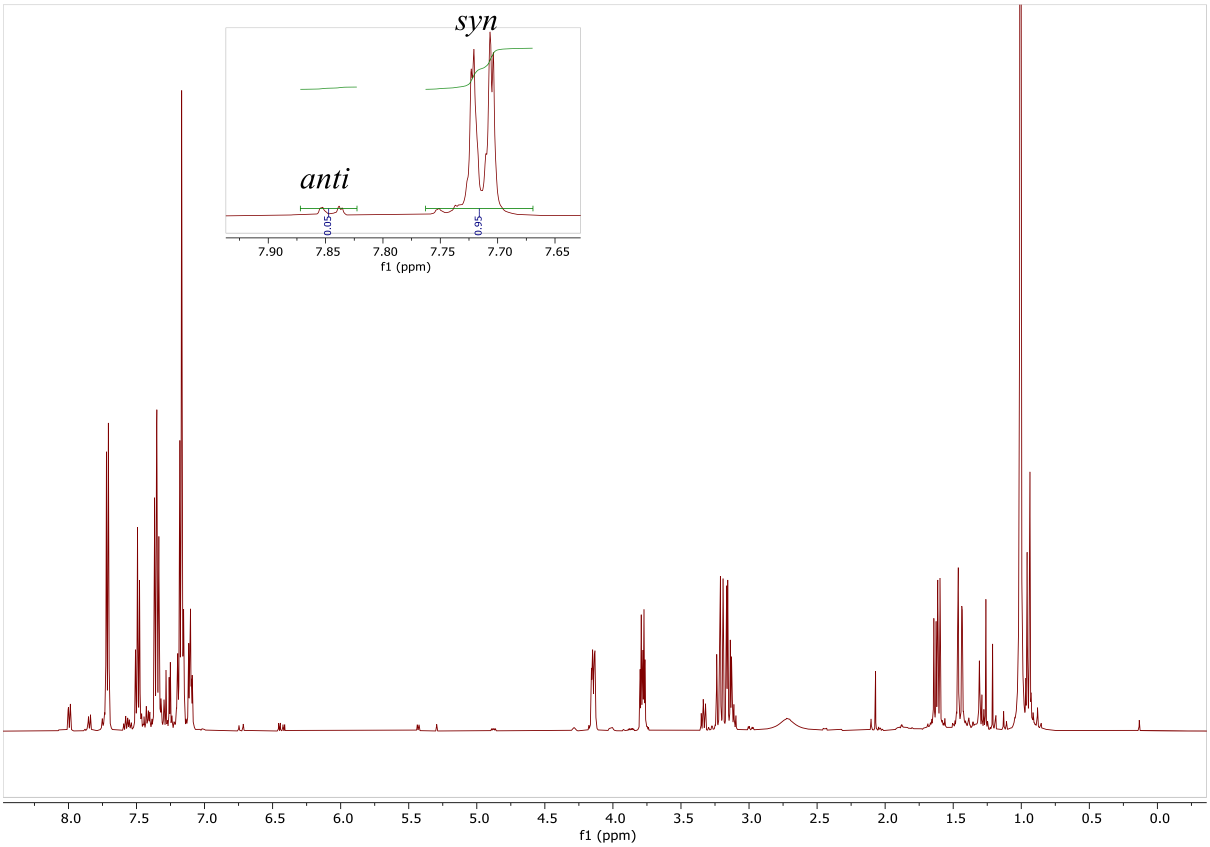

^{1}H NMR (400 MHz, $CDCl_3$) of the crude reaction mixture of the stoichiometric aldol reaction of O-*B*-9-BBN enolate with 3,3-dimethylbutyraldehyde.

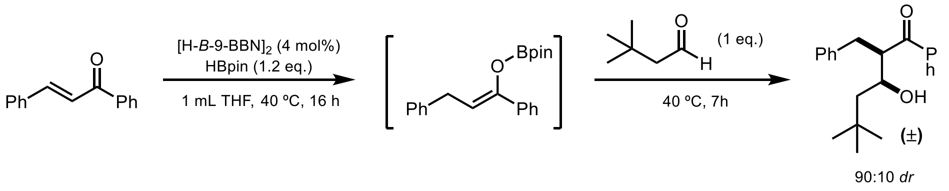

Figure S6: Aldol coupling of O-Bpin enolate with 3,3-dimethylbutyraldehyde.

Chalcone (1.00 eq.), 9-borabicyclo(3.3.1)nonane [H-*B*-9-BBN]$_2$ (0.04 eq.) and 4,4,5,5-tetramethyl-1,3,2-dioxaborolane (HBpin) (1.20 eq.) were reacted in THF (0.5 M) at 40 °C for 16 hours. Then 3,3-dimethylbutyraldehyde (1.00 eq.) was added and the mixture was stirred at 40 °C for 7 hours. The reaction was quenched with ethanolamine and the diastereoselectivity was determined by analysis of the crude reaction mixture by ^{1}H NMR spectroscopy (90:10 *dr*).

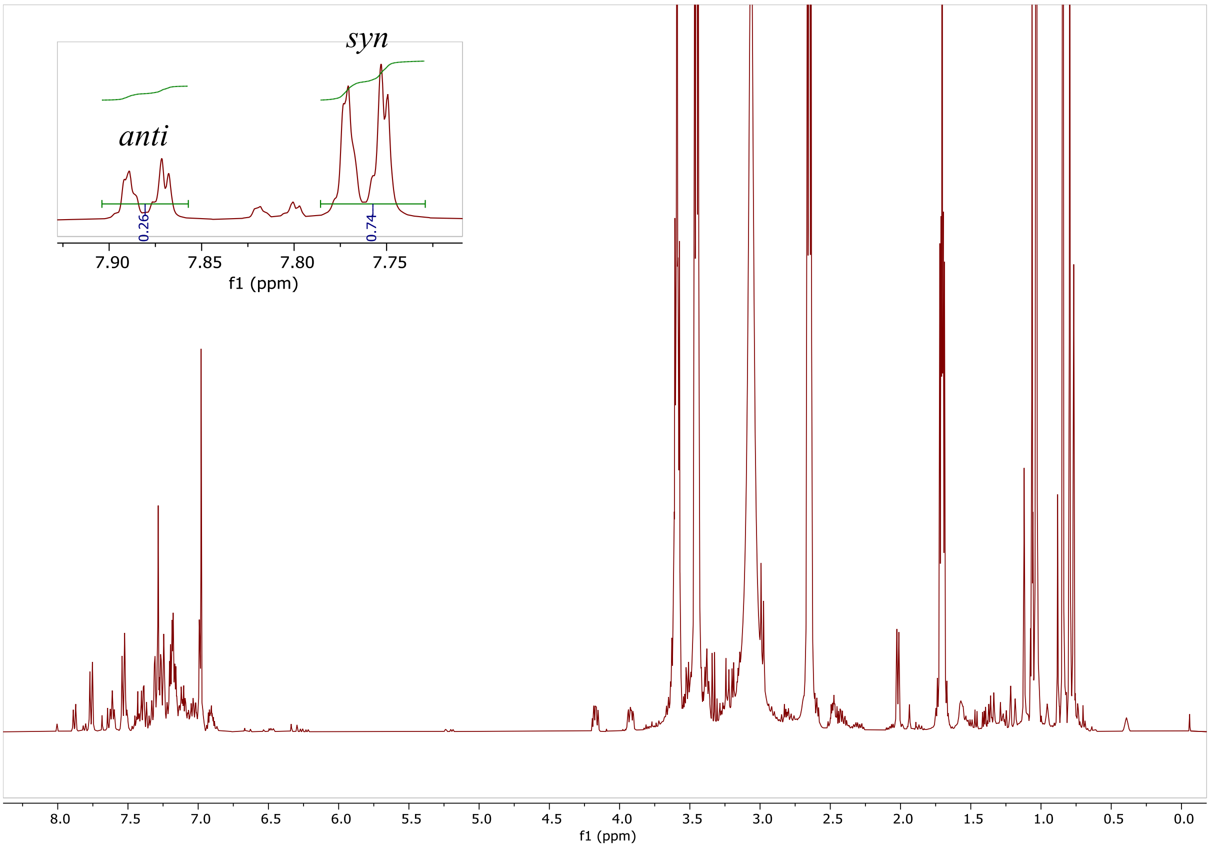

^{1}H NMR (400 MHz, $CDCl_3$) of the crude reaction mixture of the stoichiometric aldol reaction of O-Bpin enolate with 3,3-dimethylbutyraldehyde.

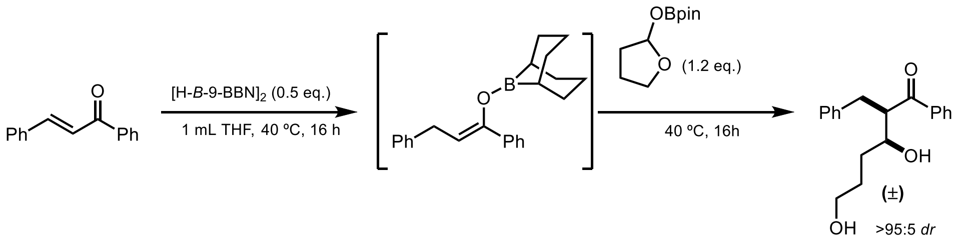

Figure S7: Aldol coupling of the O-*B*-9-BBN enolate with lactol-Bpin

Chalcone (1.00 eq.) and 9-borabicyclo(3.3.1)nonane [H-*B*-9-BBN]$_2$ (0.50 eq.), were reacted in THF (0.5 M) at 40 °C for 16 hours. Then lactol-Bpin (1.20 eq.) was added and the mixture was stirred at 40 °C for 16 hours. The reaction was quenched with ethanolamine and the diastereoselectivity was determined by analysis of the crude reaction mixture by ^{1}H NMR spectroscopy (>95:5 *dr*).

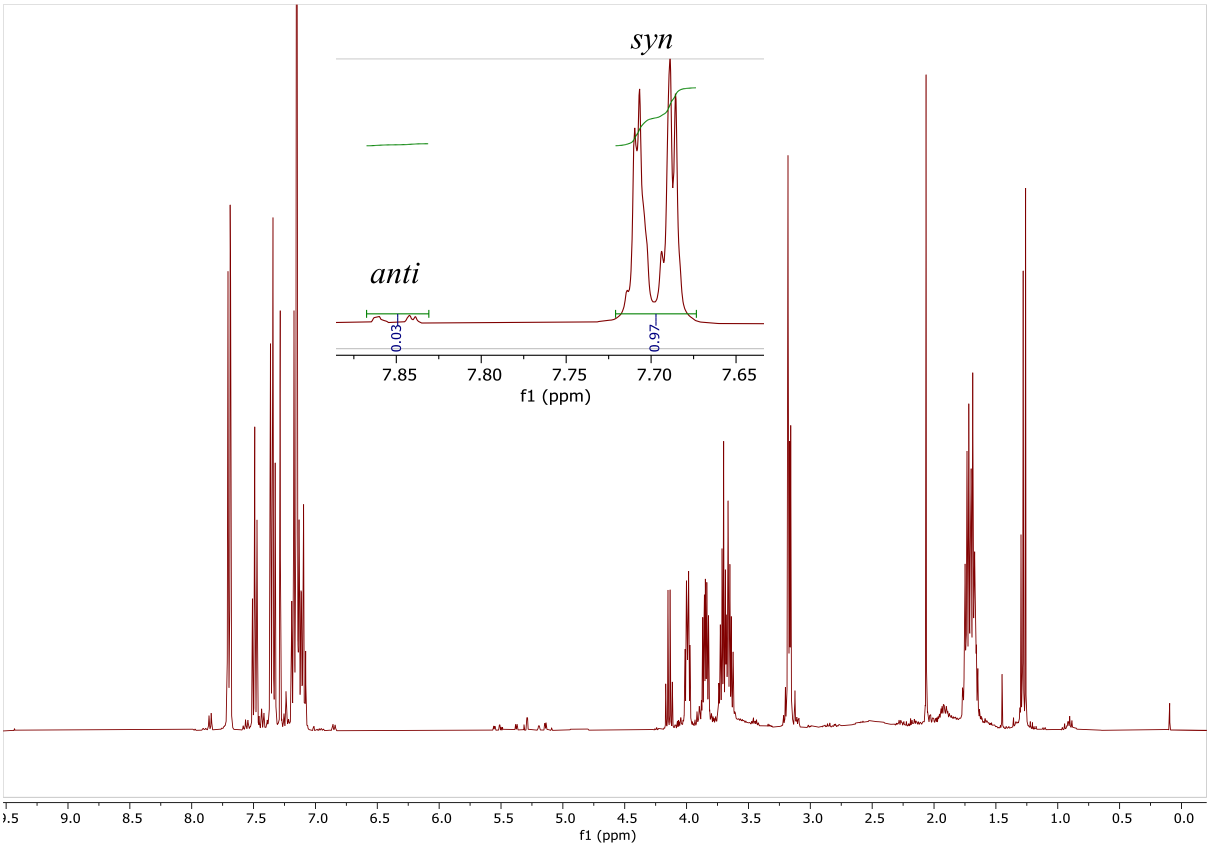

^{1}**H NMR** (500 MHz, $CDCl_3$) of the crude reaction mixture of the stoichiometric aldol reaction of O-*B*-9-BBN enolate with lactol-Bpin.

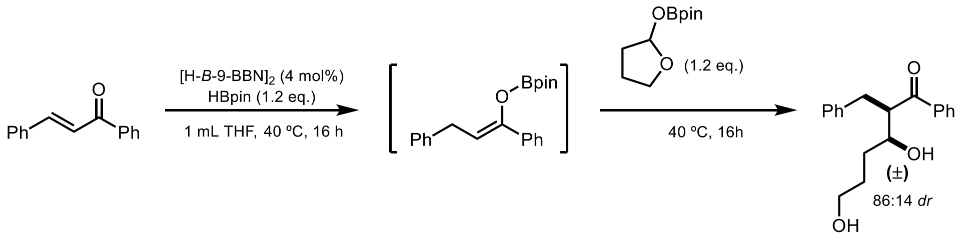

Figure S8: Aldol coupling of O-Bpin enolate with lactol-Bpin

Chalcone (1.00 eq.), 9-borabicyclo(3.3.1)nonane [H-*B*-9-BBN]$_2$ (0.04 eq.) and 4,4,5,5-tetramethyl-1,3,2-dioxaborolane (HBpin) (1.20 eq.) were reacted in THF (0.5 M) at 40 °C for 16 hours. Then lactol-Bpin (1.20 eq.) was added and the mixture was stirred at 40 °C for 16 hours. The reaction was quenched with ethanolamine and the diastereoselectivity was determined by analysis of the crude reaction mixture by ^{1}H NMR spectroscopy (86:14 *dr*).

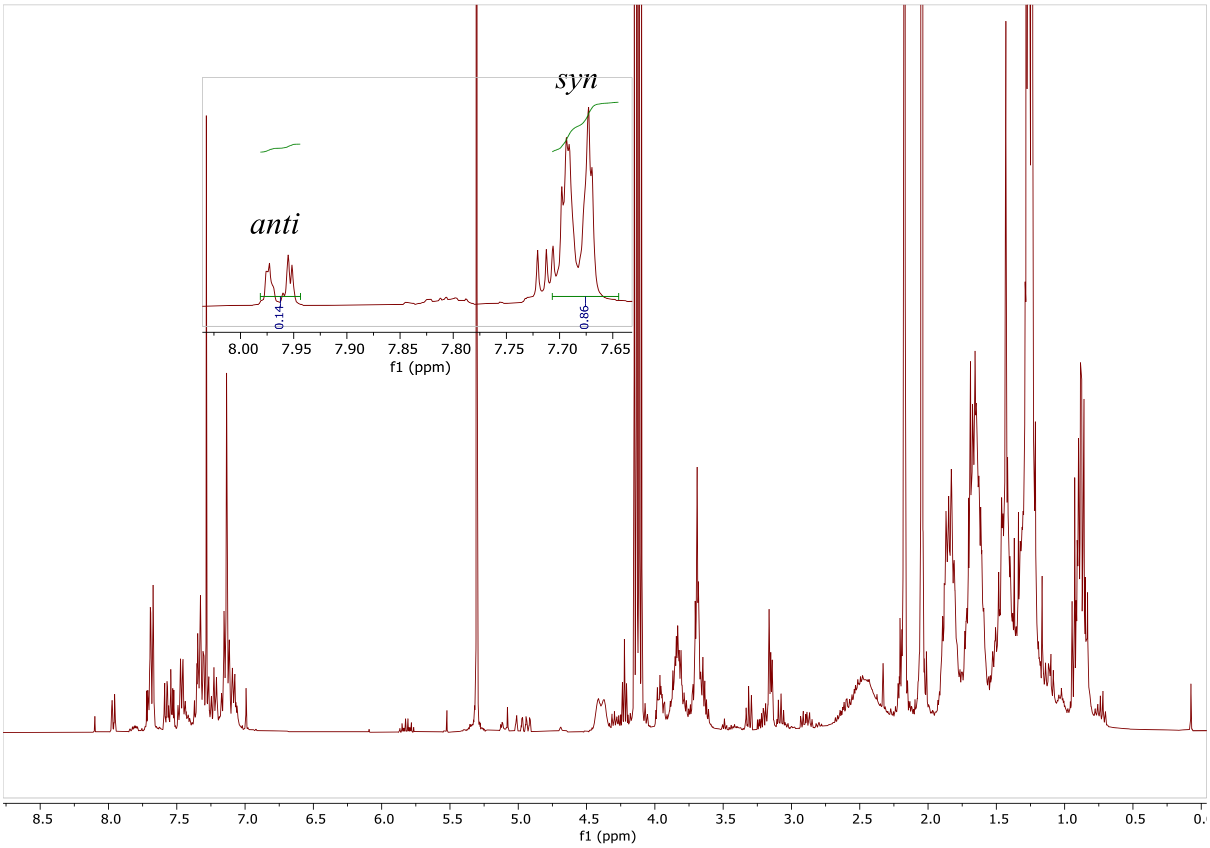

^{1}H NMR (500 MHz, $CDCl_3$) of the crude reaction mixture of the stoichiometric aldol reaction of O-Bpin enolate with lactol-Bpin.

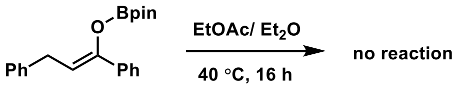

To an argon filled vial was added chalcone (104 mg, 0.5 mmol), [H-*B*-9-BBN]$_2$ (6 mg, 0.025 mmol), HBpin (87 µL, 0.6 mmol) and diethyl ether (1 mL). The mixture was stirred for 16 hours at 40 °C till enolate formation upon which ethyl acetate (1 mL) was added. The mixture was stirred for 16 hours at 40 °C upon which the reaction mixture was quenched with SiO_2 and analysed by ^{1}H NMR spectroscopy. No formation of diketone product was observed.

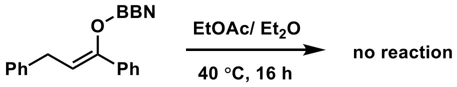

To an argon filled vial was added chalcone (20 mg, 0.1 mmol), [H-*B*-9-BBN]$_2$ (12 mg, 0.05 mmol), and diethyl ether (1 mL). The mixture was stirred for 16 hours at 40 °C until enolate formation was completed upon which ethyl acetate (1 mL) was added. The mixture was stirred for 16 hours at 40 °C upon which the reaction mixture was quenched with SiO_2 and analysed by ^{1}H NMR spectroscopy. No formation of diketone product was observed.

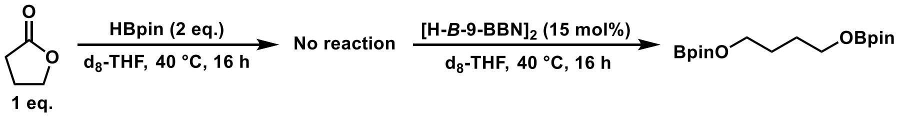

To an argon filled NMR tube was added γ-butyrolactone (25 mg, 0.25 mmol), HBpin (73 µL, 0.5 mmol), and d_8-THF (0.4 mL). The mixture was heated for 16 hours at 40 °C and analysed by ^{1}H NMR spectroscopy. [H-*B*-9-BBN]$_2$ (15 mol%) was then added and the mixture was heated for another 16 hours at 40 °C and analysed by ^{1}H NMR spectroscopy

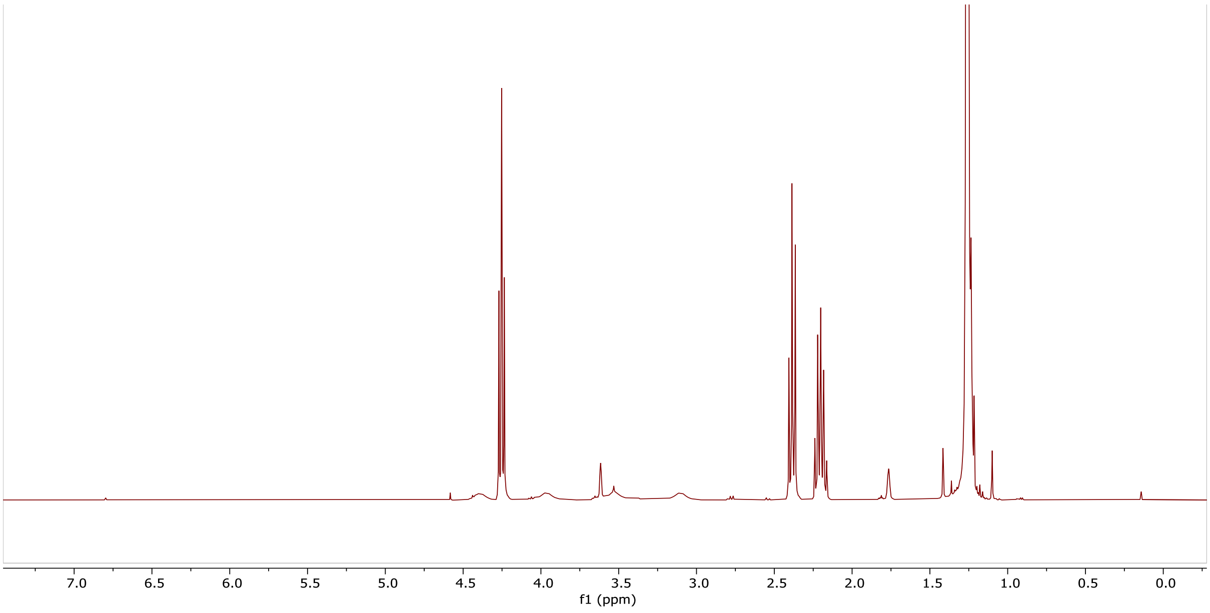

^{1}H NMR of the reaction mixture of γ-butyrolactone with HBpin.

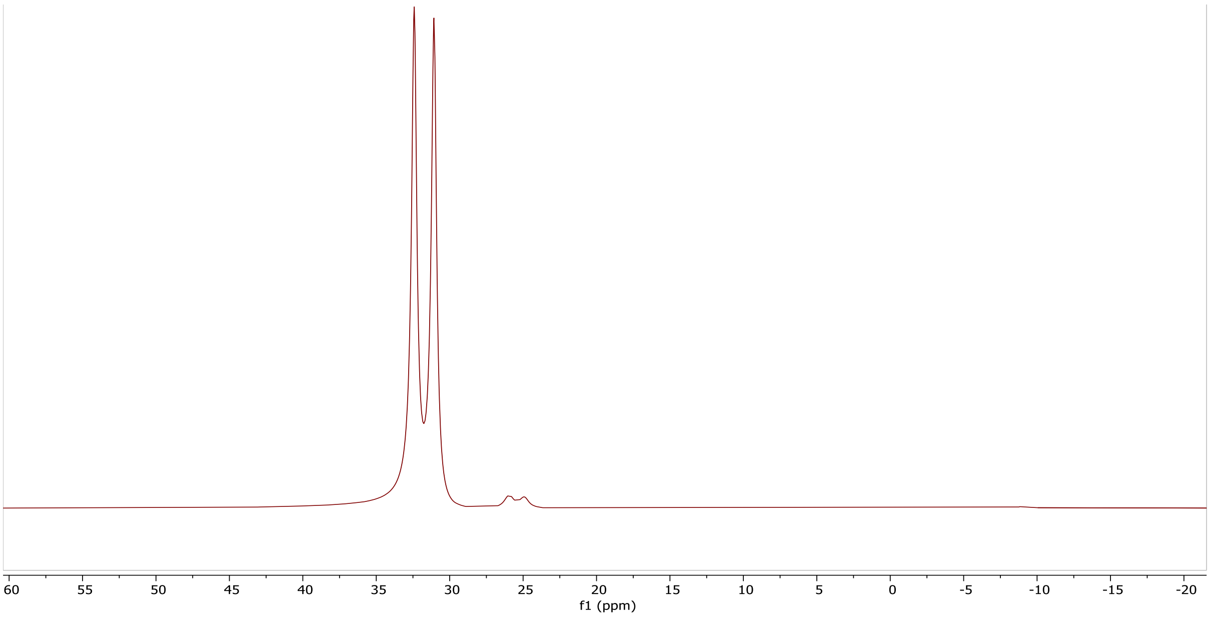

^{11}B NMR of the reaction mixture of 2-hydroxytetrahydrofuran with HBpin.

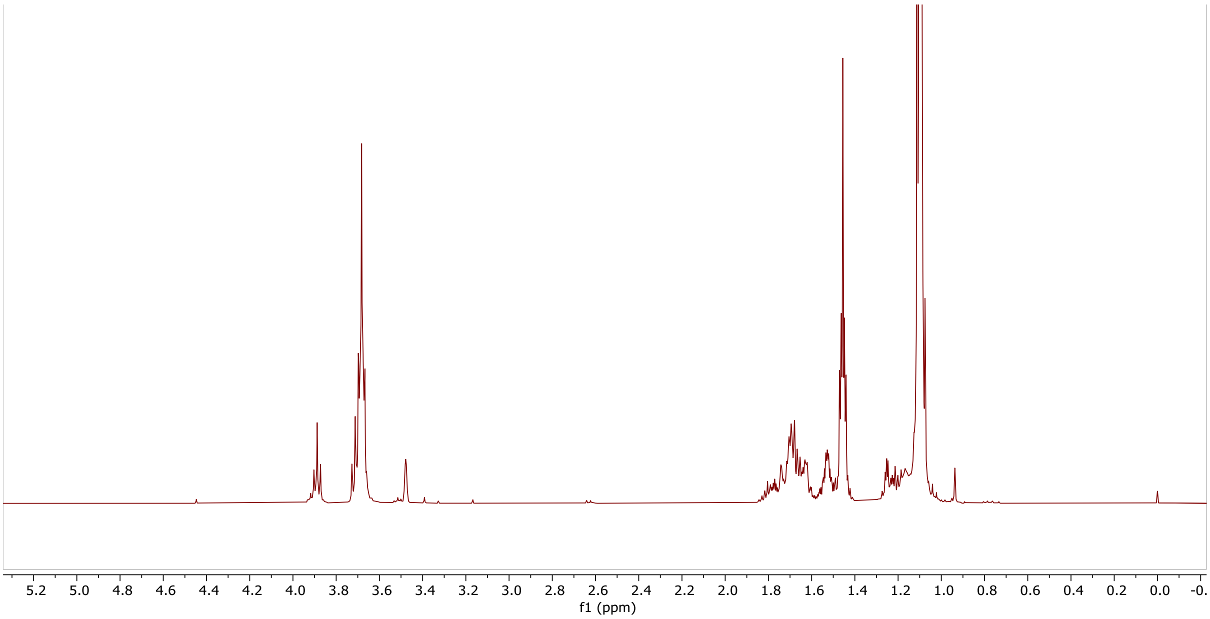

^{1}H NMR of the reaction mixture of γ-butyrolactone with HBpin and H-*B*-9-BBN.

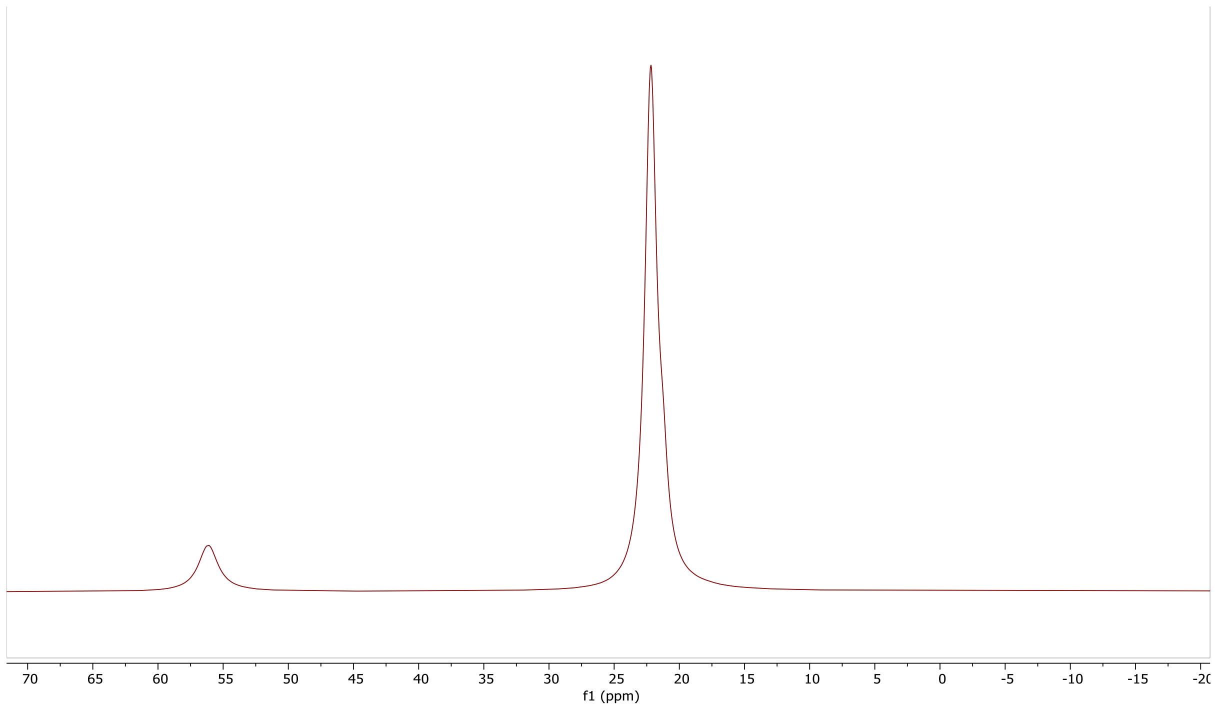

^{11}B NMR of the reaction mixture of γ-butyrolactone with HBpin and H-*B*-9-BBN.

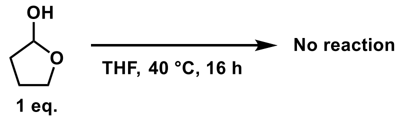

To an argon filled NMR tube was added 2–hydroxytetrahydrofuran (9 mg, 0.1 mmol) and d_8-THF (0.4 mL). The mixture was heated for 16 hours at 40 °C and analysed by ^{1}H NMR spectroscopy. No reaction was observed.

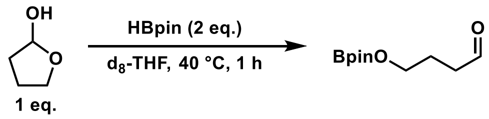

To an argon filled NMR tube was added 2–hydroxytetrahydrofuran (18 mg, 0.25 mmol), HBpin (73 μL, 0.5 mmol) and d_8-THF (0.4 mL). The mixture was heated for 1 hour at 40 °C and analysed by ^{1}H NMR spectroscopy.

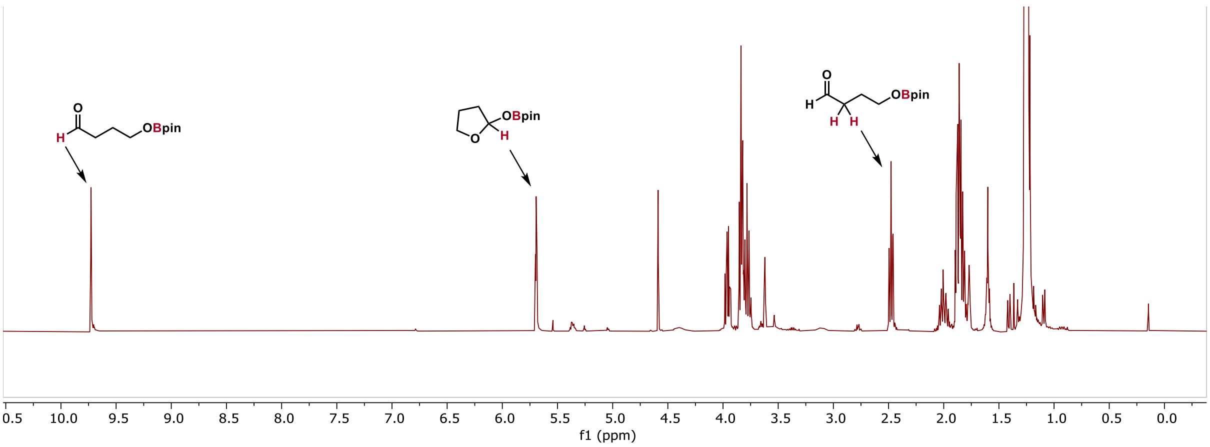

^{1}H NMR of the reaction mixture of 2-hydroxytetrahydrofuran with HBpin.

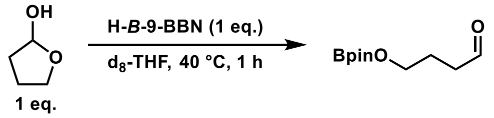

To an argon filled NMR tube was added 2–hydroxytetrahydrofuran (9 mg, 0.1 mmol), [H-*B*-9-BBN]$_2$ (24 mg, 0.1 mmol), and d$_8$-THF (0.4 mL). The mixture was heated for 1 hour at 40 °C and analysed by ^{1}H NMR spectroscopy.

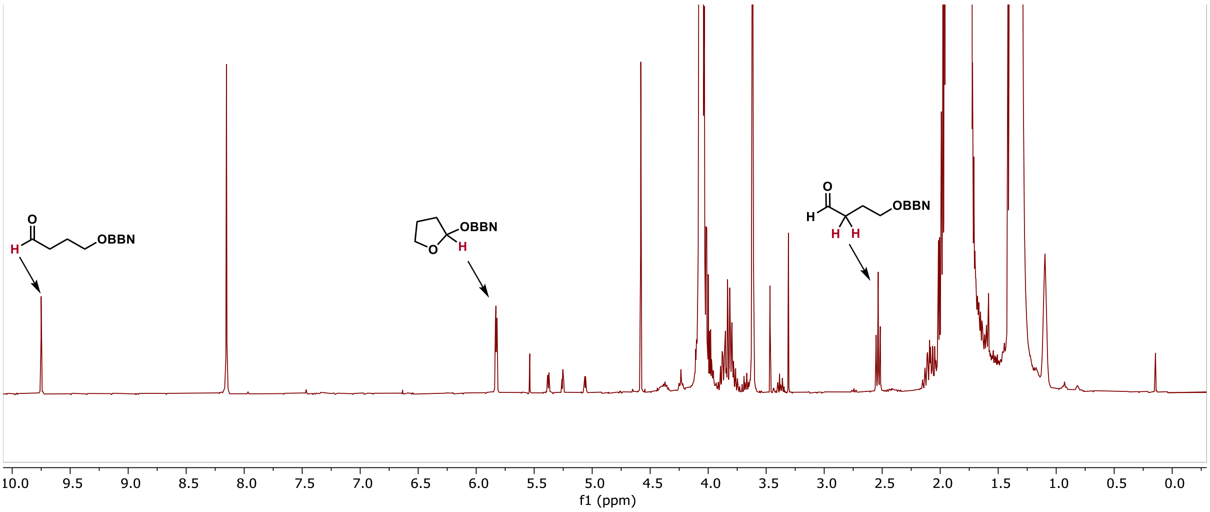

S9 Incompatible substrates

a) incompatible enones

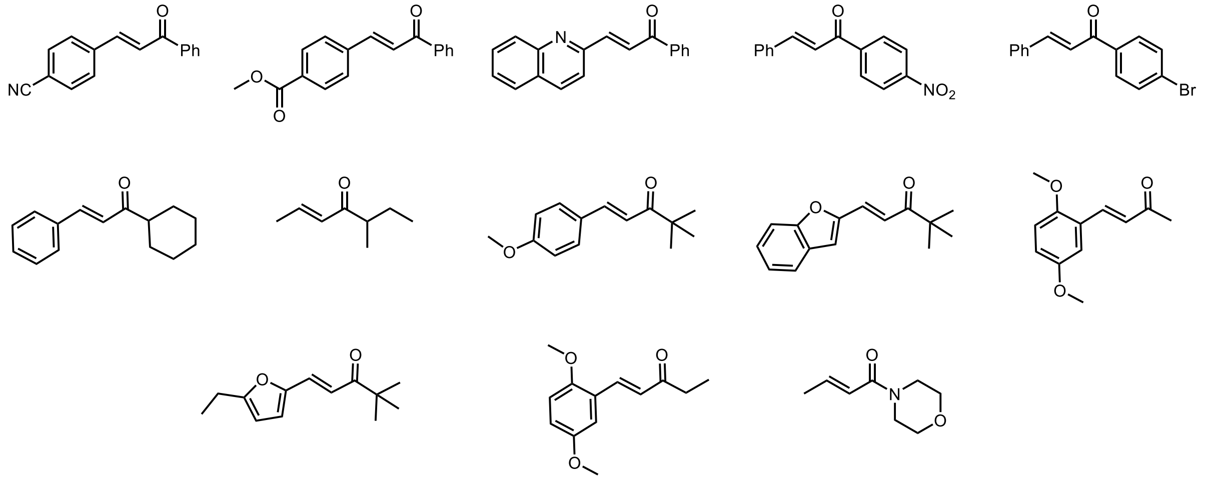

b) incompatible esters

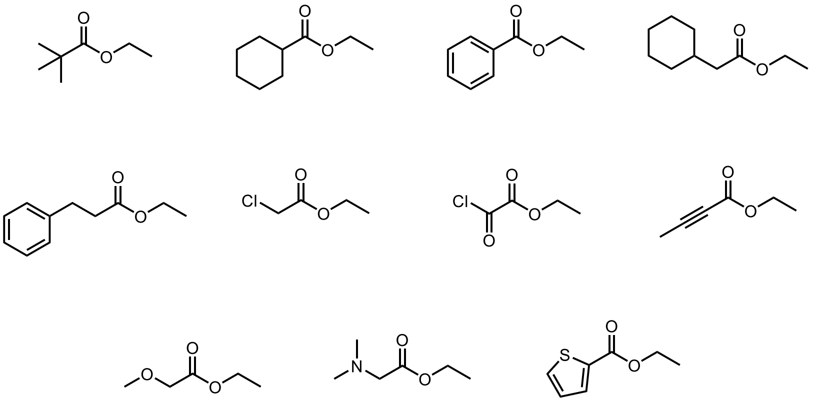

c) incompatible lactones

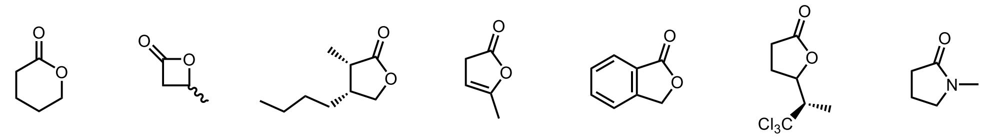

Figure S9: a) incompatible enones b) incompatible esters c) incompatible lactones.

S10 Study of diastereoselectivity over time

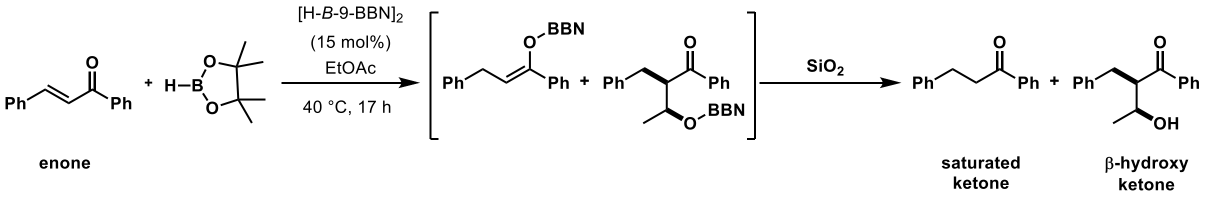

Figure S10: borane catalyzed enone-ester coupling.

In an argon filled glovebox to a reaction vial was added 1,3,5-trimethoxybenzene (16.8 mg, internal standard), chalcone (104 mg, 0.5 mmol), [H-*B*-9-BBN]$_2$ (18 mg, 0.075 mmol), HBpin (363 µL, 2.5 mmol) and EtOAc (5 mL). The reaction mixture was heated at 40 °C for 17 hours. Aliquots (0.1 mL) were taken from the reaction mixture at regular time points and quenched with ethanoamine. The resulting crude, hydrolyzed mixtures were analyzed by ^{1}H NMR spectroscopy using peaks at 6.10 and 3.81 ppm (1,3,5-trimethoxybenzene) at 8.01 (enone), 7.93 (saturated ketone), 7.75 ppm (β-hydroxy ketone, minor diastereomer) and 7.65 ppm (β-hydroxy ketone, major diastereomer) to quantify product ratios.

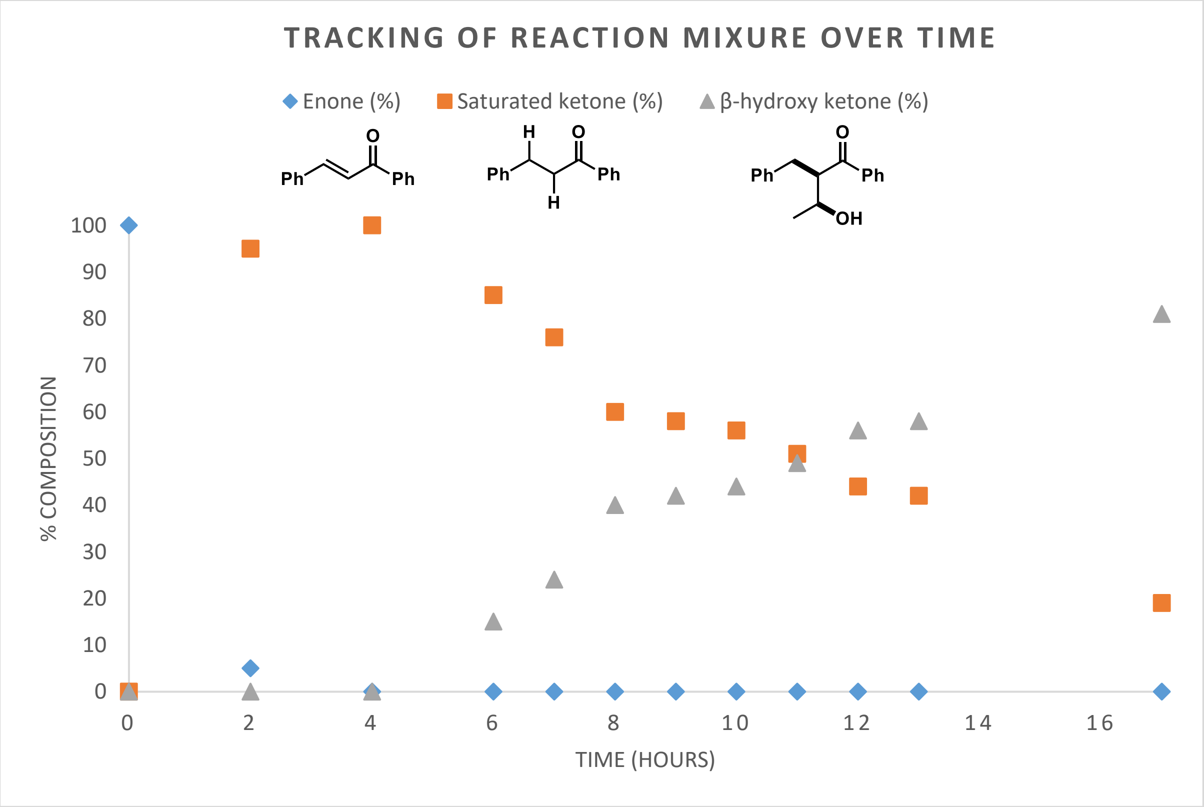

Figure S11: Tracking of reaction mixture over time.

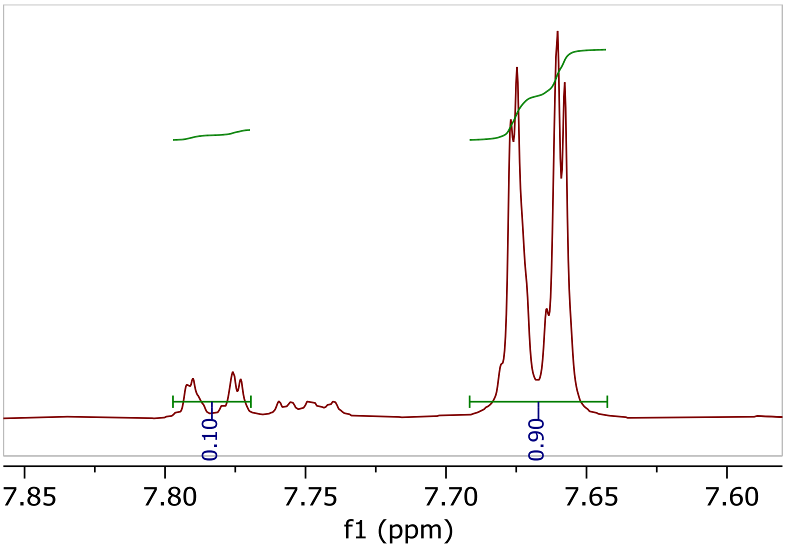

Figure S12: Diastereoselectivity of the aldol product after 11 hours of reaction.

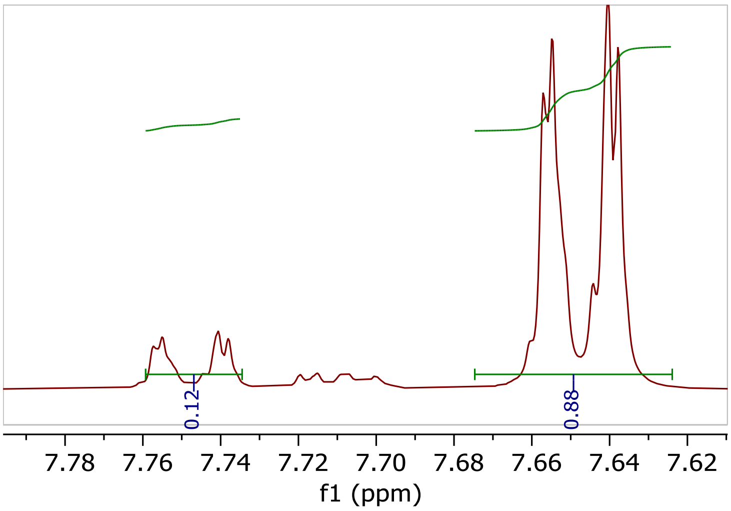

Figure S13: Diastereoselectivity of the aldol product after 12 hours of reaction.

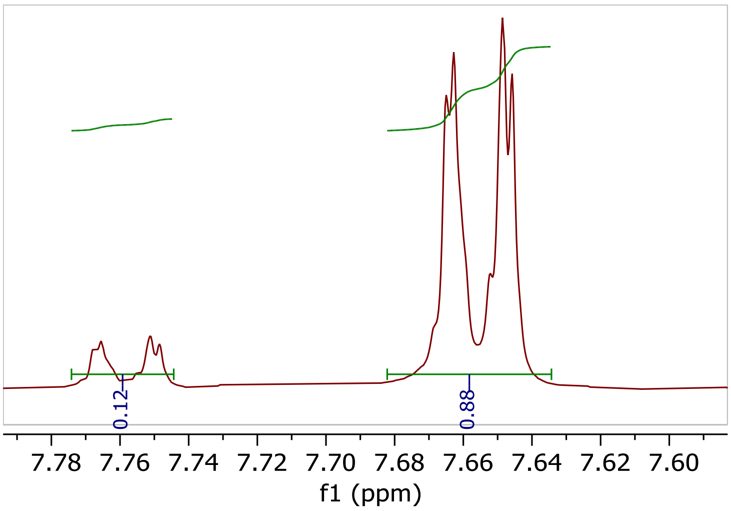

Figure S14: Diastereoselectivity of the aldol product after 13 hours of reaction.

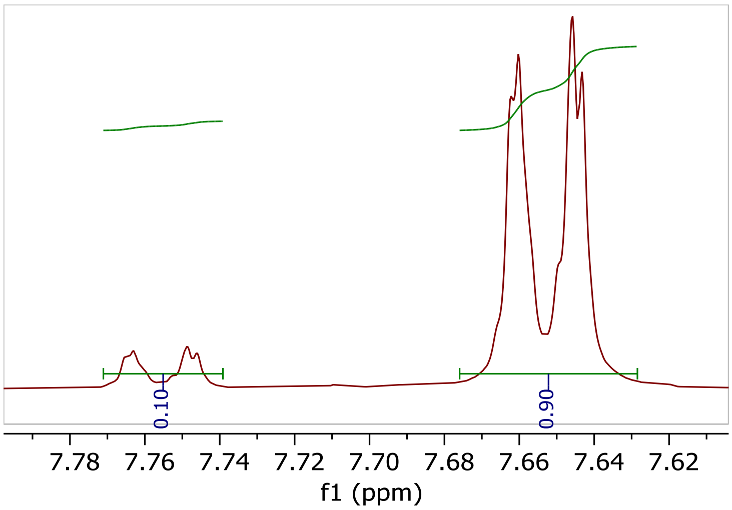

Figure S15: Diastereoselectivity of the aldol product after 17 hours of reaction.

Table S2: Tracking of reaction mixture over time

Entry	Time (h)	Enone (%)	Saturated ketone (%)	β-hydroxy ketone (%)	*d.r.*
1	0	100	0	0	
2	2	5	95	0	
3	4	0	100	0	
4	6	0	85	15	
5	7	0	76	24	
6	8	0	60	40	
7	9	0	58	42	
8	10	0	56	44	
9	11	0	51	49	90/10
10	12	0	44	56	88/12
11	13	0	42	58	88/12
12	17	0	19	81	90/10

Diastereomeric ratio are only shown where both diasteromers represent >5% of total integration in the ^{1}H NMR Spectrum.

S11 Spectroscopic data

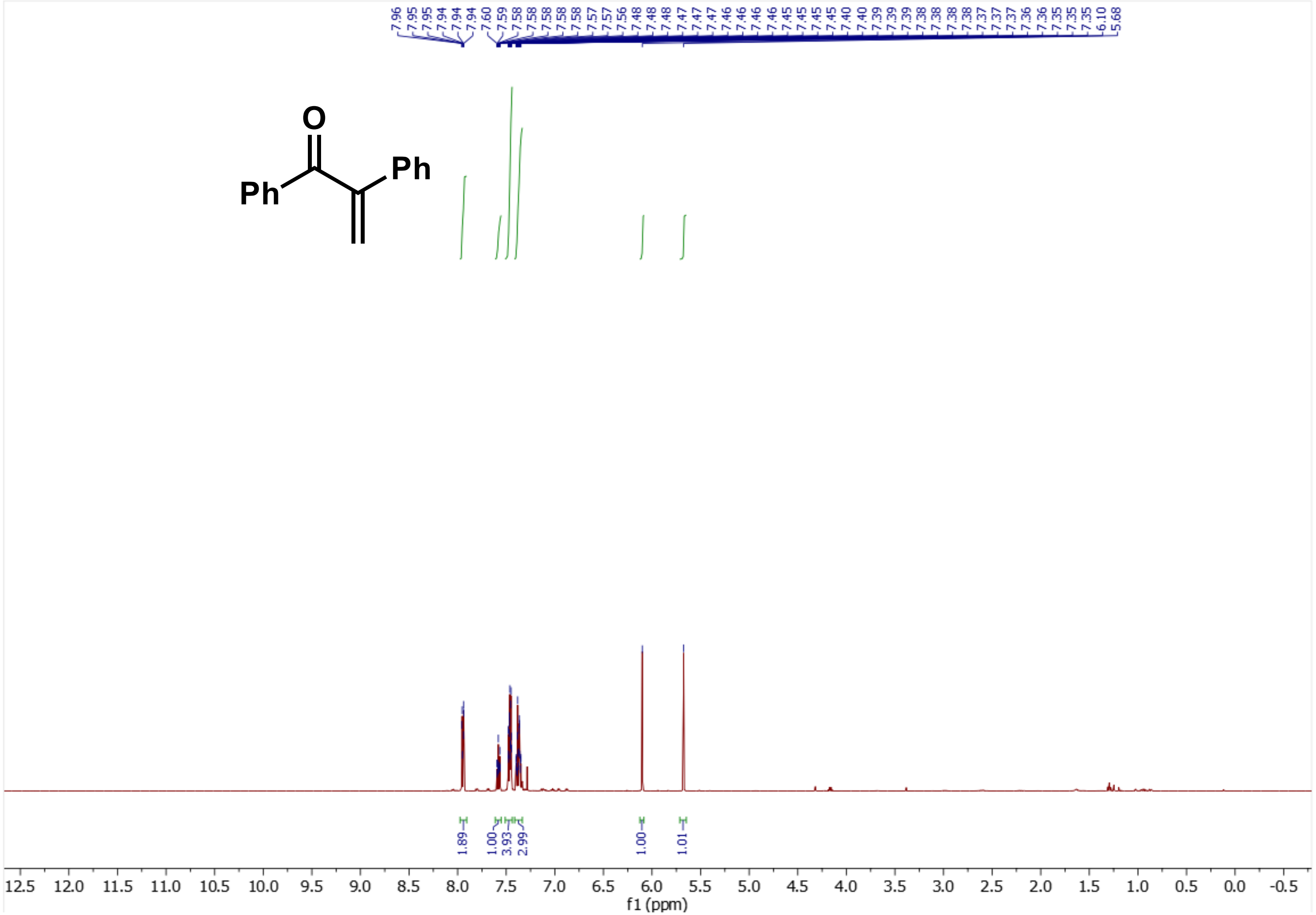

^{1}H NMR (500 MHz, $CDCl_3$) Spectrum of 1,2-diphenylprop-2-en-1-one.

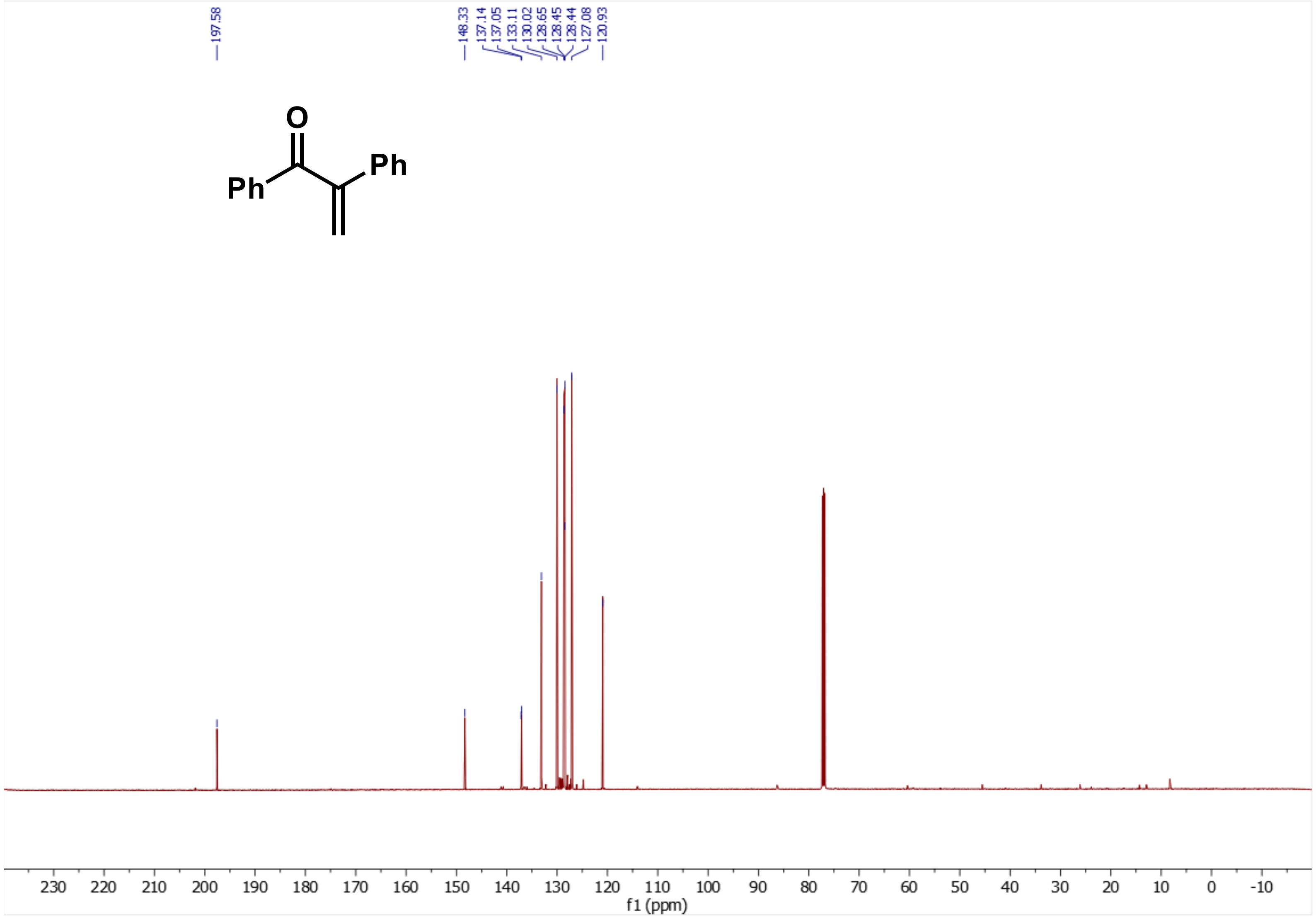

^{13}C NMR (126 MHz, CDCl$_3$) Spectrum of 1,2-diphenylprop-2-en-1-one.

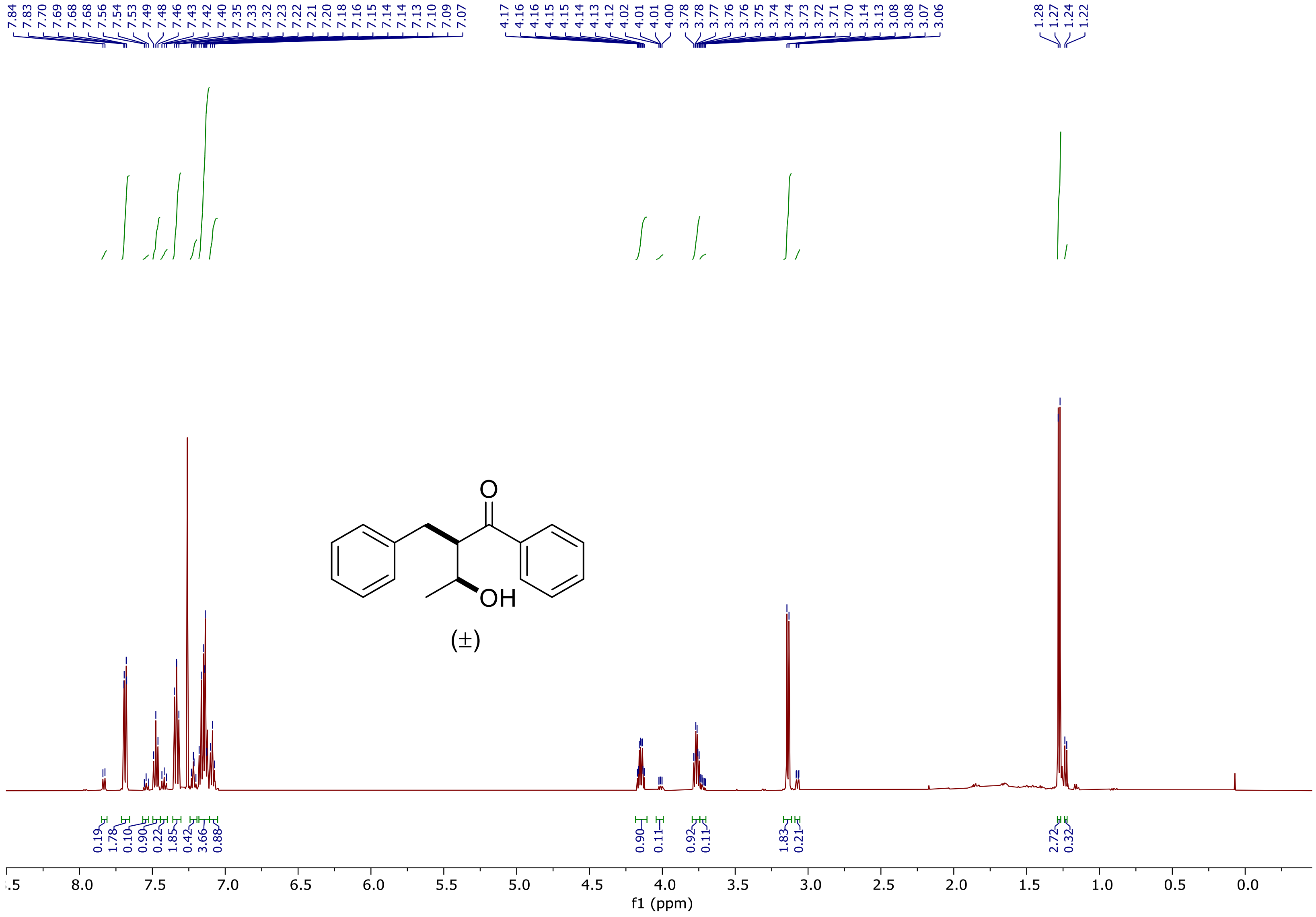

^{1}H NMR (500 MHz, $CDCl_3$) Spectrum of (*2RS,3RS*)-2-benzyl-3-hydroxy-1-phenyl-1-butanone **3a**.

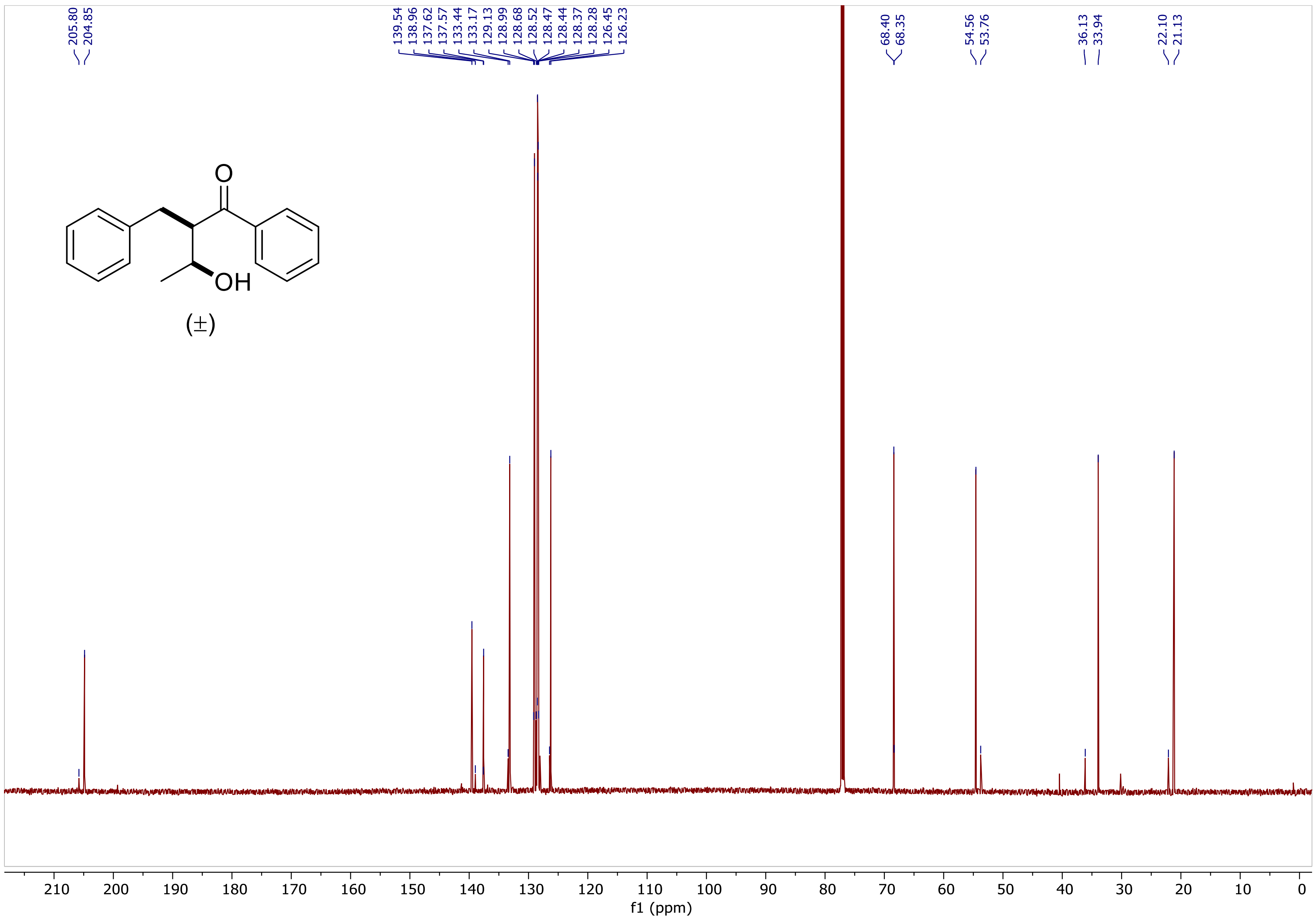

^{13}C NMR (126 MHz, $CDCl_3$) Spectrum of (*2RS,3RS*)-2-benzyl-3-hydroxy-1-phenyl-1-butanone **3a**.

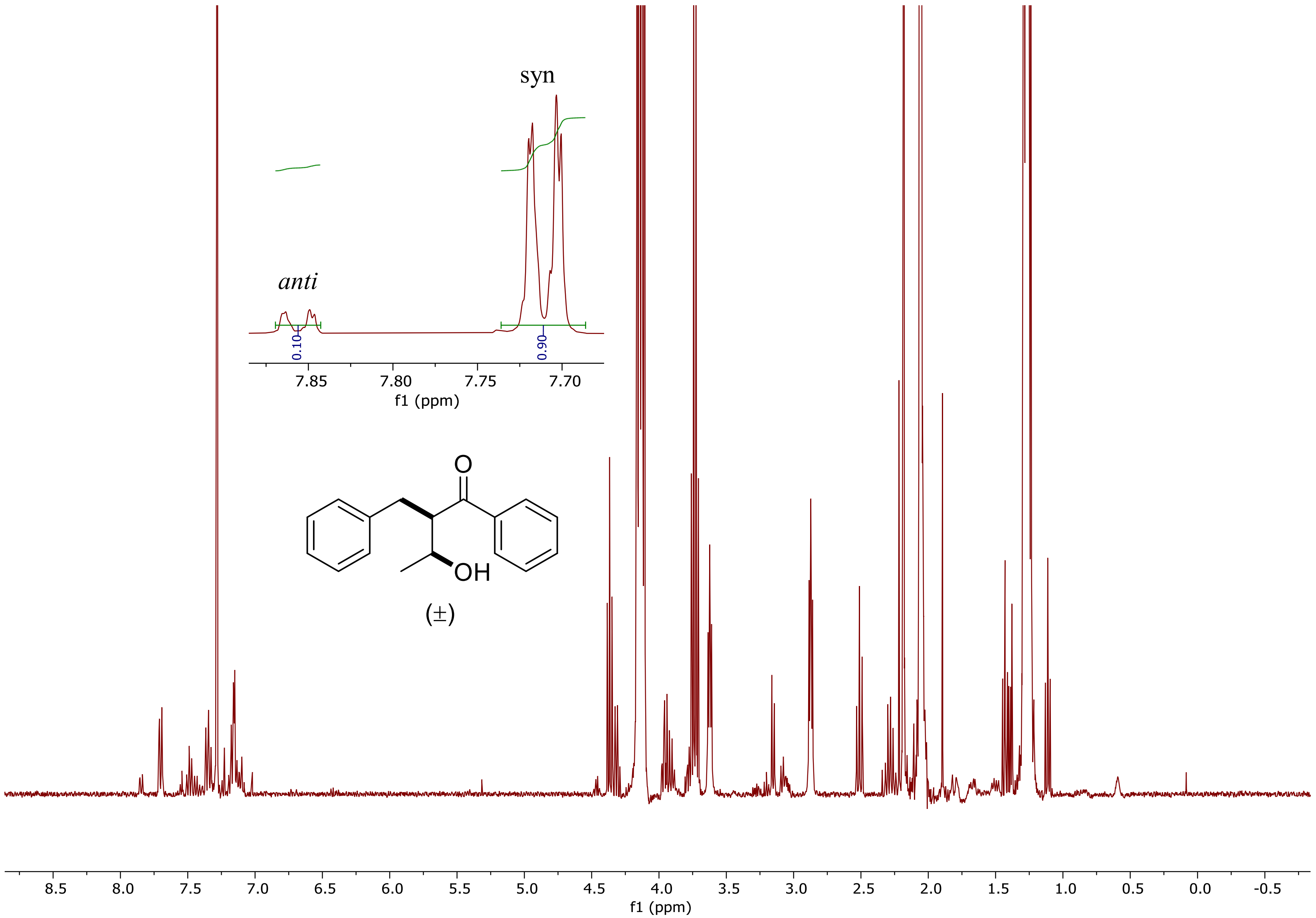

^{1}H NMR (400 MHz, $CDCl_3$) Spectrum of the crude reaction mixture for (*2RS,3RS*)-2-benzyl-3-hydroxy-1-phenyl-1-butanone **3a**.

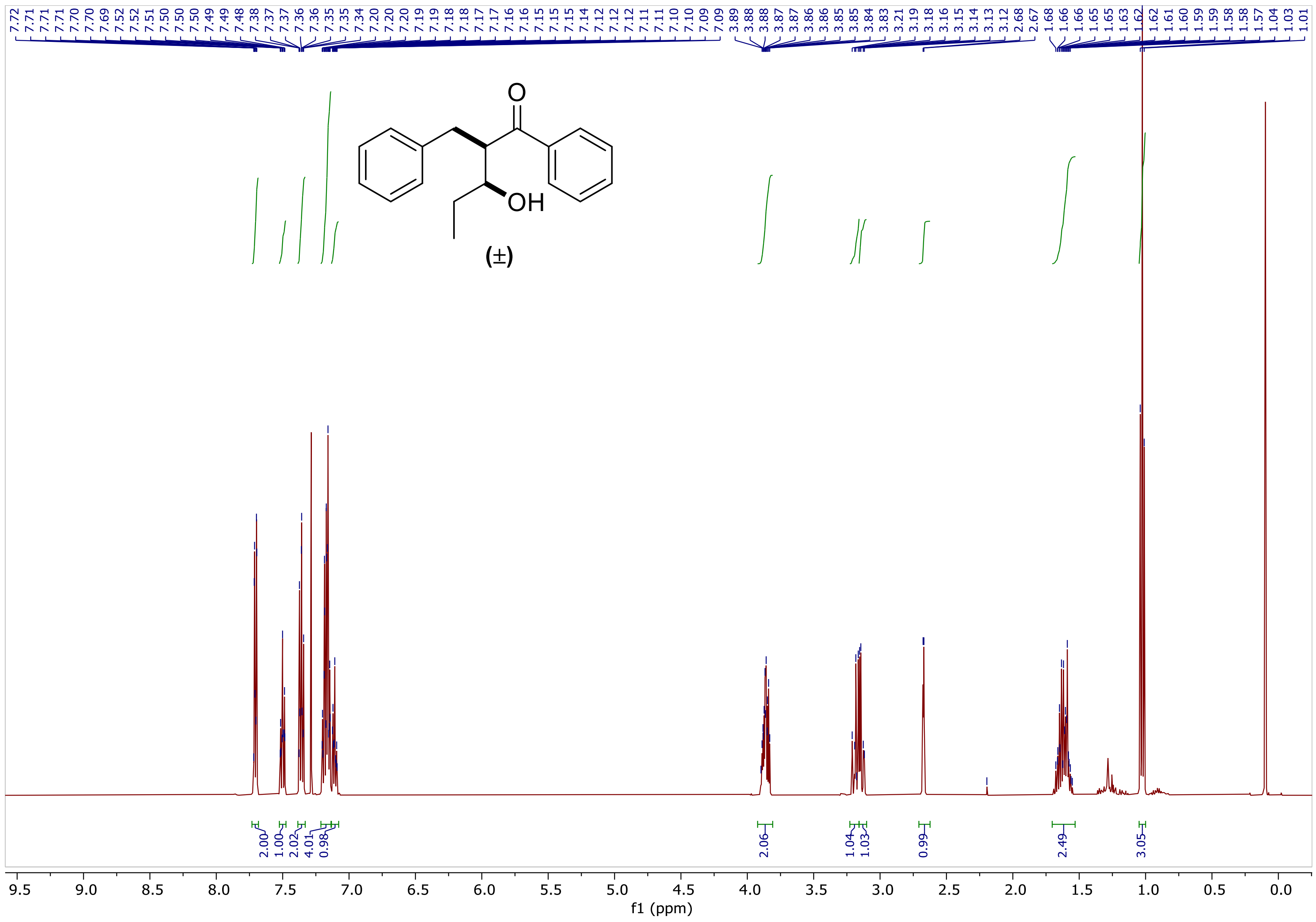

^{1}H NMR (500 MHz, $CDCl_3$) Spectrum of (*2RS,3RS*)-2-benzyl-3-hydroxy-1-phenyl-1-pentanone **3b**.

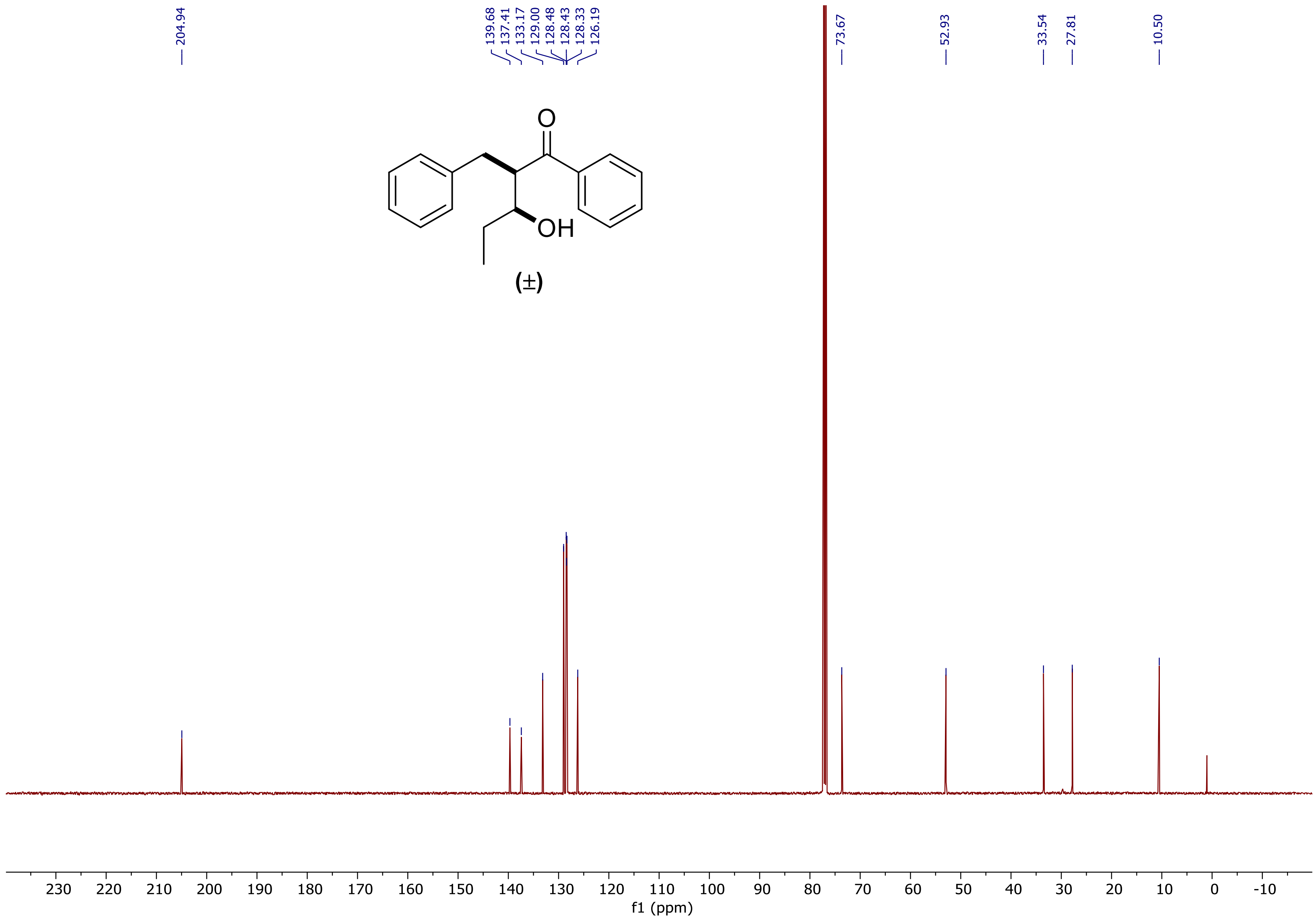

^{13}C NMR (126 MHz, $CDCl_3$) Spectrum of (*2RS,3RS*)-2-benzyl-3-hydroxy-1-phenyl-1-pentanone **3b**.

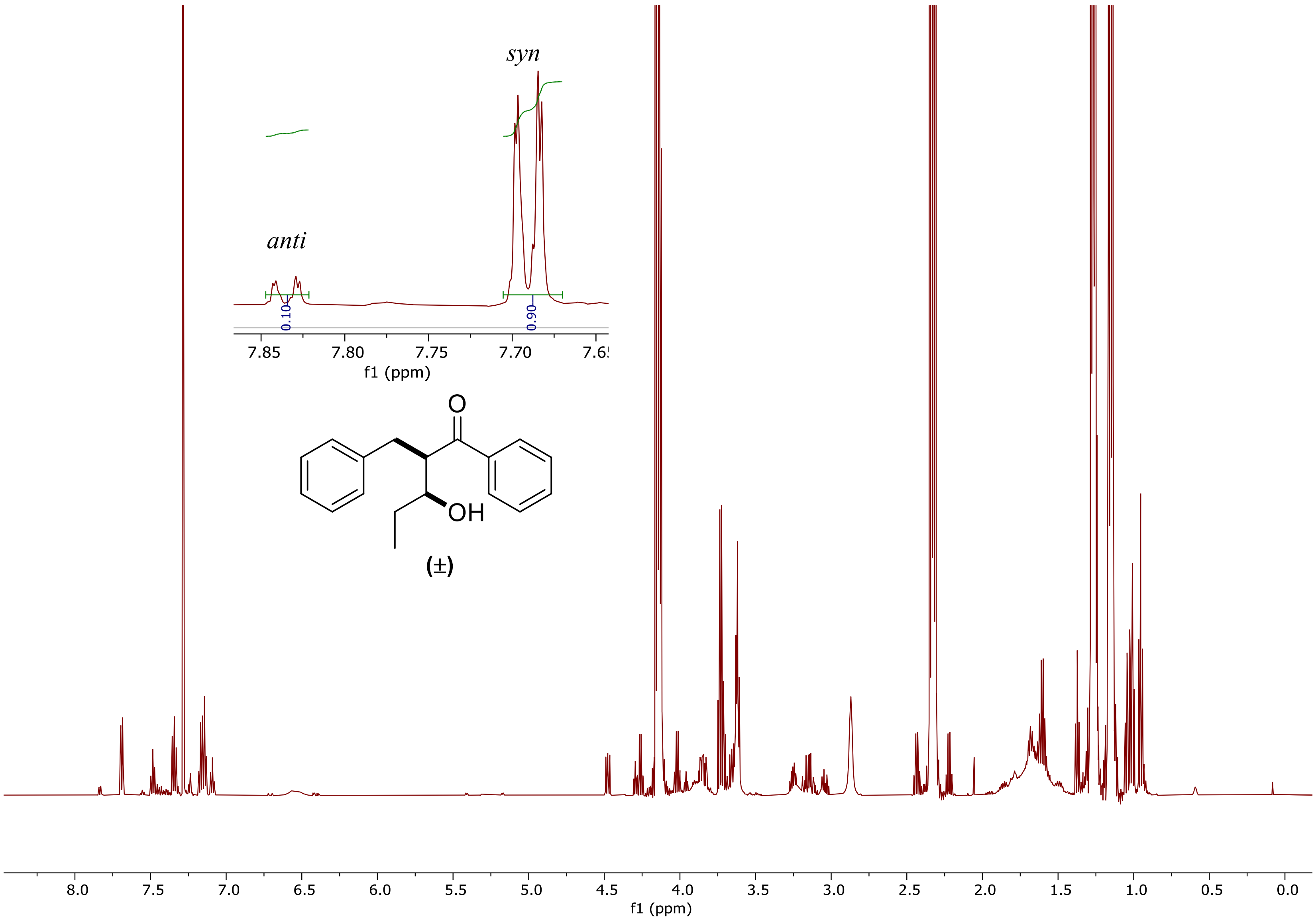

^{1}H NMR (400 MHz, $CDCl_3$) Spectrum of the crude reaction mixture for (*2RS,3RS*)-2-benzyl-3-hydroxy-1-phenyl-1-pentanone **3b**.

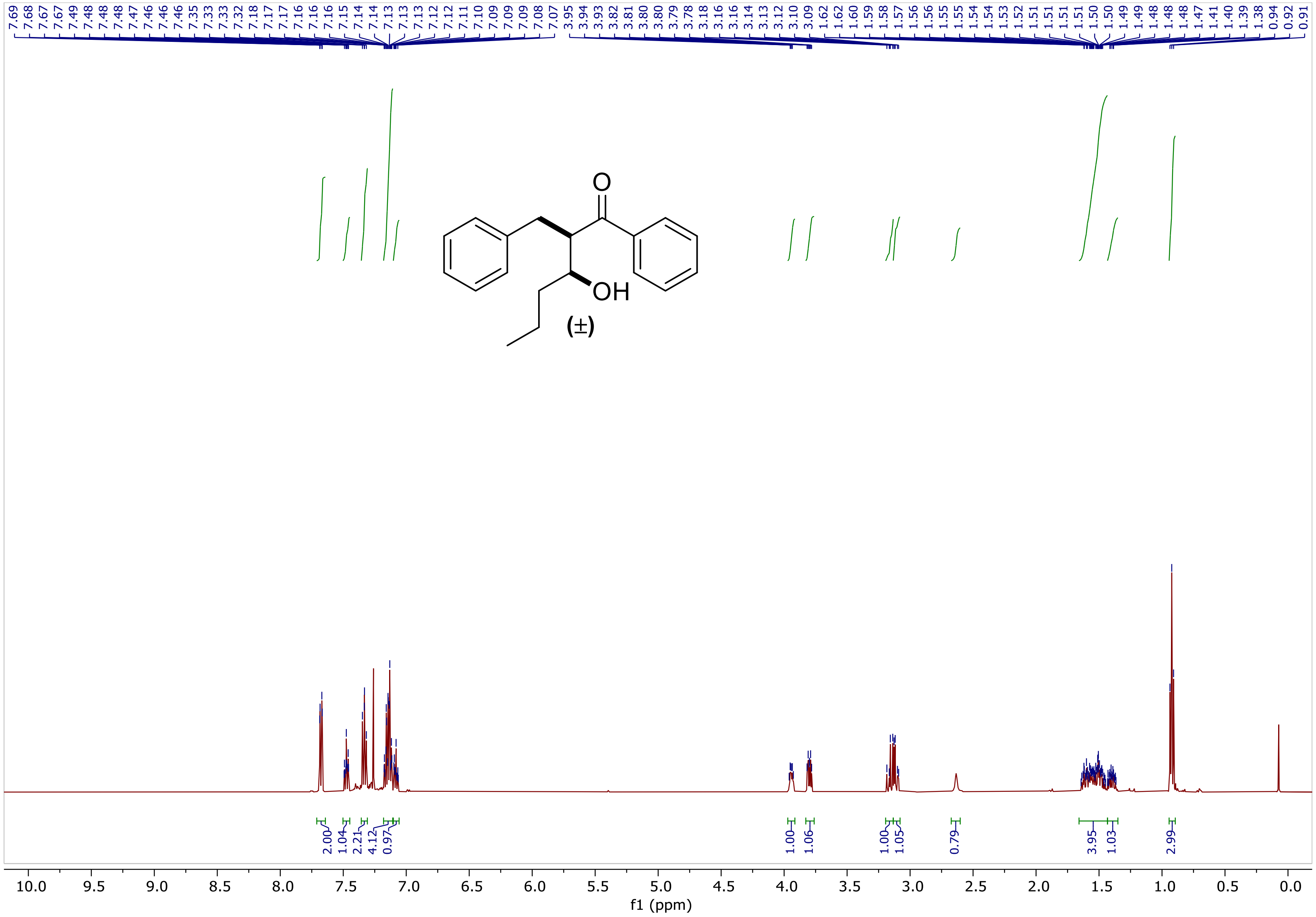

^{1}H NMR (500 MHz, $CDCl_3$) Spectrum of (*2RS,3RS*)-2-benzyl-3-hydroxy-1-phenyl-1-hexanone **3c**.

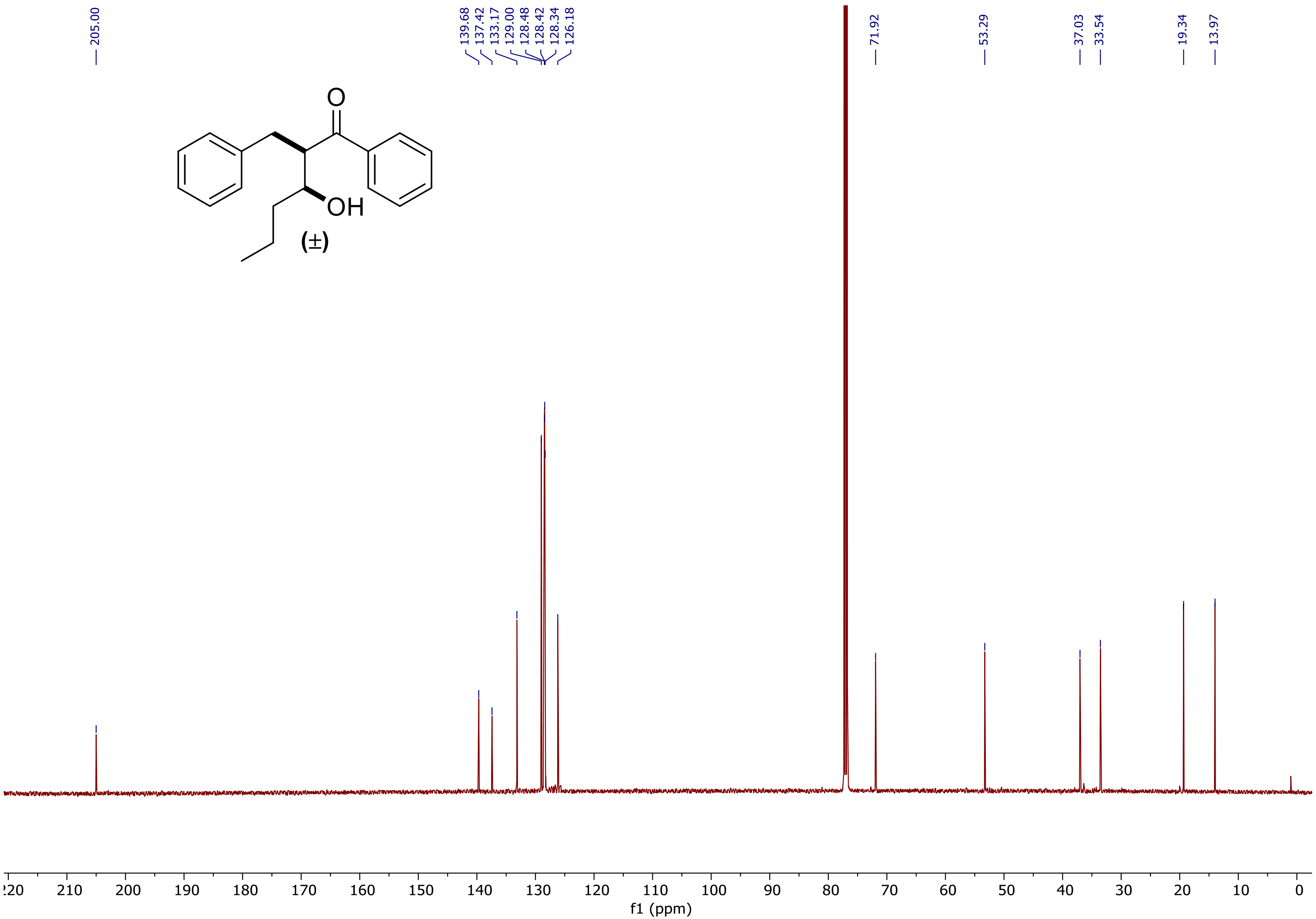

^{13}C NMR (126 MHz, $CDCl_3$) Spectrum of (*2RS,3RS*)-2-benzyl-3-hydroxy-1-phenyl-1-hexanone **3c**.

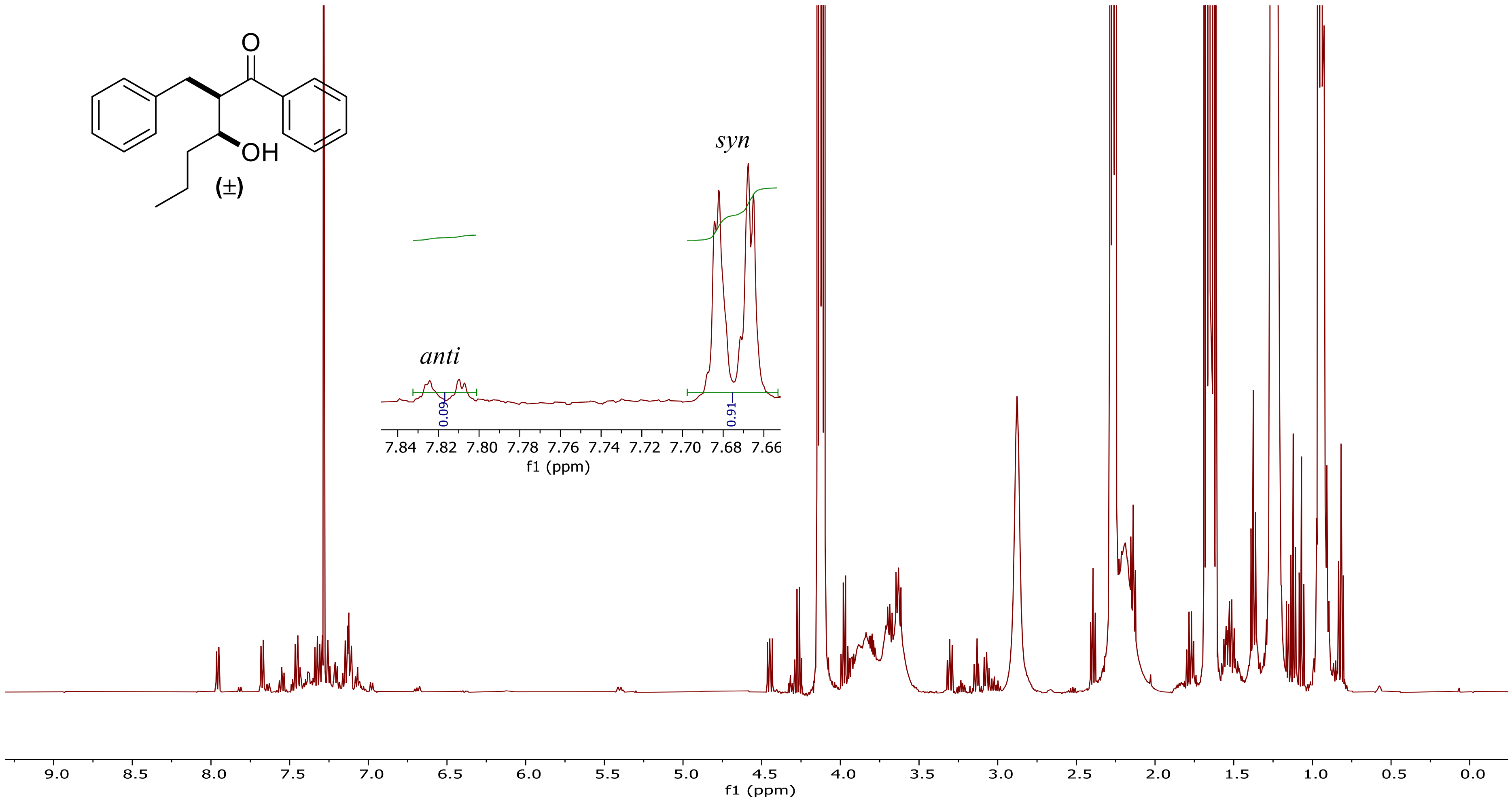

^{1}H NMR (400 MHz, $CDCl_3$) Spectrum of the crude reaction mixture for (*2RS,3RS*)-2-benzyl-3-hydroxy-1-phenyl-1-hexanone **3c**.

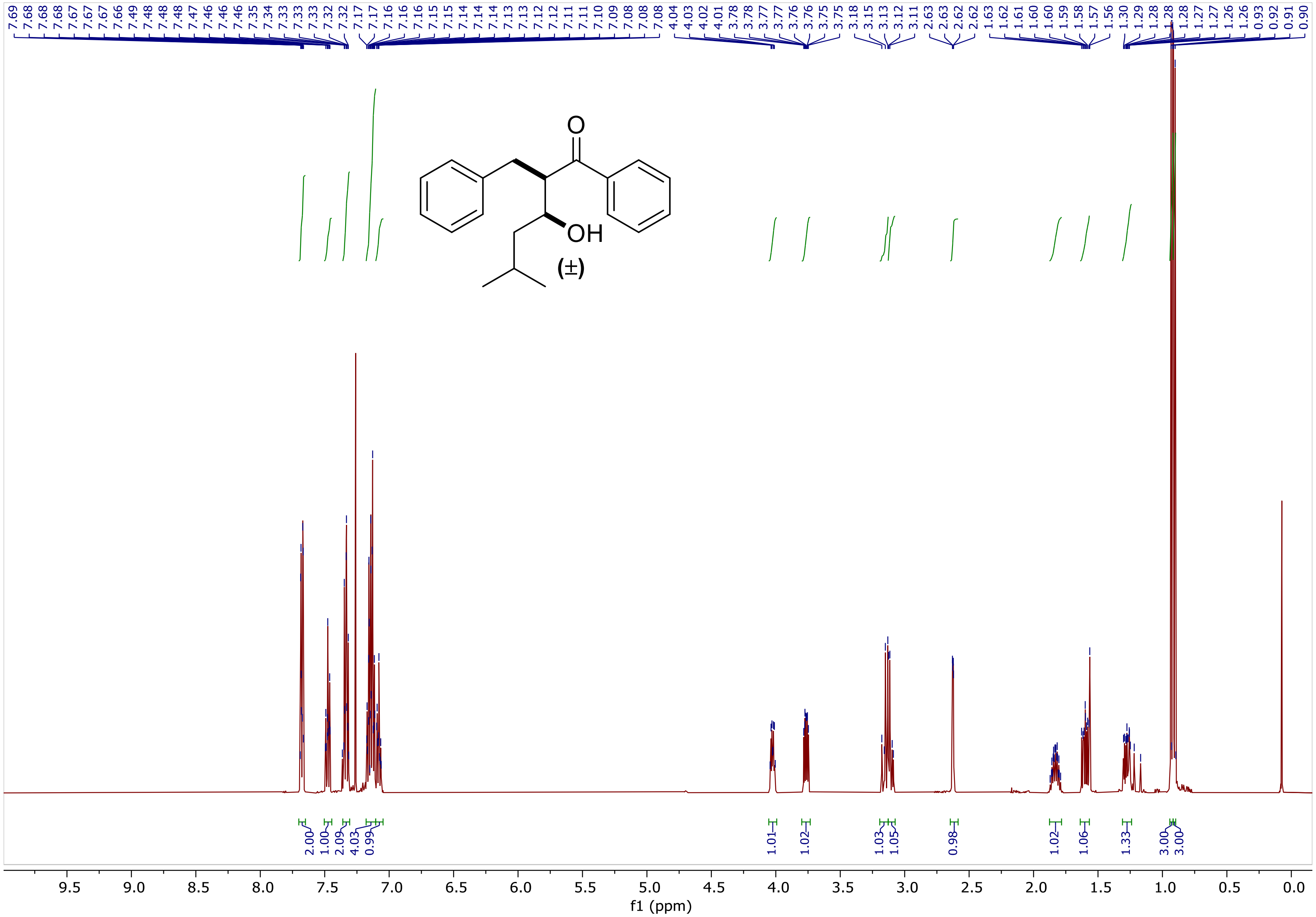

^{1}H NMR (500 MHz, $CDCl_3$) Spectrum of (*2RS,3RS*)-2-benzyl-3-hydroxy-5-methyl-1-phenyl-1-hexanone **3d**.

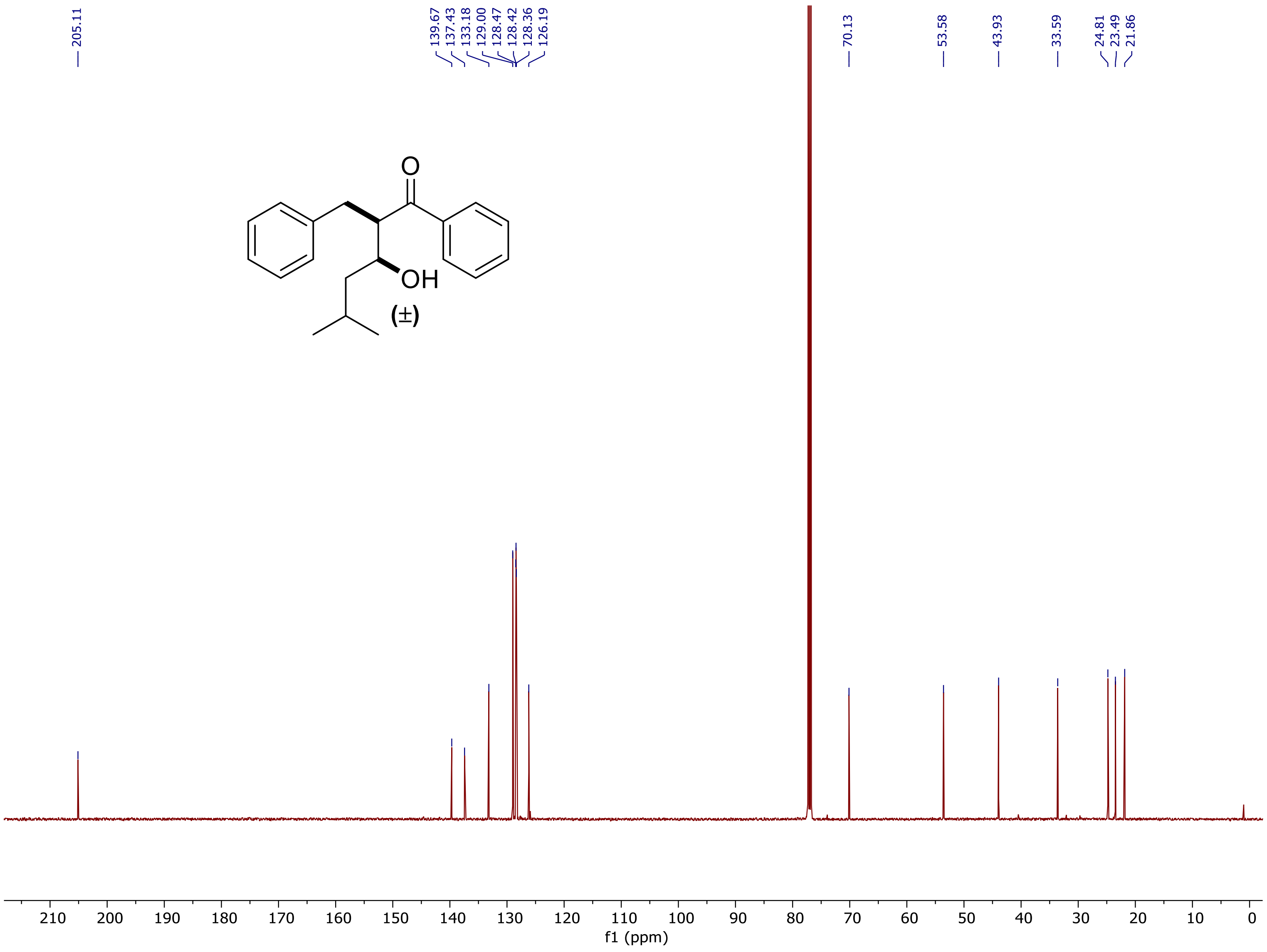

^{13}C **NMR** (126 MHz, $CDCl_3$) Spectrum of (*2RS,3RS*)-2-benzyl-3-hydroxy-5-methyl-1-phenyl-1-hexanone **3d**.

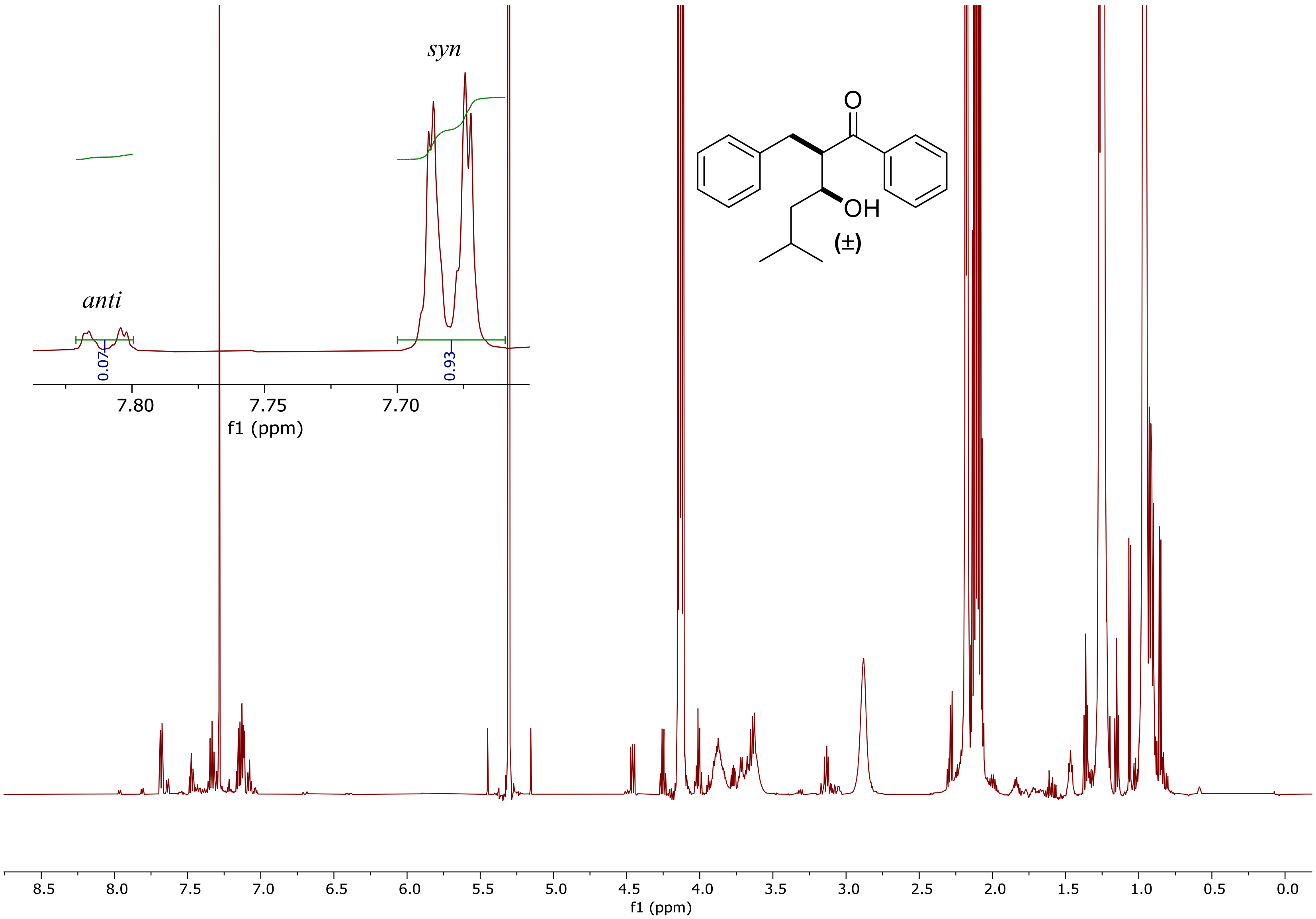

^{1}H NMR (400 MHz, $CDCl_3$) Spectrum of the crude reaction mixture for (*2RS,3RS*)-2-benzyl-3-hydroxy-5-methyl-1-phenyl-1-hexanone **3d**.

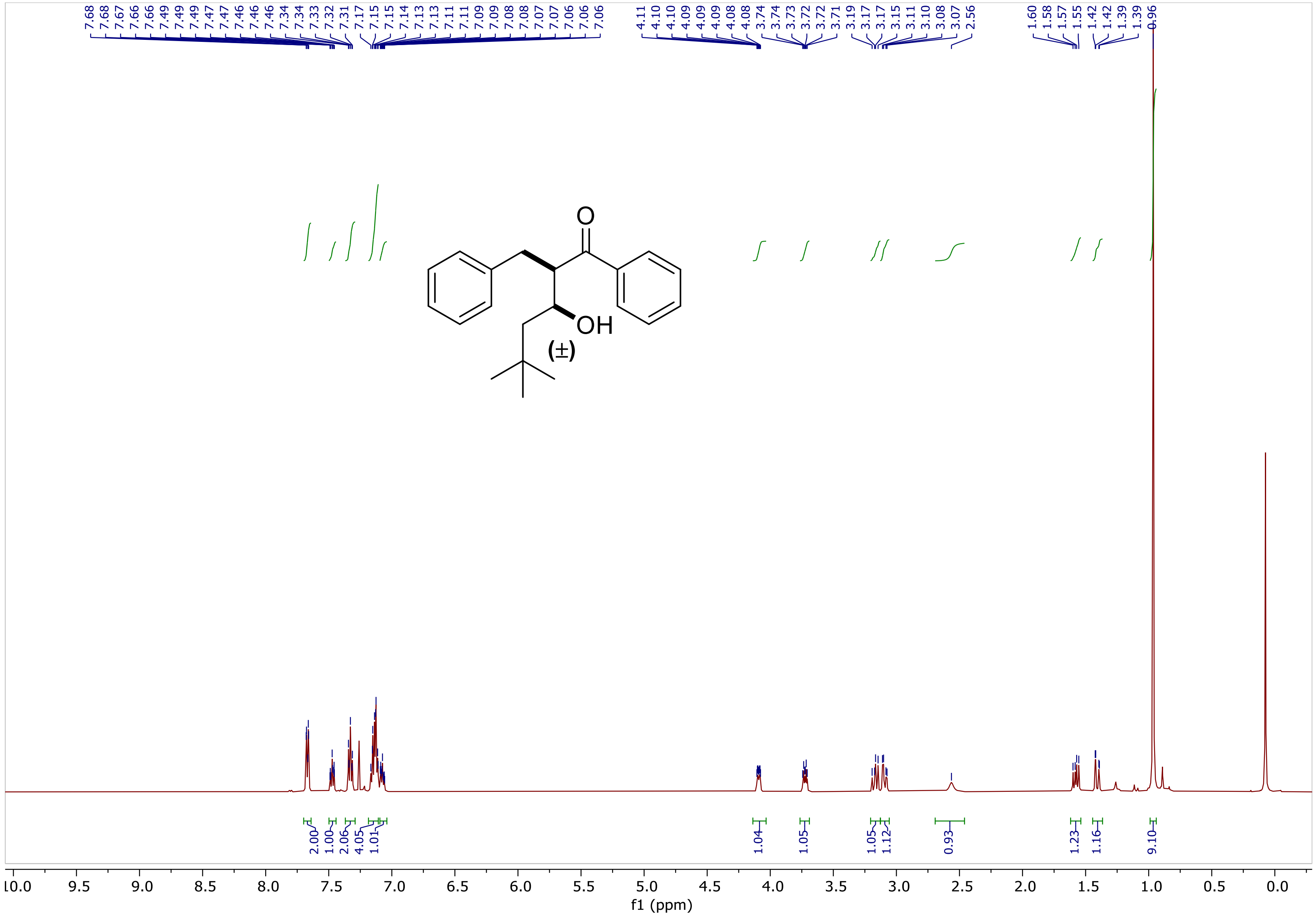

^{1}H NMR (500 MHz, $CDCl_3$) Spectrum of (*2RS,3RS*)-2-benzyl-3-hydroxy-5,5-dimethyl-1-phenyl-1-hexanone **3e**.

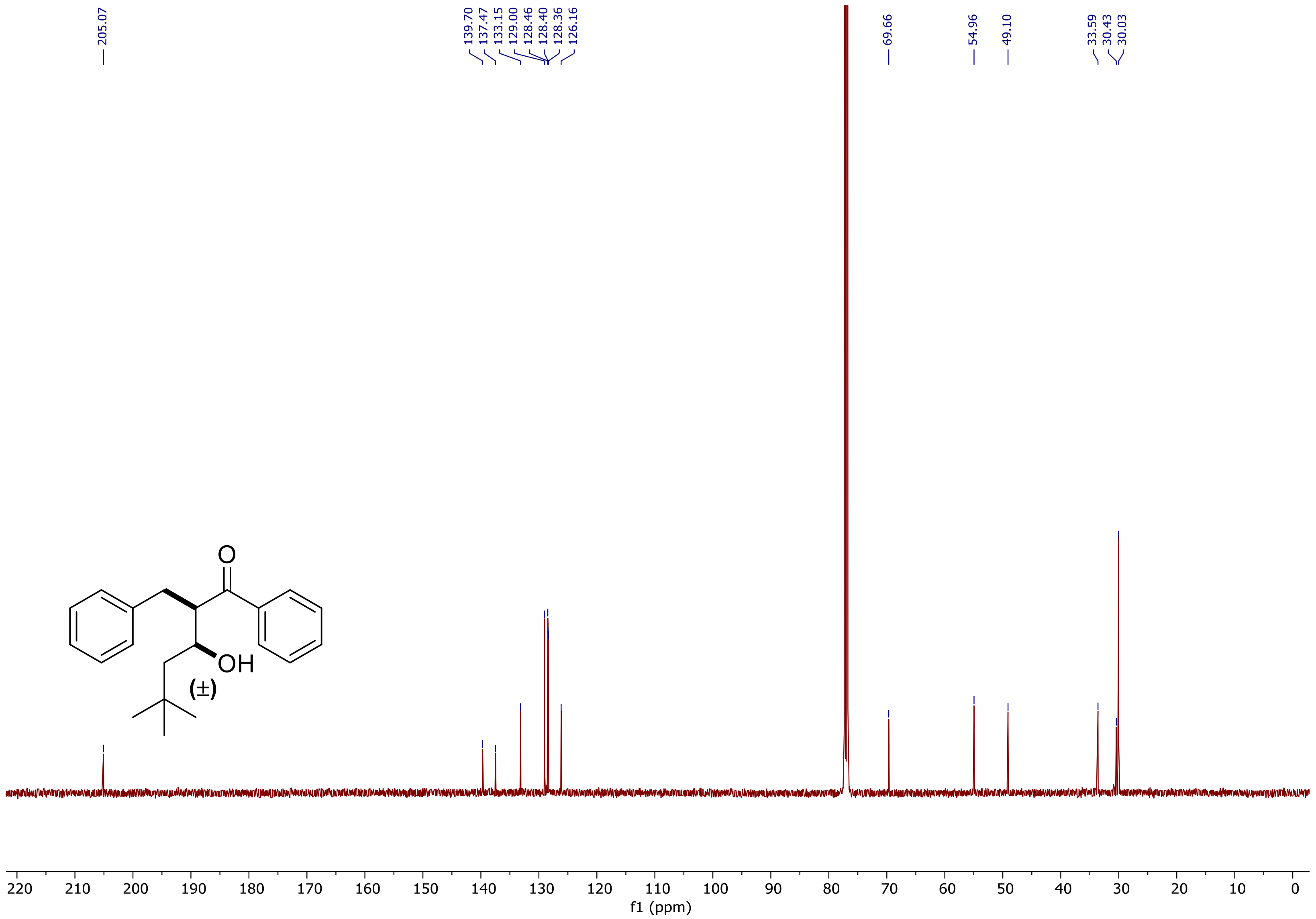

^{13}C NMR (126 MHz, $CDCl_3$) Spectrum of (*2RS,3RS*)-2-benzyl-3-hydroxy-5,5-dimethyl-1-phenyl-1-hexanone **3e**.

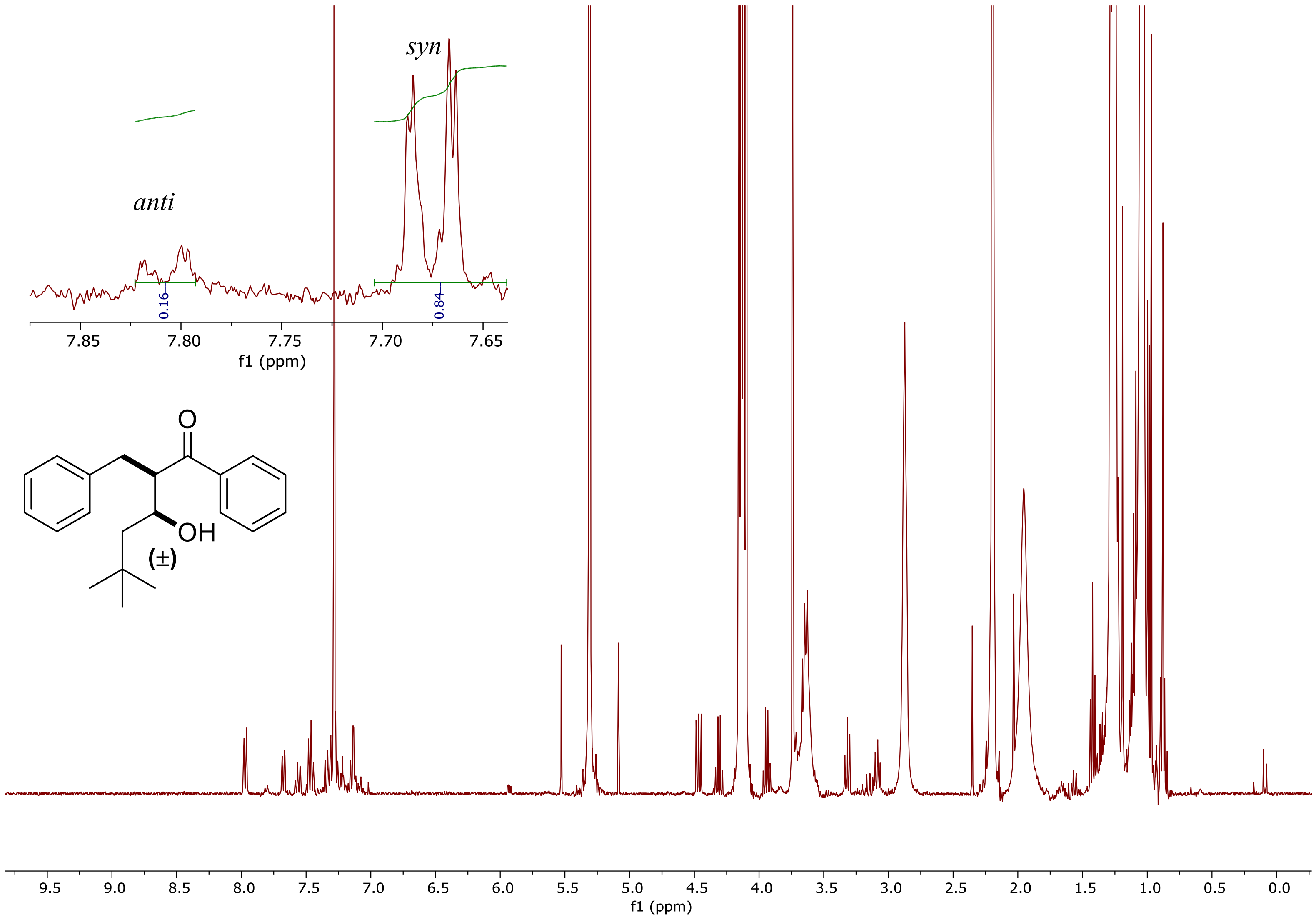

^{1}H NMR (400 MHz, $CDCl_3$) Spectrum of the crude reaction mixture for (*2RS,3RS*)-2-benzyl-3-hydroxy-5,5-dimethyl-1-phenyl-1-hexanone **3e**.

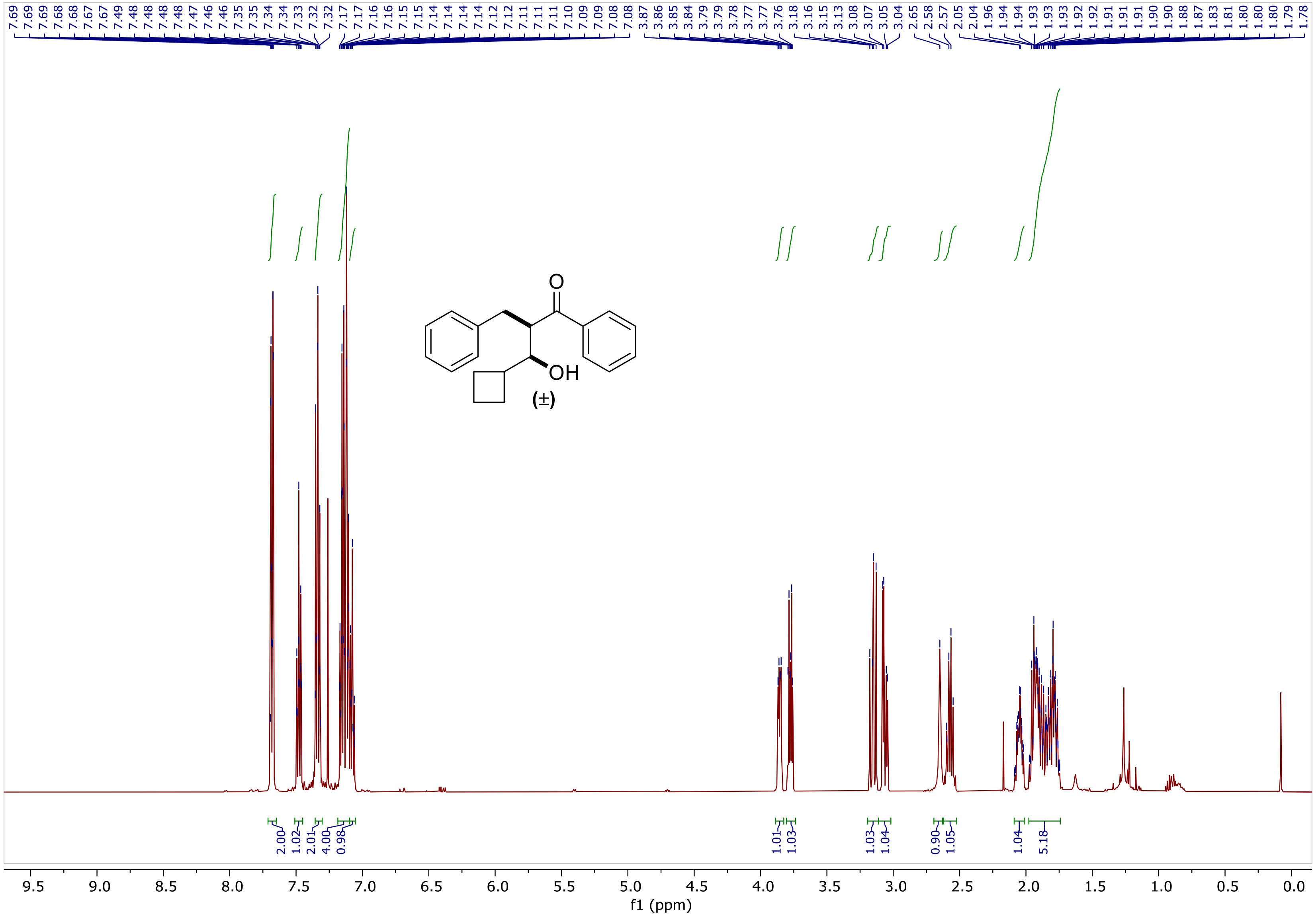

^{1}H NMR (500 MHz, $CDCl_3$) Spectrum of (*2RS,3RS*)-2-benzyl-3-cyclobutyl-3-hydroxy-1-phenyl-1-propanone **3f**.

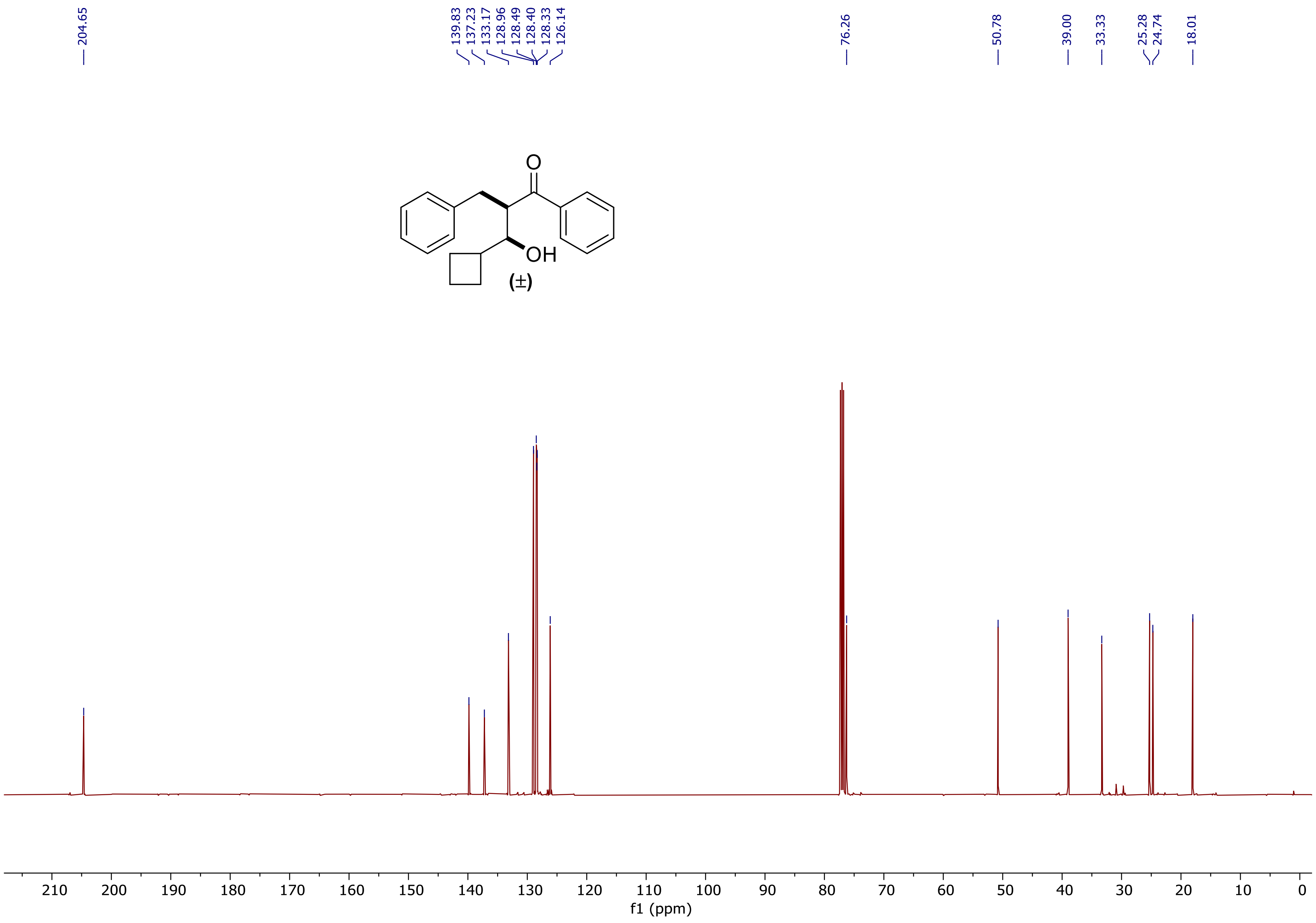

^{13}C NMR (126 MHz, $CDCl_3$) Spectrum of (*2RS,3RS*)-2-benzyl-3-cyclobutyl-3-hydroxy-1-phenyl-1-propanone **3f**.

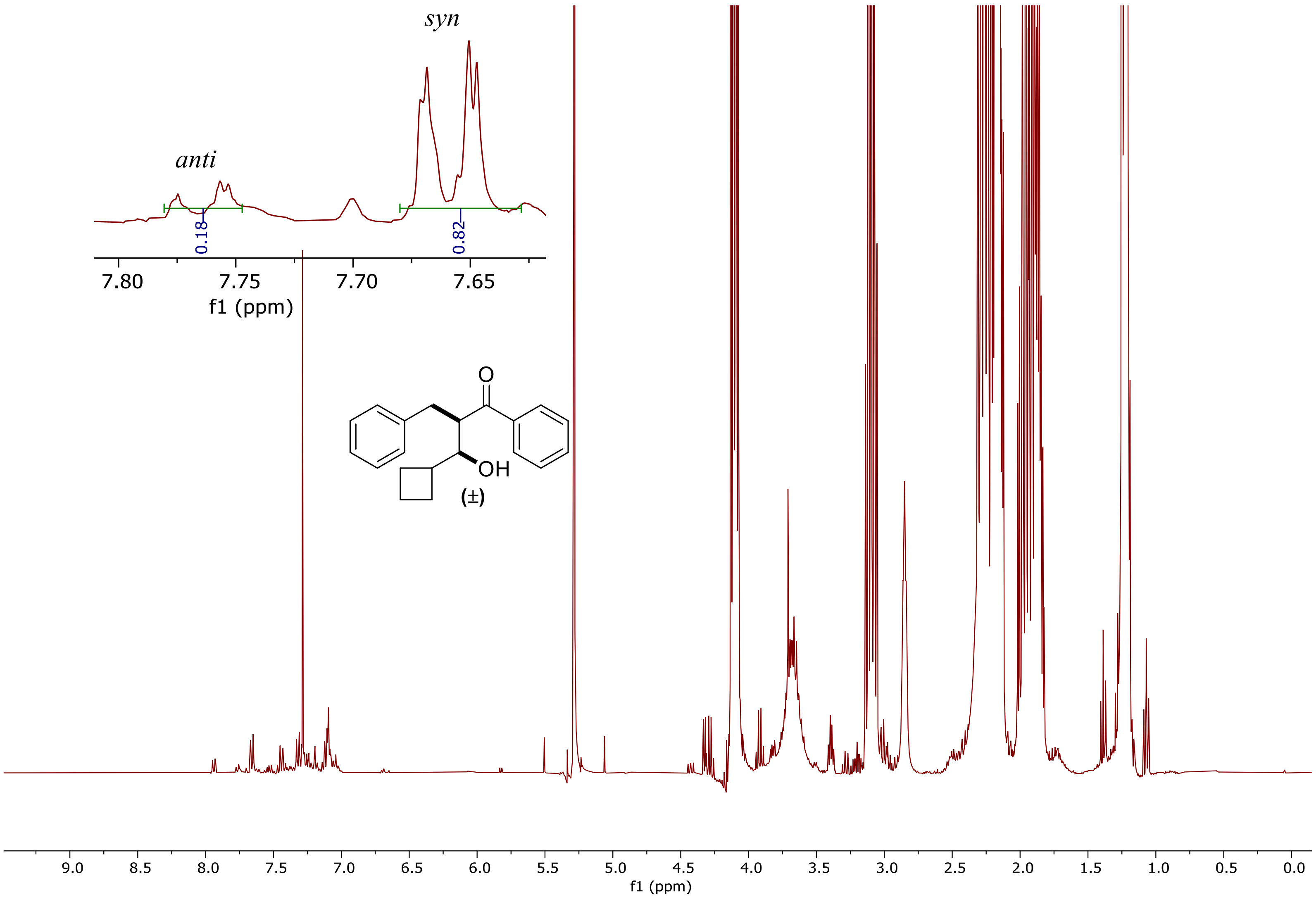

^{1}H NMR (400 MHz, $CDCl_3$) Spectrum of the crude reaction mixture for (*2RS,3RS*)-2-benzyl-3-cyclobutyl-3-hydroxy-1-phenyl-1-propanone **3f**.

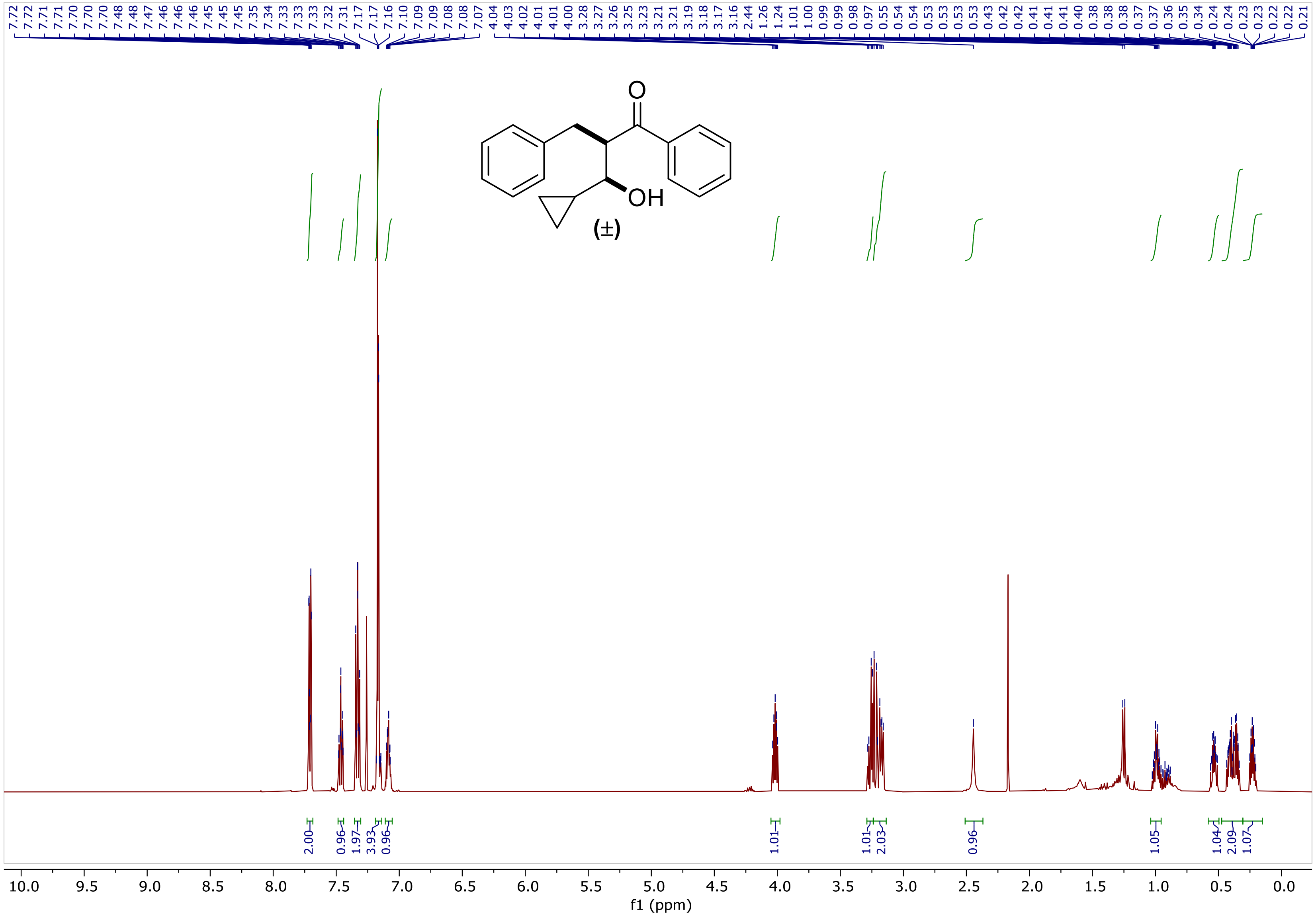

¹H NMR (500 MHz, $CDCl_3$) Spectrum of (*2RS,3RS*)-2-benzyl-3-cyclopropyl-3-hydroxy-1-phenyl-1-propanone **3g**.

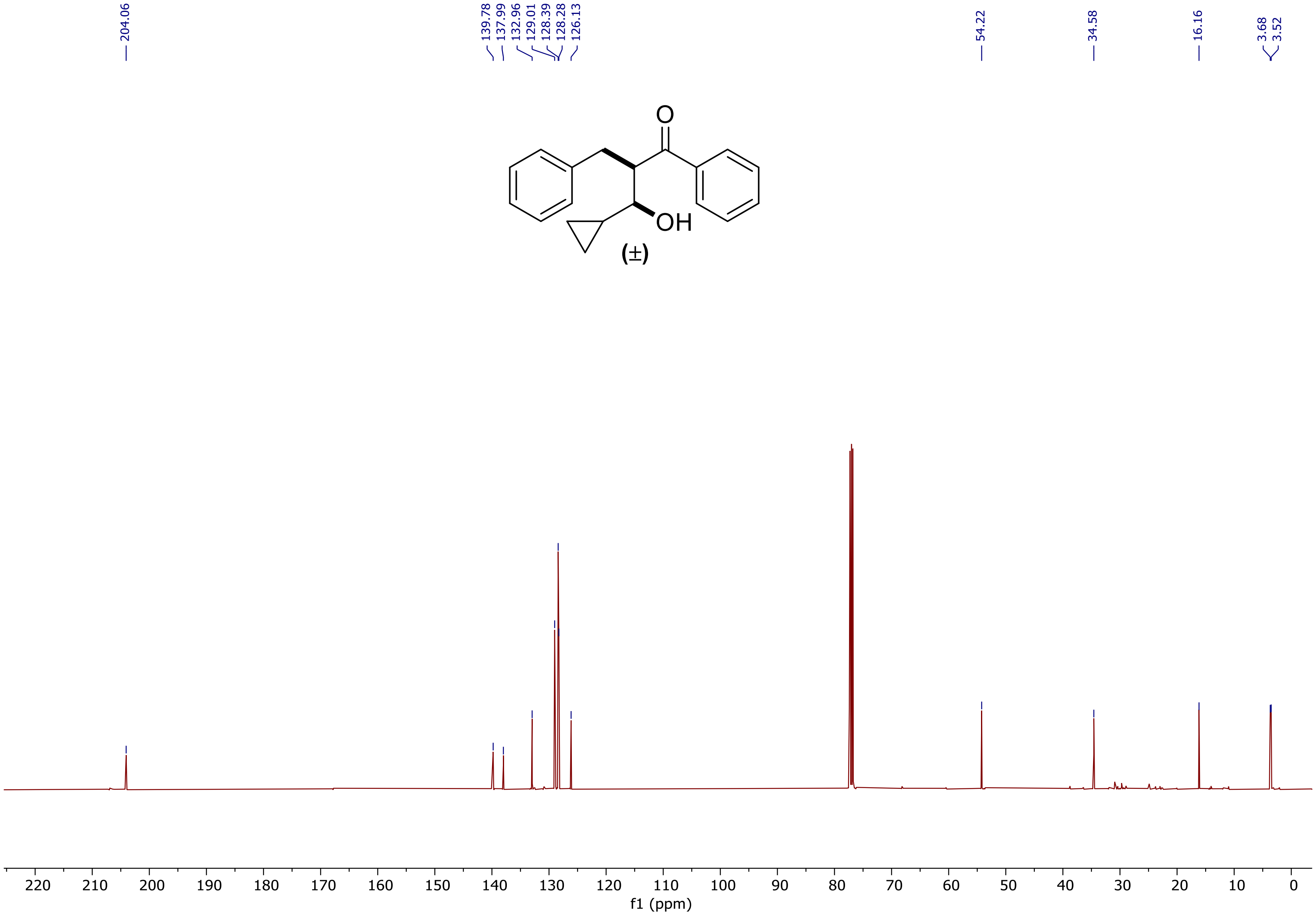

^{13}C NMR (126 MHz, $CDCl_3$) Spectrum of (*2RS,3RS*)-2-benzyl-3-cyclopropyl-3-hydroxy-1-phenyl-1-propanone **3g**.

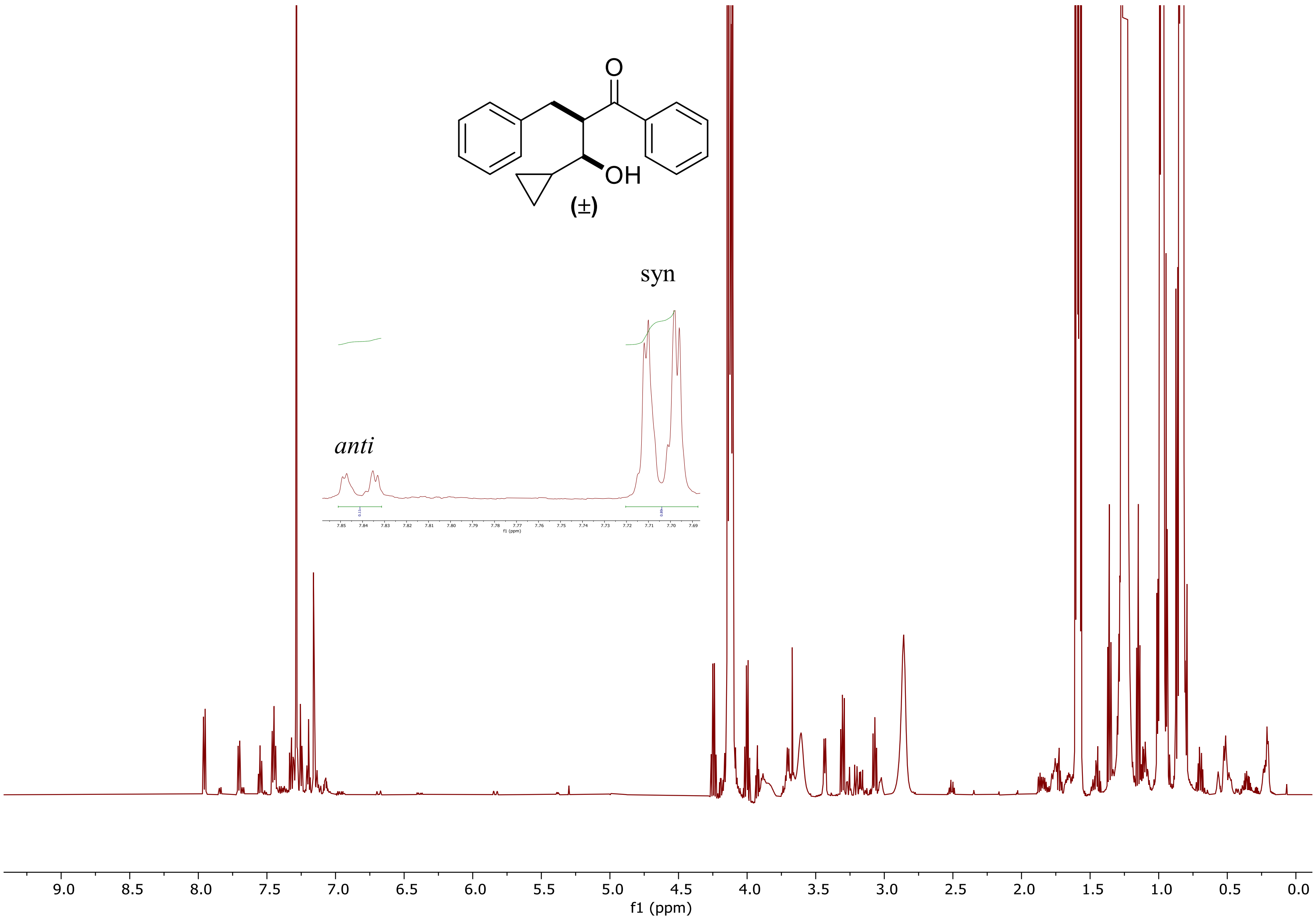

^{1}H NMR (400 MHz, $CDCl_3$) Spectrum of the crude reaction mixture for (*2RS,3RS*)-2-benzyl-3-cyclopropyl-3-hydroxy-1-phenyl-1-propanone **3g**.

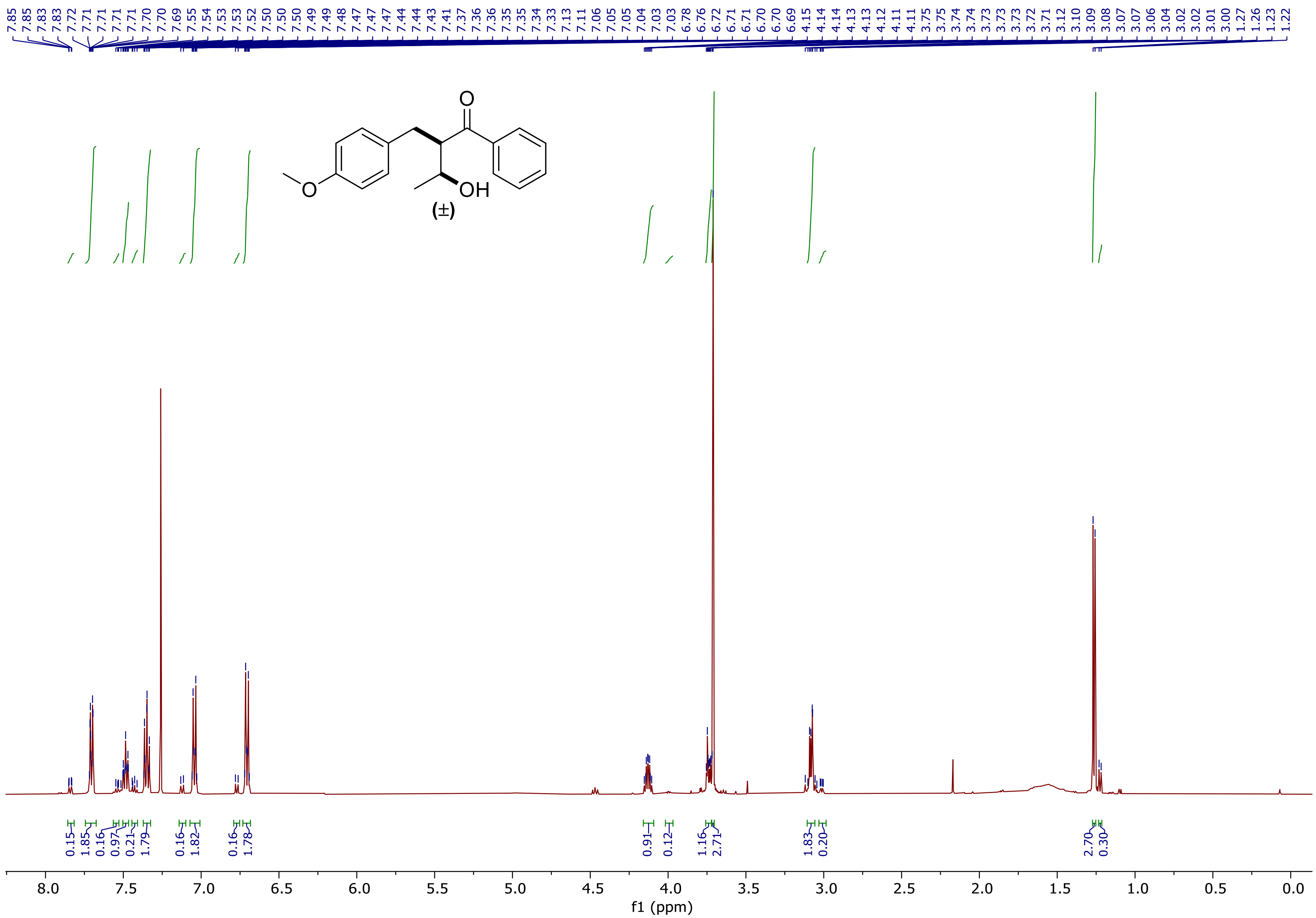

^{1}**H NMR** (500 MHz, CDCl$_3$) Spectrum of (2*RS*,3*RS*)-3-hydroxy-2-(4-methoxybenzyl)-1-phenyl-1-butanone **3h**.

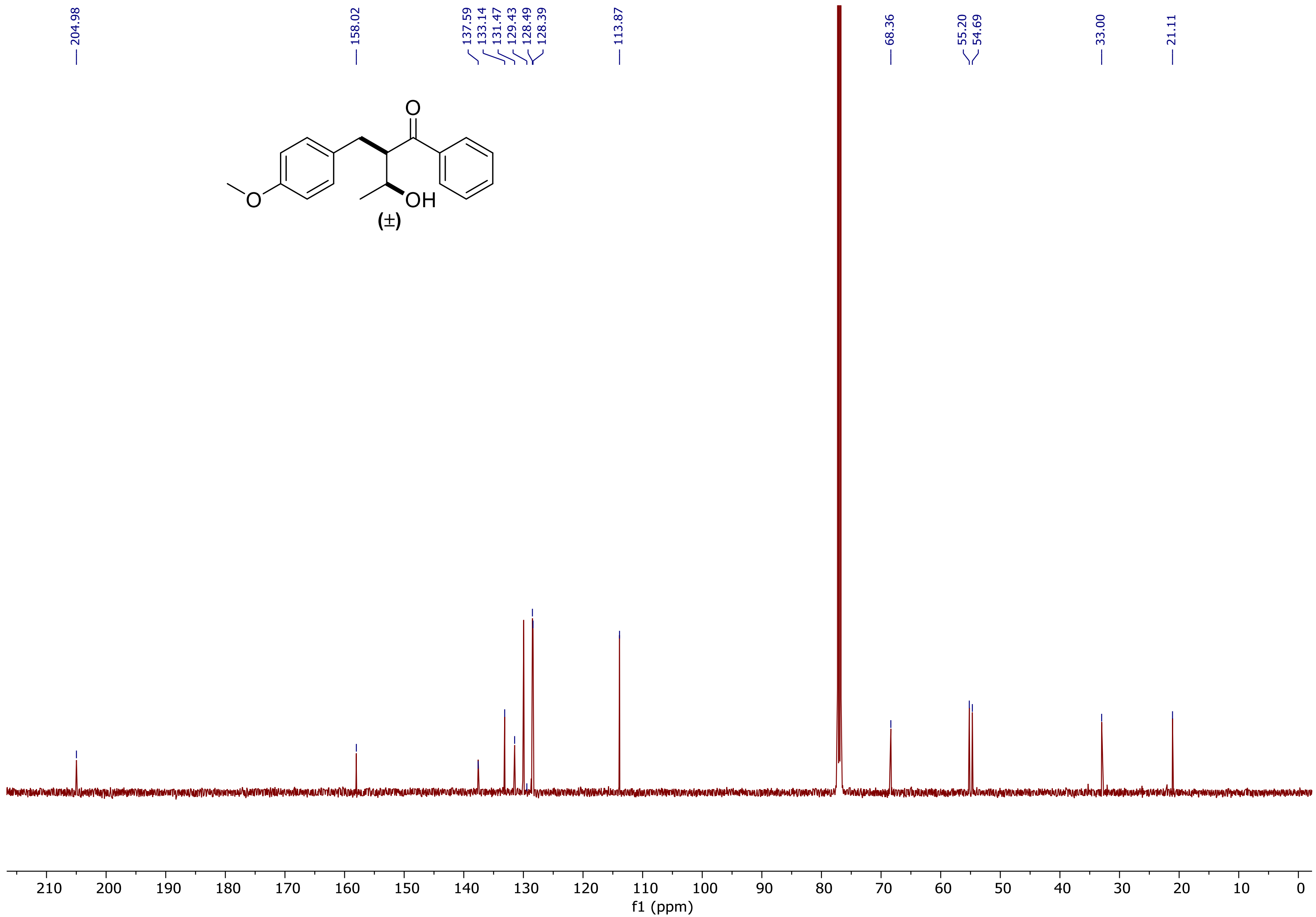

^{13}C NMR (126 MHz, $CDCl_3$) Spectrum of (2*RS*,3*RS*)-3-hydroxy-2-(4-methoxybenzyl)-1-phenyl-1-butanone **3h**.

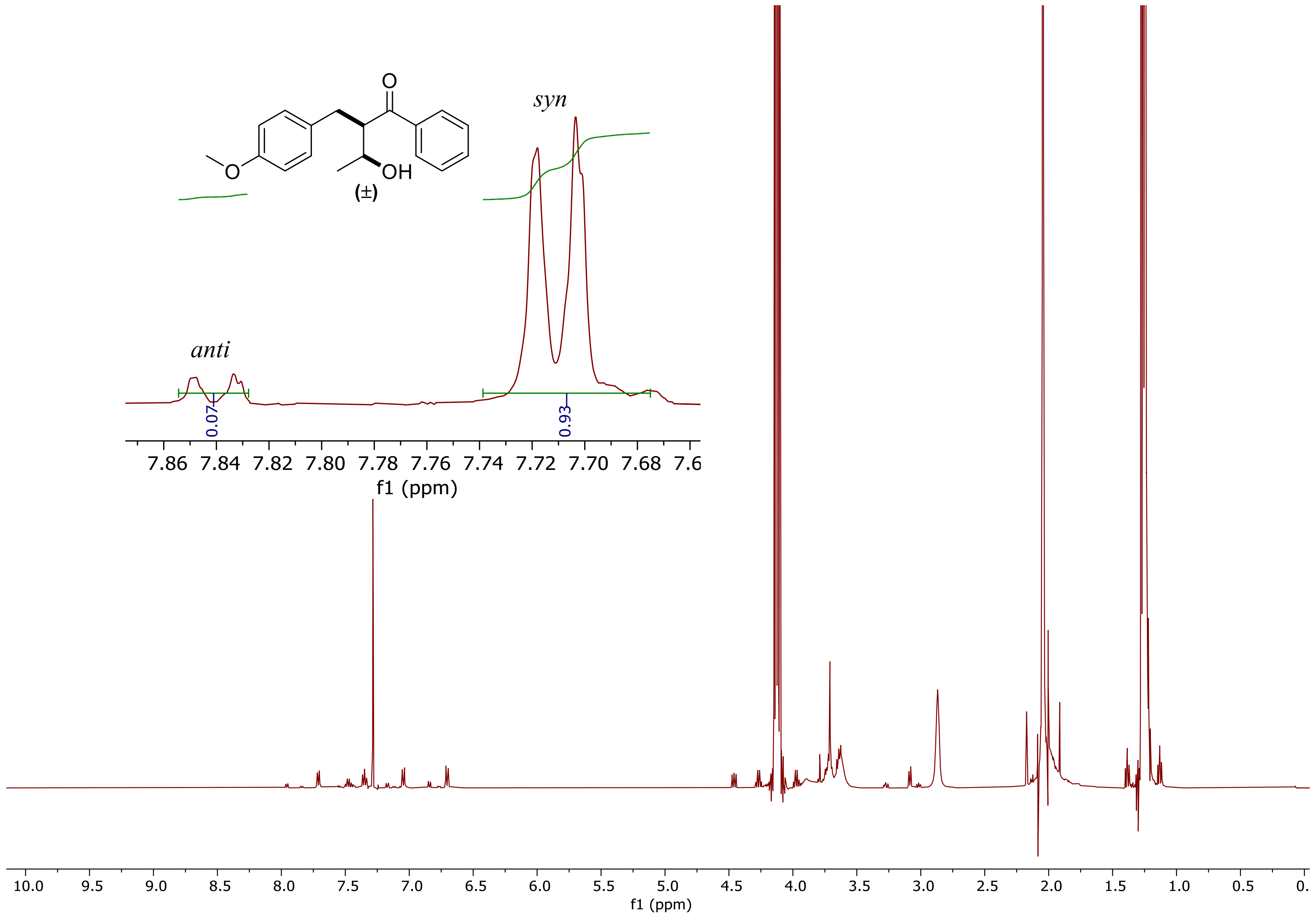

^{1}H NMR (400 MHz, $CDCl_3$) Spectrum of the crude reaction mixture for (2*RS*,3*RS*)-3-hydroxy-2-(4-methoxybenzyl)-1-phenyl-1-butanone **3h**.

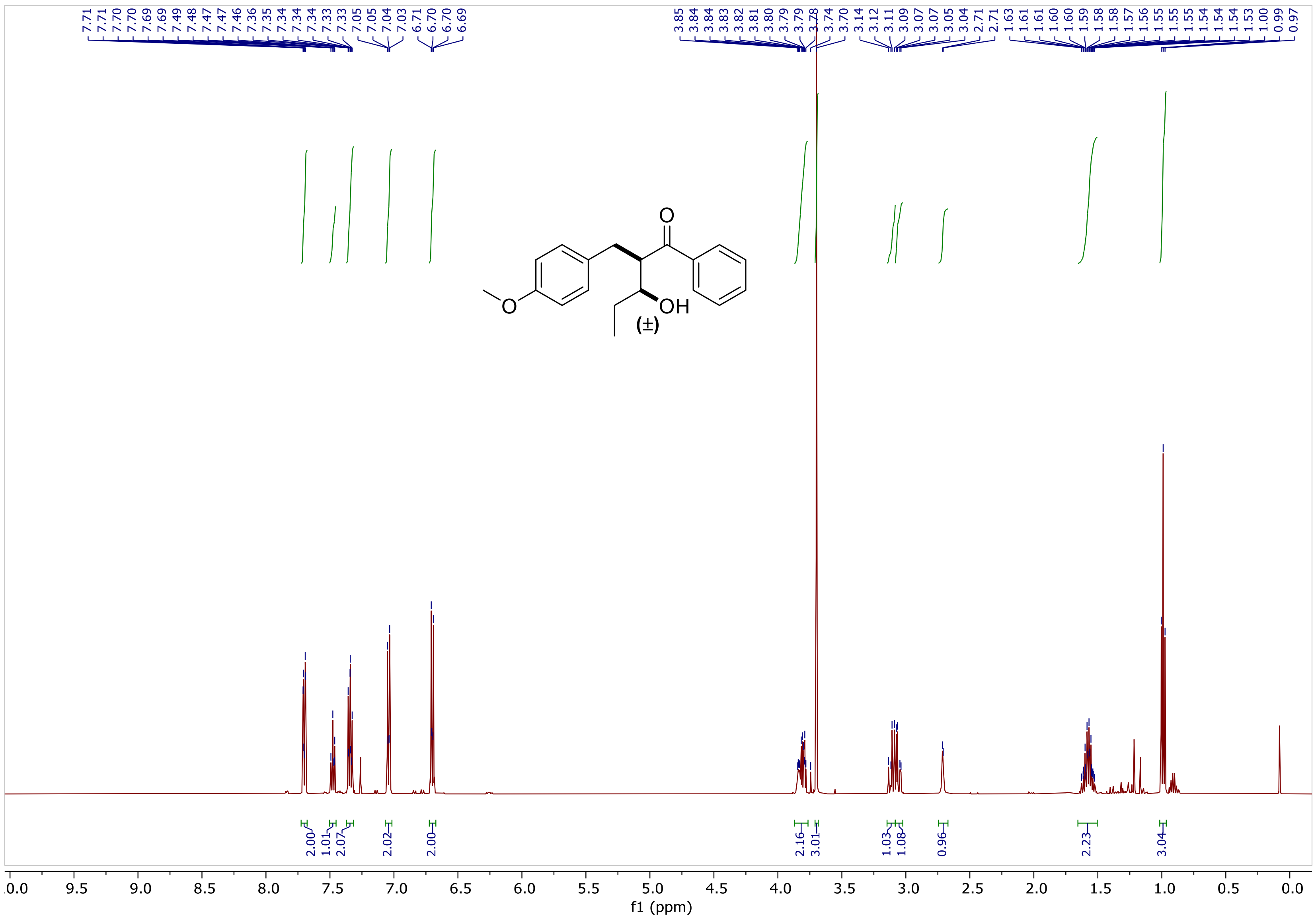

^{1}H NMR (500 MHz, $CDCl_3$) Spectrum of (2*RS*,3*RS*)-3-hydroxy-2-(4-methoxybenzyl)-1-phenyl-1-pentanone **3i**.

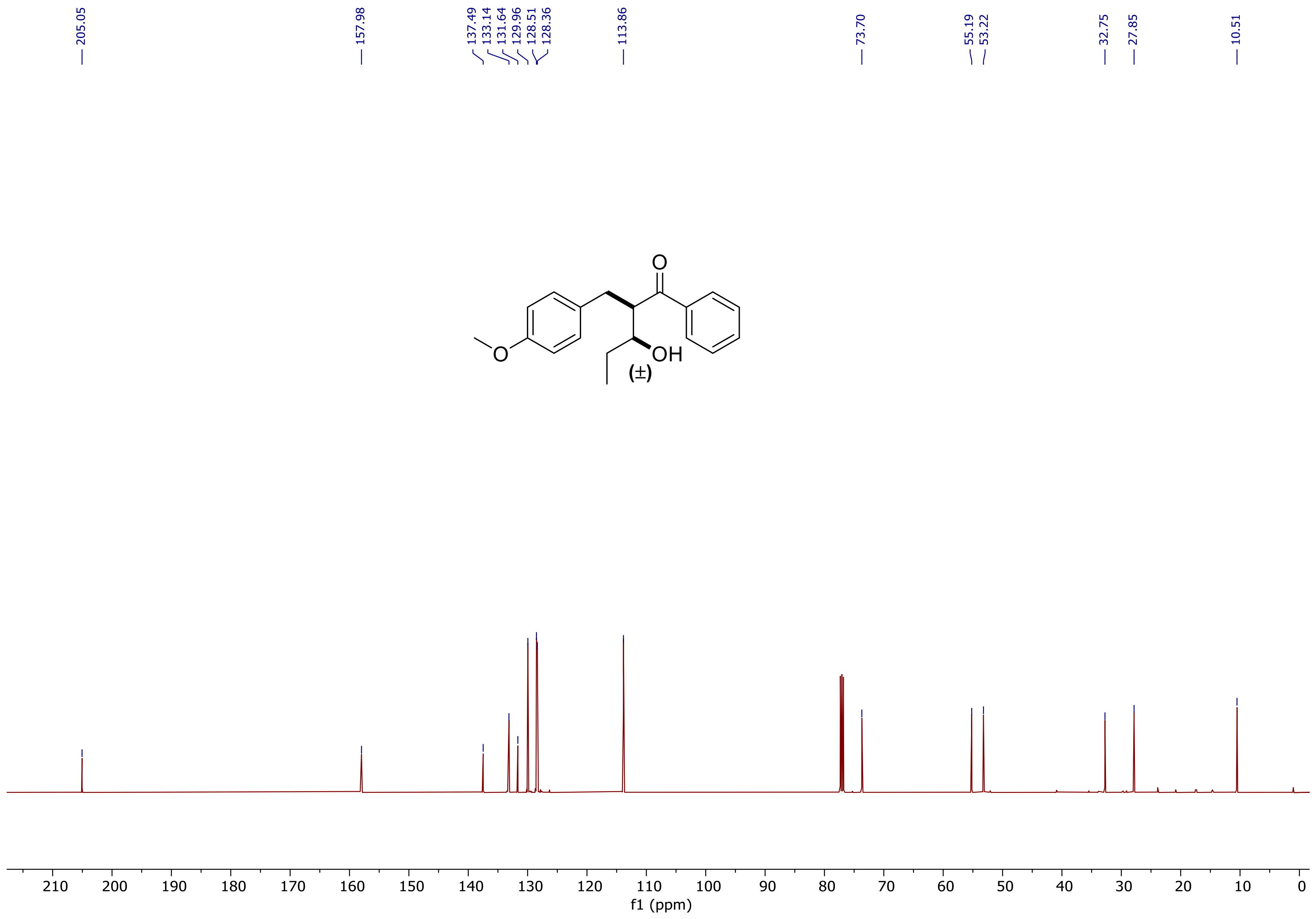

^{13}C NMR (126 MHz, $CDCl_3$) Spectrum of (2*RS*,3*RS*)-3-hydroxy-2-(4-methoxybenzyl)-1-phenyl-1-pentanone **3i**.

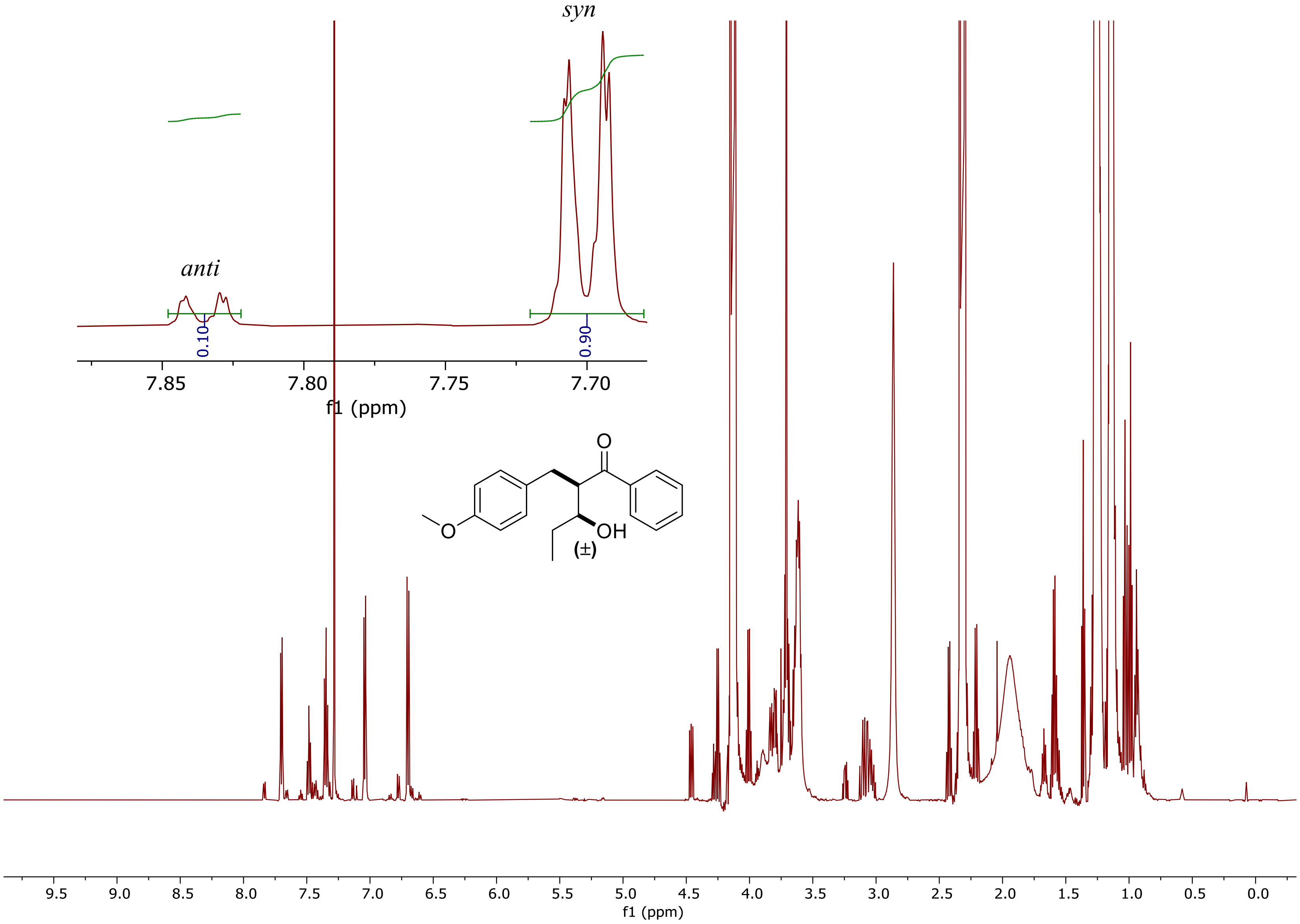

^{1}H NMR (400 MHz, $CDCl_3$) Spectrum of the crude reaction mixture for (2*RS*,3*RS*)-3-hydroxy-2-(4-methoxybenzyl)-1-phenyl-1-pentanone **3i**.

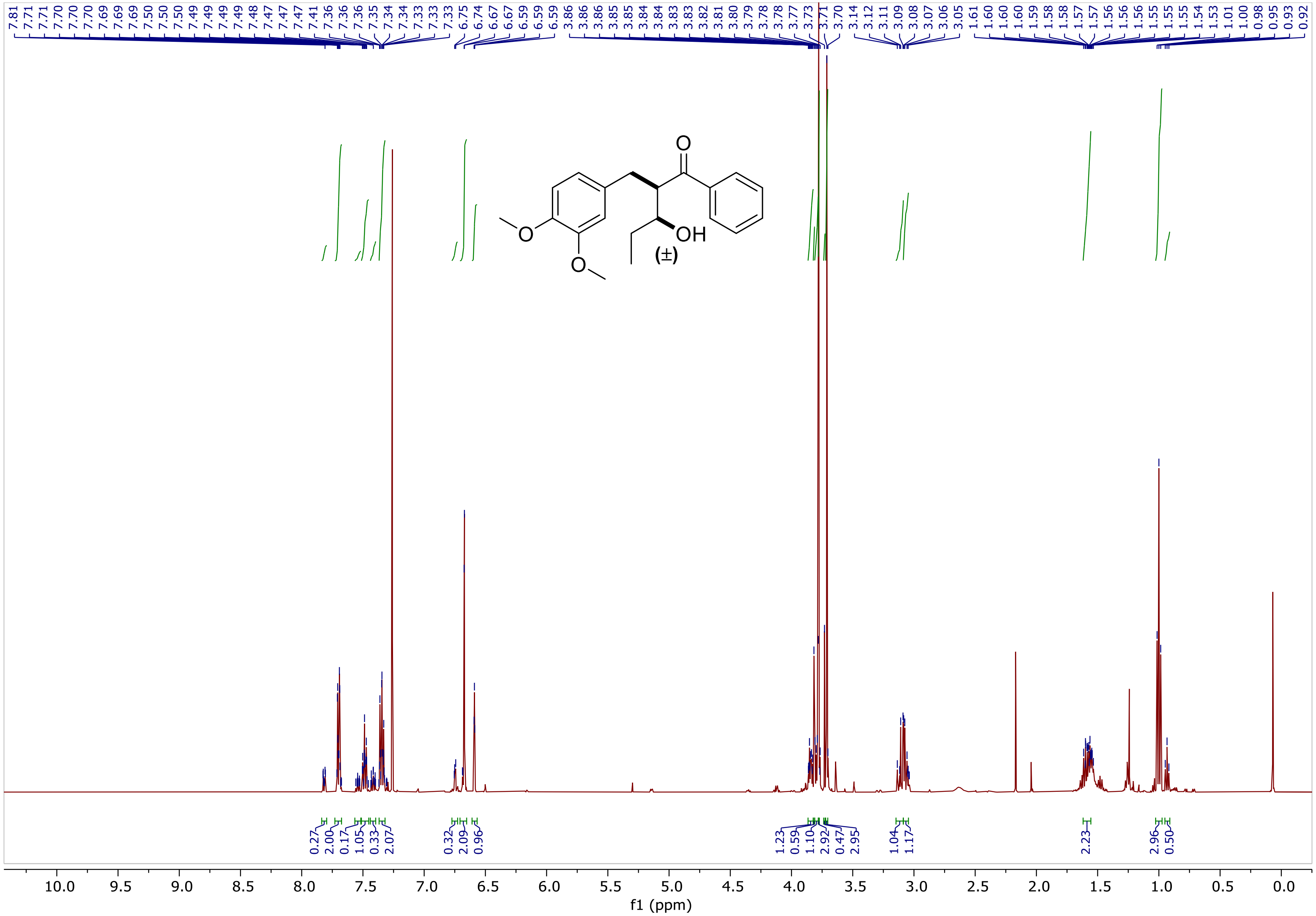

^{1}H NMR (500 MHz, $CDCl_3$) Spectrum of (2*RS*,3*RS*)-3-hydroxy-2-(3,4-dimethoxybenzyl)-1-phenyl-1-pentanone **3j**.

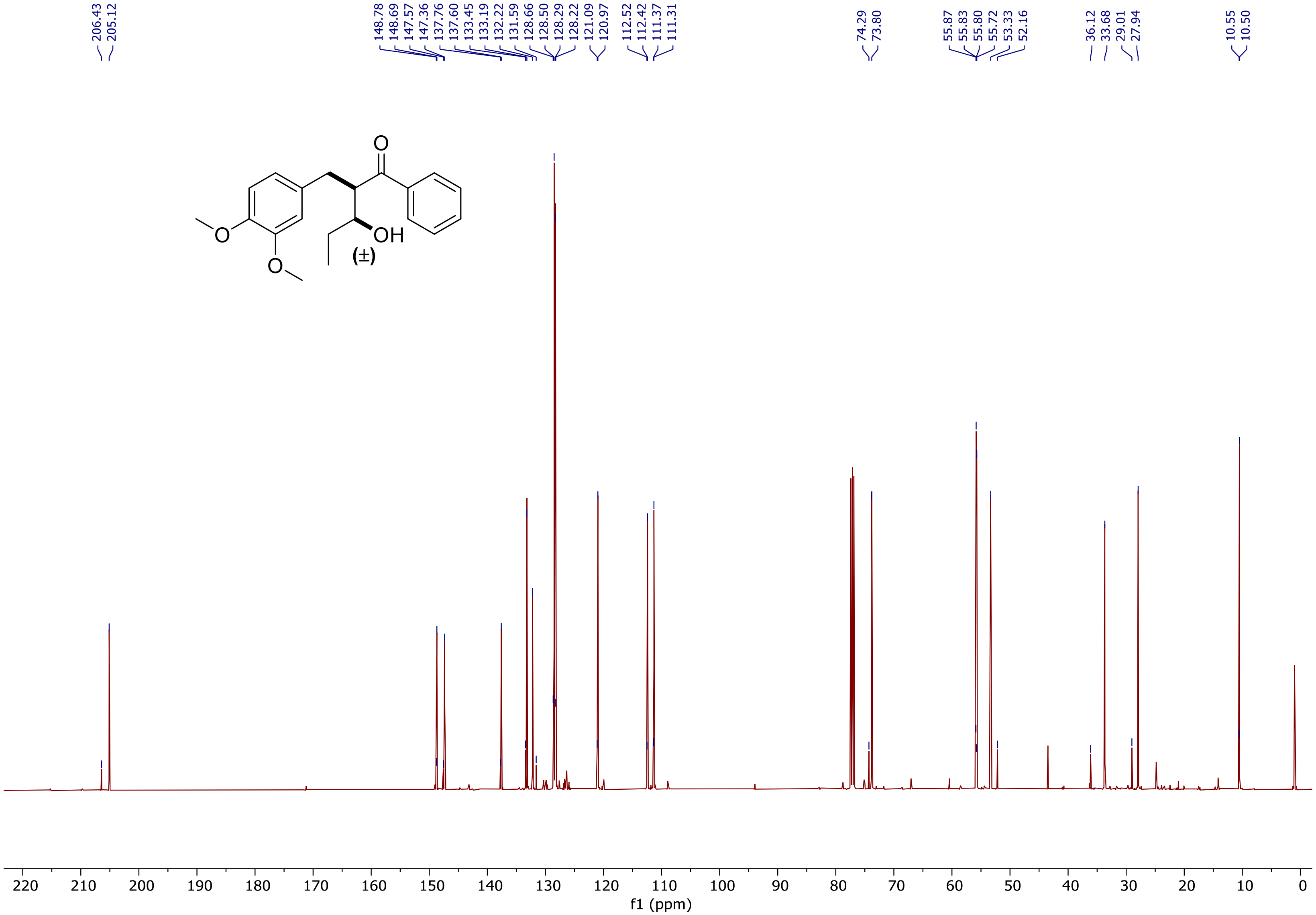

^{13}C NMR (126 MHz, $CDCl_3$) Spectrum of (2*RS*,3*RS*)-3-hydroxy-2-(3,4-dimethoxybenzyl)-1-phenyl-1-pentanone **3j**.

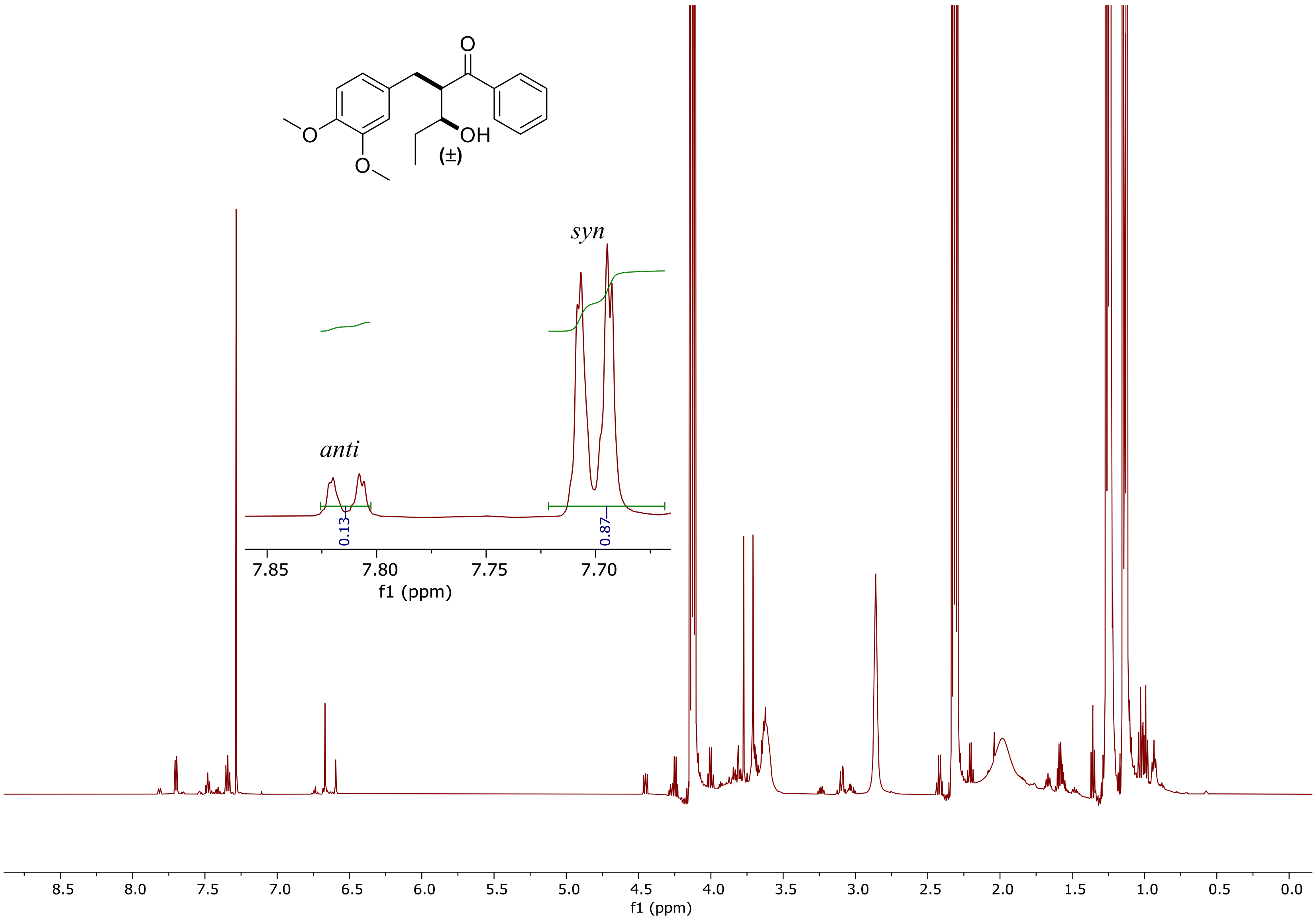

^{1}H NMR (400 MHz, $CDCl_3$) Spectrum of the crude reaction mixture for (2*RS*,3*RS*)-3-hydroxy-2-(3,4-dimethoxybenzyl)-1-phenyl-1-pentanone **3j**.

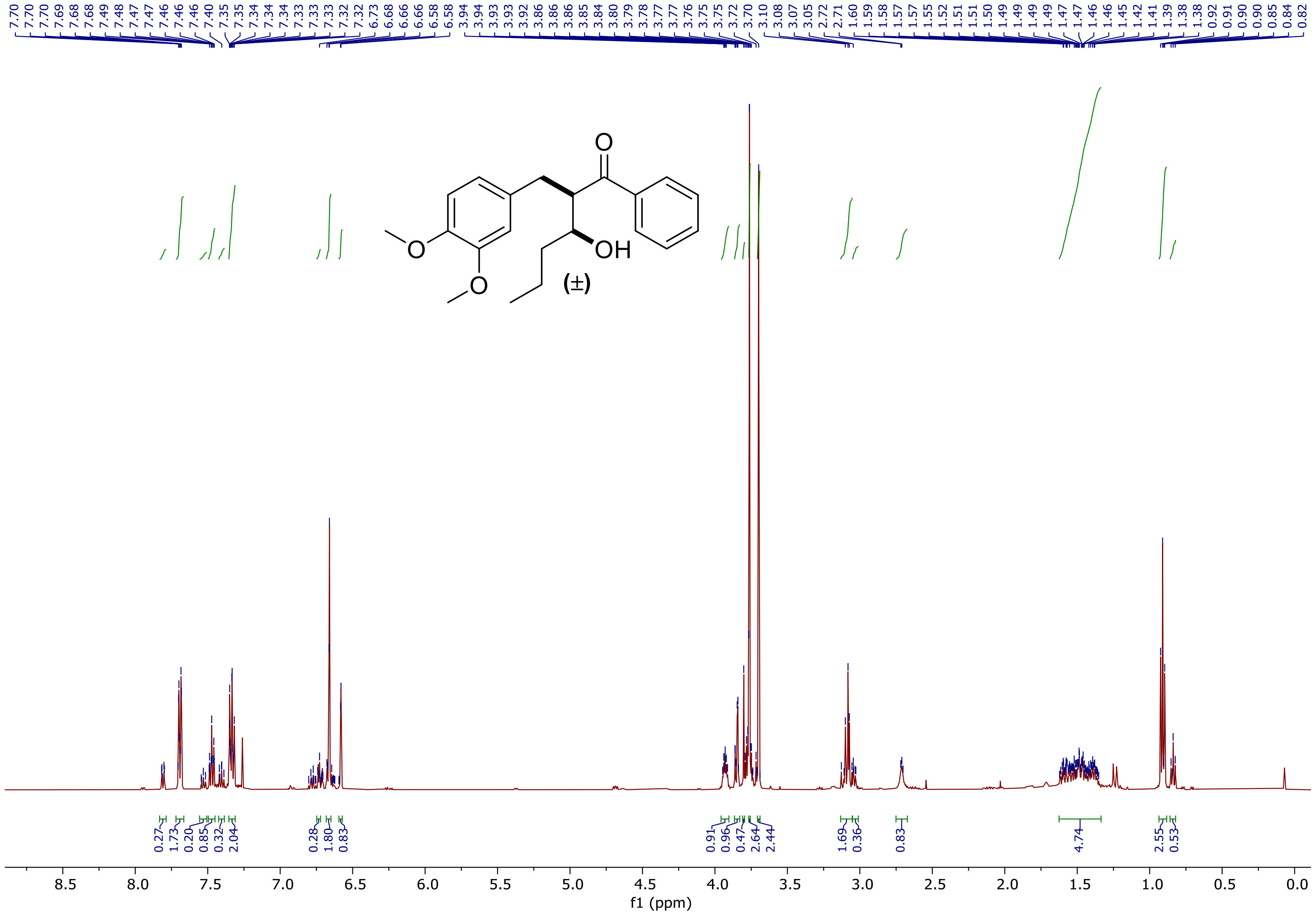

^{1}H NMR (500 MHz, $CDCl_3$) Spectrum of (2*RS*,3*RS*)-3-hydroxy-2-(3,4-dimethoxybenzyl)-1-phenyl-1-hexanone **3k**.

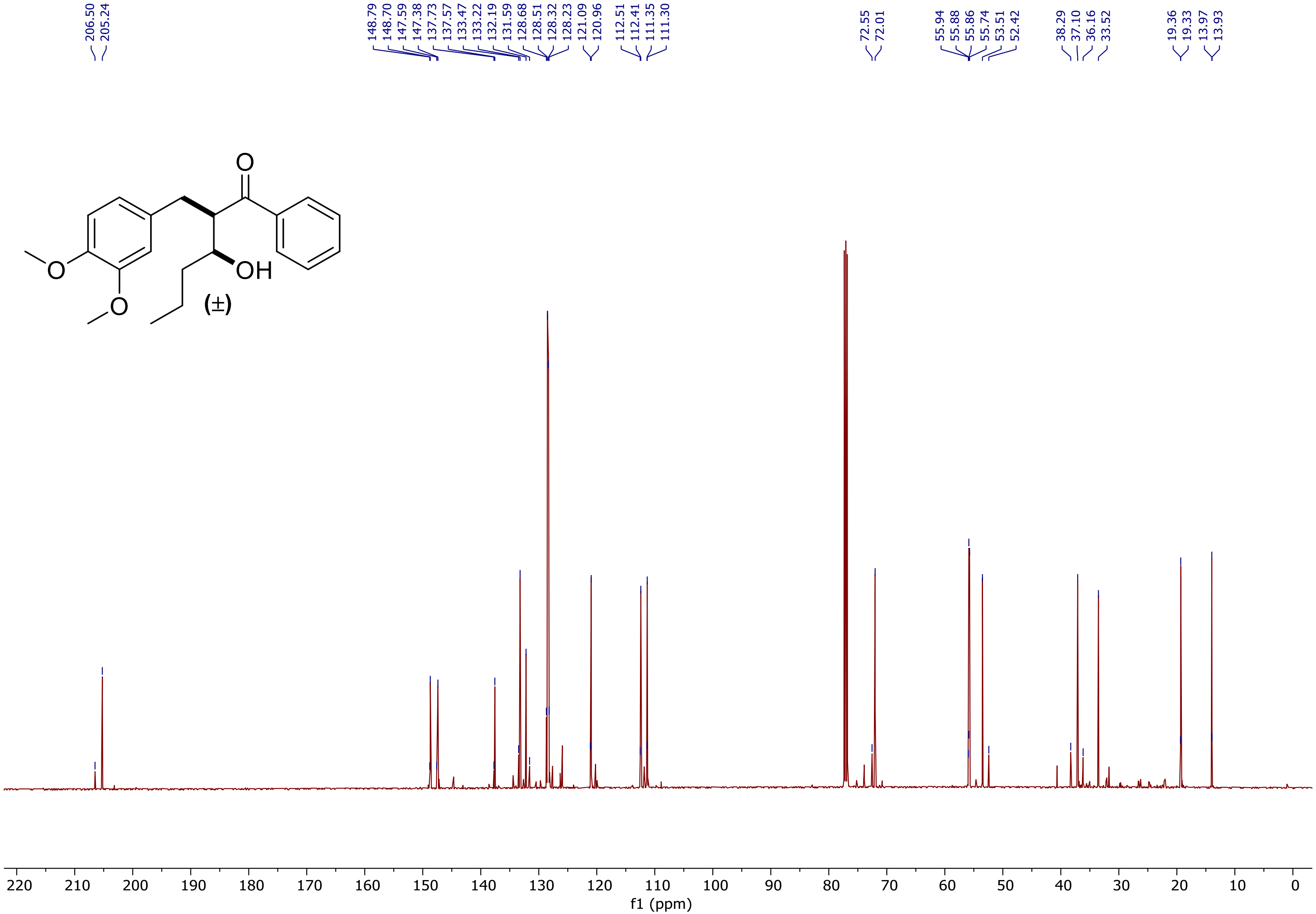

^{13}C NMR (126 MHz, $CDCl_3$) Spectrum of (2*RS*,3*RS*)-3-hydroxy-2-(3,4-dimethoxybenzyl)-1-phenyl-1-hexanone **3k**.

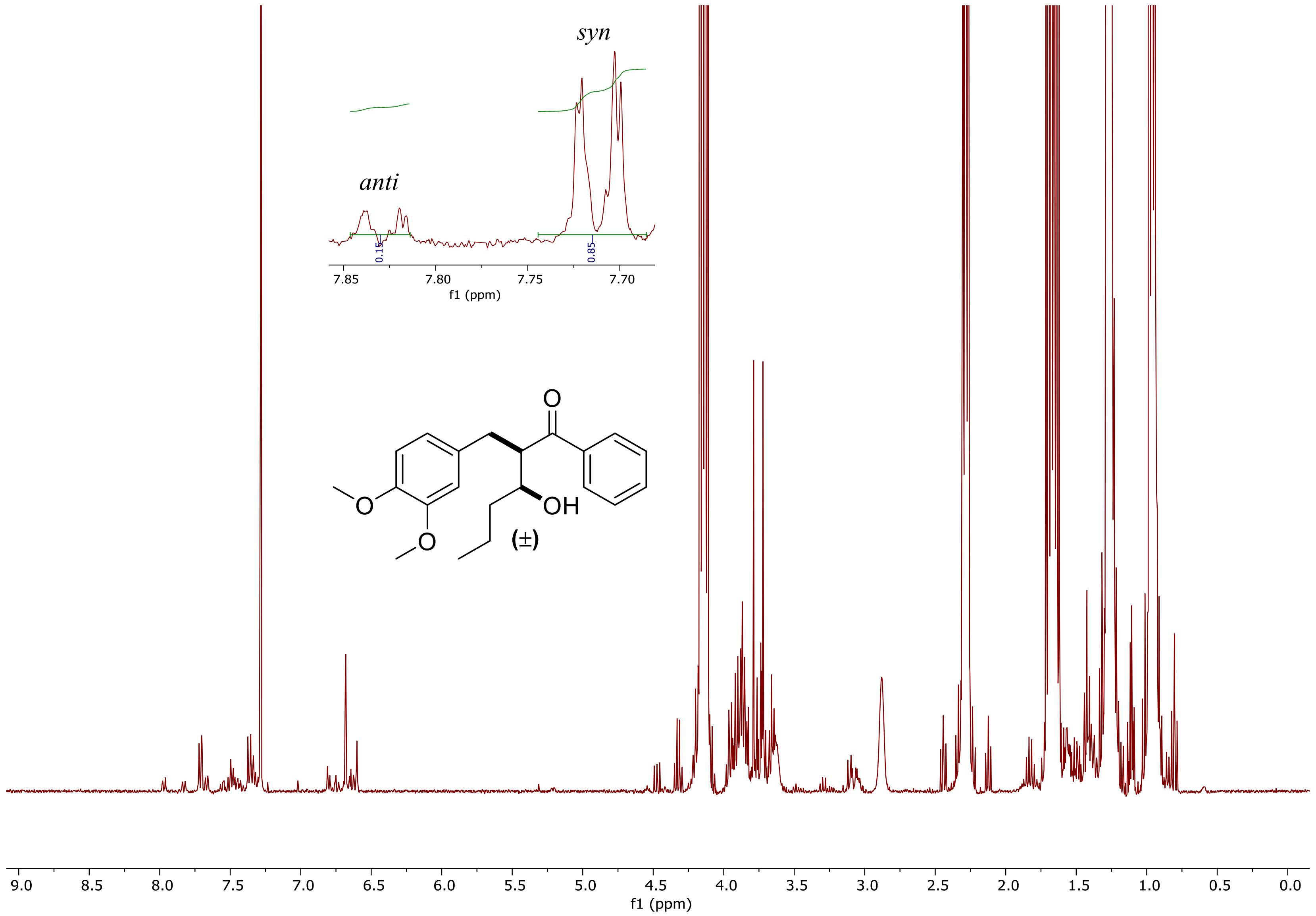

^{1}H NMR (400 MHz, $CDCl_3$) Spectrum of the crude reaction mixture for (2*RS*,3*RS*)-3-hydroxy-2-(3,4-dimethoxybenzyl)-1-phenyl-1-hexanone **3k**.

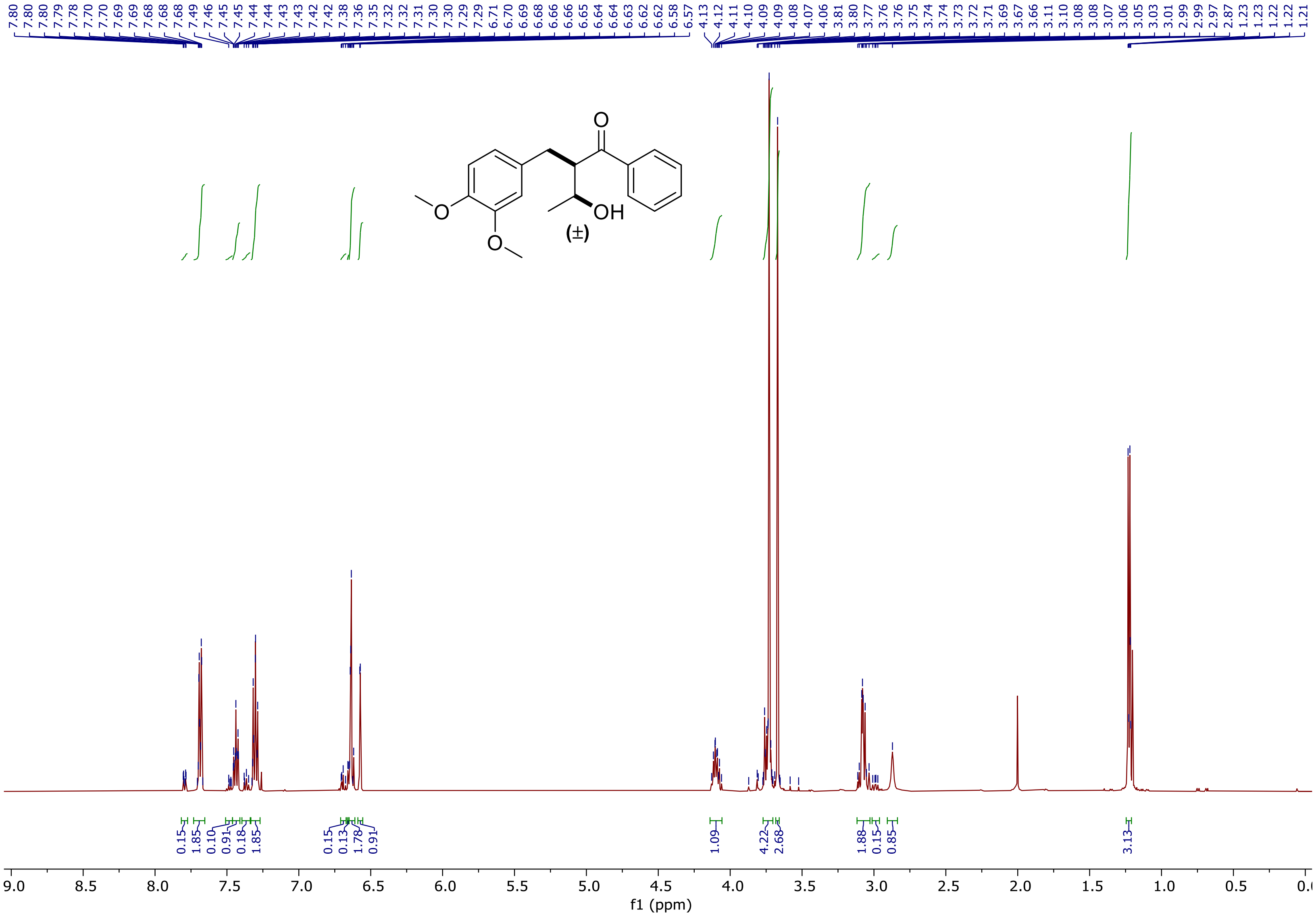

^{1}H NMR (500 MHz, $CDCl_3$) Spectrum of (2*RS*,3*RS*)-3-hydroxy-2-(3,4-dimethoxybenzyl)-1-phenyl-1-butanone **3l**.

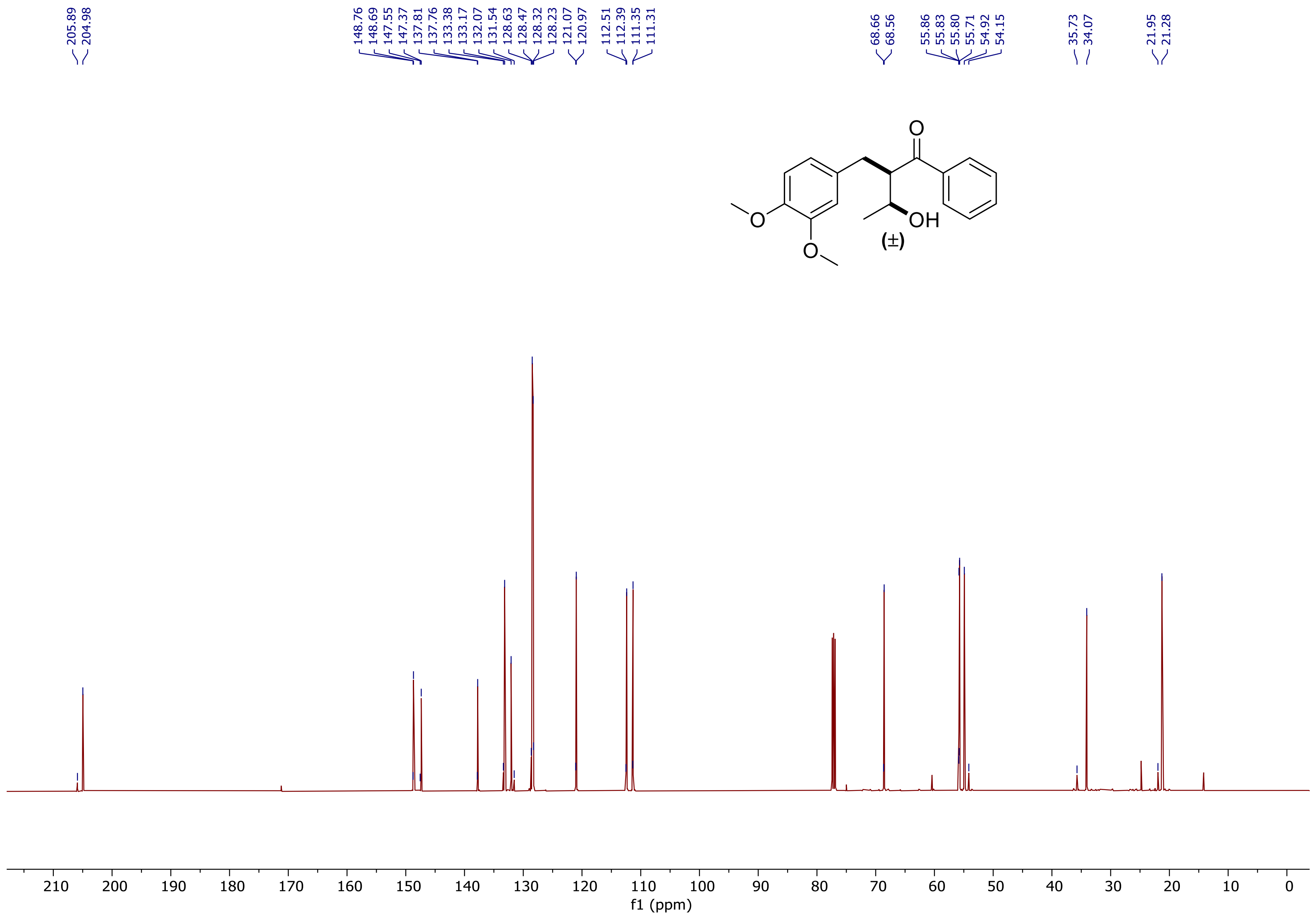

^{13}C NMR (126 MHz, $CDCl_3$) Spectrum of (2*RS*,3*RS*)-3-hydroxy-2-(3,4-dimethoxybenzyl)-1-phenyl-1-butanone **3l**.

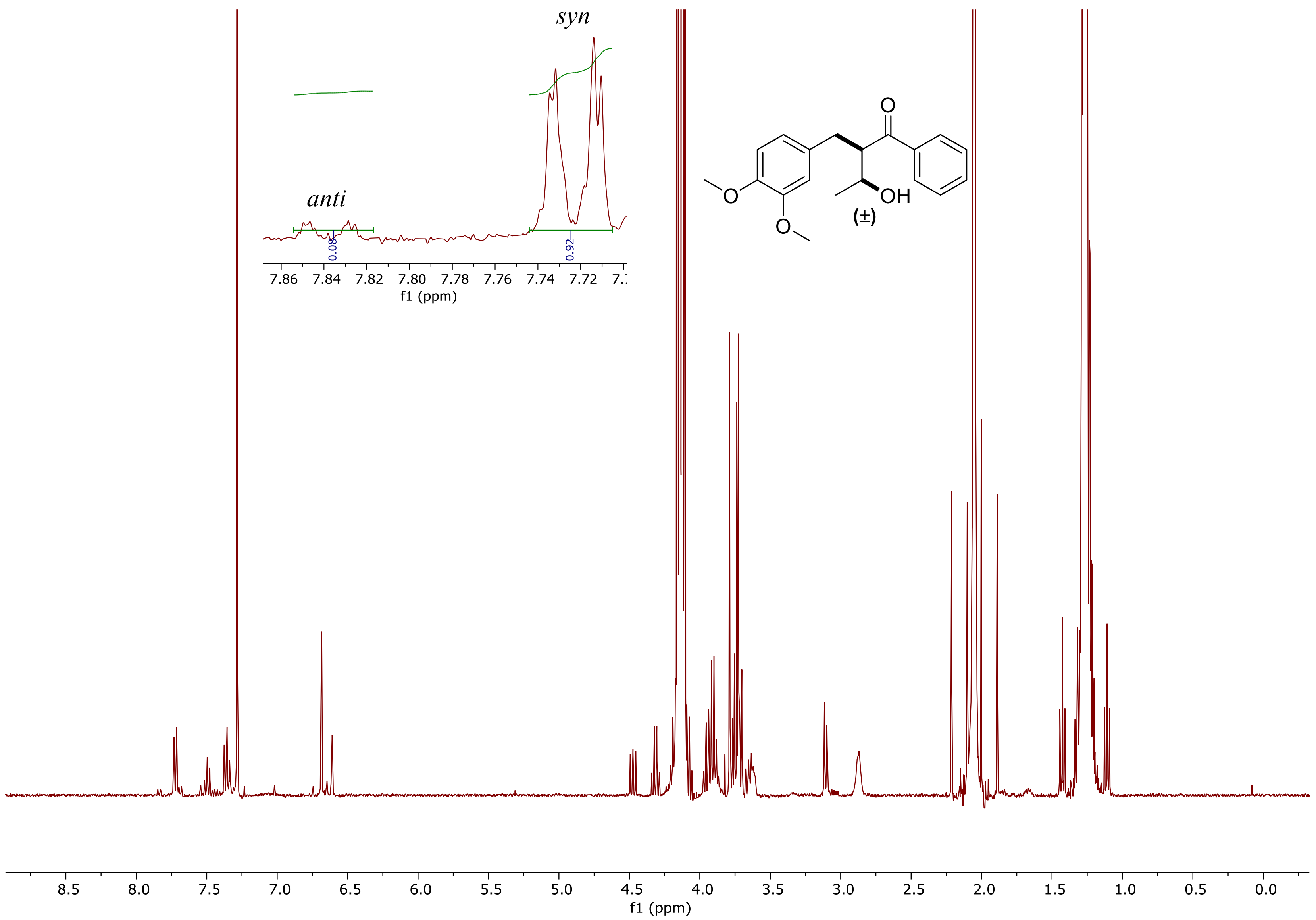

^{1}H NMR (400 MHz, $CDCl_3$) Spectrum of the crude reaction mixture for (2*RS*,3*RS*)-3-hydroxy-2-(3,4-dimethoxybenzyl)-1-phenyl-1-butanone **3l**.

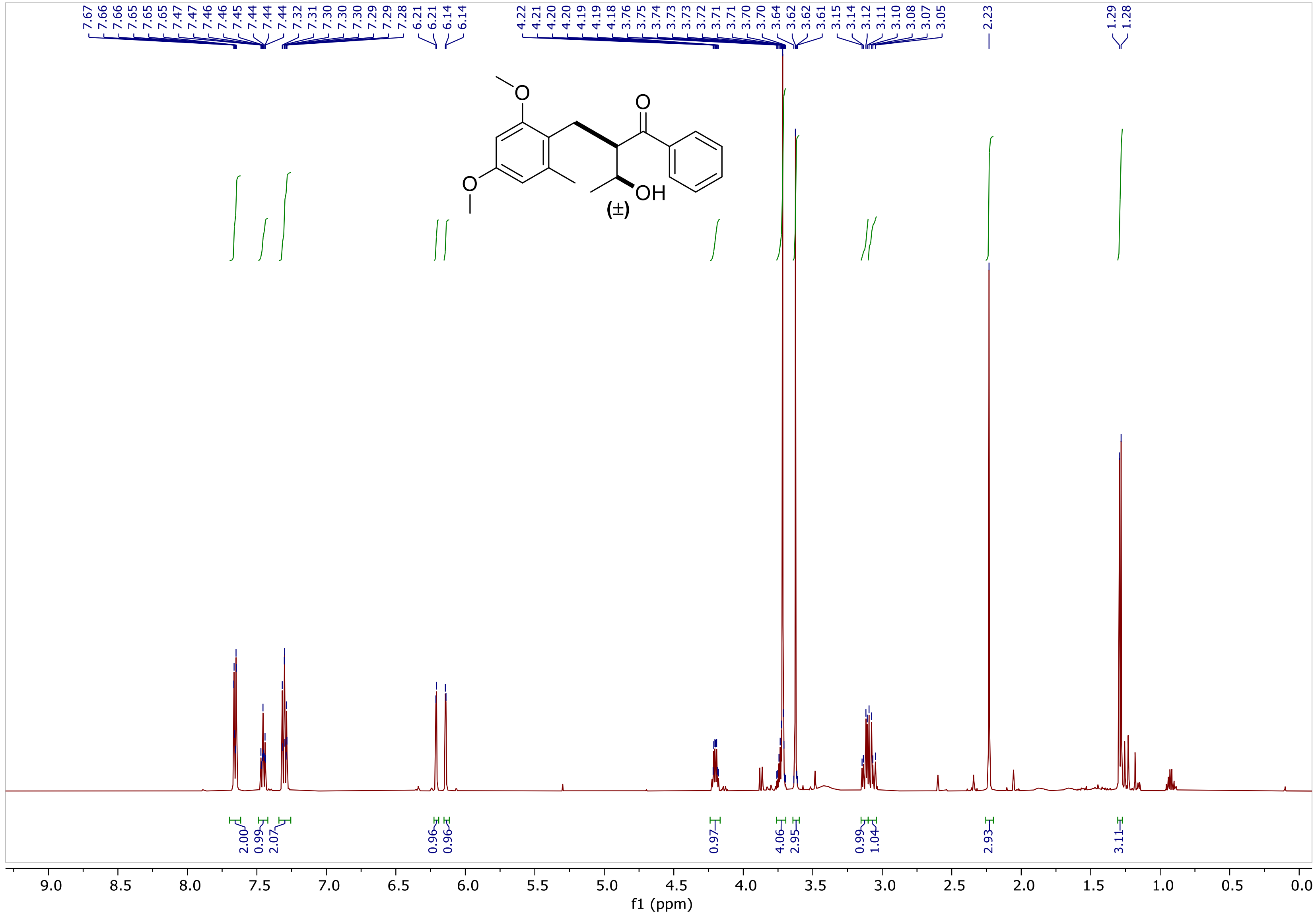

^{1}H NMR (500 MHz, $CDCl_3$) Spectrum of (2*RS*,3*RS*)-3-hydroxy-2-(2,4-dimethoxy-6-methylbenzyl)-1-phenyl-1-butanone **3m**.

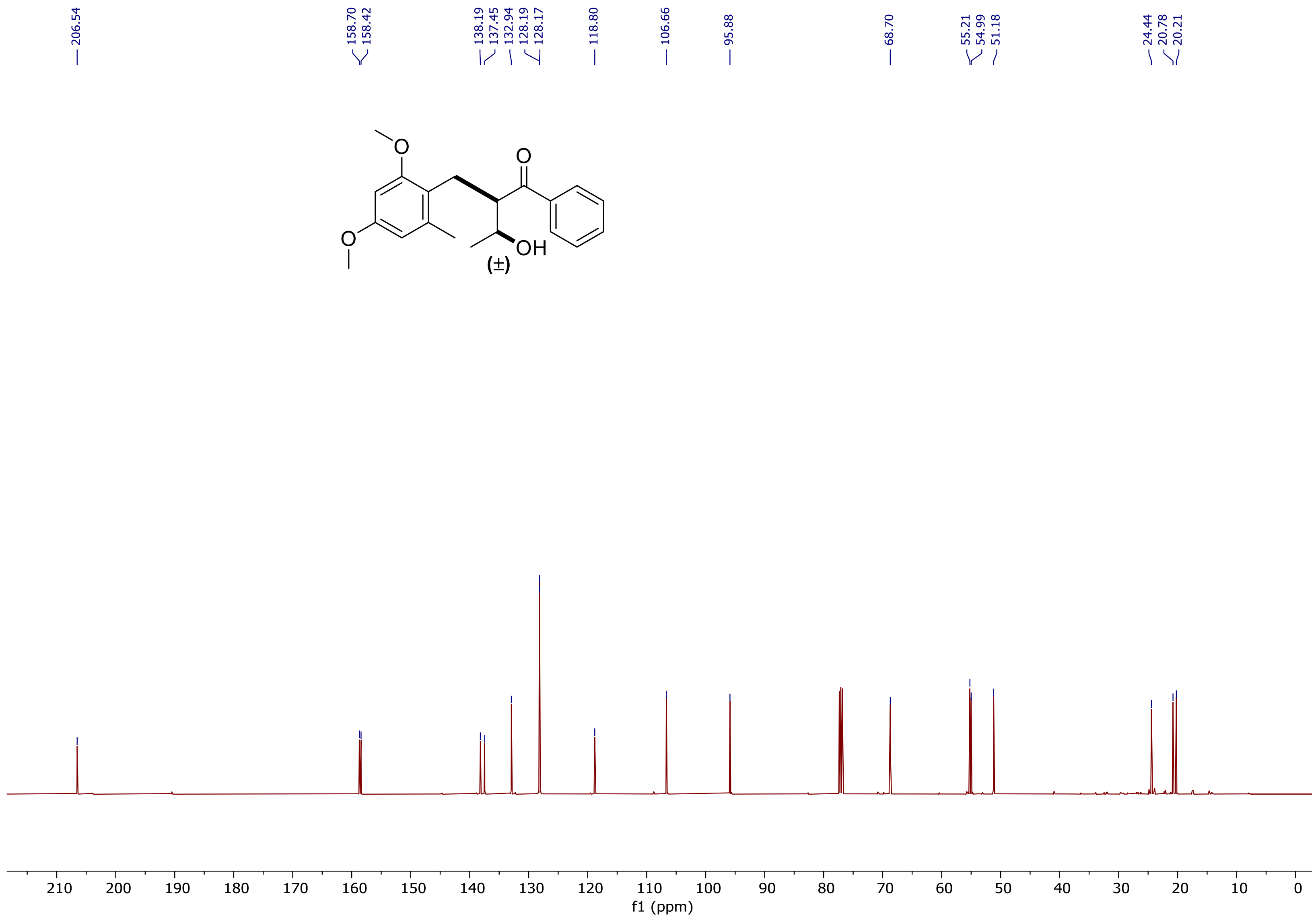

^{13}C NMR (126 MHz, $CDCl_3$) Spectrum of (2*RS*,3*RS*)-3-hydroxy-2-(2,4-dimethoxy-6-methylbenzyl)-1-phenyl-1-butanone **3m**.

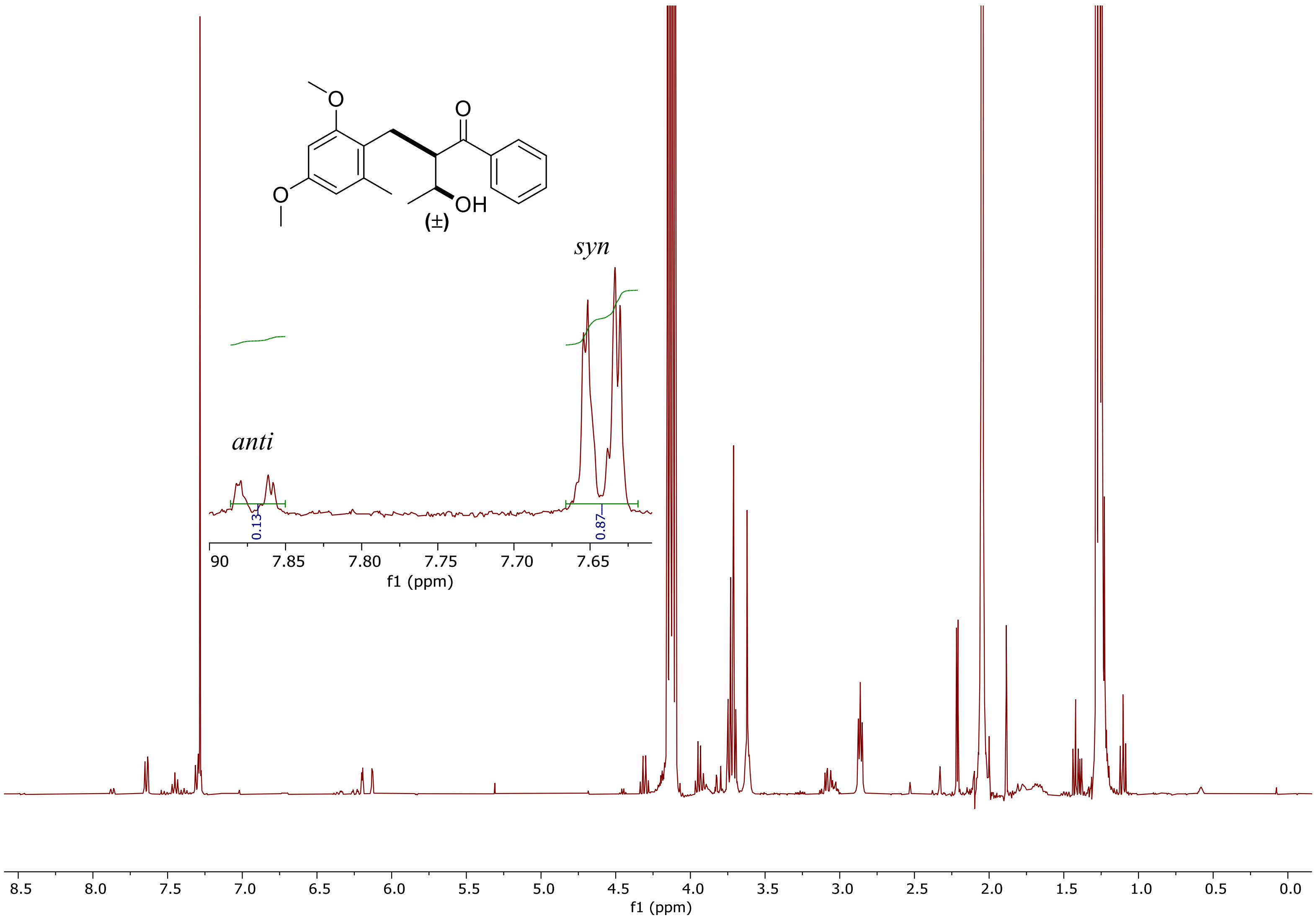

^{1}H NMR (400 MHz, $CDCl_3$) Spectrum of the crude reaction mixture for (2*RS*,3*RS*)-3-hydroxy-2-(2,4-dimethoxy-6-methylbenzyl)-1-phenyl-1-butanone **3m**.

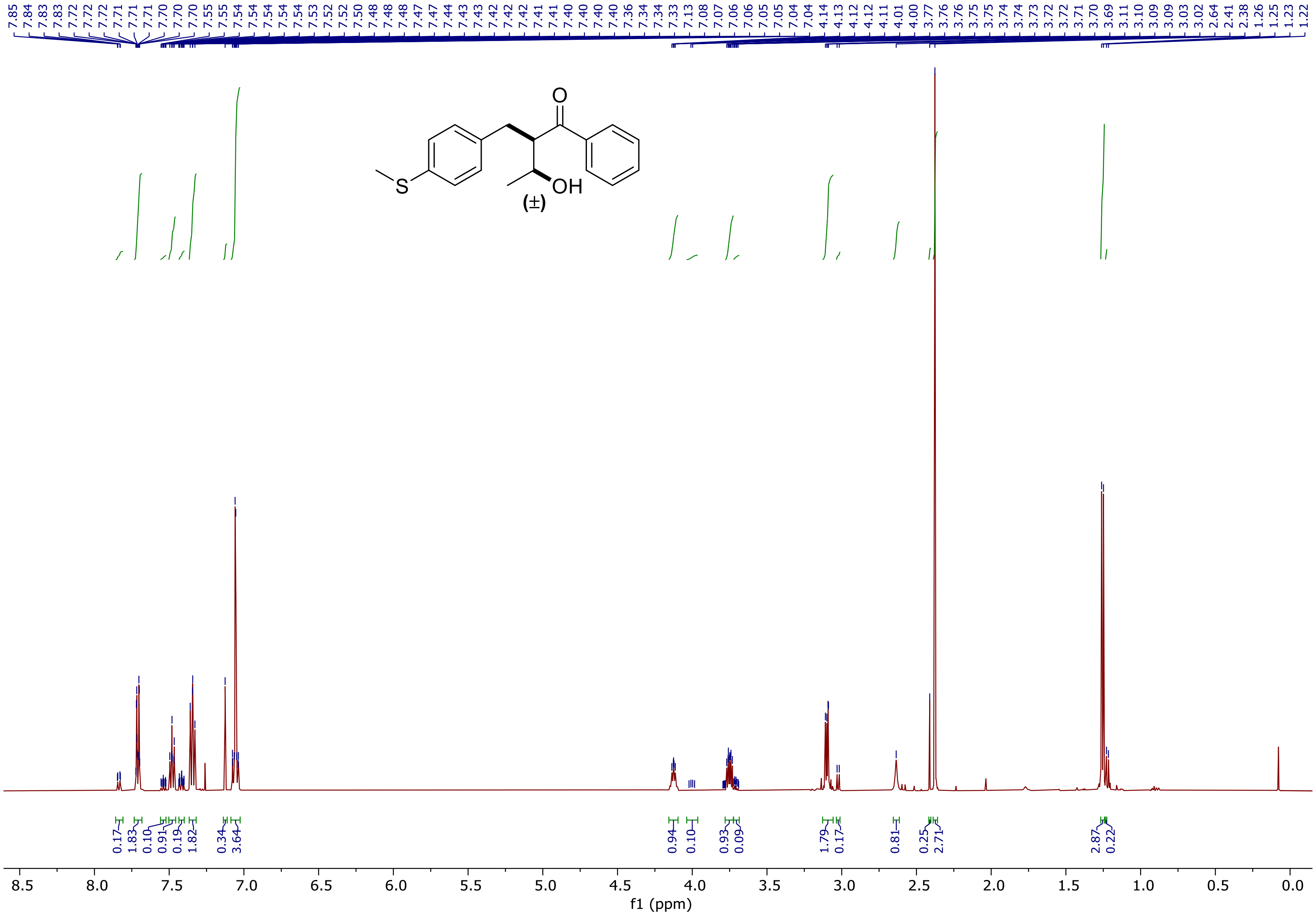

[1]**H NMR** (500 MHz, $CDCl_3$) Spectrum of (2*RS*,3*RS*)-3-hydroxy-2-(4-thiomethoxybenzyl)-1-phenyl-1-butanone **3n**.

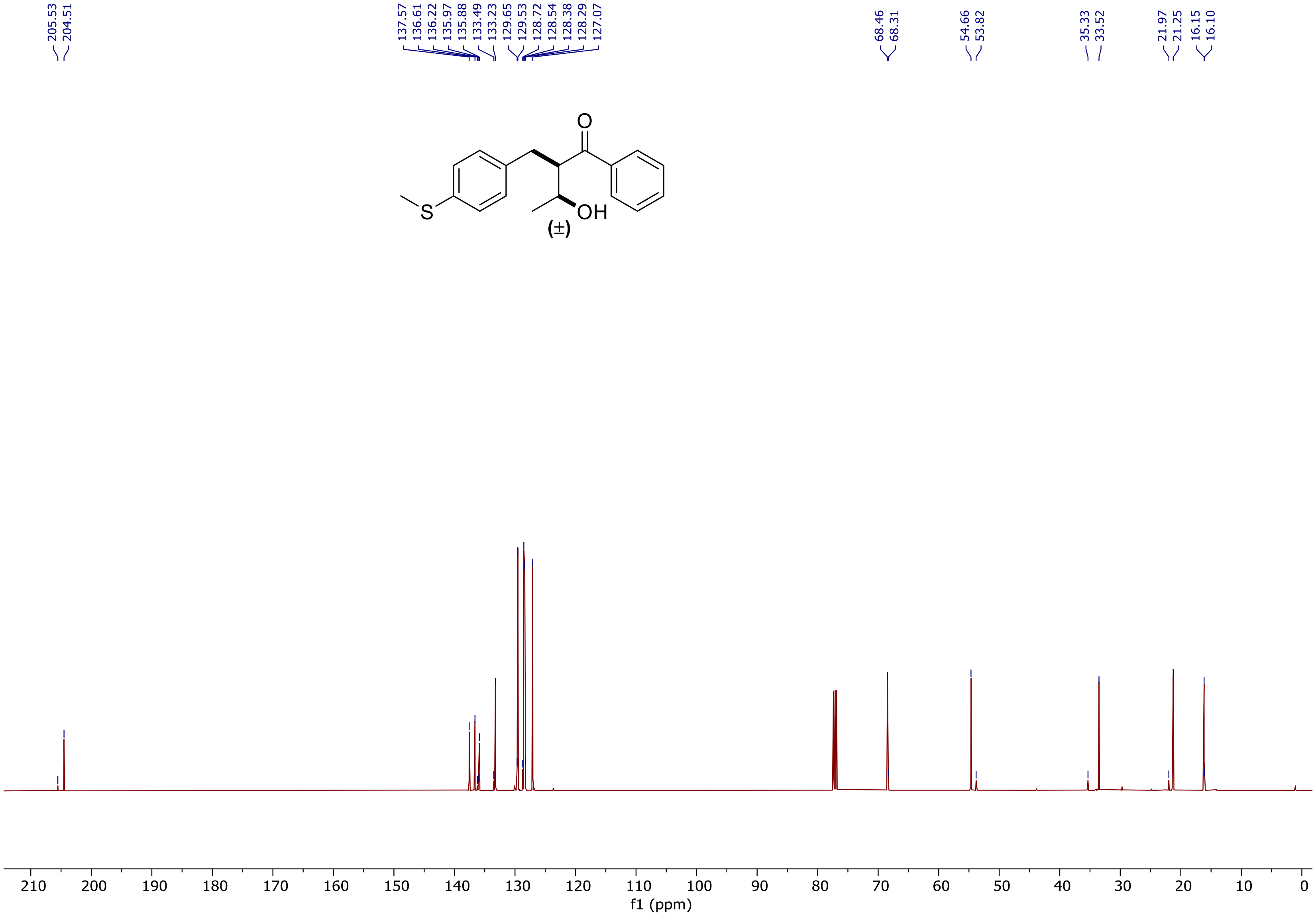

^{13}C NMR (126 MHz, $CDCl_3$) Spectrum of (2*RS*,3*RS*)-3-hydroxy-2-(4-thiomethoxybenzyl)-1-phenyl-1-butanone **3n**.

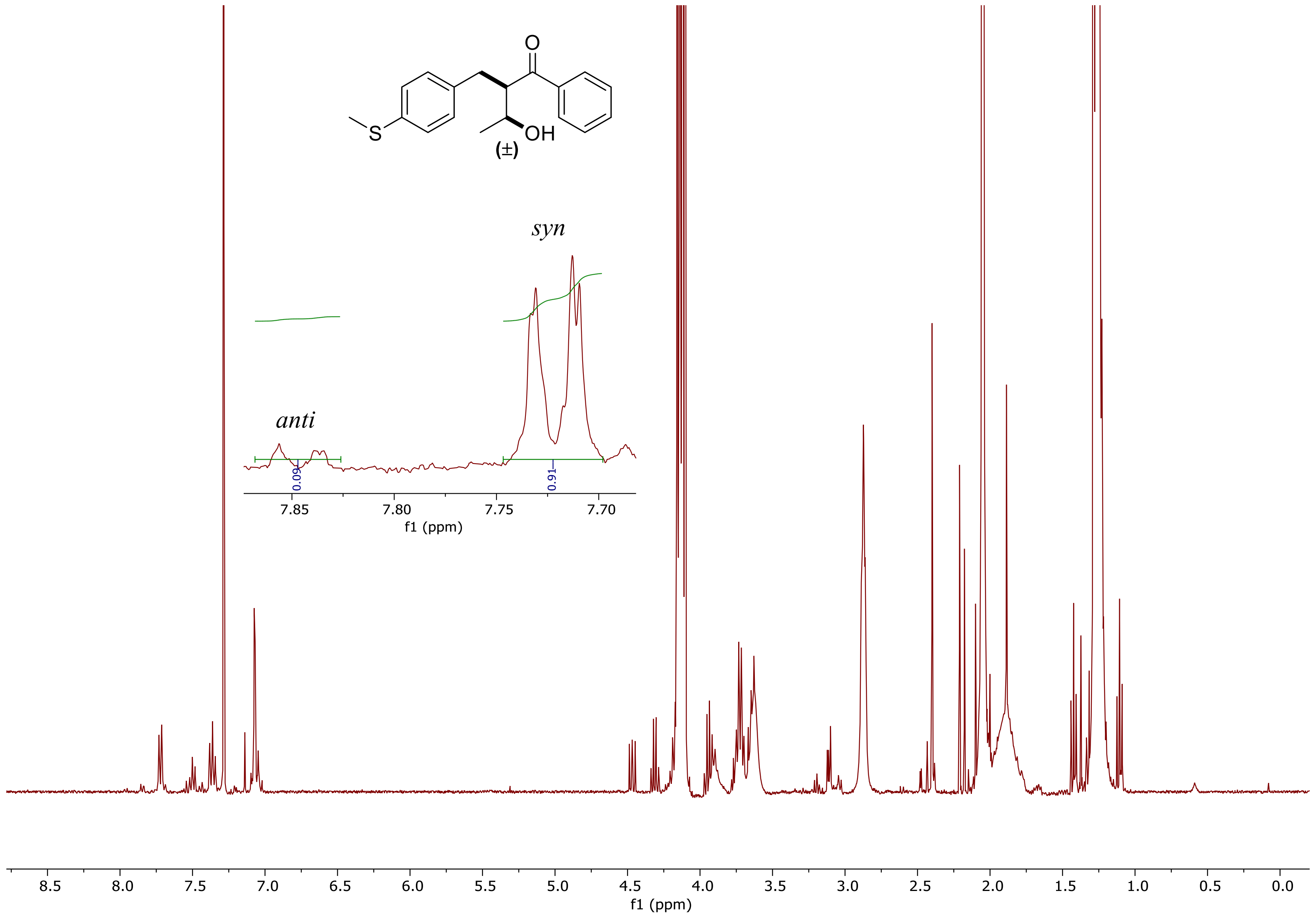

^{1}H NMR (400 MHz, $CDCl_3$) Spectrum of the crude reaction mixture for (2*RS*,3*RS*)-3-hydroxy-2-(4-thiomethoxybenzyl)-1-phenyl-1-butanone **3n**.

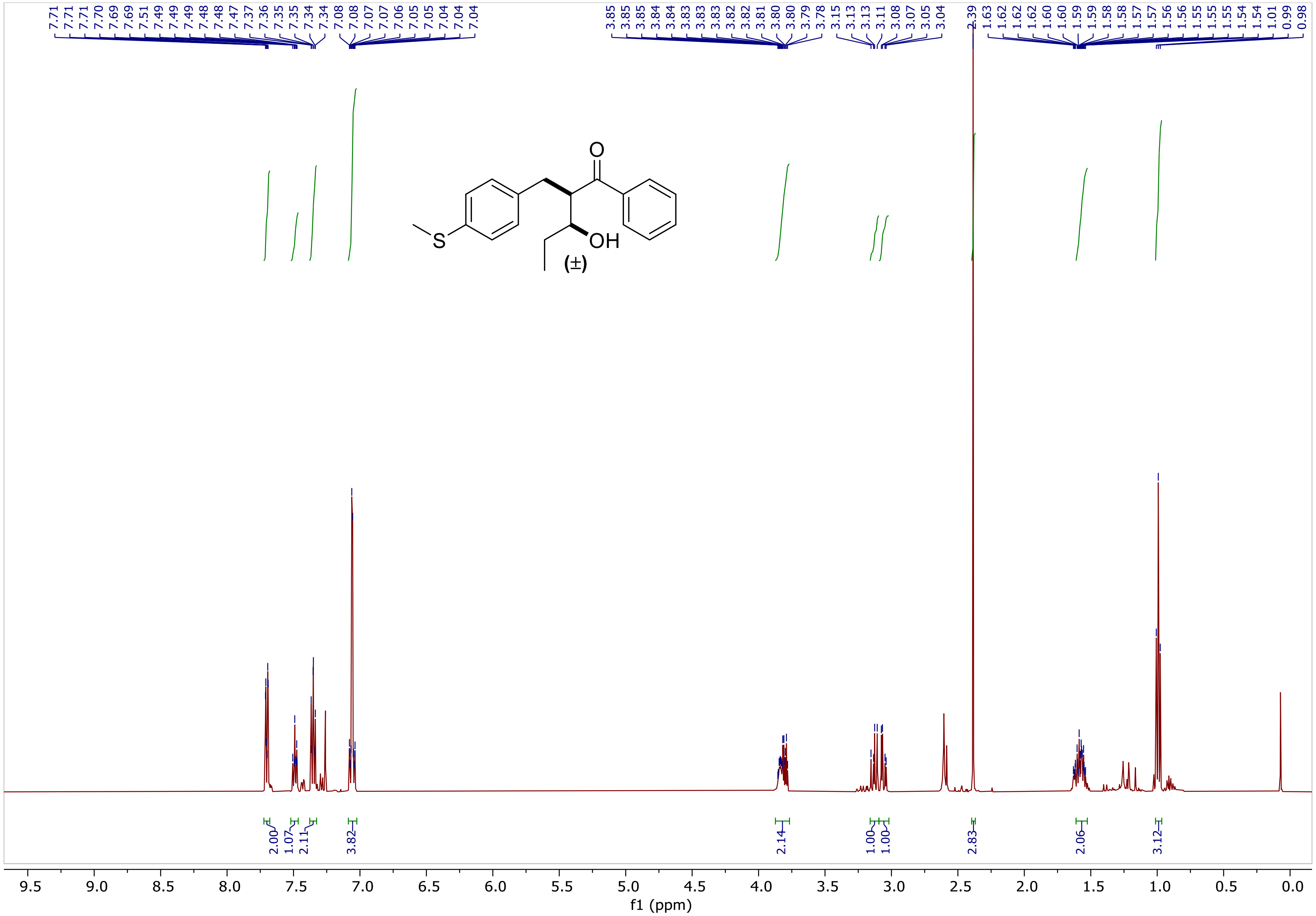

^{1}H NMR (500 MHz, $CDCl_3$) Spectrum of (2*RS*,3*RS*)-3-hydroxy-2-(4-thiomethoxybenzyl)-1-phenyl-1-pentanone **3o**.

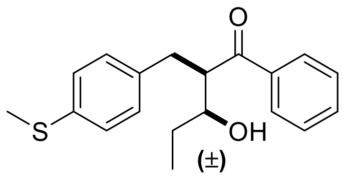

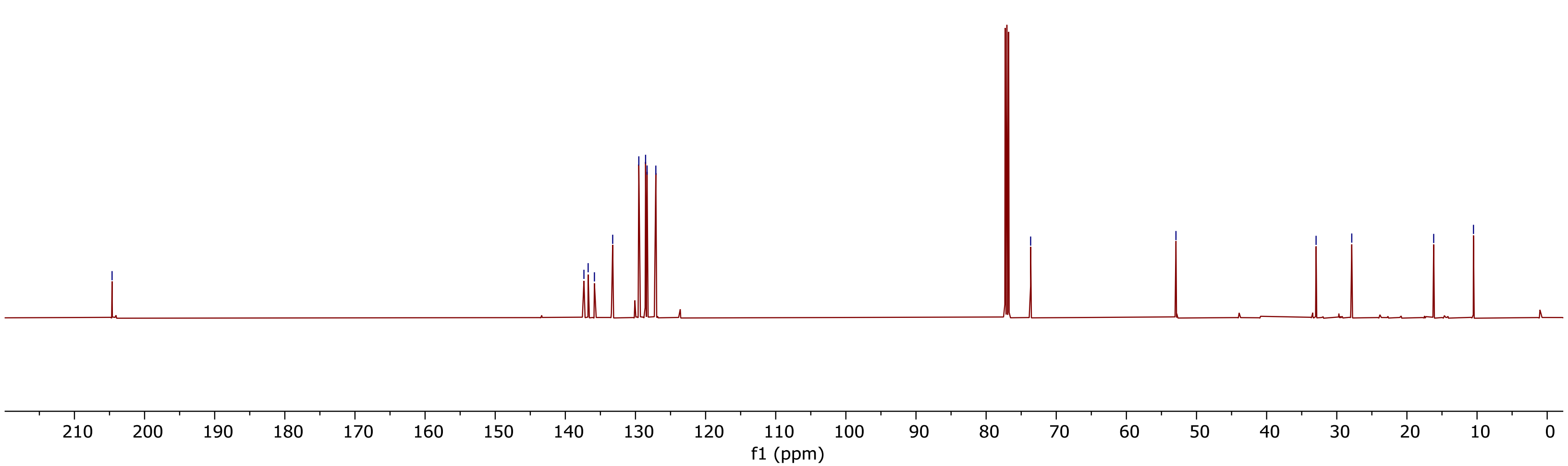

^{13}C NMR (126 MHz, $CDCl_3$) Spectrum of (2*RS*,3*RS*)-3-hydroxy-2-(4-thiomethoxybenzyl)-1-phenyl-1-pentanone **3o**.

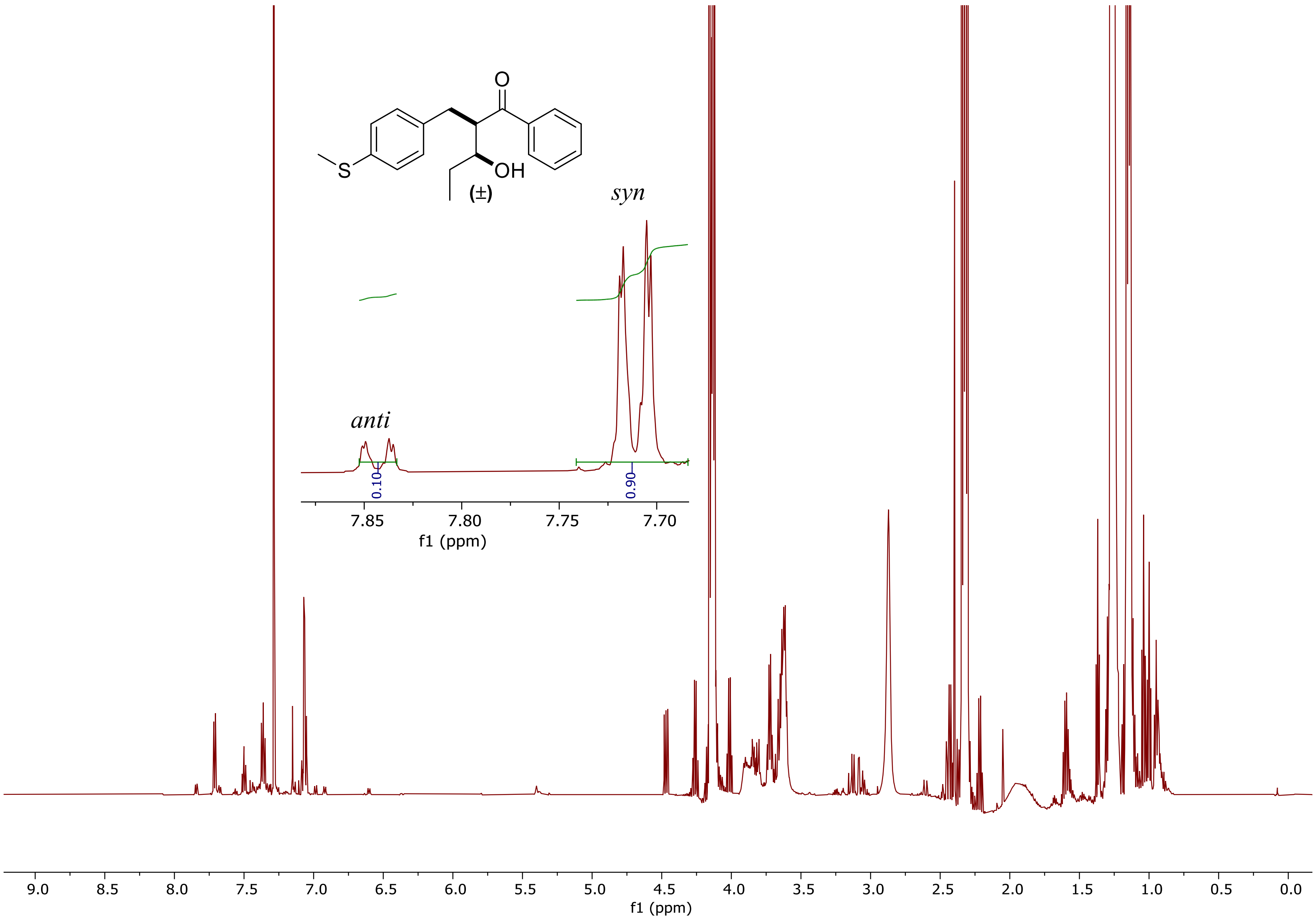

^{1}H NMR (400 MHz, $CDCl_3$) Spectrum of the crude reaction mixture for (2*RS*,3*RS*)-3-hydroxy-2-(4-thiomethoxybenzyl)-1-phenyl-1-pentanone **3o**.

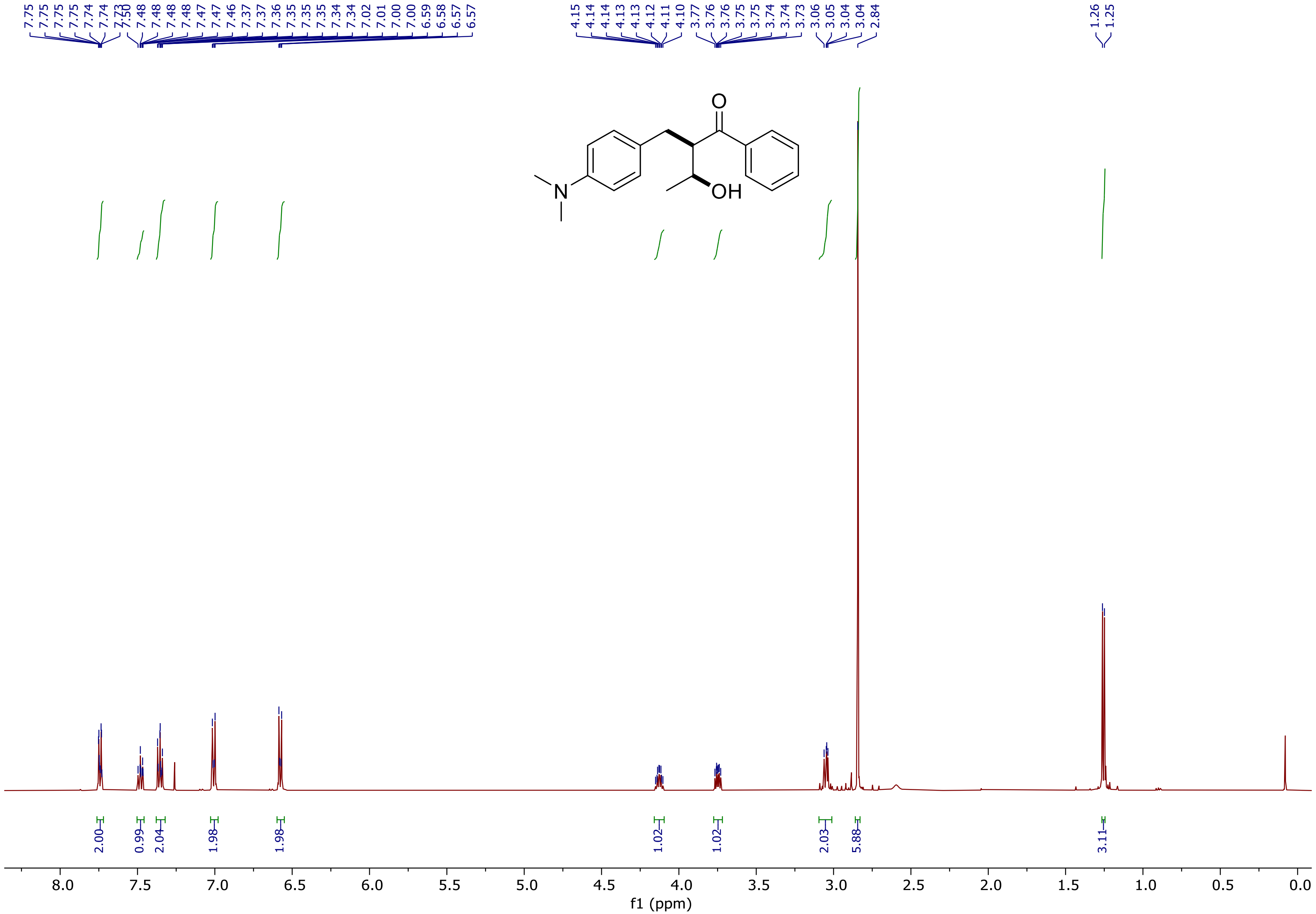

^{1}H NMR (500 MHz, $CDCl_3$) Spectrum of (2*RS*,3*RS*)-3-hydroxy-2-(4-(dimethyamino)benzyl)-1-phenyl-1-butanone **3p**.

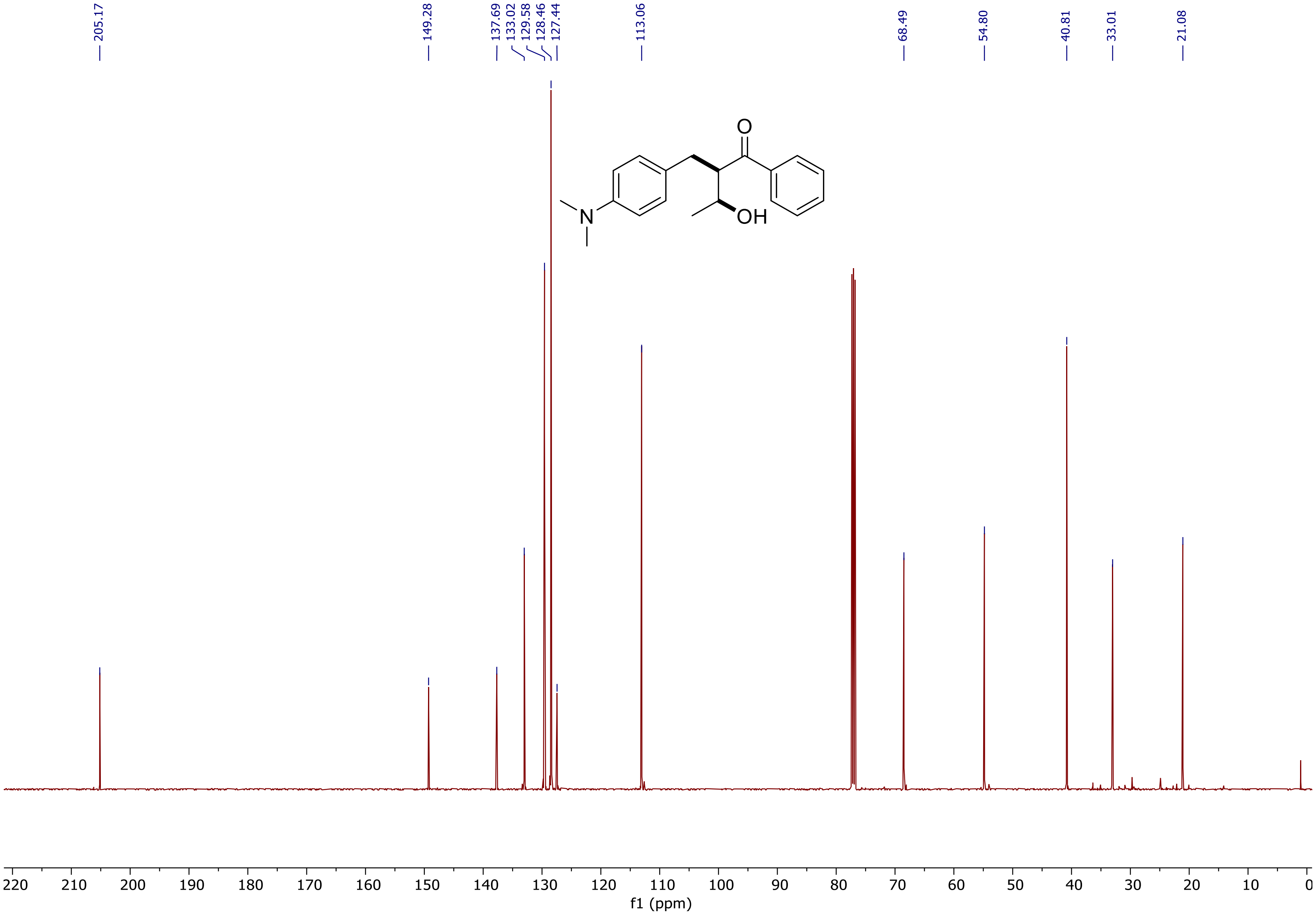

^{13}C NMR (126 MHz, $CDCl_3$) Spectrum of (2*RS*,3*RS*)-3-hydroxy-2-(4-(dimethyamino)benzyl)-1-phenyl-1-butanone **3p**.

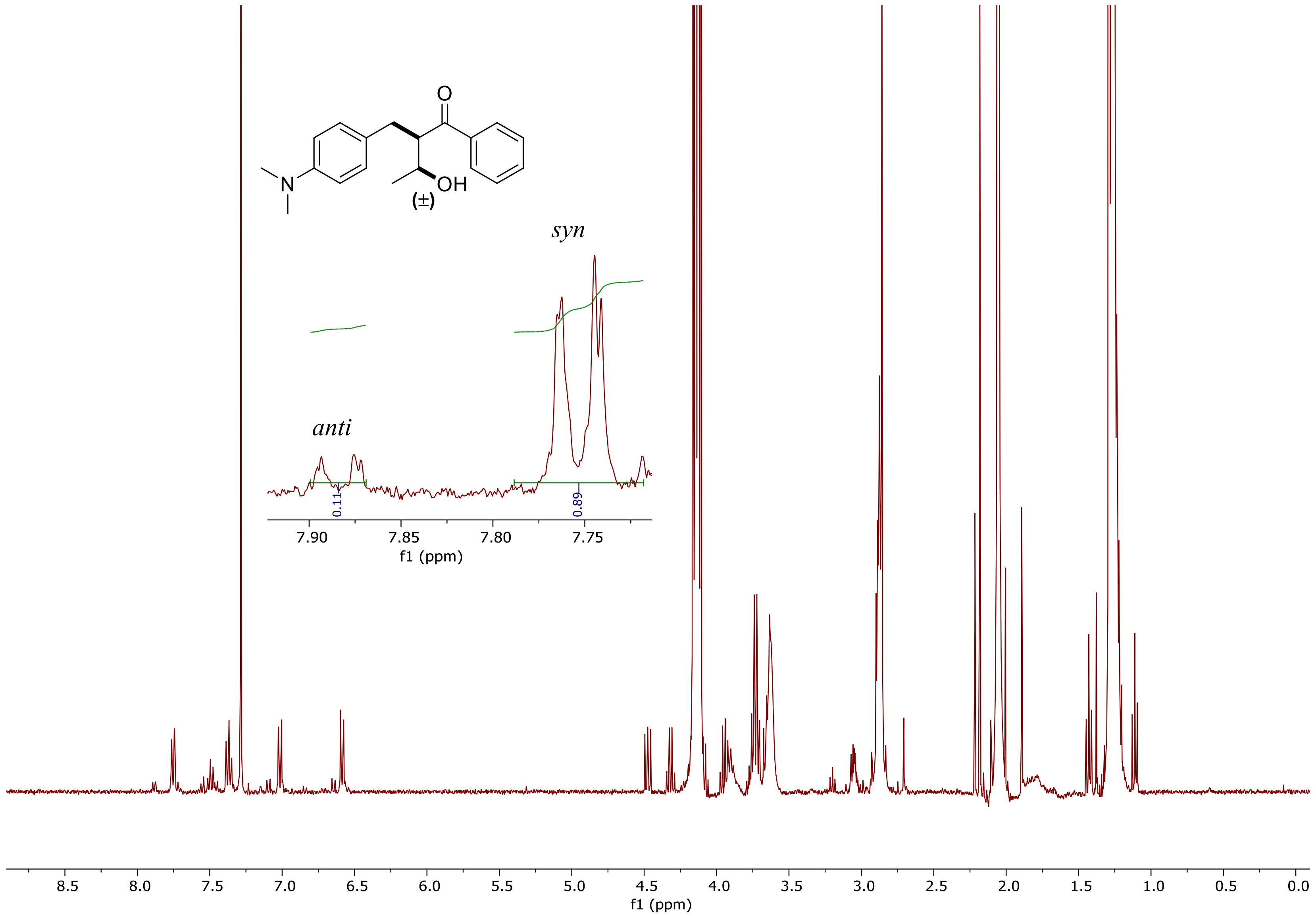

^{1}H NMR (400 MHz, $CDCl_3$) Spectrum of the crude reaction mixture for (2*RS*,3*RS*)-3-hydroxy-2-(4-(dimethyamino)benzyl)-1-phenyl-1-butanone **3p**.

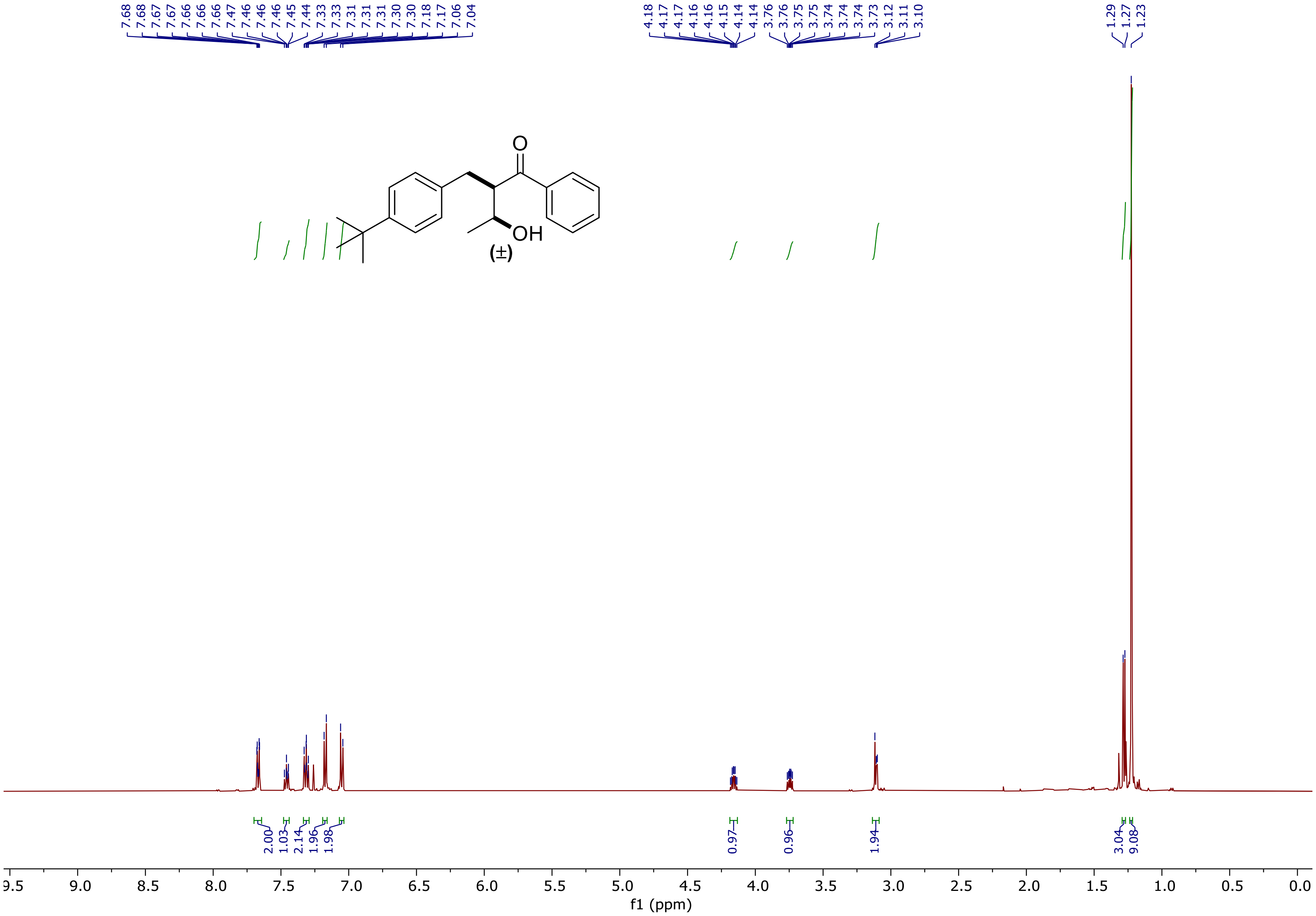

^{1}H NMR (500 MHz, $CDCl_3$) Spectrum of (2*RS*,3*RS*)-2-(4-*tert*-butylbenzyl)-3-hydroxy-1-phenyl-1-butanone **3q**.

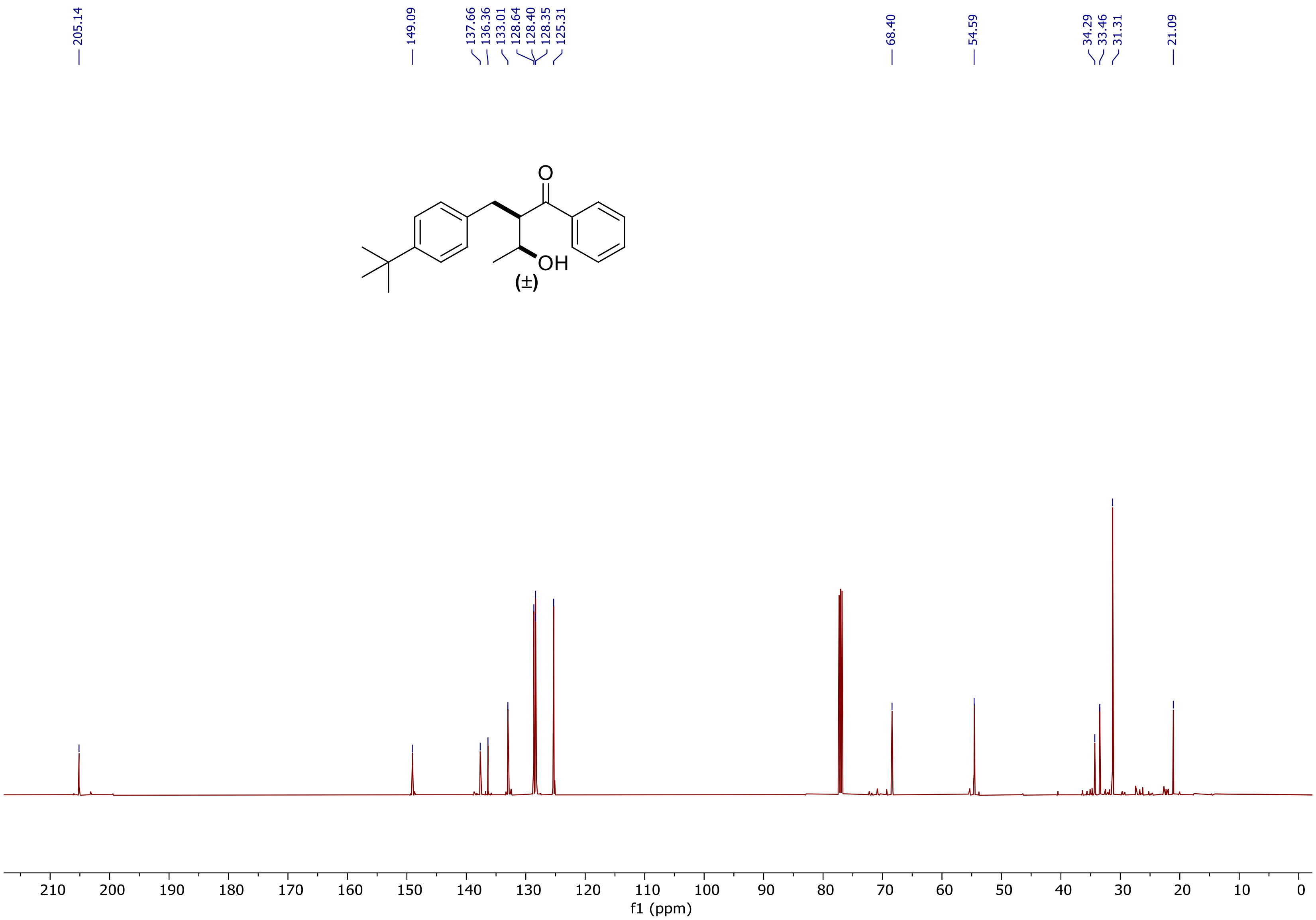

^{13}C NMR (126 MHz, $CDCl_3$) Spectrum of (2*RS*,3*RS*)-2-(4-*tert*-butylbenzyl)-3-hydroxy-1-phenyl-1-butanone **3q**.

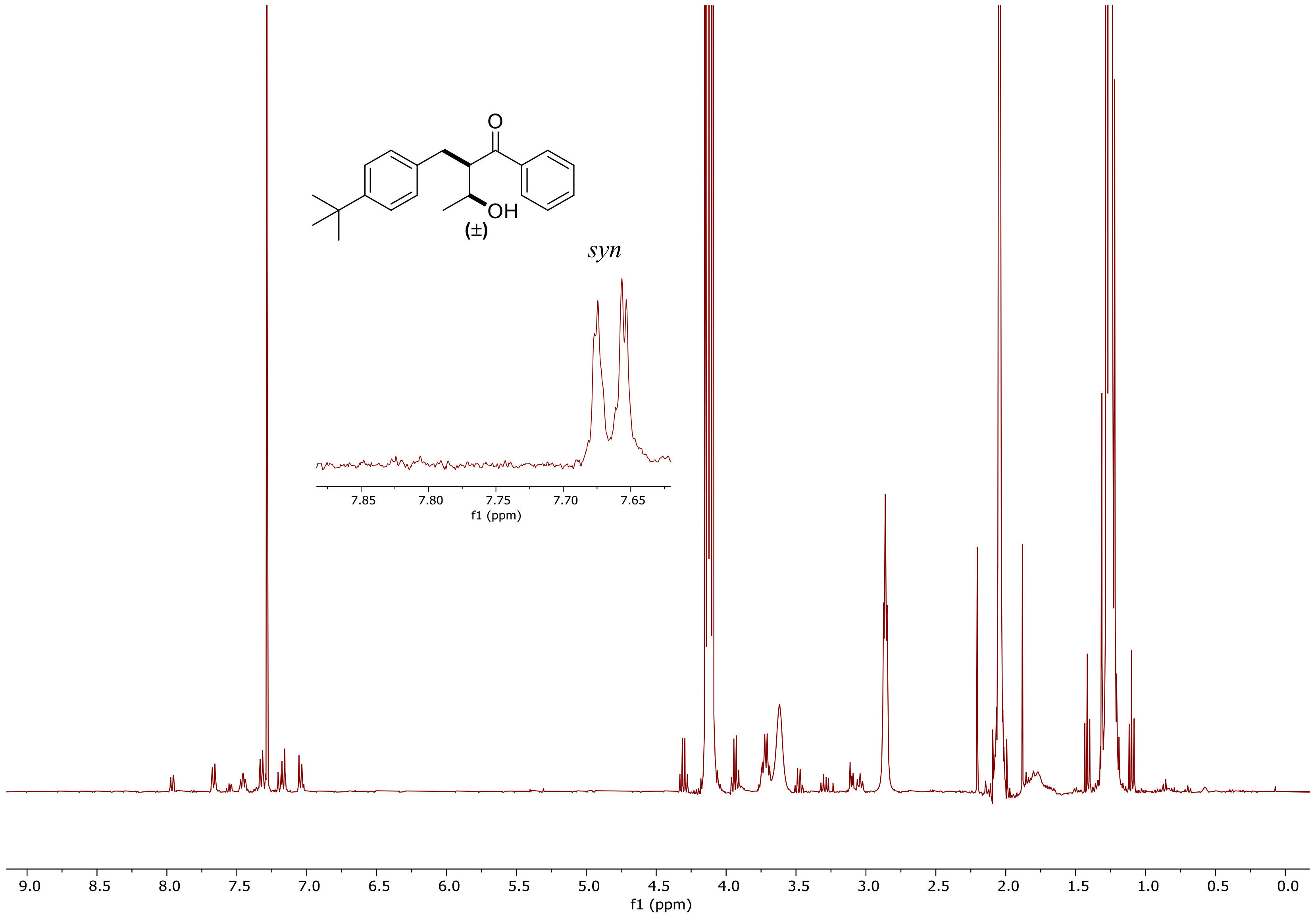

^{1}H NMR (400 MHz, $CDCl_3$) Spectrum of the crude reaction mixture for (2*RS*,3*RS*)-2-(4-*tert*-butylbenzyl)-3-hydroxy-1-phenyl-1-butanone **3q**.

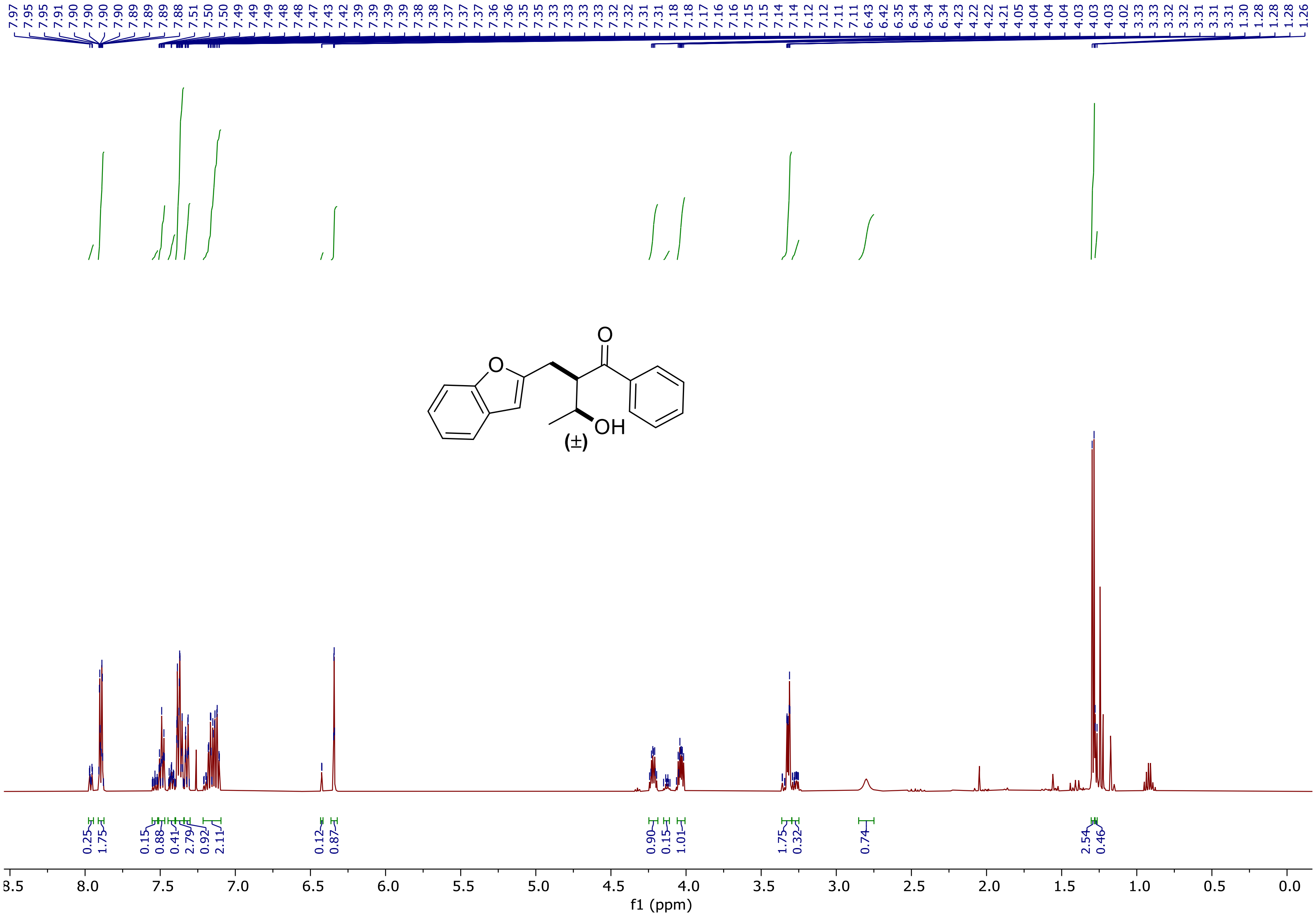

^{1}**H NMR** (500 MHz, $CDCl_3$) Spectrum of (2*RS*,3*RS*)-2-(benzofuran-2-ylmethyl)-3-hydroxy-1-phenyl-1-butanone **3r**.

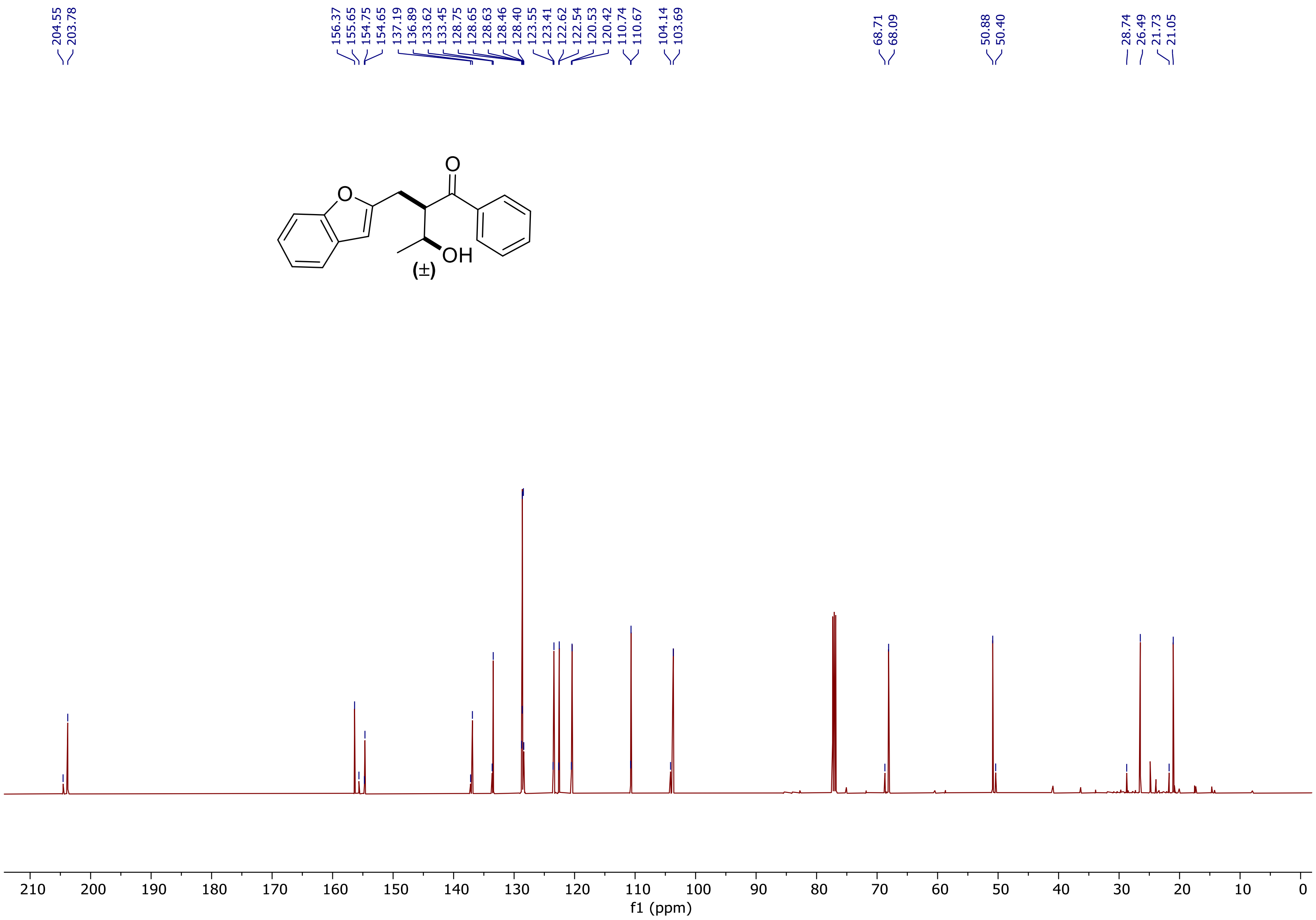

^{13}C NMR (126 MHz, $CDCl_3$) Spectrum of (2*RS*,3*RS*)-2-(benzofuran-2-ylmethyl)-3-hydroxy-1-phenyl-1-butanone **3r**.

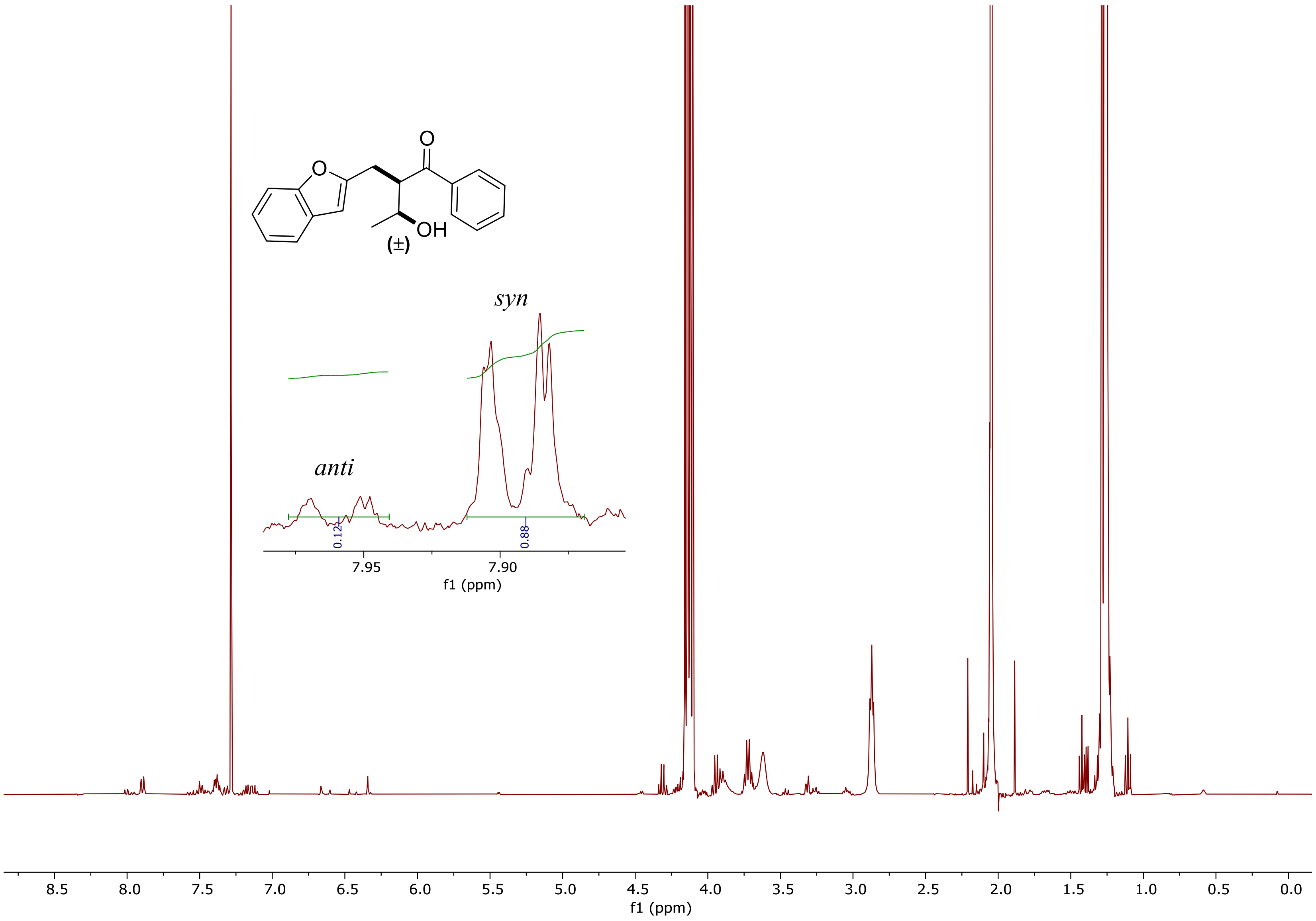

^{1}H NMR (400 MHz, $CDCl_3$) Spectrum of the crude reaction mixture for (2*RS*,3*RS*)-2-(benzofuran-2-ylmethyl)-3-hydroxy-1-phenyl-1-butanone **3r**.

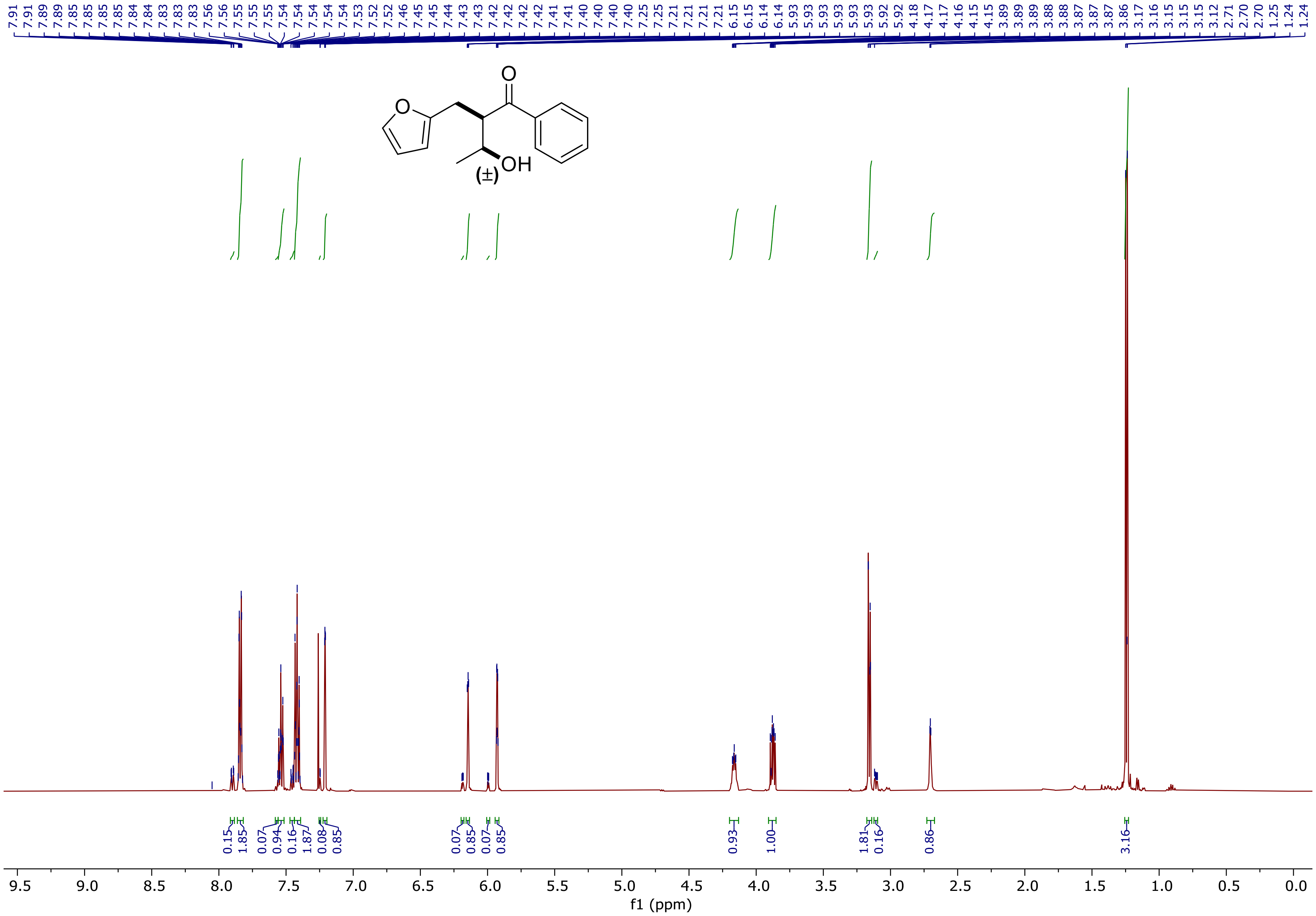

^{1}H NMR (500 MHz, $CDCl_3$) Spectrum of (2*RS*,3*RS*)-3-hydroxy-2-(2-methylfuryl)-1-phenyl-1-butanone **3s**.

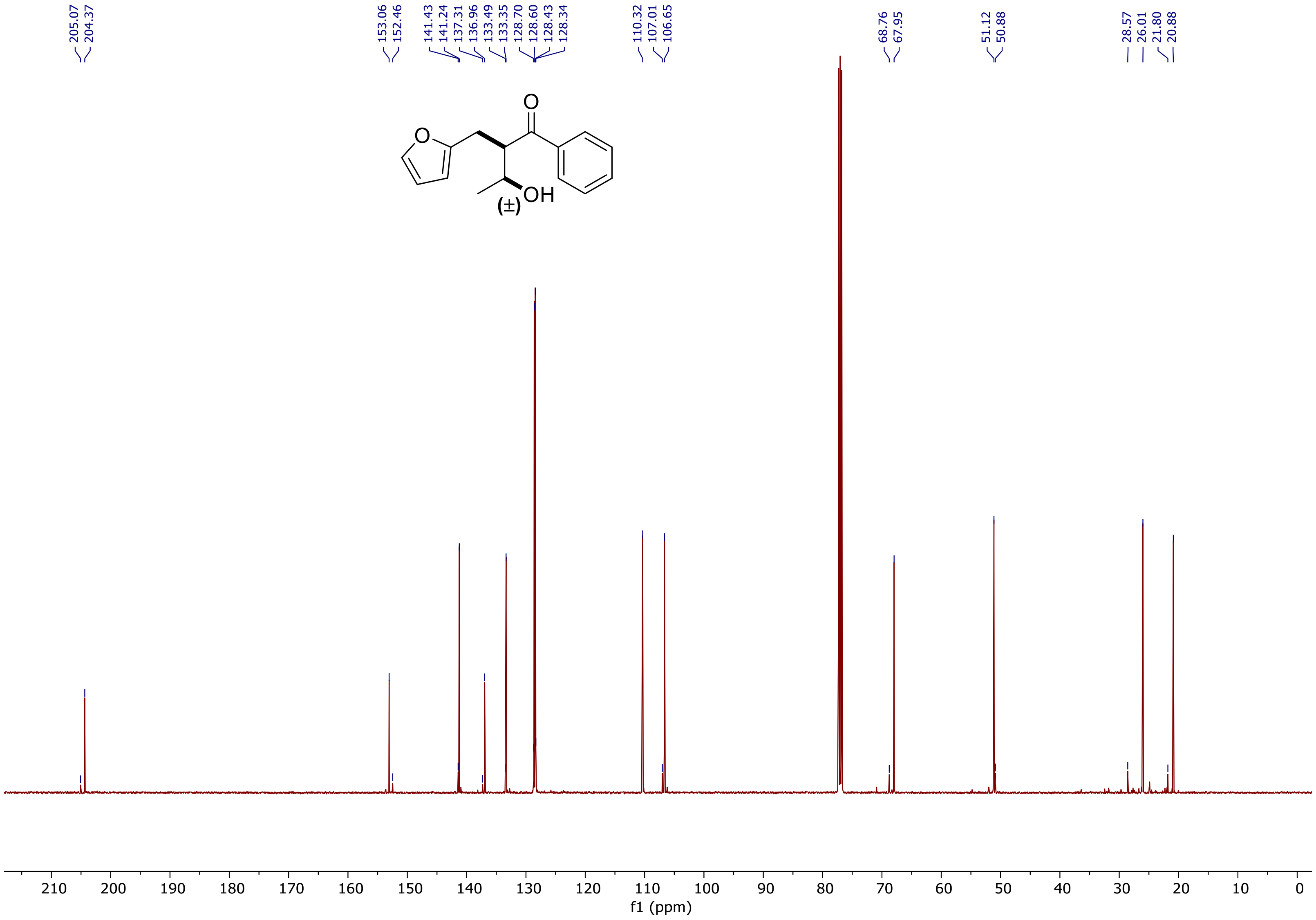

^{13}C NMR (126 MHz, $CDCl_3$) Spectrum of (2*RS*,3*RS*)-3-hydroxy-2-(2-methylfuryl)-1-phenyl-1-butanone **3s**.

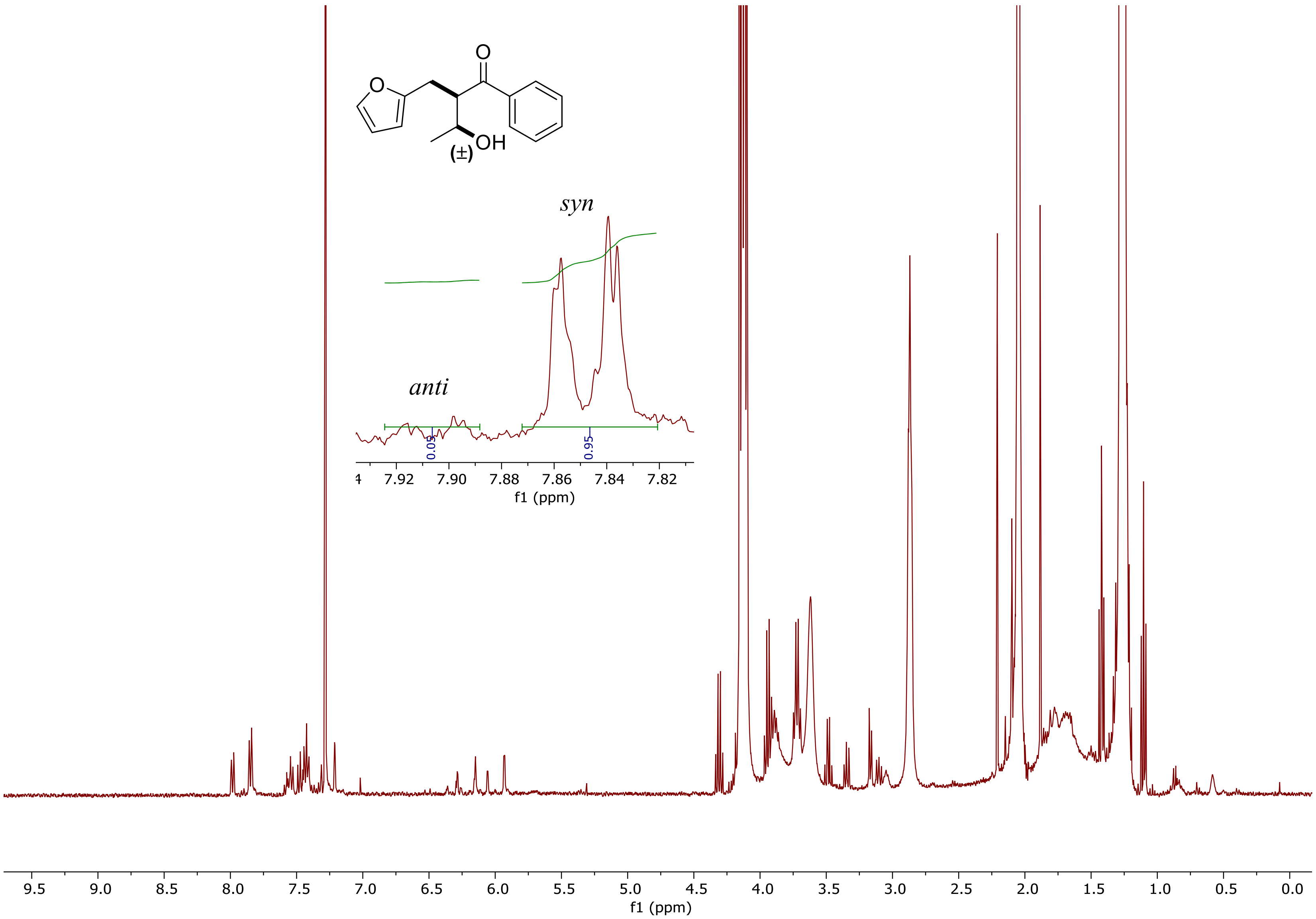

^{1}**H NMR** (400 MHz, $CDCl_3$) Spectrum of the crude reaction mixture for (2*RS*,3*RS*)-3-hydroxy-2-(2-methylfuryl)-1-phenyl-1-butanone **3s**.

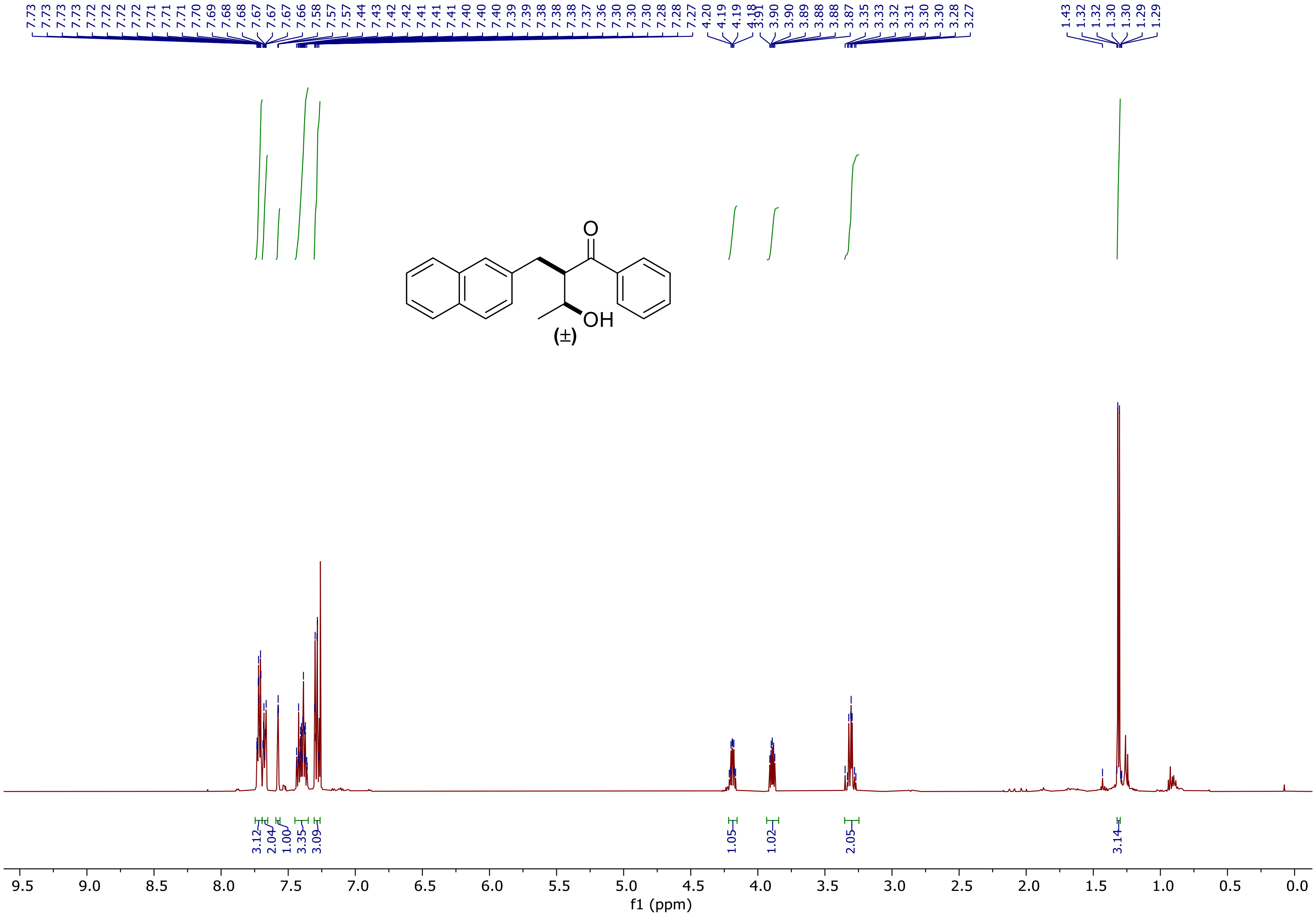

^{1}H NMR (500 MHz, $CDCl_3$) Spectrum of (2*RS*,3*RS*)-3-hydroxy-2-(naphthalen-2-ylmethyl)-1-phenyl-1-butanone **3t**.

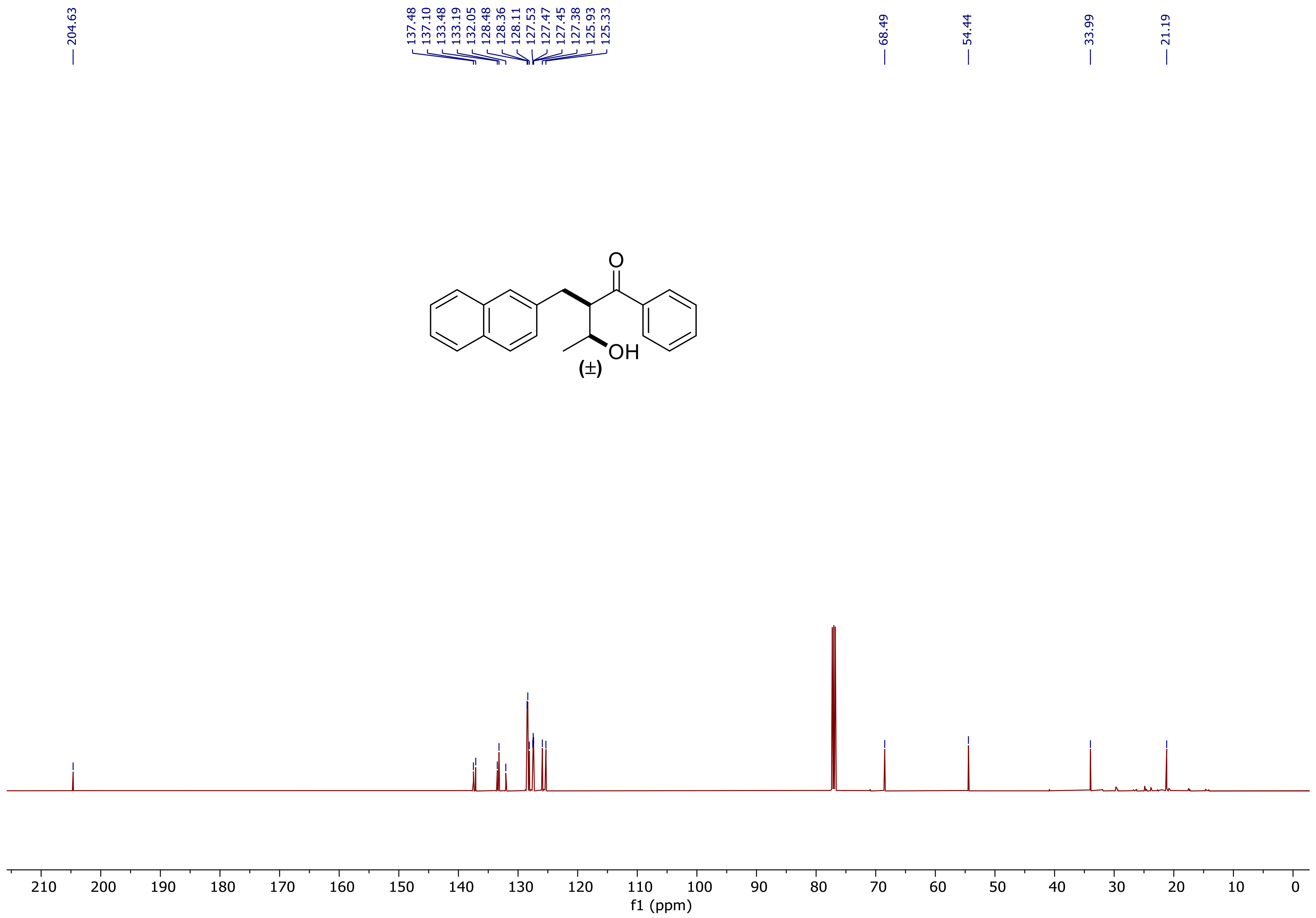

^{13}C NMR (126 MHz, $CDCl_3$) Spectrum of (2*RS*,3*RS*)-3-hydroxy-2-(naphthalen-2-ylmethyl)-1-phenyl-1-butanone **3t**.

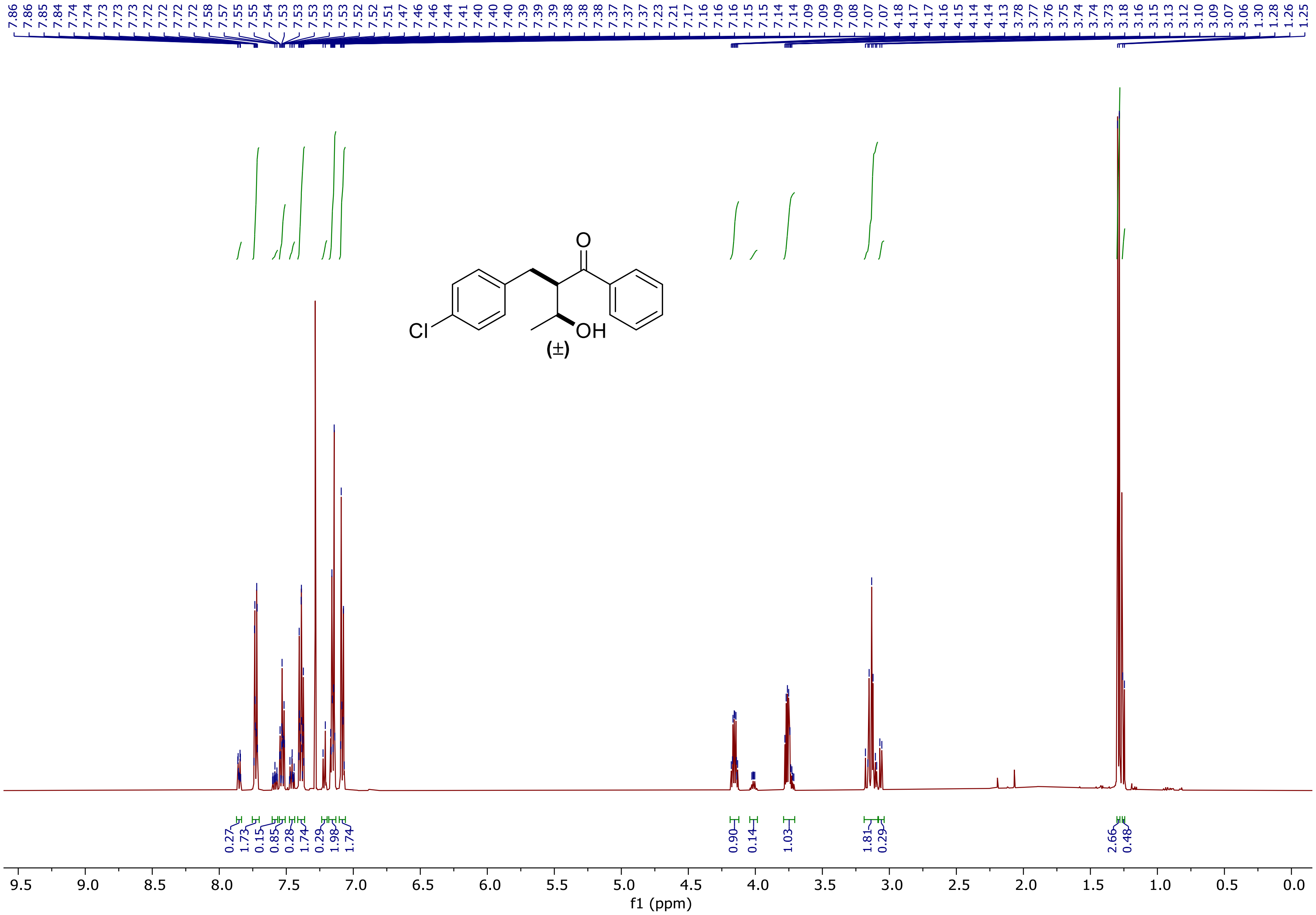

¹H NMR (500 MHz, $CDCl_3$) Spectrum of (2*RS*,3*RS*)-2-(4-chlorobenzyl)-3-hydroxy-1-phenyl-1-butanone **3u**.

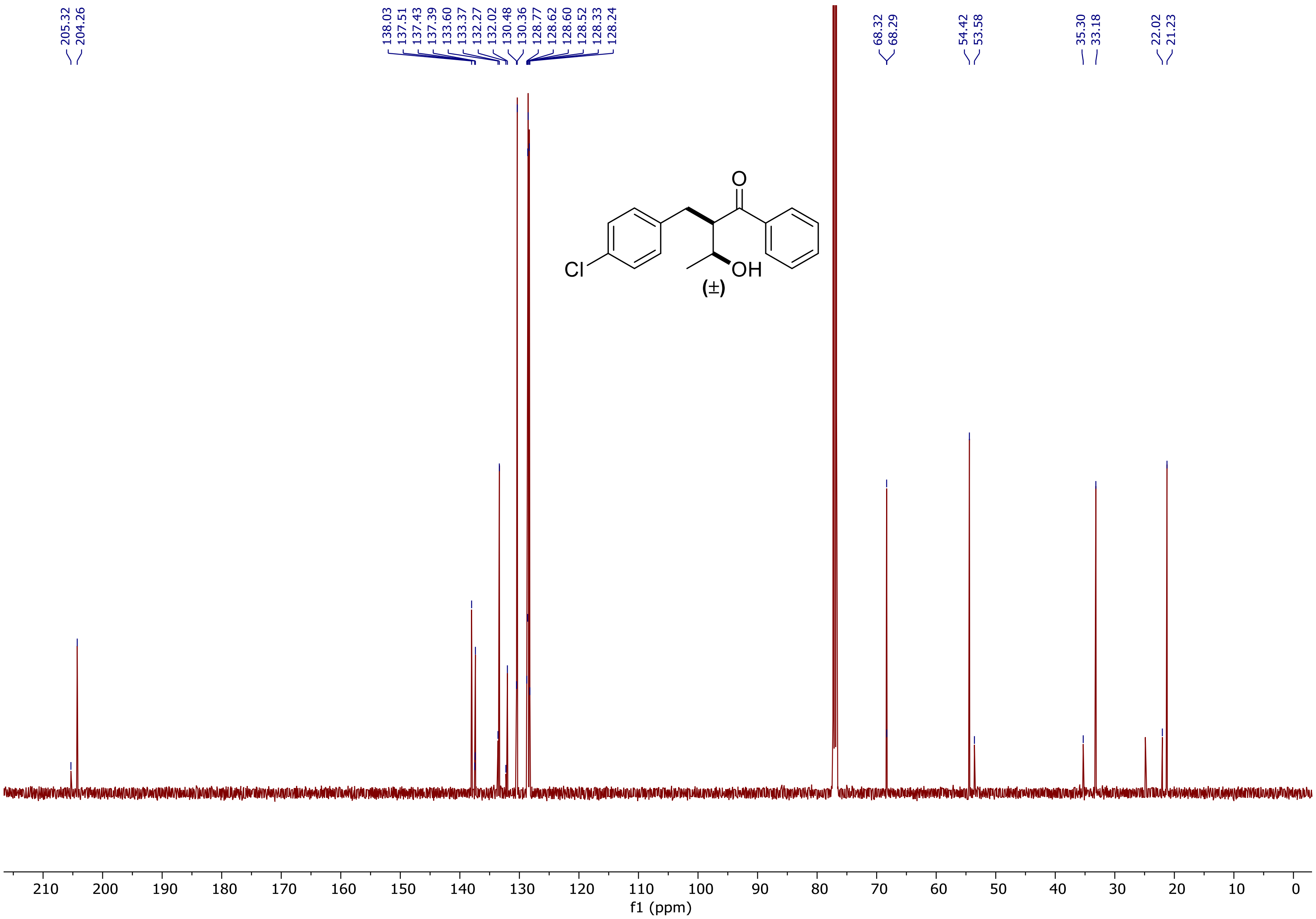

^{13}C NMR (126 MHz, $CDCl_3$) Spectrum of (2*RS*,3*RS*)-2-(4-chlorobenzyl)-3-hydroxy-1-phenyl-1-butanone **3u**.

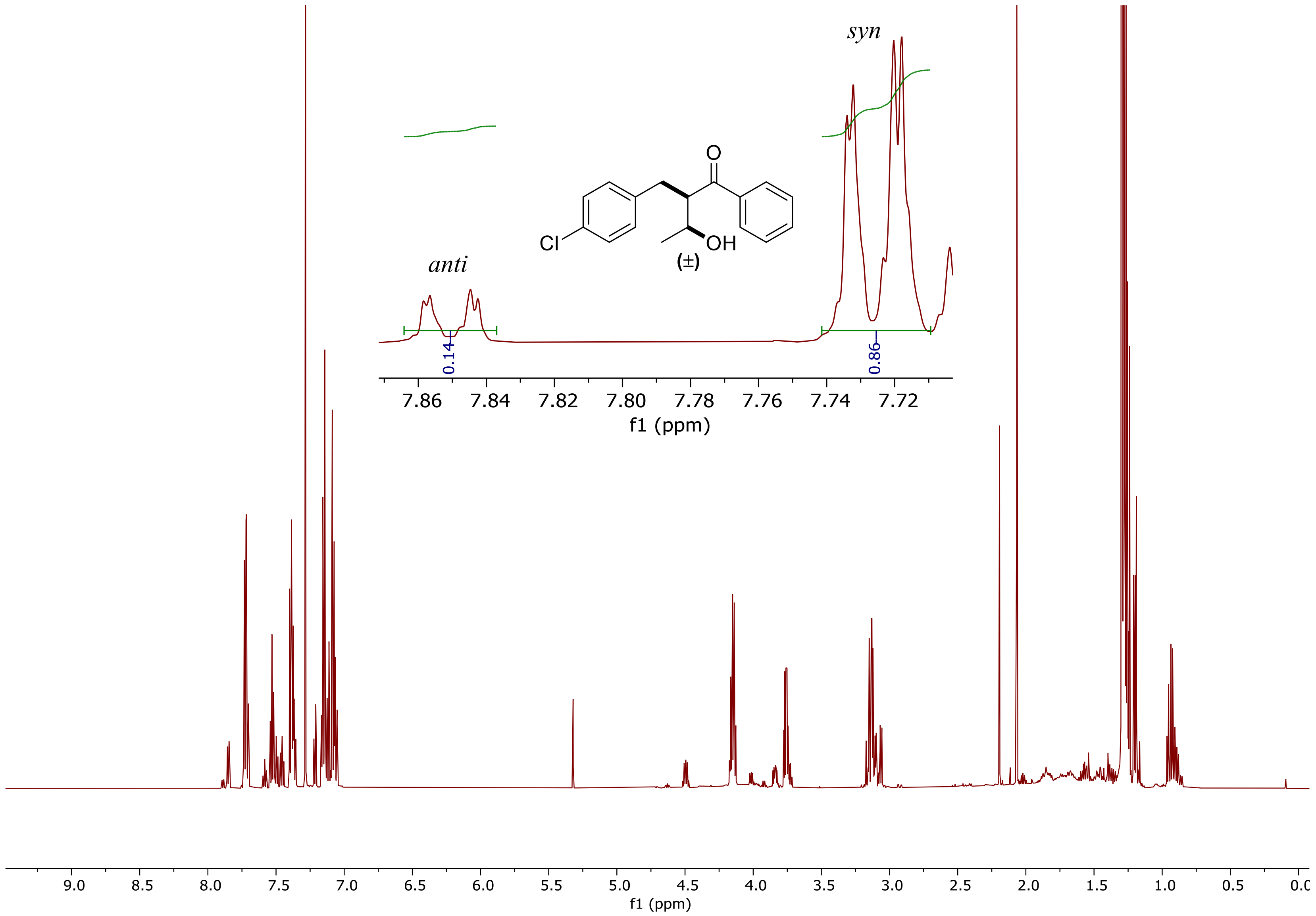

^{1}H NMR (400 MHz, $CDCl_3$) Spectrum of the crude reaction mixture for (2*RS*,3*RS*)-2-(4-chlorobenzyl)-3-hydroxy-1-phenyl-1-butanone **3u**.

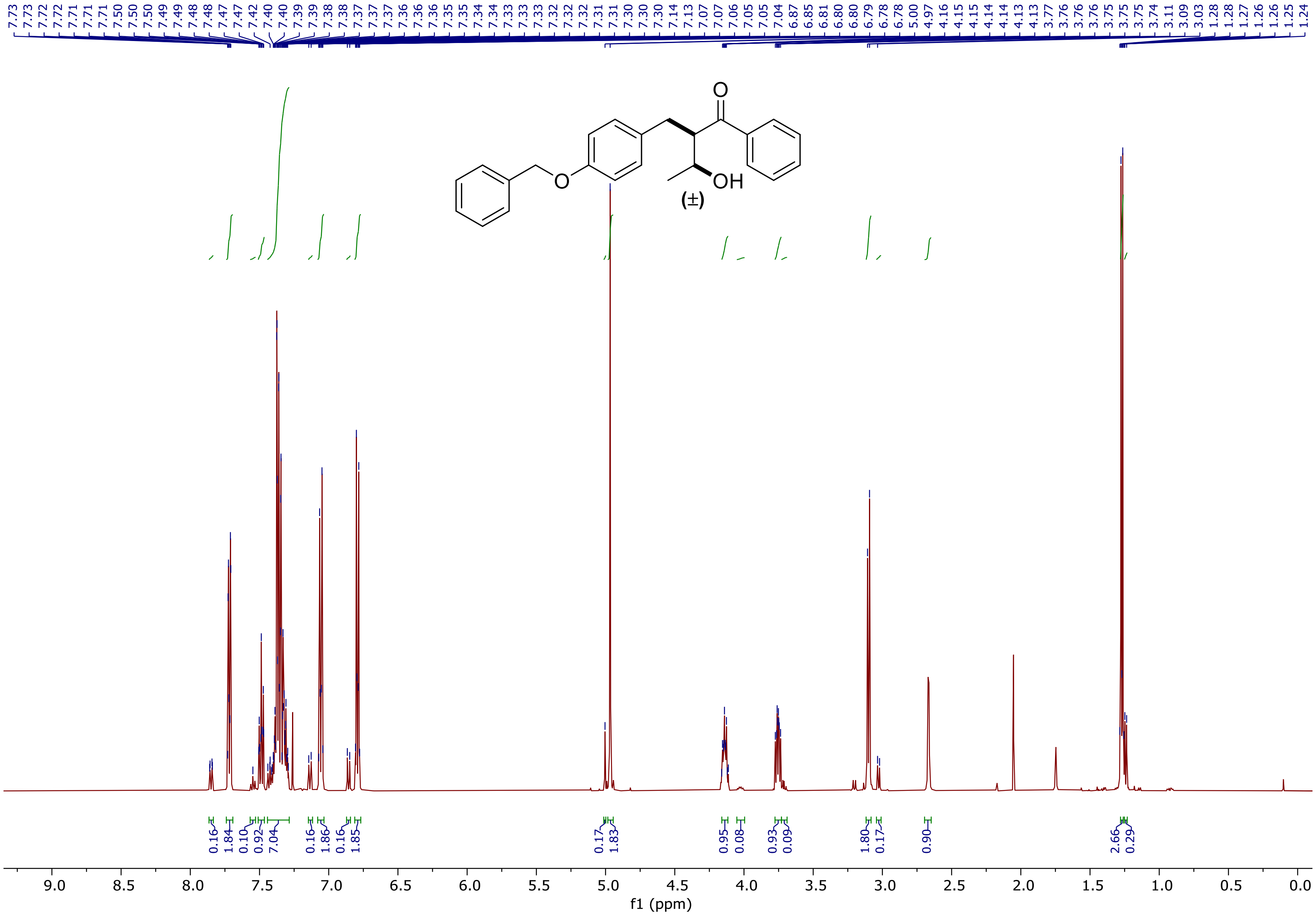

^{1}H NMR (500 MHz, $CDCl_3$) Spectrum of (2*RS*,3*RS*)-2-(4-(benzyloxy)benzyl)-3-hydroxy-1-phenyl-1-butanone **3v**.

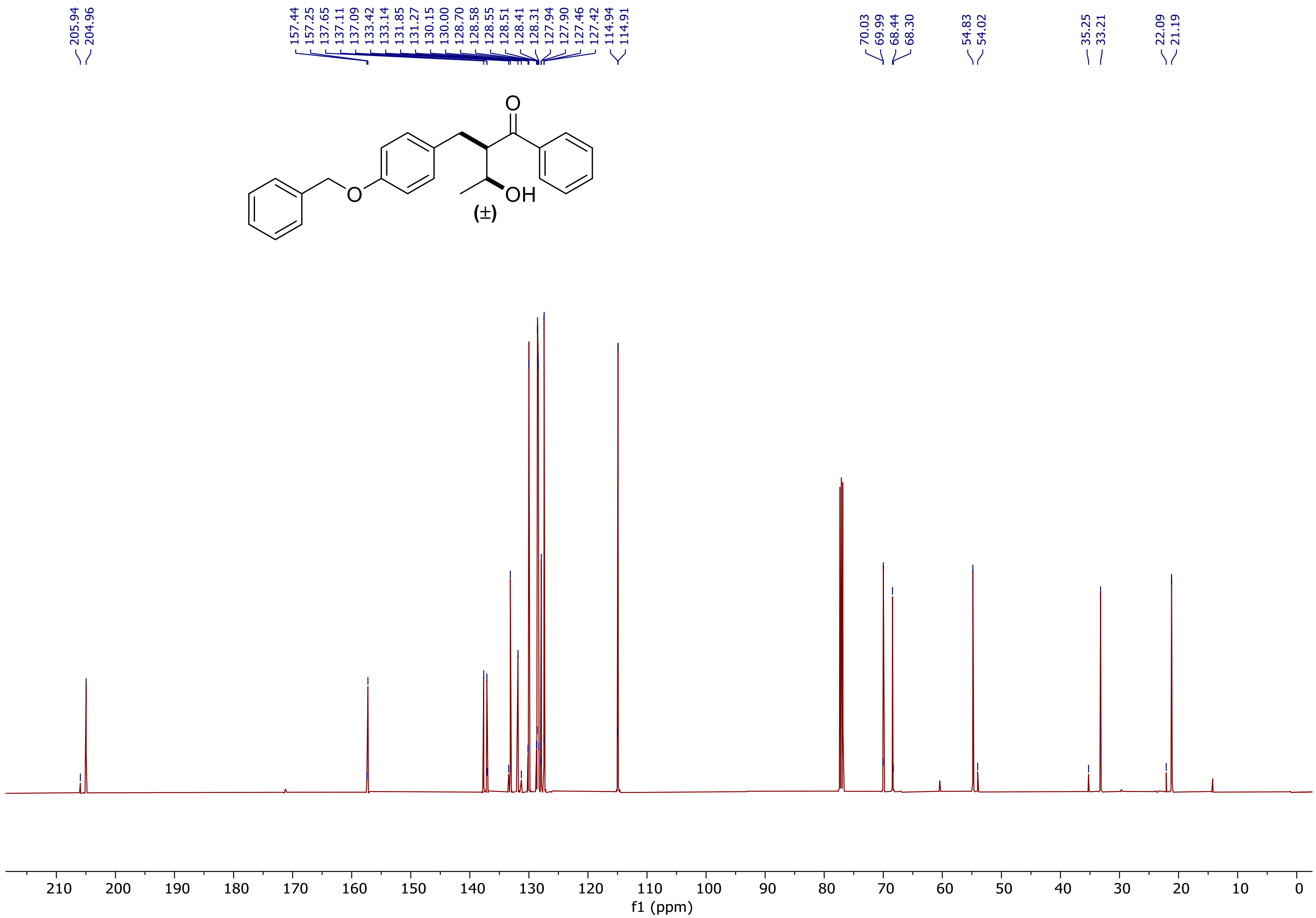

13C NMR (126 MHz, $CDCl_3$) Spectrum of (2*RS*,3*RS*)-2-(4-(benzyloxy)benzyl)-3-hydroxy-1-phenyl-1-butanone **3v**.

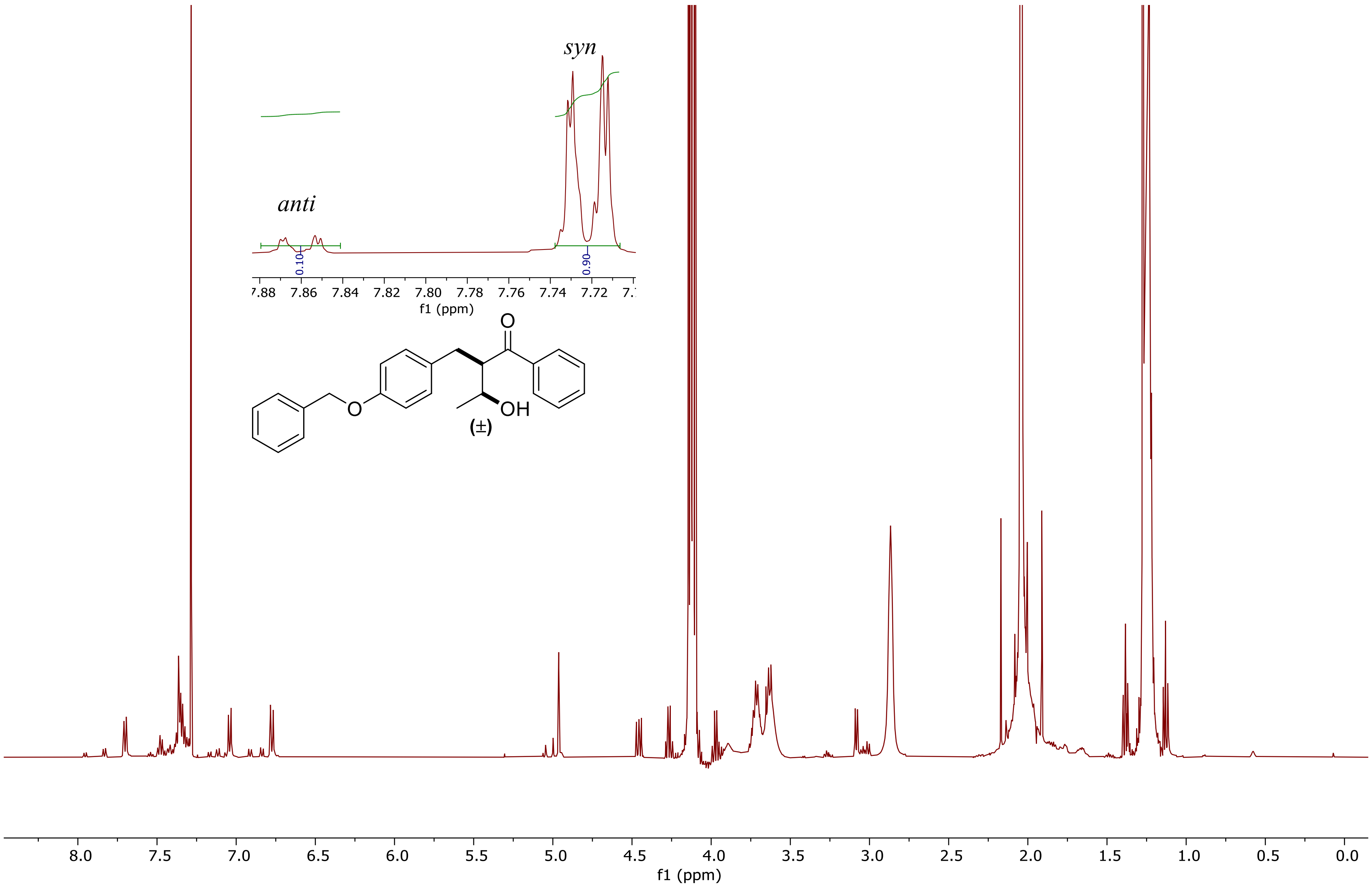

^{1}H NMR (400 MHz, $CDCl_3$) Spectrum of the crude reaction mixture for (2*RS*,3*RS*)-2-(4-(benzyloxy)benzyl)-3-hydroxy-1-phenyl-1-butanone **3v**.

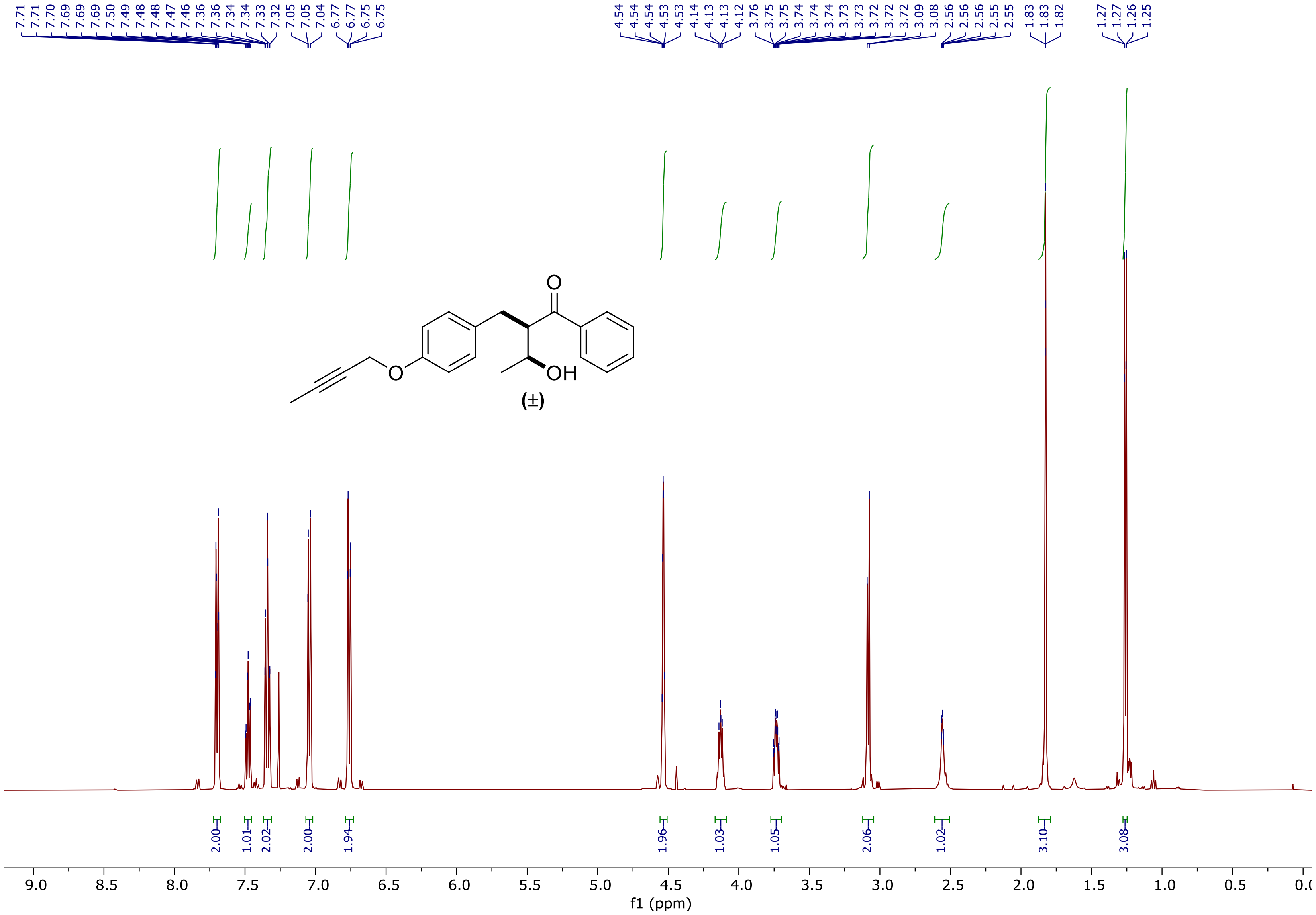

^{1}H NMR (500 MHz, $CDCl_3$) Spectrum of (2*RS*,3*RS*)-2-(4-(2-butynyloxy)benzyl)-3-hydroxy-1-phenyl-1-butanone **3w**.

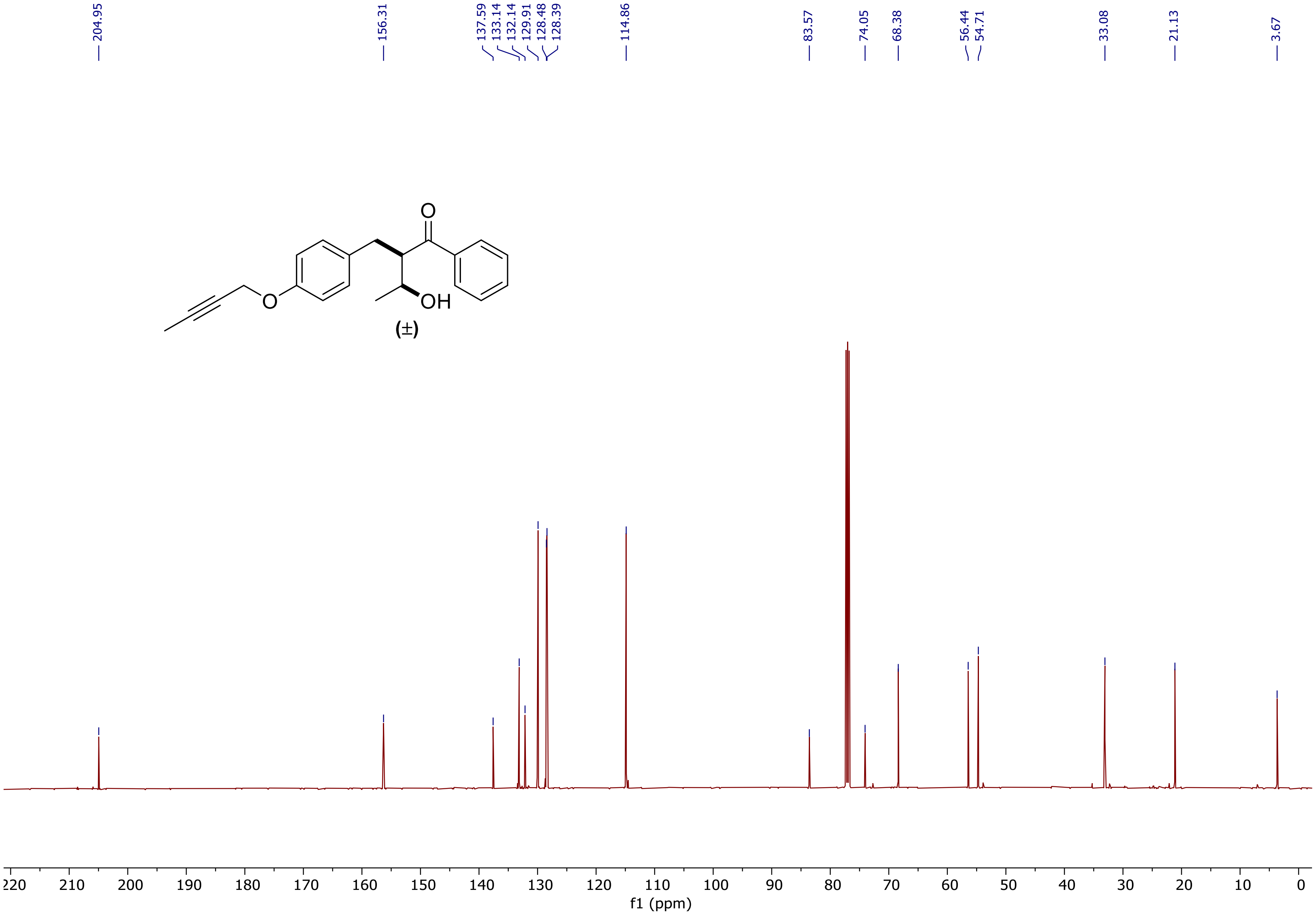

^{13}C NMR (126 MHz, $CDCl_3$) Spectrum of (2*RS*,3*RS*)-2-(4-(2-butynyloxy)benzyl)-3-hydroxy-1-phenyl-1-butanone **3w**.

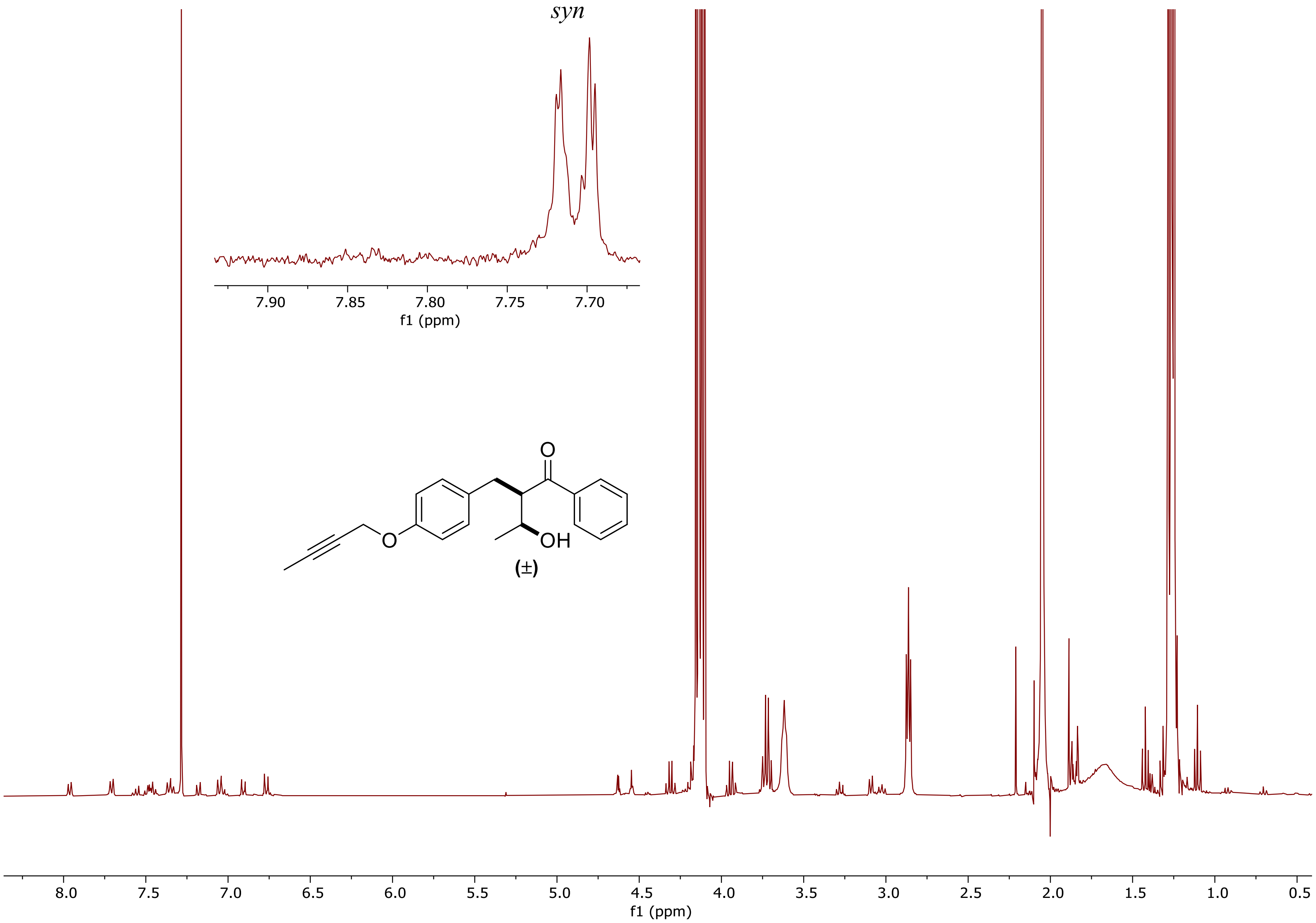

^{1}H NMR (400 MHz, $CDCl_3$) Spectrum of the crude reaction mixture for (2*RS*,3*RS*)-2-(4-(2-butynyloxy)benzyl)-3-hydroxy-1-phenyl-1-butanone **3w**.

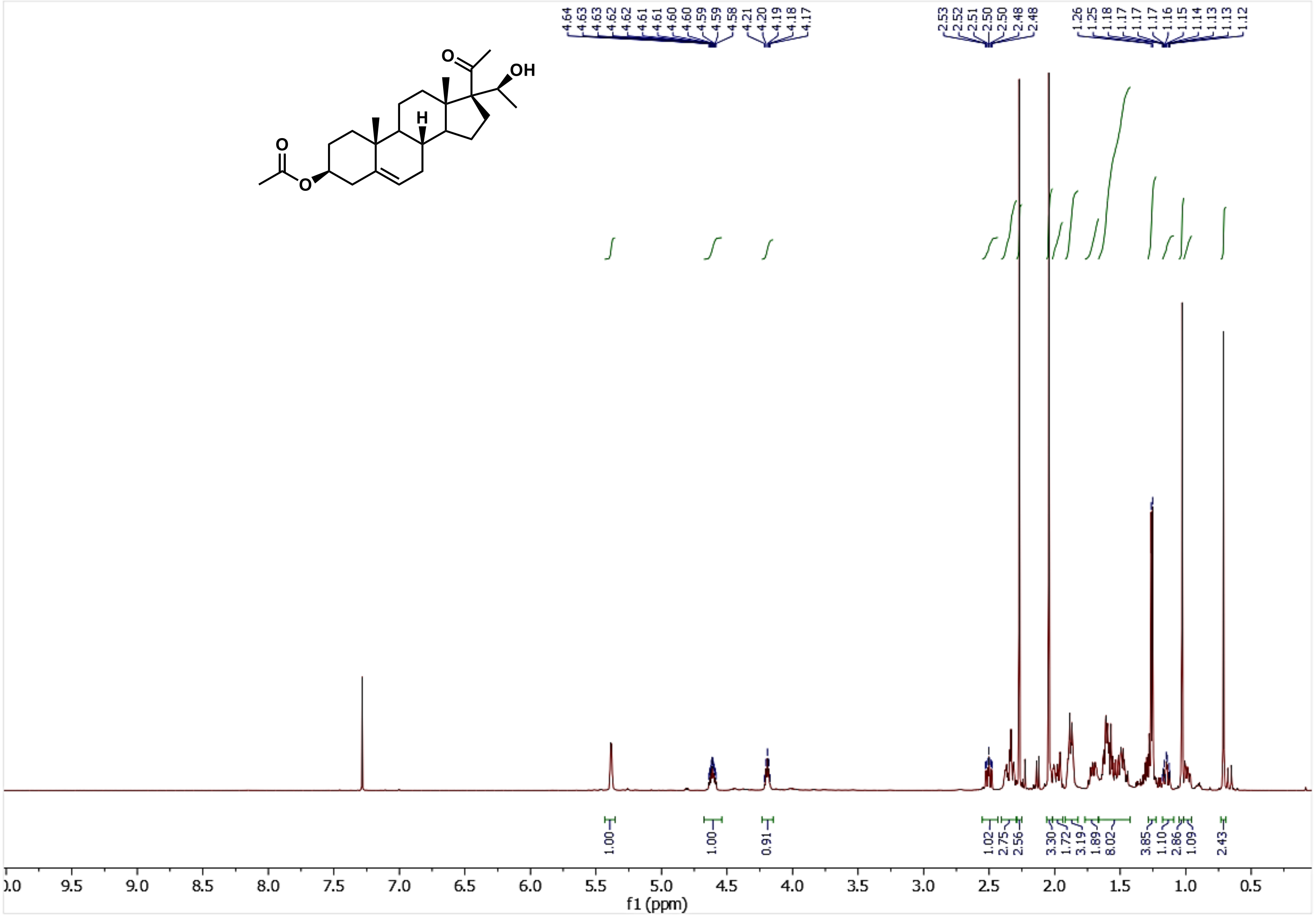

^{1}H NMR (600 MHz, $CDCl_3$) Spectrum of 3β-acetoxy-17 α-(ethan-1-ol)-5-pregnen-2-one, **3x**

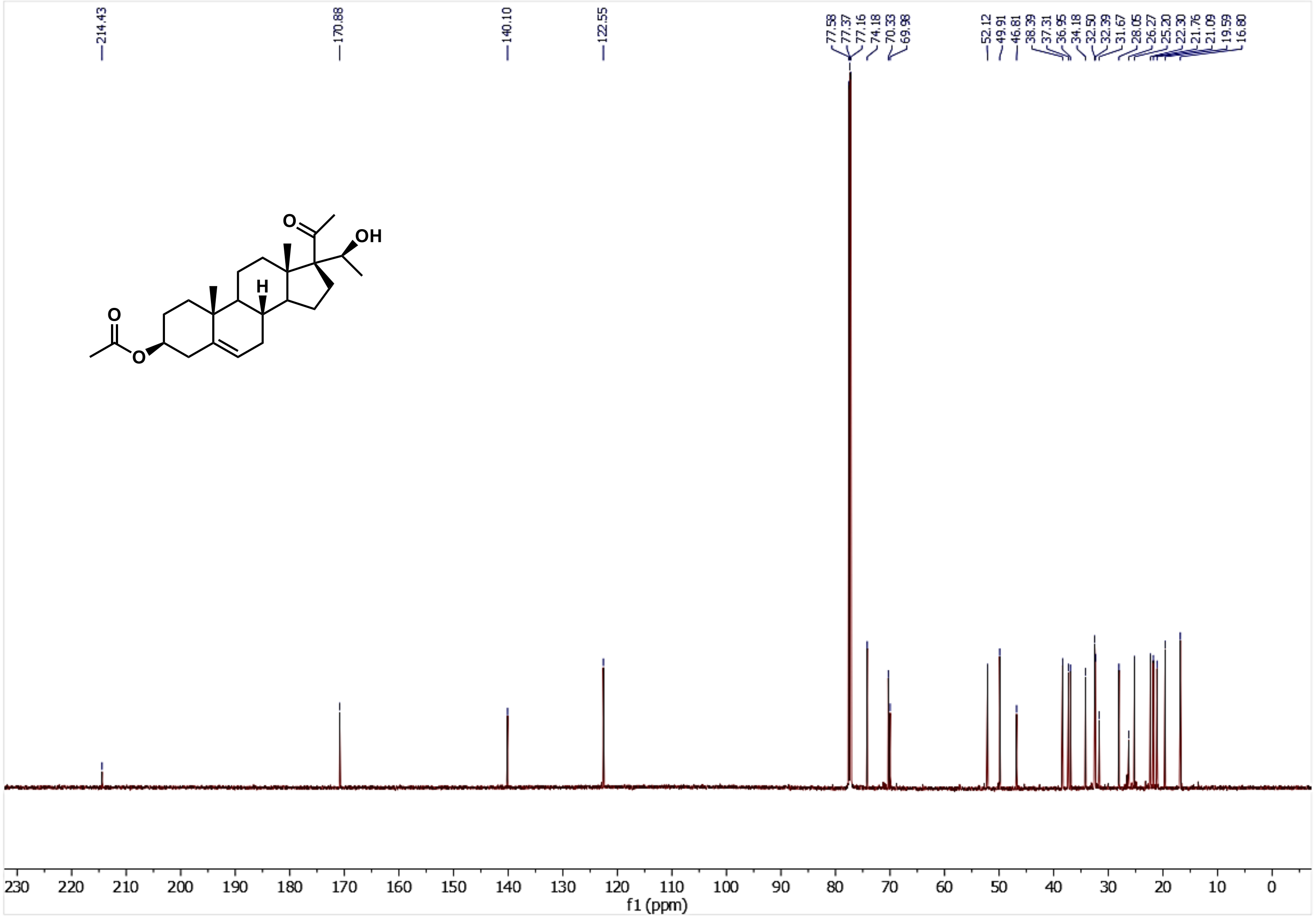

^{13}C NMR (151 MHz, $CDCl_3$) Spectrum 3β-acetoxy-17 α-(ethan-1-ol)-5-pregnen-2-one, **3x**

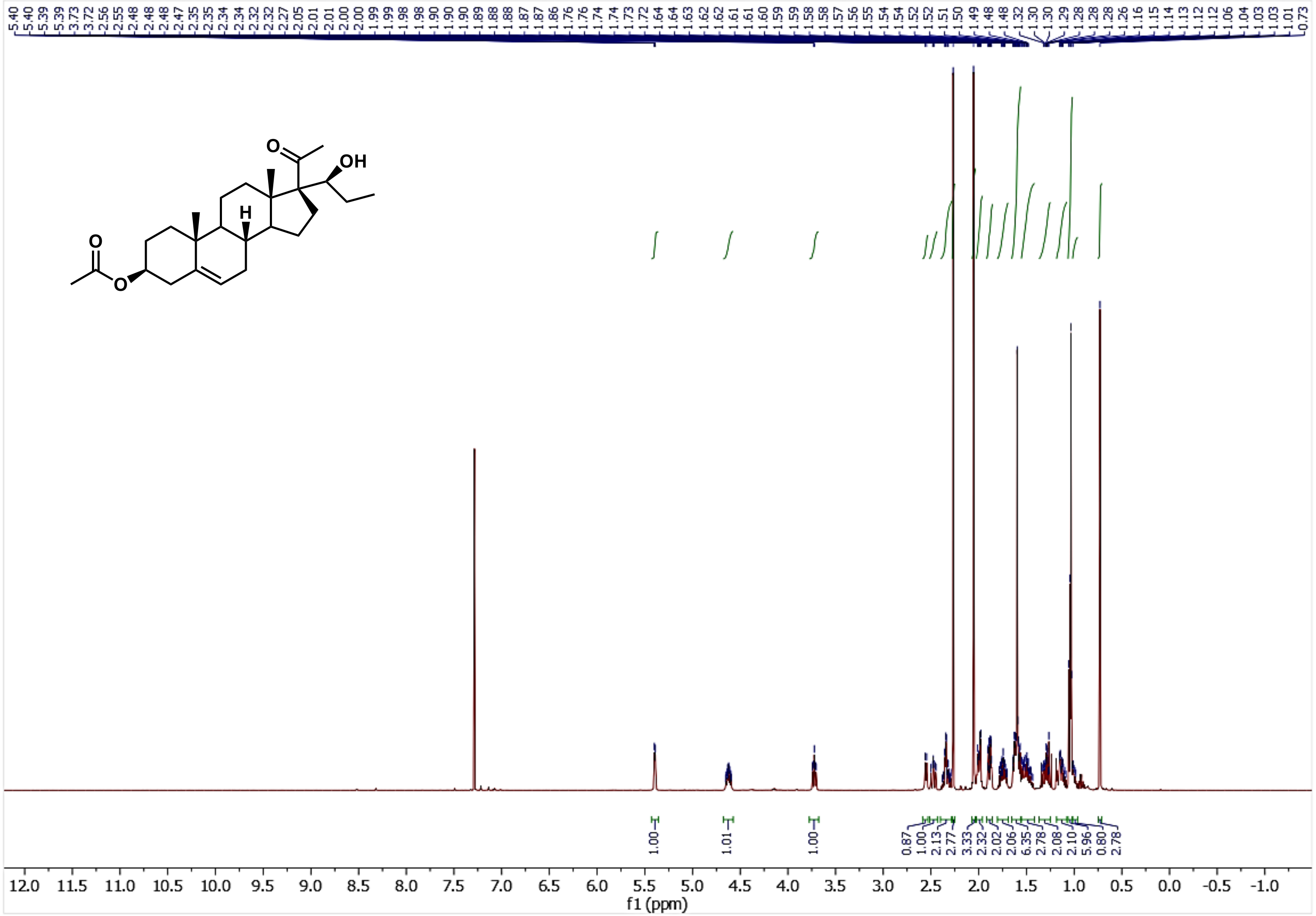

^{1}H NMR (600 MHz, $CDCl_3$) Spectrum of 3β-acetoxy-17 α-(propan-1-ol)-5-pregnen-2-one, **3y**

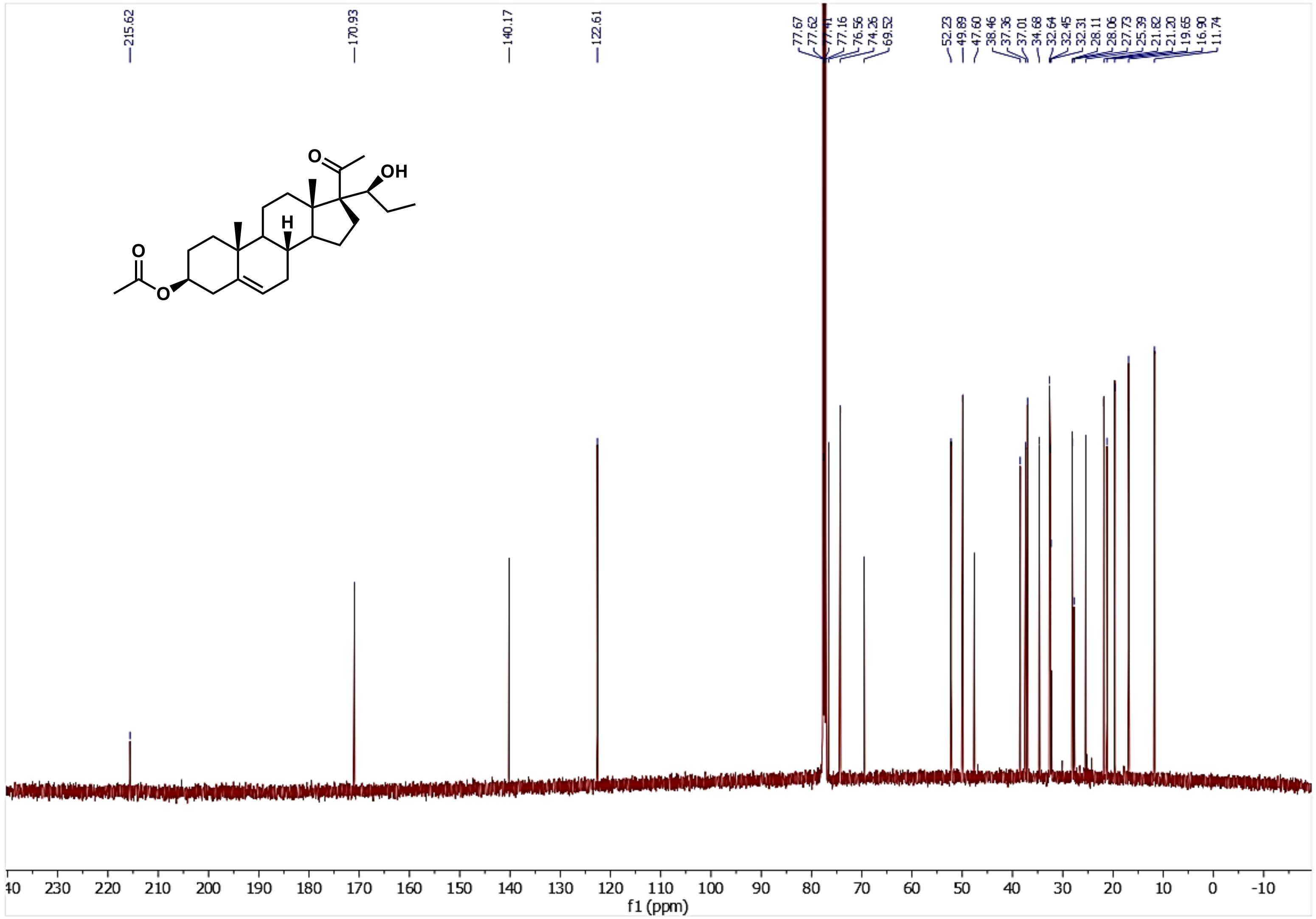

^{13}C NMR (126 MHz, $CDCl_3$) Spectrum of 3β-acetoxy-17 α-(propan-1-ol)-5-pregnen-2-one, **3y**

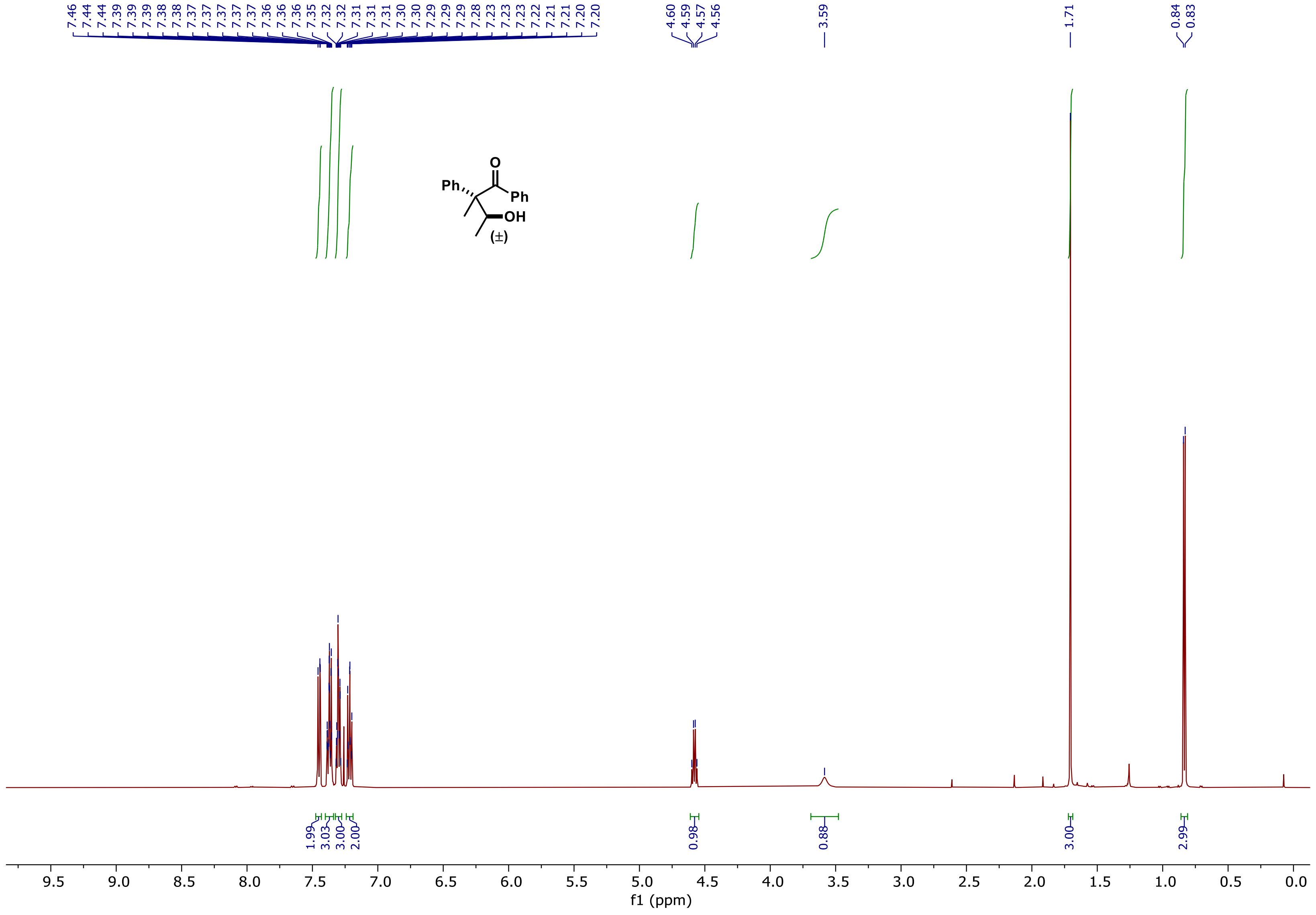

^{1}H NMR (500 MHz, $CDCl_3$) Spectrum of (*2RS,3RS*)-3-hydroxy-2-methyl-1,2-diphenyl-1-butanone **3z**.

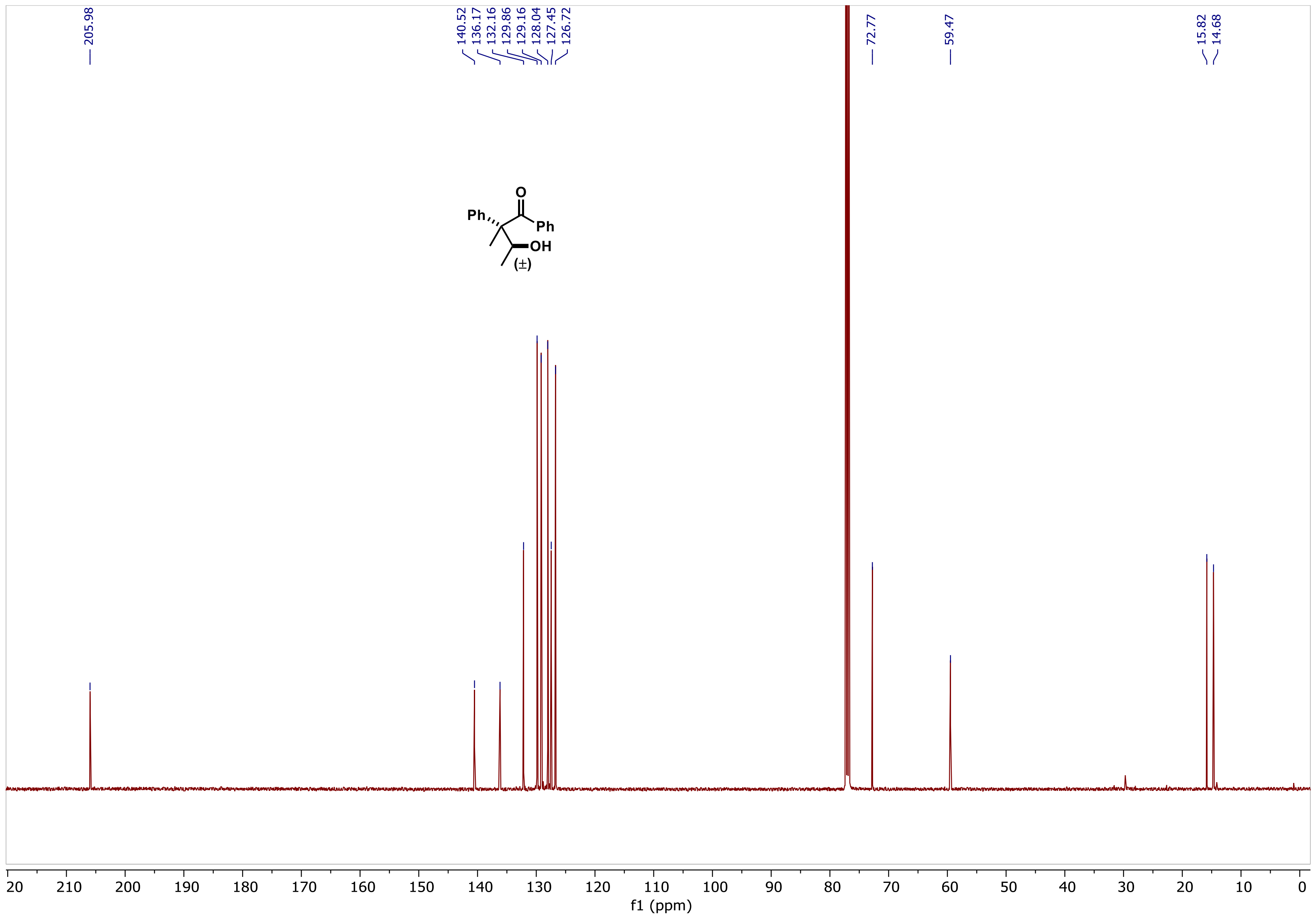

^{13}C NMR (126 MHz, $CDCl_3$) Spectrum of (*2RS,3RS*)-3-hydroxy-2-methyl-1,2-diphenyl-1-butanone **3z**.

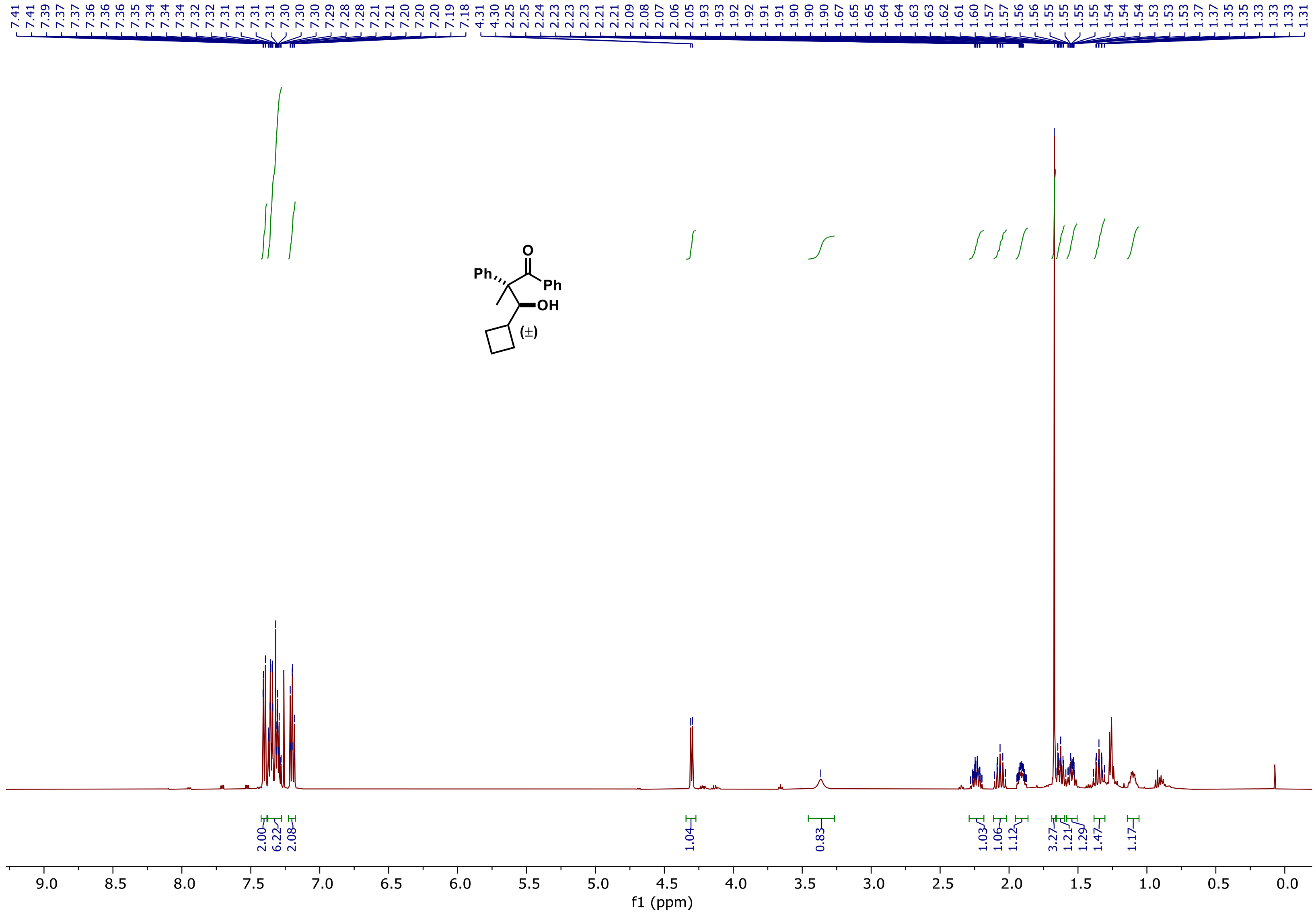

[1]**H NMR** (500 MHz, $CDCl_3$) Spectrum of (*2RS,3RS*)-3-cyclobutyl-3-hydroxy-2-methyl-1,2-diphenyl-1-propanone, **3aa**

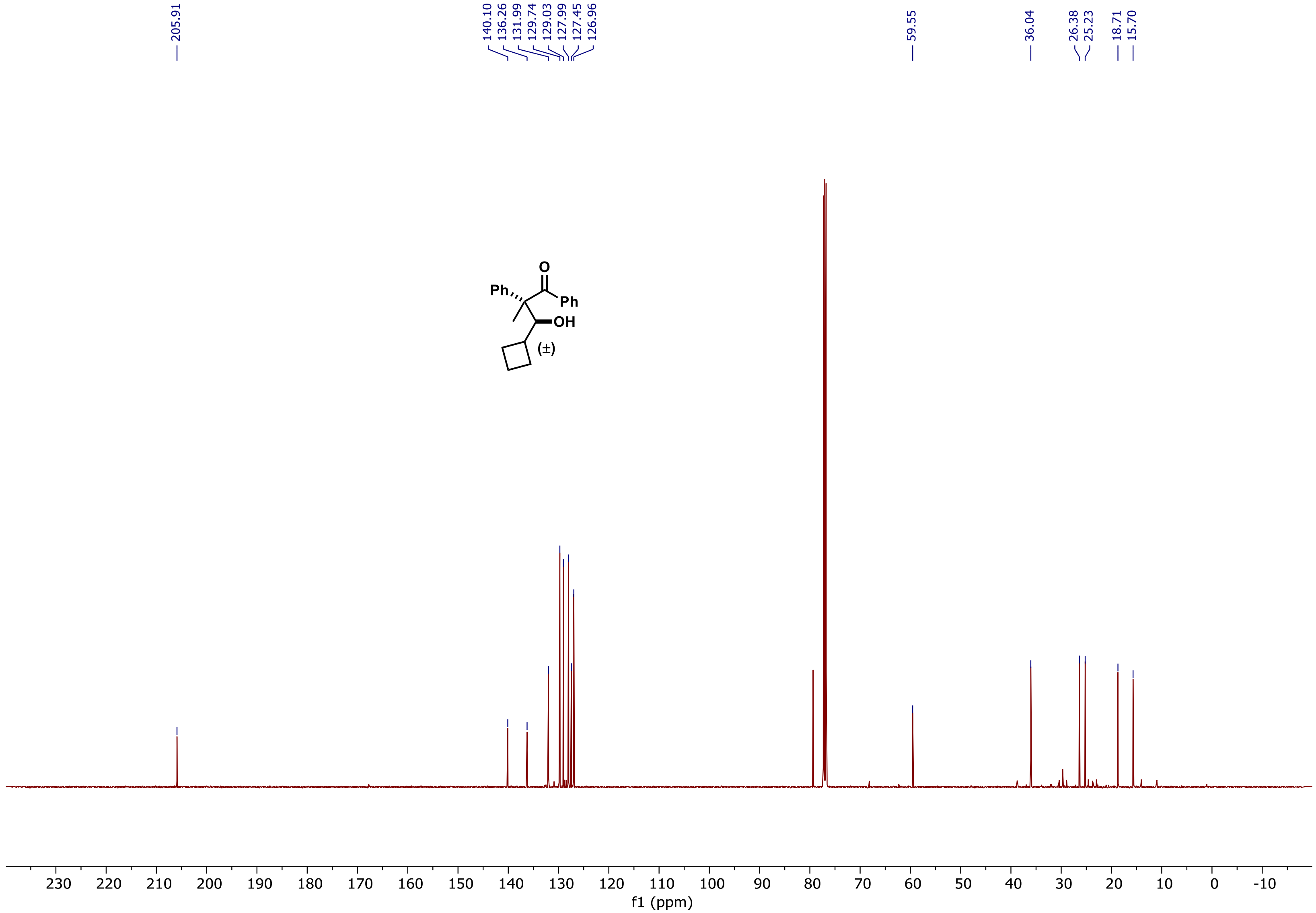

^{13}C NMR (126 MHz, $CDCl_3$) Spectrum of (*2RS,3RS*)-3-cyclobutyl-3-hydroxy-2-methyl-1,2-diphenyl-1-propanone, **3aa**

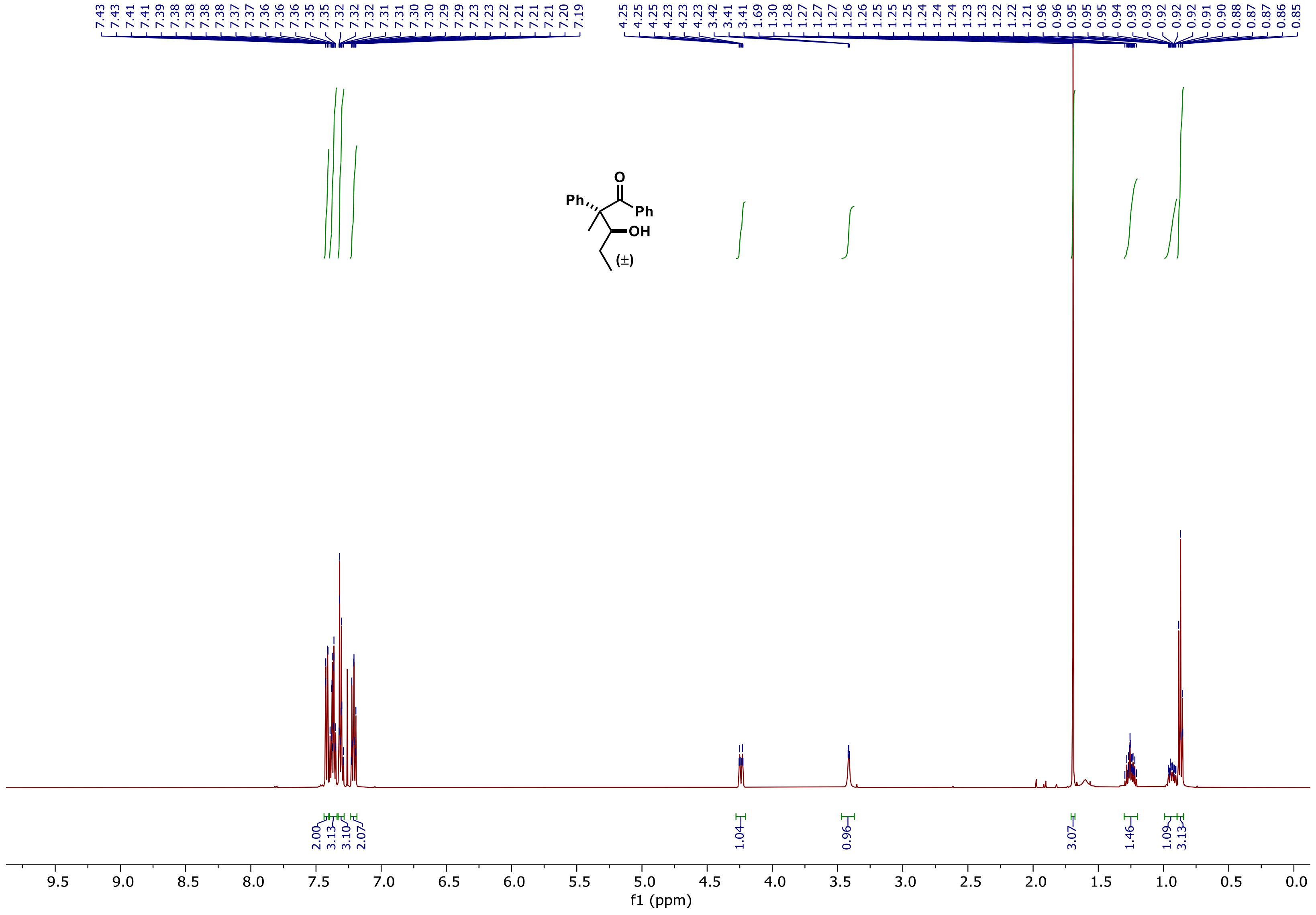

^{1}H NMR (500 MHz, $CDCl_3$) Spectrum of (*2RS,3RS*)-3-hydroxy-2-methyl-1,2-diphenyl-1-pentanone, **3ab**

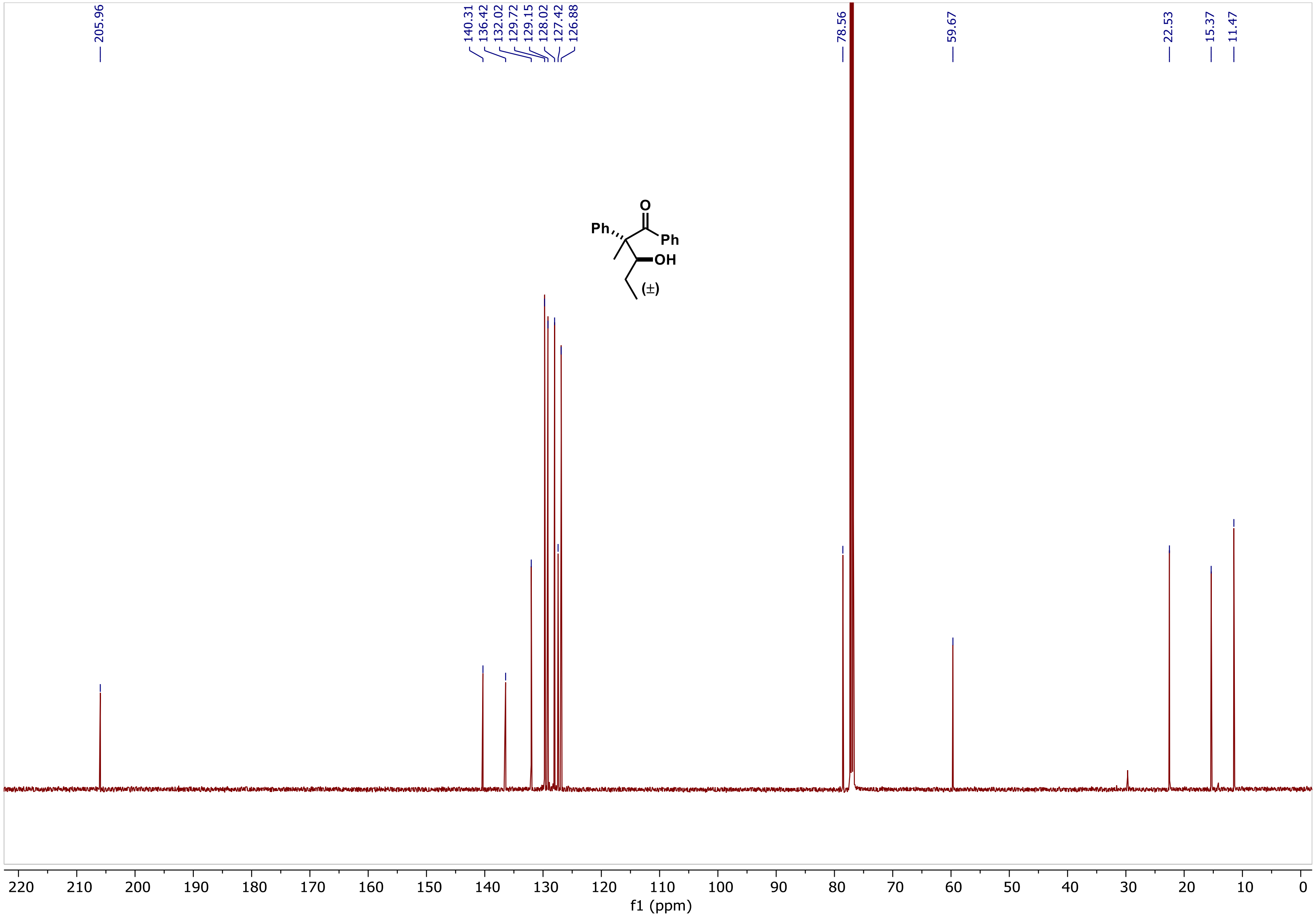

^{13}C NMR (126 MHz, $CDCl_3$) Spectrum of (*2RS,3RS*)-3-hydroxy-2-methyl-1,2-diphenyl-1-pentanone, **3ab**

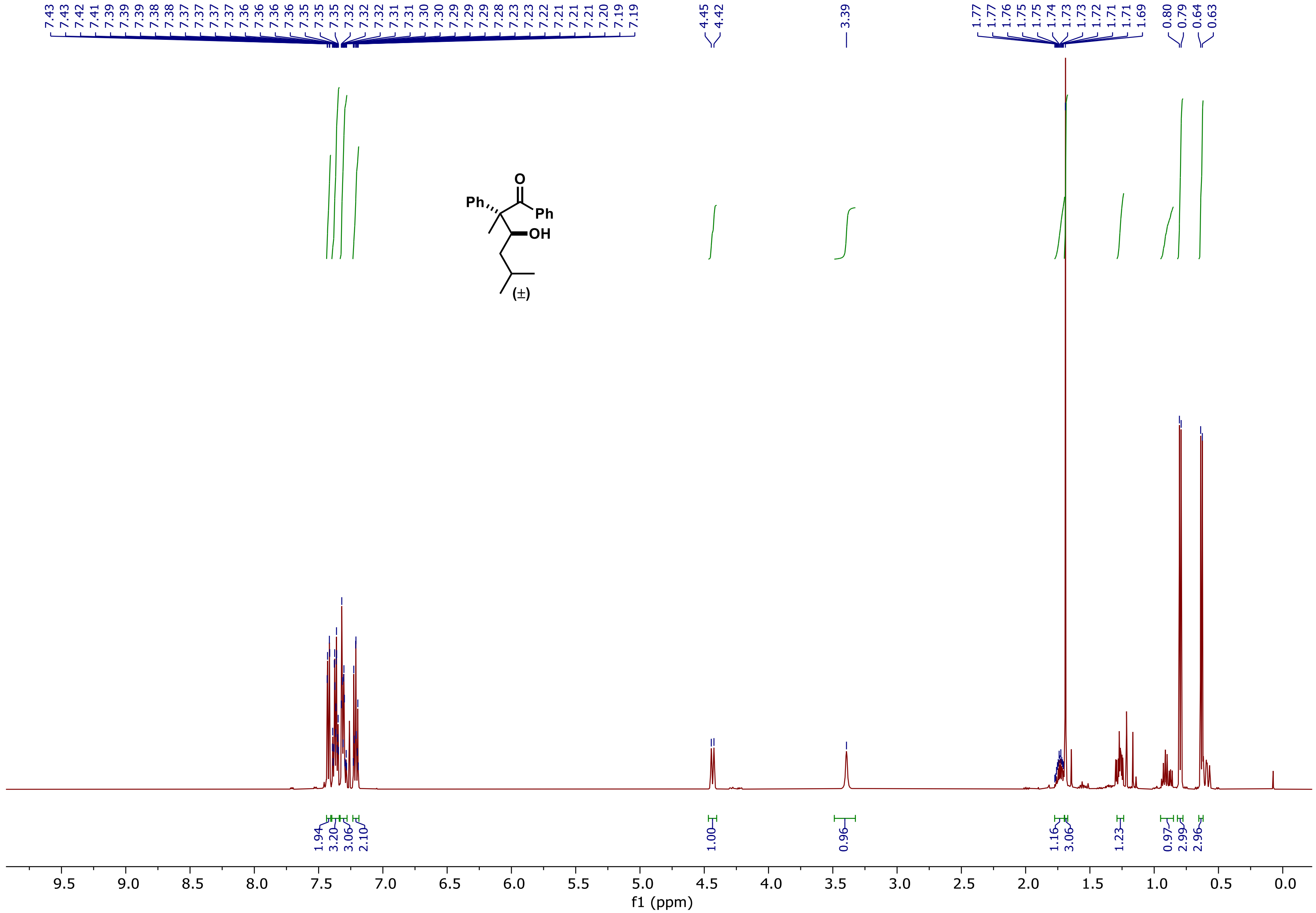

^{1}H NMR (500 MHz, $CDCl_3$) Spectrum of (*2RS,3RS*)-3-hydroxy-2,5-dimethyl-1,2-diphenyl-1-hexanone, **3ac**

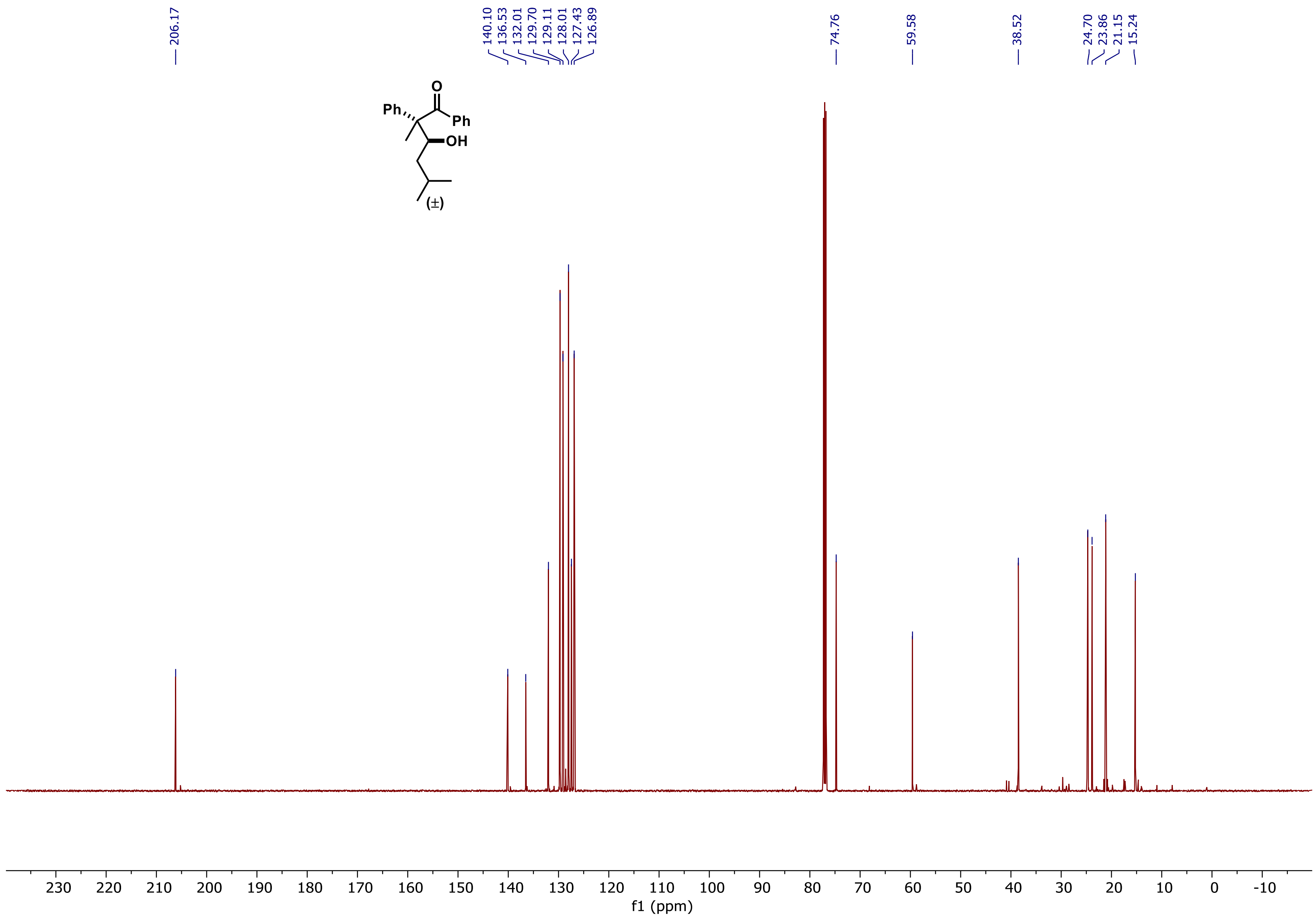

^{13}C NMR (126 MHz, $CDCl_3$) Spectrum of (*2RS,3RS*)-3-hydroxy-2,5-dimethyl-1,2-diphenyl-1-hexanone, **3ac**

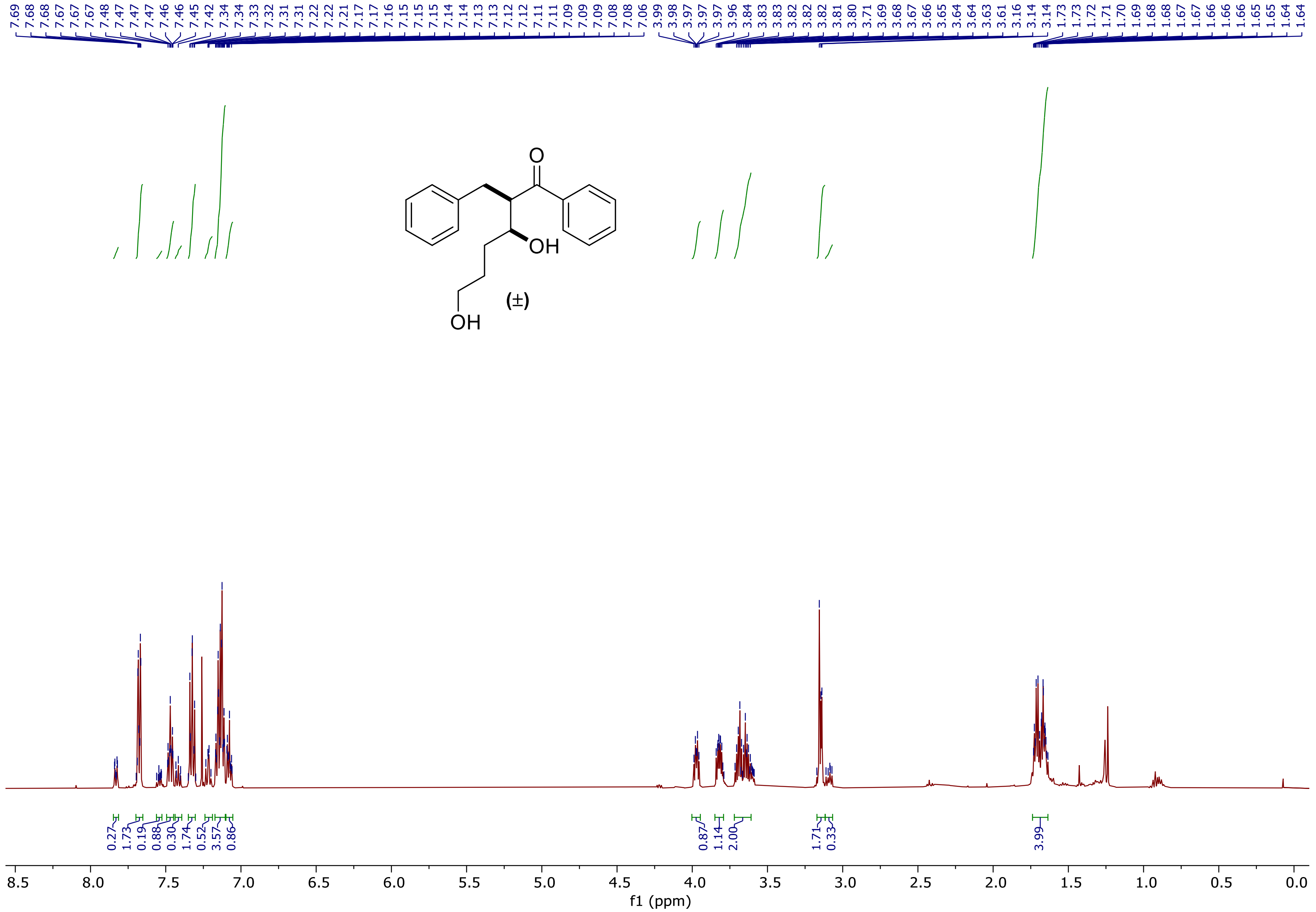

¹H NMR (500 MHz, $CDCl_3$) Spectrum of (2*RS*,3*RS*)-2-benzyl-3,6-dihydroxy-1-phenyl-1-hexanone **4a**.

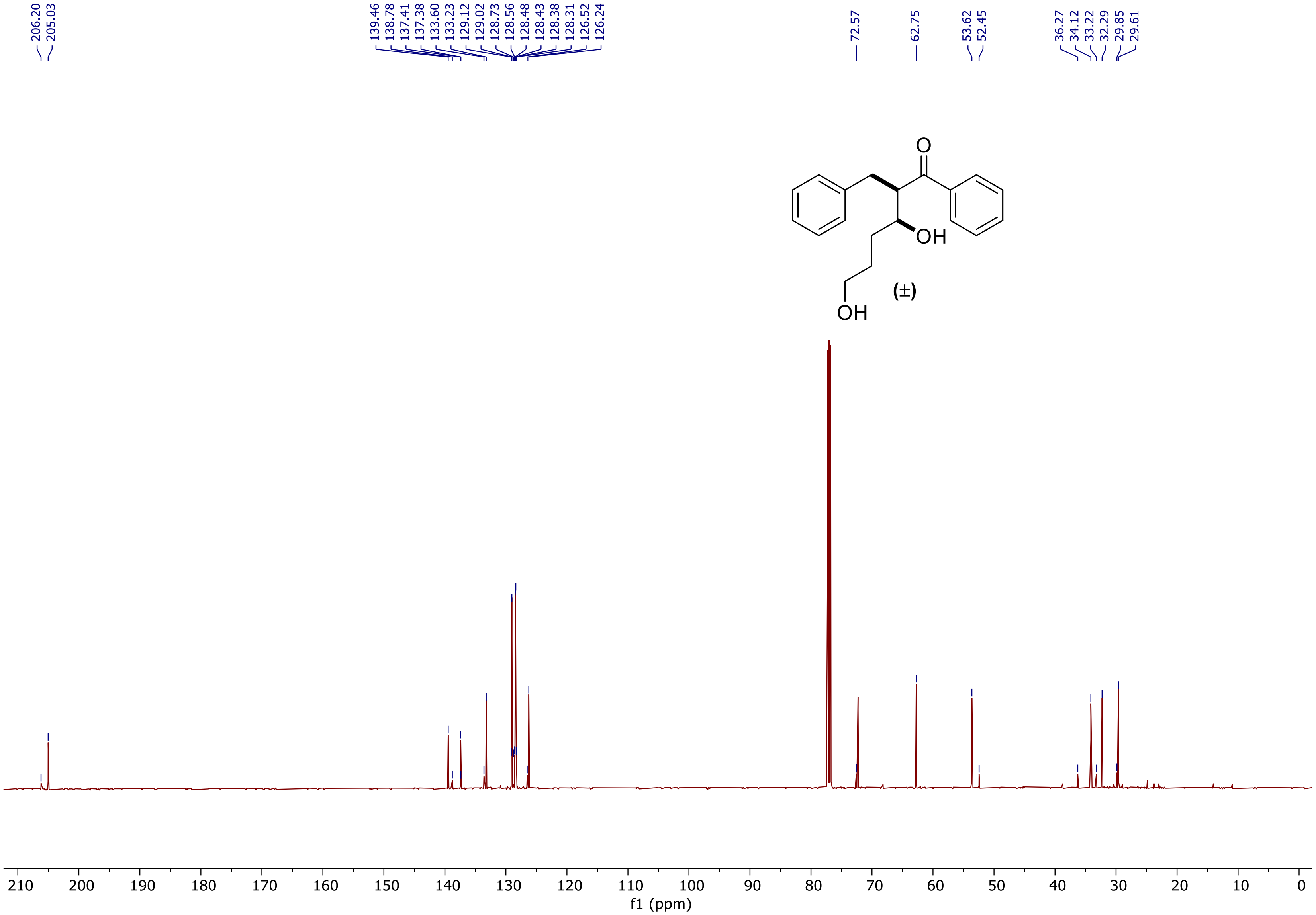

^{13}C NMR (126 MHz, $CDCl_3$) Spectrum of (2*RS*,3*RS*)-2-benzyl-3,6-dihydroxy-1-phenyl-1-hexanone **4a**.

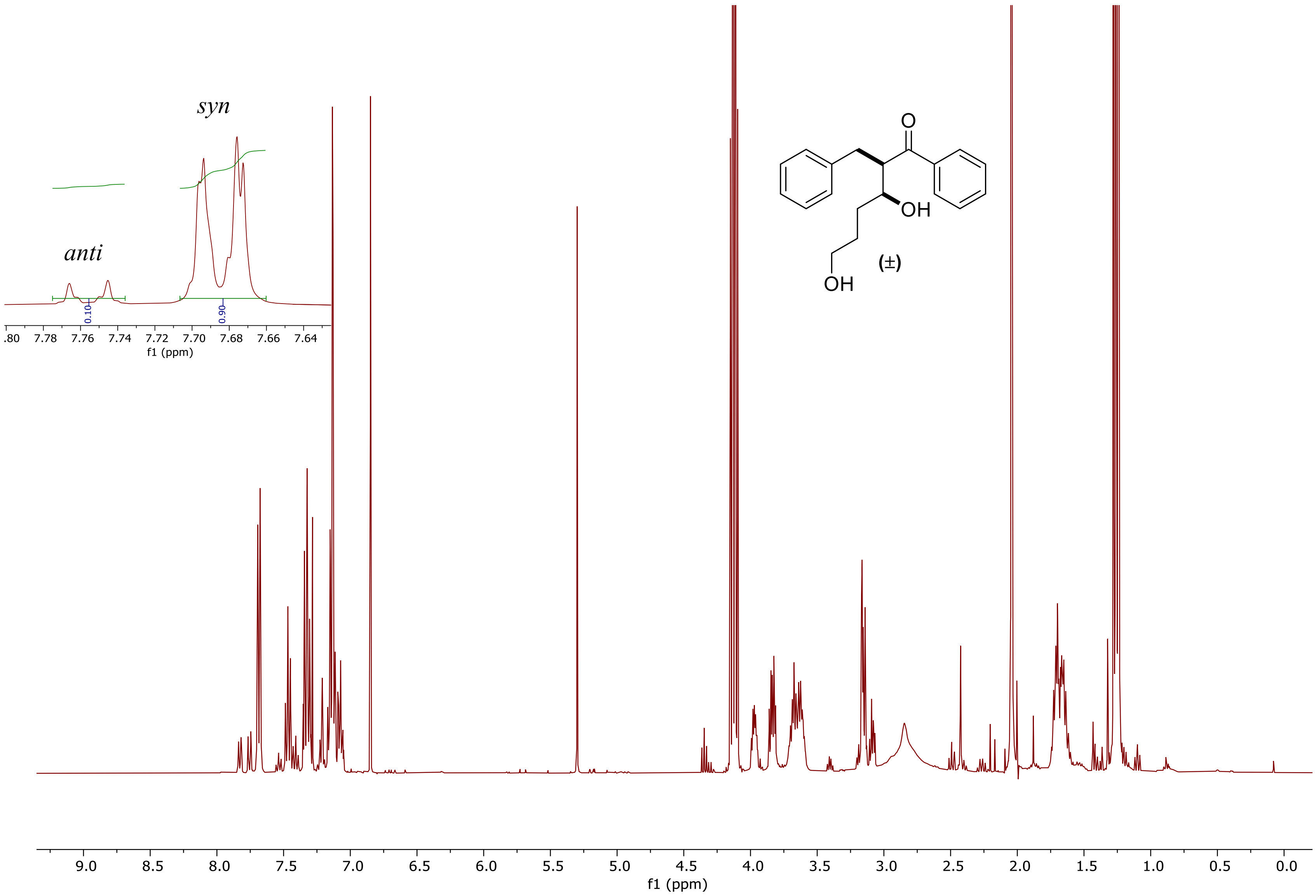

[1]**H NMR** (400 MHz, $CDCl_3$) Spectrum of the crude reaction mixture for (2*RS*,3*RS*)-2-benzyl-3,6-dihydroxy-1-phenyl-1-hexanone **4a**.

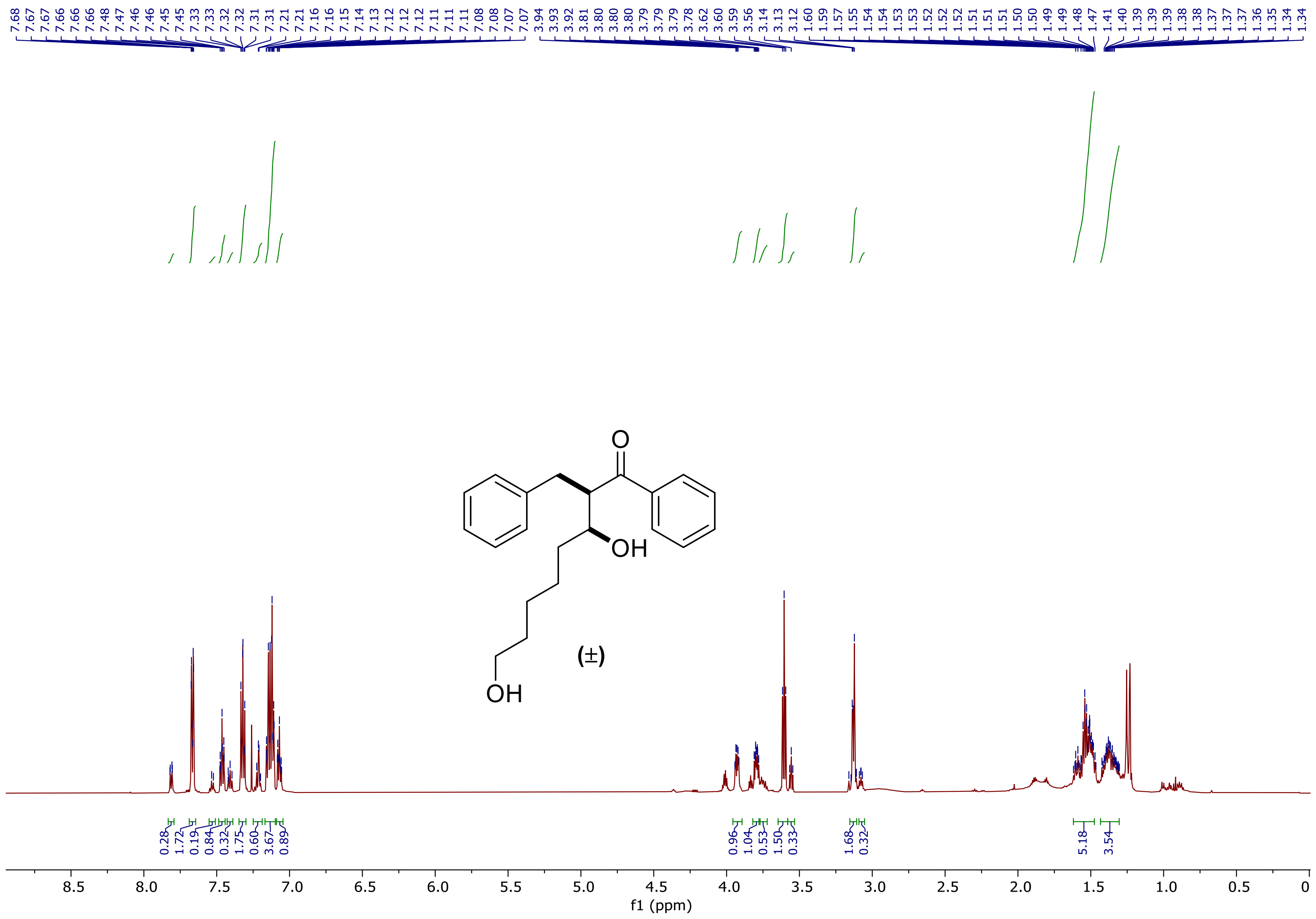

^{1}H NMR (500 MHz, $CDCl_3$) Spectrum of (2*RS*,3*RS*)-2-benzyl-3,8-dihydroxy-1-phenyl-1-octanone **4b**.

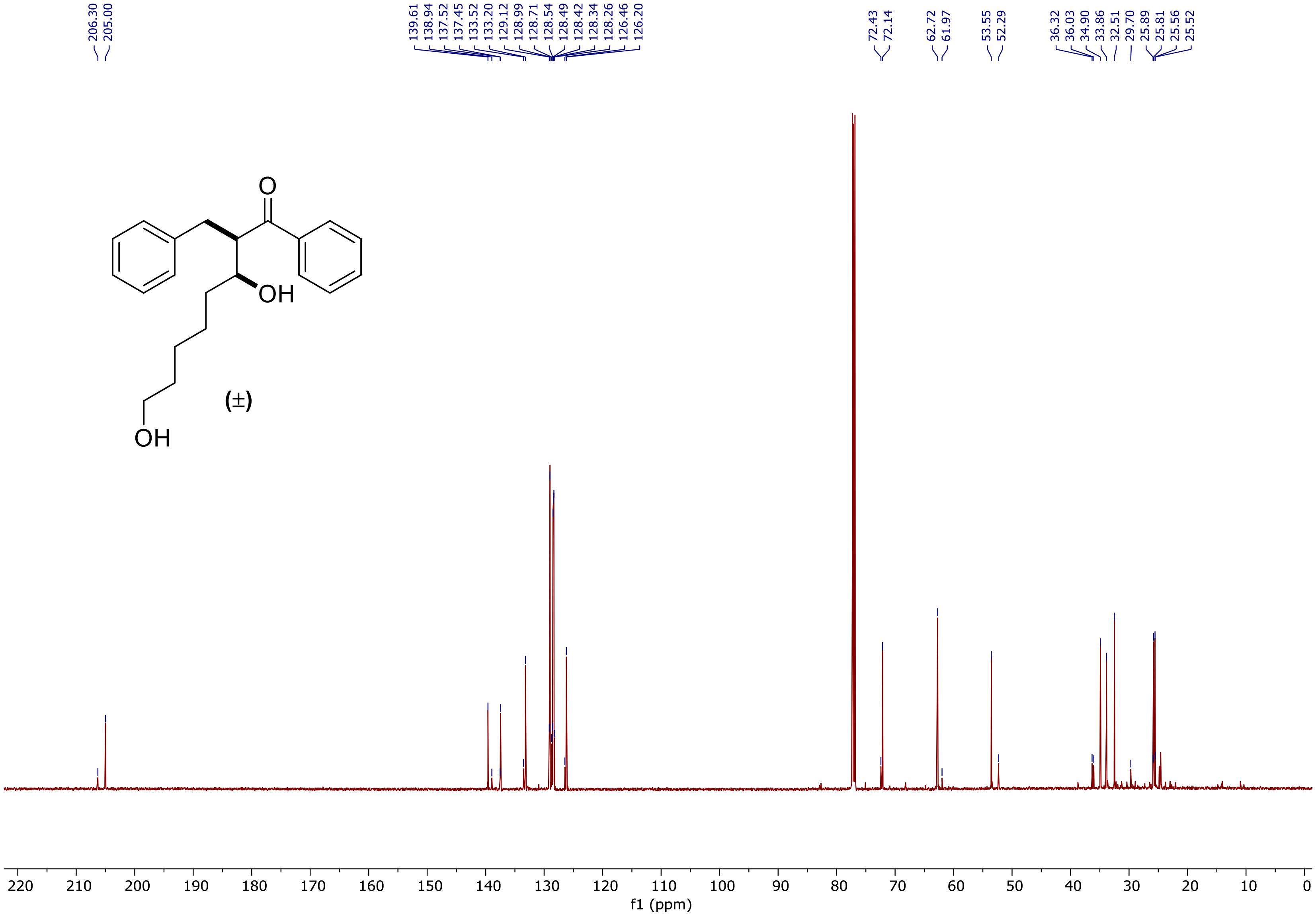

^{13}C NMR (500 MHz, $CDCl_3$) Spectrum of (2*RS*,3*RS*)-2-benzyl-3,8-dihydroxy-1-phenyl-1-octanone **4b**.

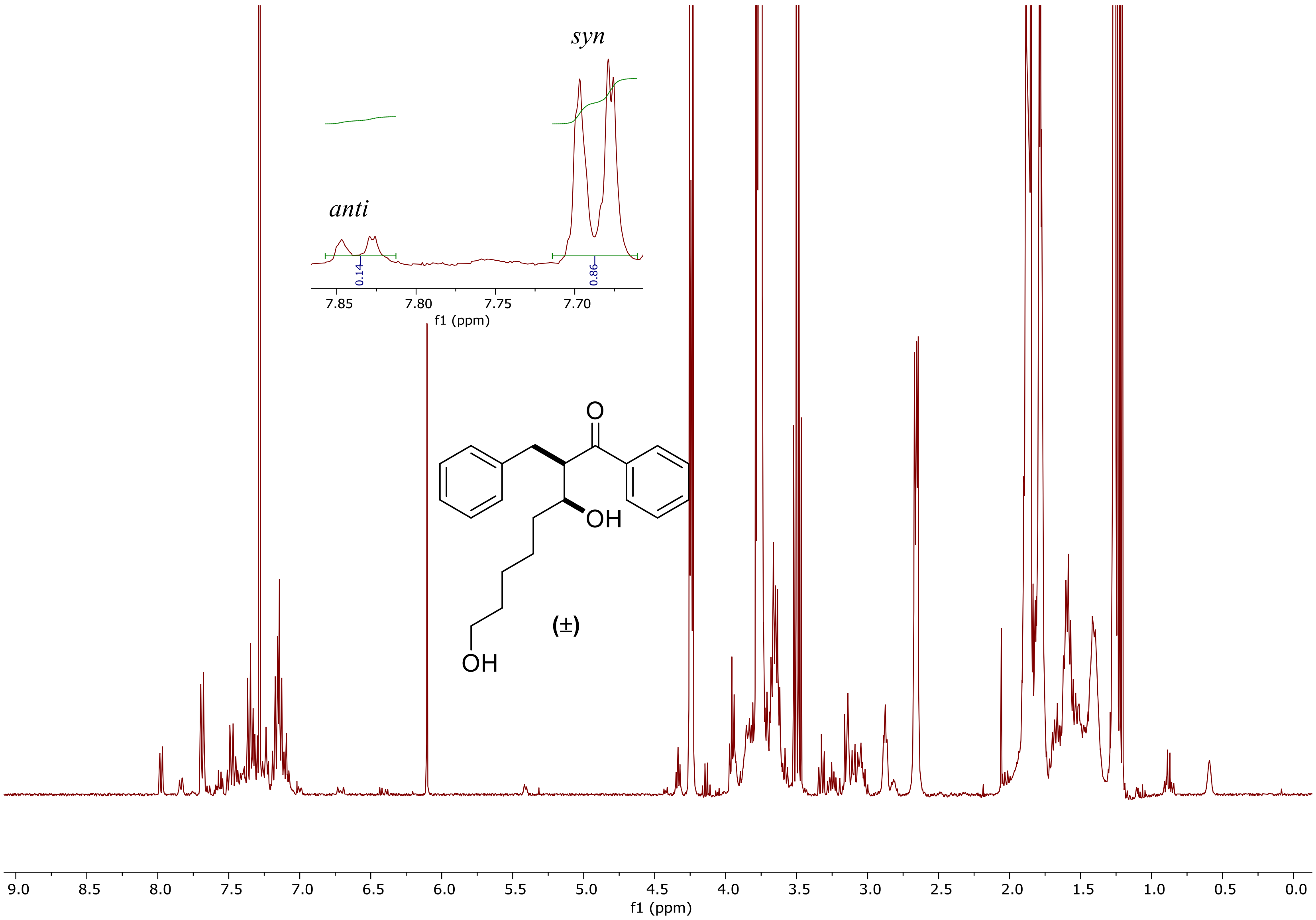

^{1}H NMR (400 MHz, $CDCl_3$) Spectrum of the crude reaction mixture for (2*RS*,3*RS*)-2-benzyl-3,8-dihydroxy-1-phenyl-1-octanone **4b**.

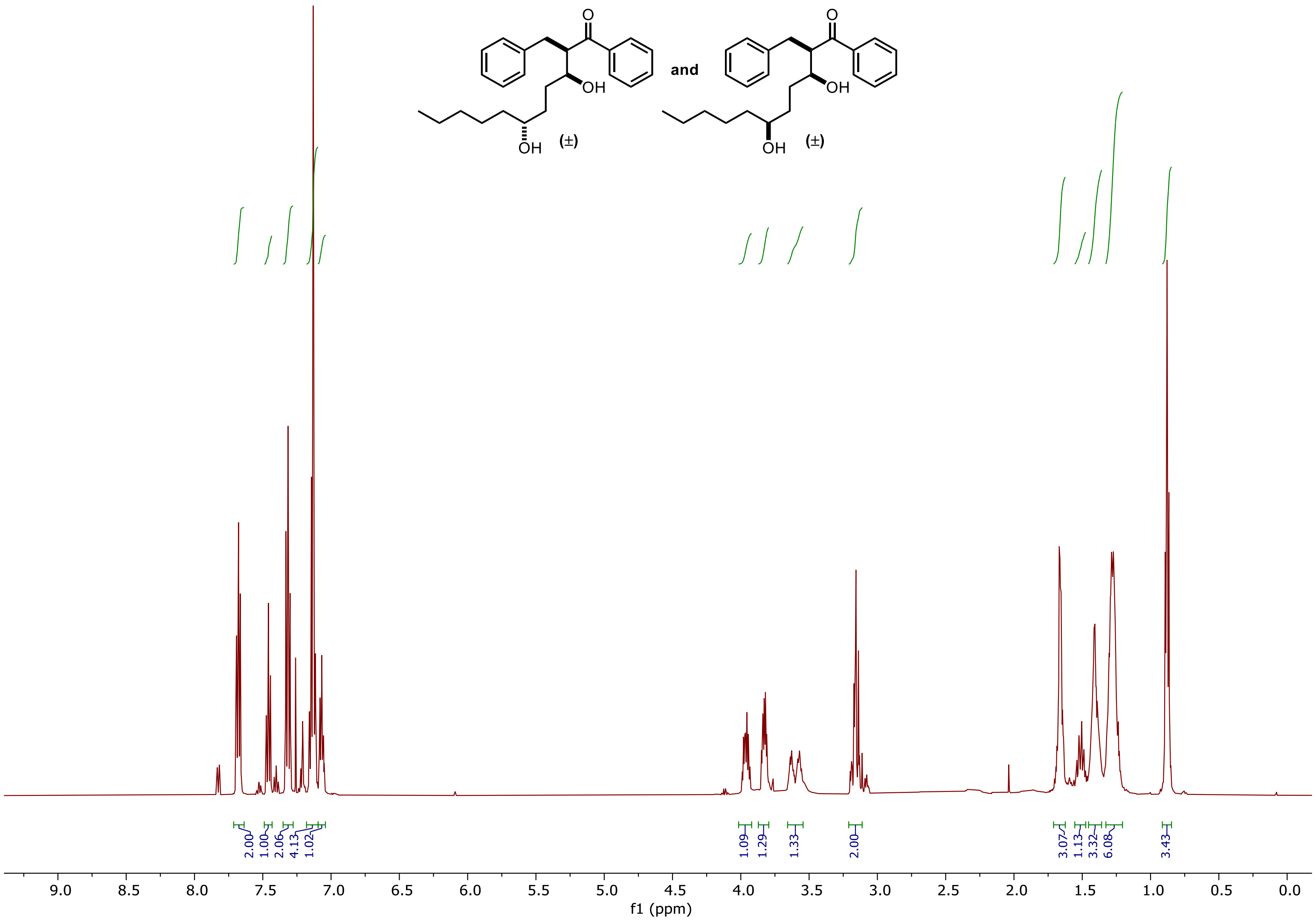

^{1}H NMR (500 MHz, $CDCl_3$) Spectrum of (2RS,3RS,6R)-2-Benzyl-3,6-dihydroxy-1-phenyl-1-undecanone and (2RS,3RS,6S)-2-benzyl-3,6-dihydroxy-1-phenyl-1-undecanone **4c**.

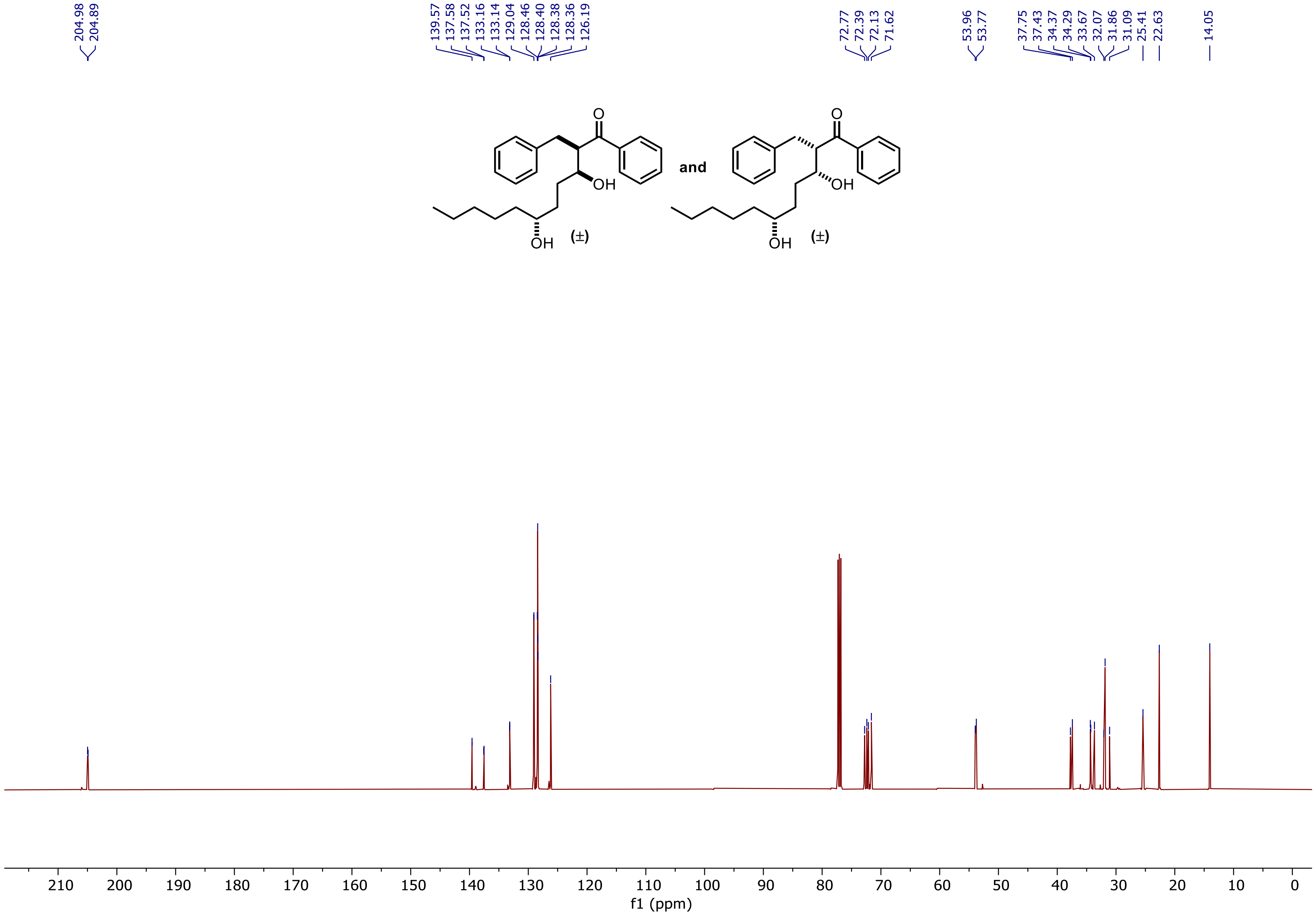

^{13}C NMR (126 MHz, $CDCl_3$) Spectrum (2RS,3RS,6R)-2-Benzyl-3,6-dihydroxy-1-phenyl-1-undecanone and (2RS,3RS,6S)-2-benzyl-3,6-dihydroxy-1-phenyl-1-undecanone **4c**.

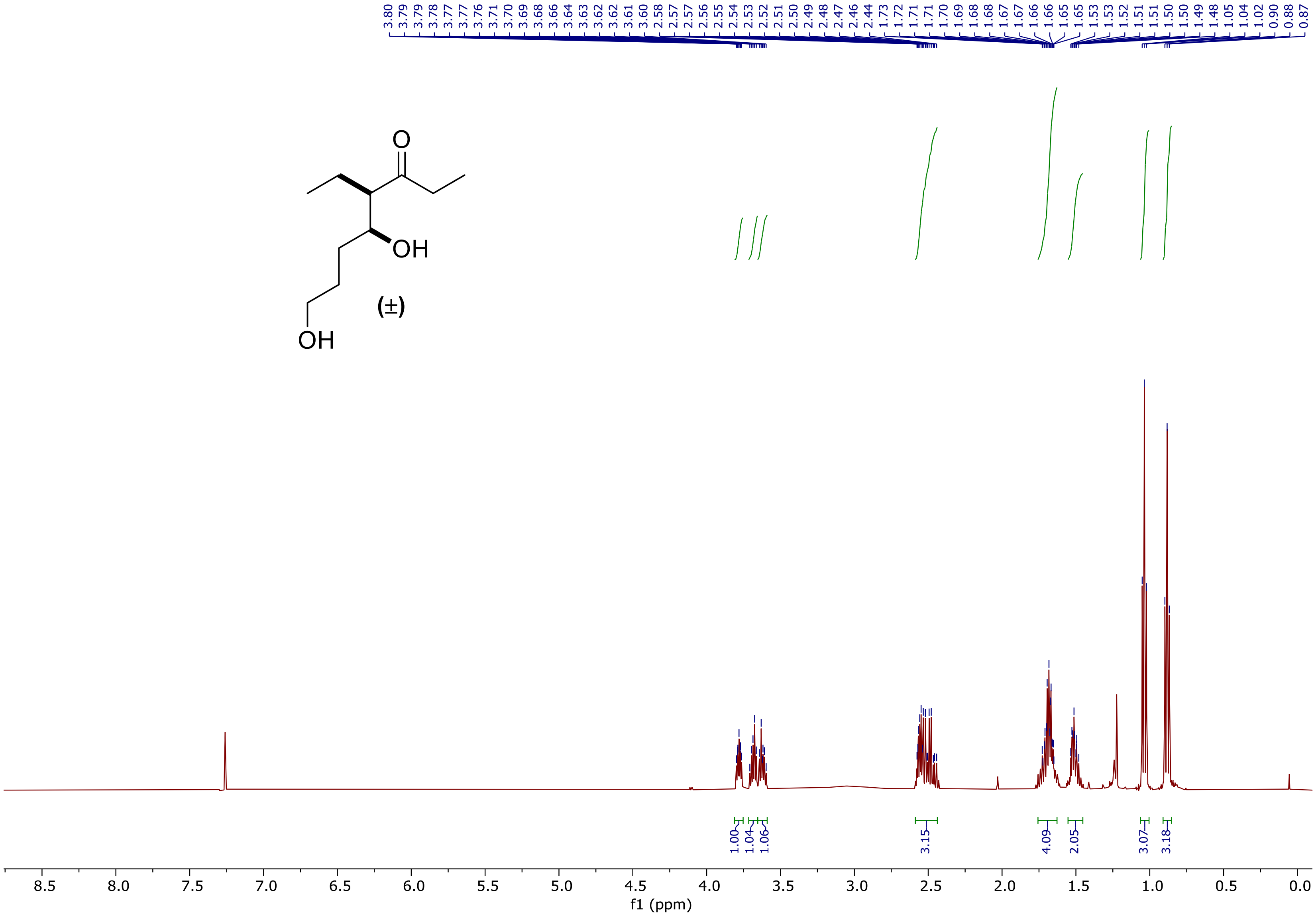

^{1}H NMR (500 MHz, $CDCl_3$) Spectrum of (2*RS*,3*RS*)-5-ethyl-1,4-dihydroxy-6-octanone **4d**.

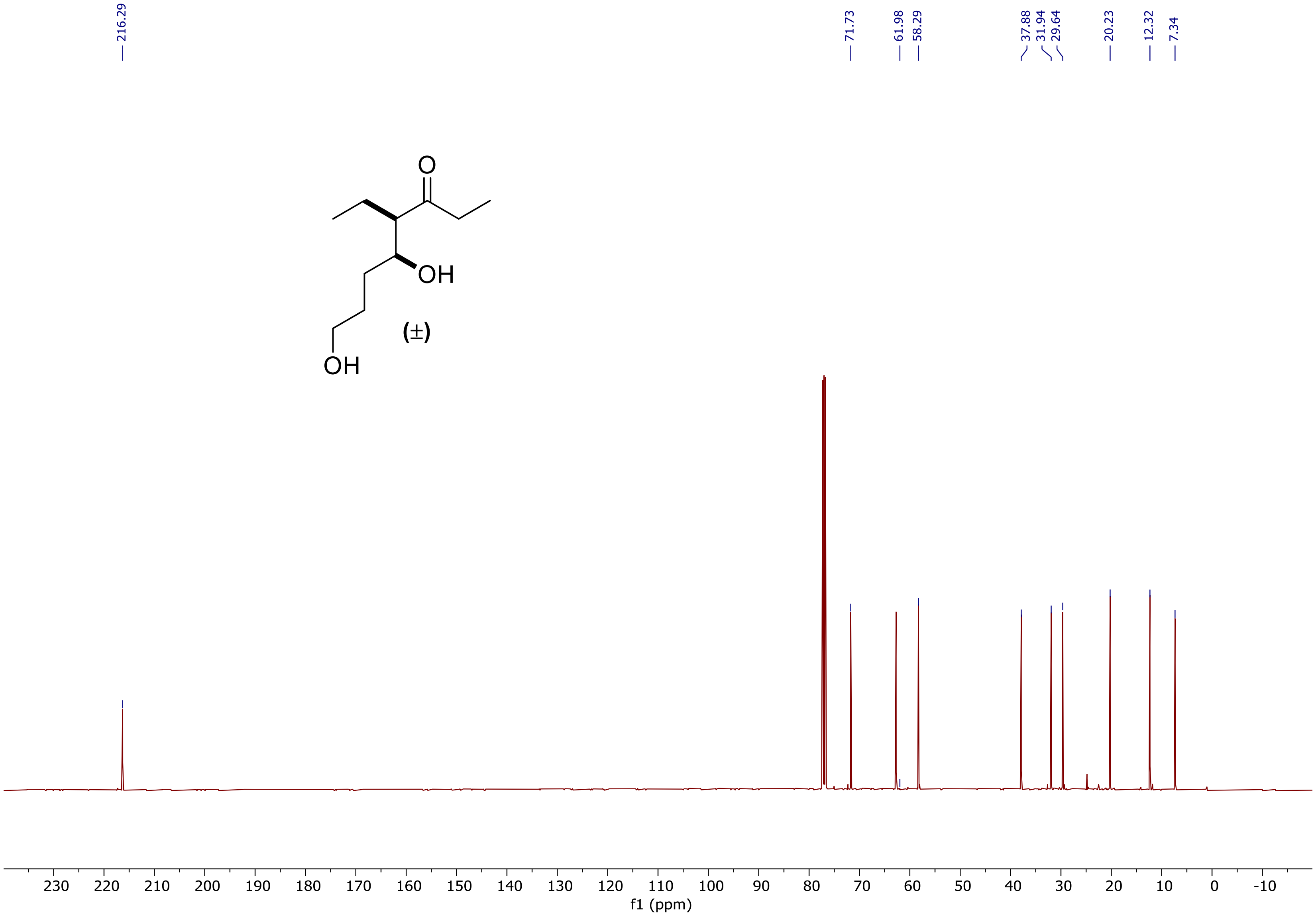

^{13}C NMR (126 MHz, $CDCl_3$) Spectrum of (2*RS*,3*RS*)-5-ethyl-1,4-dihydroxy-6-octanone **4d**.

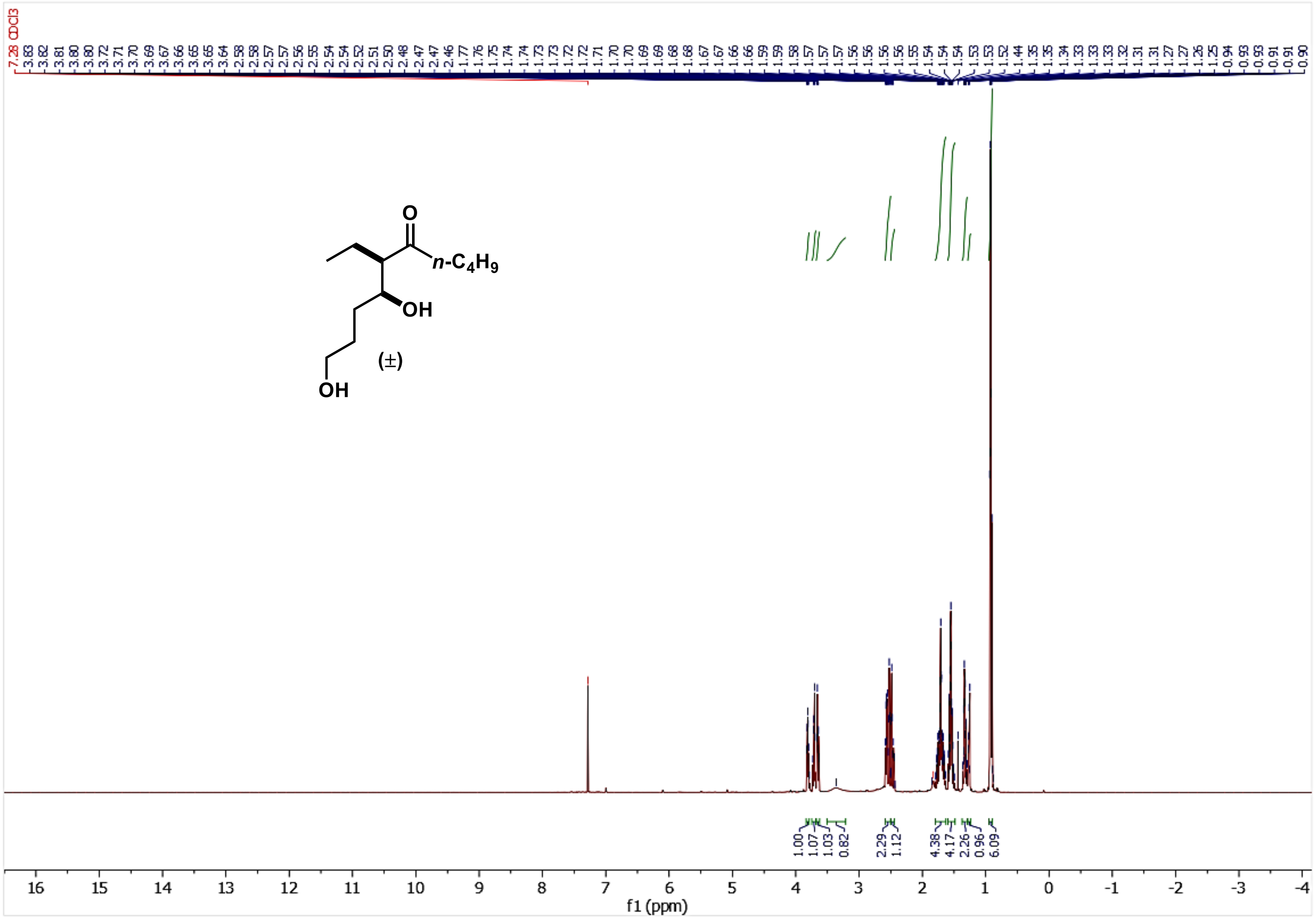

^{1}H NMR (600 MHz, $CDCl_3$) Spectrum of (*2RS,3RS*)-5-ethyl-1,4-dihydroxy-6-decanone, **4e**

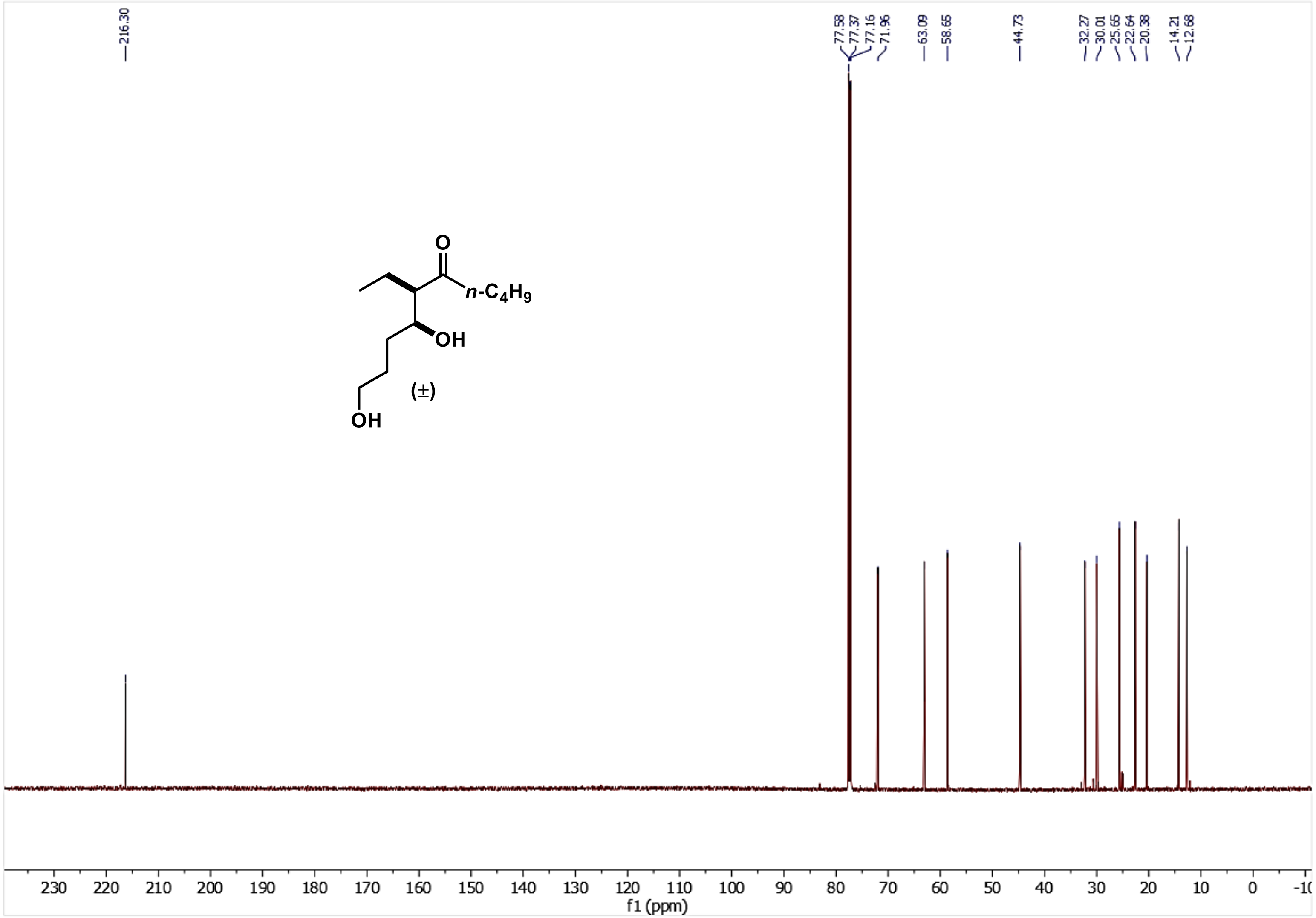

^{13}C NMR (151 MHz, $CDCl_3$) Spectrum of (*2RS,3RS*)-5-ethyl-1,4-dihydroxy-6-decanone, **4e**

THE UNIVERSITY *of* EDINBURGH
Crystal Structure Service

Dr Gary S. Nichol
Crystallography Service Manager
Phone: +44 (0)131 650 4806
E–mail: g.s.nichol@ed.ac.uk

S12 Crystal Data and Experimental

(*2RS,3RS*)-2-Benzyl-3-hydroxy-5,5-dimethyl-1-phenyl-1-hexanone **3d**

Submitted by: **Adrian Moreno**

Solved by: **Gary S Nichol**

Submitted by: **None**

R_1=4.86%

Compound AMG-119 was provided as crystals suitable for single crystal X-ray diffraction, yielding structure **3d**.

Crystal Data and Experimental

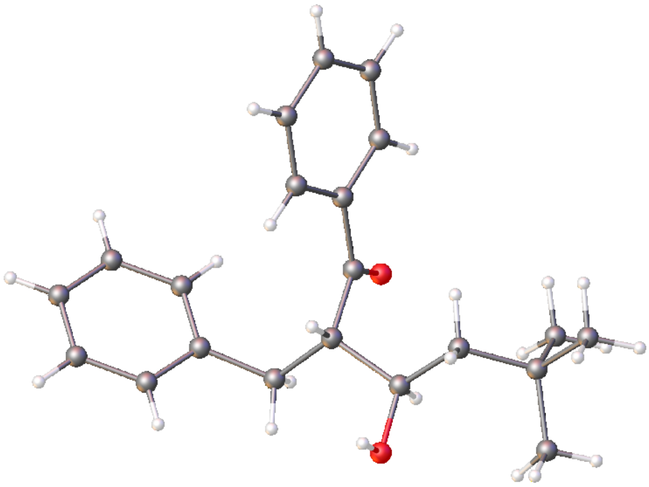

Experimental. Single colourless needle crystals of **3d** recrystallised from a mixture of 40-60 petroleum ether and diethyl ether by slow evaporation. A suitable crystal with dimensions 0.39 × 0.04 × 0.02 mm^3 was selected and mounted on a MITIGEN holder in Paratone oil on a Rigaku Oxford Diffraction SuperNova diffractometer. The crystal was kept at a steady T = 120.00(10) K during data collection. The structure was solved with the ShelXT[5] solution program using dual methods and by using Olex2[6] as the graphical interface. The model was refined with ShelXL 2018/3[7] (Sheldrick, 2015) using full matrix least squares minimisation on $\boldsymbol{F}^2$.

Crystal Data. $C_{21}H_{26}O_2$, M_r = 310.42, monoclinic, *C*2/*c* (No. 15), a = 25.4763(6) Å, b = 5.67818(7) Å, c = 26.6699(6) Å, β = 115.705(3)°, α = γ = 90°, V = 3476.23(14) Å^3, T = 120.00(10) K, Z = 8, Z' = 1, μ(Cu K$_\alpha$) = 0.578, 35134 reflections measured, 3619 unique (R_{int} = 0.0848) which were used in all calculations. The final wR_2 was 0.1395 (all data) and R_1 was 0.0486 (I≥2 σ(I)).

**Compound**	**3d**
Formula	$C_{21}H_{26}O_2$
$D_{calc.}$/ g cm^{-3}	1.186
μ/mm^{-1}	0.578
Formula Weight	310.42
Colour	colourless
Shape	needle
Size/mm^3	0.39×0.04×0.02
T/K	120.00(10)
Crystal System	monoclinic
Space Group	*C*2/*c*
a/Å	25.4763(6)
b/Å	5.67818(7)
c/Å	26.6699(6)
α/°	90
β/°	115.705(3)
γ/°	90
V/Å^3	3476.23(14)
Z	8
Z'	1
Wavelength/Å	1.54184
Radiation type	Cu K$_\alpha$
Θ_{min}/°	3.679
Θ_{max}/°	76.028
Measured Refl's.	35134
Indep't Refl's	3619
Refl's I≥2 σ(I)	3220
R_{int}	0.0848
Parameters	313
Restraints	0
Largest Peak	0.370
Deepest Hole	-0.275
GooF	1.035
wR_2 (all data)	0.1395
wR_2	0.1334
R_1 (all data)	0.0533
R_1	0.0486

Structure Quality Indicators

**Reflections:**	d min (Cu\a) 2θ=152.1° 0.79	I/σ(I) 27.1	Rint 8.48%	CAP 152.1° 99.6
**Refinement:**	Shift -0.001	Max Peak 0.4	Min Peak -0.3	GooF 1.035

A colourless needle-shaped crystal with dimensions 0.39 × 0.04 × 0.02 mm^3 was mounted on a MITIGEN holder in Paratone oil. Data were collected using a Rigaku Oxford Diffraction SuperNova diffractometer equipped with an Oxford Cryosystems Cryostream 700+ low-temperature device operating at T = 120.00(10) K.

Data were measured using ω scans using Cu K_α radiation. The diffraction pattern was indexed and the total number of runs and images was based on the strategy calculation from the program CrysAlisPro[8]. The maximum resolution that was achieved was Θ = 76.028° (0.79 Å).

The diffraction pattern was indexed and the total number of runs and images was based on the strategy calculation from the program CrysAlisPro[8]. The unit cell was refined using CrysAlisPro[8] on 9729 reflections, 28% of the observed reflections.

Data reduction, scaling and absorption corrections were performed using CrysAlisPro[8]. The final completeness is 100.00 % out to 76.028° in Θ. A multi-scan absorption correction was performed using CrysAlisPro[7] 1.171.41.99a. Empirical absorption correction using spherical harmonics, implemented in SCALE3 ABSPACK scaling algorithm. The absorption coefficient μ of this material is 0.578 mm^{-1} at this wavelength (λ = 1.54184Å) and the minimum and maximum transmissions are 0.620 and 1.000.

The structure was solved and the space group *C*2/*c* (# 15) determined by the ShelXT[4] structure solution program using using dual methods and refined by full matrix least squares minimisation on $\boldsymbol{F}^2$ using version 2018/3 of ShelXL 2018/3[7]. All non-hydrogen atoms were refined anisotropically. Hydrogen atom positions were calculated geometrically and refined using the riding model.

_refine_special_details: H atoms were identified from a difference map and freely refined.

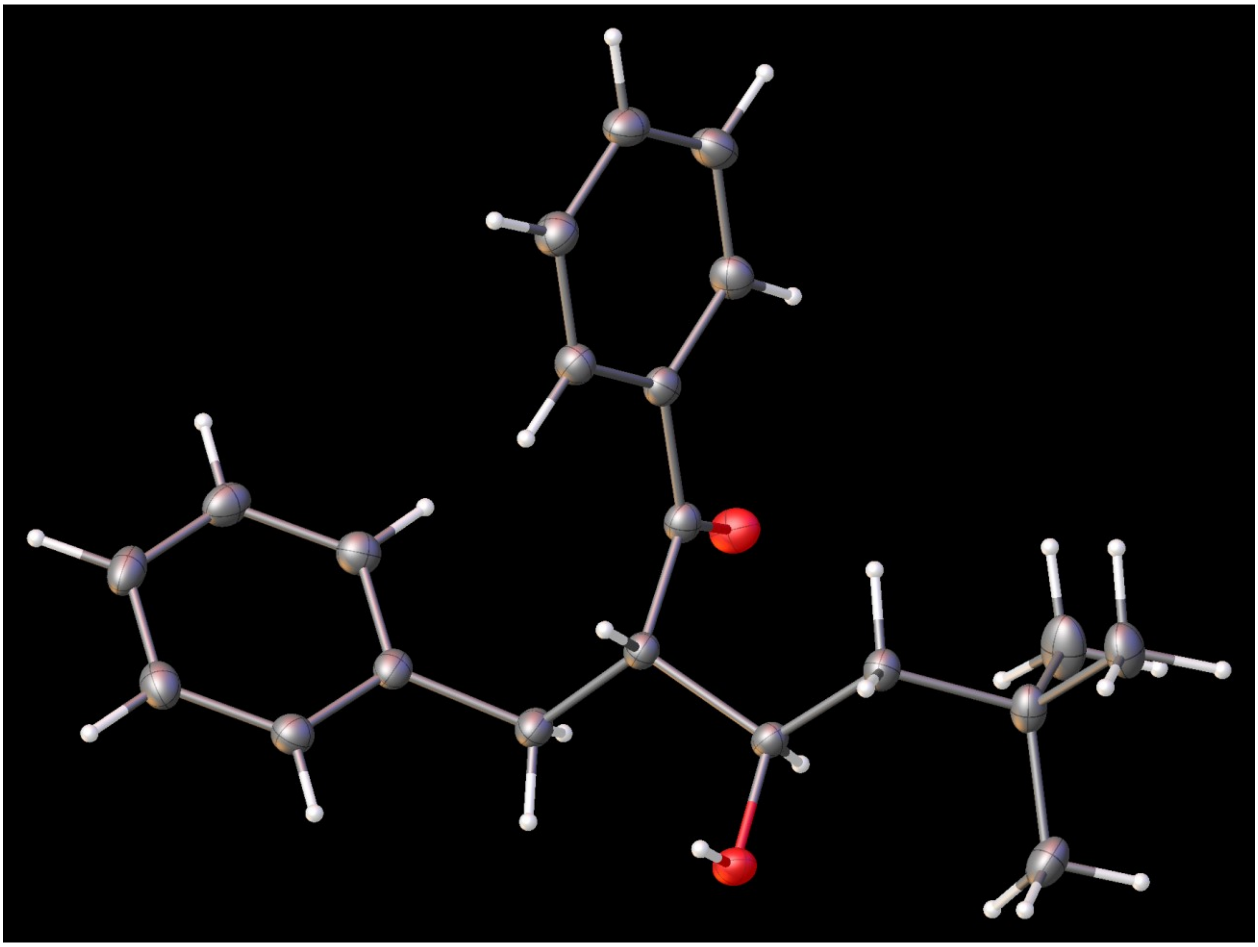

Figure 1: The molecular structure of **3d**. Displacement ellipsoids are at the 50% probability level.

Data Plots: Diffraction Data

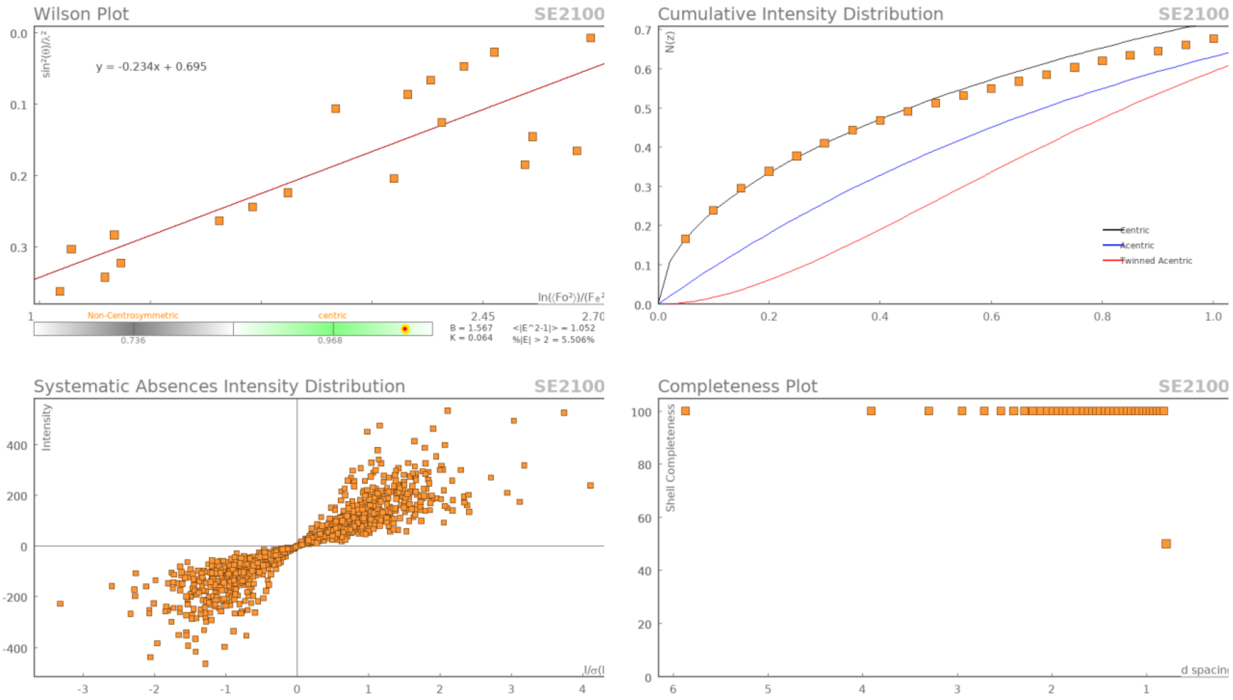

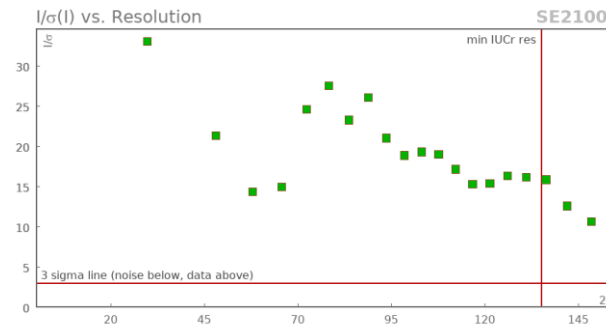

Data Plots: Refinement and Data

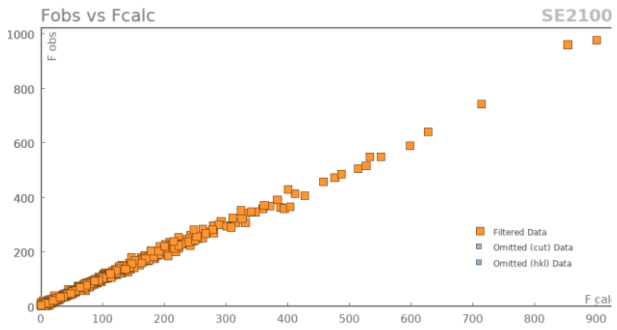

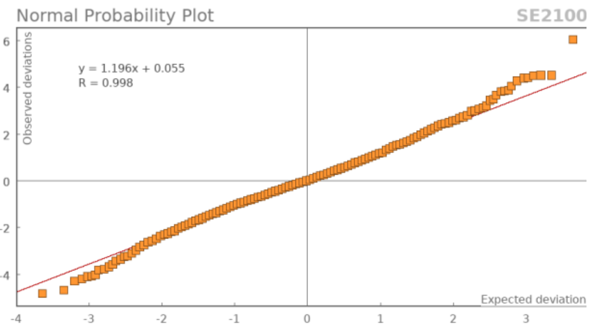

Reflection Statistics

Total reflections (after filtering)	36837	Unique reflections	3619
Completeness	0.996	Mean I/σ	19.16
hkl_{max} collected	(32, 7, 32)	hkl_{min} collected	(-32, -5, -33)
hkl_{max} used	(28, 7, 33)	hkl_{min} used	(-32, 0, 0)
Lim d_{max} collected	100.0	Lim d_{min} collected	0.77
d_{max} used	12.02	d_{min} used	0.79
Friedel pairs	2828	Friedel pairs merged	1
Inconsistent equivalents	8	R_{int}	0.0848
R_{sigma}	0.0369	Intensity transformed	0
Omitted reflections	0	Omitted by user (OMIT hkl)	0
Multiplicity	(2712, 1618, 1362, 1084, 891, 796, 661, 377, 167, 119, 81, 51, 49, 29, 14, 9)	Maximum multiplicity	33
Removed systematic absences	1703	Filtered off (Shel/OMIT)	0

Table 1: Fractional Atomic Coordinates (×10^4) and Equivalent Isotropic Displacement Parameters (Å^2×10^3) for **3d**. U_{eq} is defined as 1/3 of the trace of the orthogonalised U_{ij}.

Atom	x	y	z	U_{eq}
O1	5255.6(4)	8763.1(16)	4492.7(4)	19.6(2)
O2	4455.0(4)	2336.5(16)	3770.2(4)	22.5(2)
C1	5840.7(7)	7352(3)	3151.7(6)	27.2(3)
C2	5760.3(6)	6416(2)	3654.2(6)	20.1(3)
C3	5158.2(5)	7243(2)	3598.5(5)	17.7(3)
C4	5044.1(5)	6839(2)	4112.0(5)	15.4(3)
C5	4390.8(5)	6382(2)	3959.4(5)	15.2(3)
C6	4200.9(5)	4202(2)	3593.1(5)	16.5(3)
C7	3715.4(5)	4290(2)	3018.6(5)	16.4(3)
C8	3311.2(6)	6129(2)	2833.3(5)	19.1(3)
C9	2854.1(6)	6071(2)	2298.9(6)	21.9(3)

Atom	x	y	z	U_{eq}
C10	2806.7(6)	4194(2)	1947.6(5)	22.1(3)
C11	3211.8(6)	2368(2)	2127.9(6)	23.1(3)
C12	3661.0(6)	2403(2)	2660.2(6)	20.9(3)
C13	4311.9(5)	5978(2)	4496.4(5)	18.4(3)
C14	3691.1(5)	6121(2)	4426.1(5)	17.4(3)
C15	3511.1(6)	8080(2)	4627.7(5)	20.0(3)
C16	2948.5(6)	8198(2)	4590.3(5)	22.9(3)
C17	2559.7(6)	6363(3)	4349.4(6)	23.6(3)
C18	2733.9(6)	4411(2)	4144.6(6)	23.0(3)
C19	3294.7(6)	4288(2)	4181.8(5)	20.6(3)
C20	5776.7(7)	3722(3)	3647.7(7)	30.6(4)
C21	6258.7(6)	7316(3)	4191.5(6)	25.8(3)

Table 2: Anisotropic Displacement Parameters (×10^4) for **3d**. The anisotropic displacement factor exponent takes the form: $-2\pi^2[h^2a^{*2} \times U_{11} + ... +2hka^* \times b^* \times U_{12}]$

Atom	U_{11}	U_{22}	U_{33}	U_{23}	U_{13}	U_{12}
O1	18.0(5)	18.6(5)	20.1(4)	-3.8(3)	6.4(4)	-2.2(3)
O2	22.7(5)	16.5(5)	24.6(5)	1.2(3)	6.7(4)	2.2(3)
C1	28.9(7)	29.4(7)	31.7(8)	0.0(6)	21.0(6)	-0.2(6)
C2	20.2(6)	17.0(6)	27.7(7)	-0.1(5)	14.8(5)	0.4(4)
C3	16.2(6)	18.6(6)	19.9(6)	0.7(4)	9.1(5)	0.0(4)
C4	13.5(5)	15.3(6)	16.8(6)	-1.2(4)	5.9(4)	-1.2(4)
C5	13.8(5)	15.9(6)	16.2(5)	-0.1(4)	6.6(4)	-0.4(4)
C6	15.9(6)	15.9(6)	19.2(6)	0.2(4)	8.9(5)	-1.2(4)
C7	16.4(6)	16.4(6)	17.9(6)	-0.1(4)	8.8(5)	-2.3(4)
C8	18.8(6)	18.5(6)	20.4(6)	-1.3(5)	8.9(5)	-0.1(4)
C9	19.7(6)	23.8(7)	21.3(6)	1.6(5)	8.2(5)	1.2(5)
C10	20.8(6)	27.9(7)	16.1(6)	-0.3(5)	6.6(5)	-4.2(5)
C11	26.1(7)	22.9(7)	21.5(6)	-5.5(5)	11.4(5)	-4.6(5)
C12	22.6(6)	17.1(6)	23.8(6)	-2.2(5)	10.9(5)	-1.3(5)
C13	15.0(6)	23.9(6)	16.3(6)	0.6(5)	6.6(5)	0.0(5)
C14	16.4(6)	21.5(6)	14.7(5)	2.9(4)	7.1(4)	0.5(4)
C15	20.6(6)	21.8(6)	17.8(6)	-0.7(5)	8.5(5)	-1.5(5)
C16	23.1(7)	27.3(7)	20.3(6)	2.5(5)	11.5(5)	4.8(5)
C17	16.4(6)	34.6(7)	21.0(6)	6.2(5)	9.2(5)	1.7(5)
C18	20.4(6)	26.4(7)	21.3(6)	2.2(5)	8.2(5)	-5.8(5)
C19	22.1(6)	19.7(6)	20.6(6)	0.7(5)	9.8(5)	-1.3(5)
C20	33.6(8)	17.6(7)	49.7(9)	-0.8(6)	26.7(8)	2.1(5)
C21	16.1(6)	30.5(7)	30.5(7)	2.0(5)	9.9(5)	-0.1(5)

Table 3: Bond Lengths in Å for **3d**.

Atom	Atom	Length/Å
O1	C4	1.4285(14)
O2	C6	1.2245(16)
C1	C2	1.5358(18)
C2	C3	1.5482(16)
C2	C20	1.5307(18)
C2	C21	1.532(2)
C3	C4	1.5357(16)
C4	C5	1.5543(15)
C5	C6	1.5200(16)
C5	C13	1.5474(16)
C6	C7	1.4953(17)
C7	C8	1.3976(18)

Atom	Atom	Length/Å
C7	C12	1.4021(17)
C8	C9	1.3959(19)
C9	C10	1.3895(19)
C10	C11	1.393(2)
C11	C12	1.384(2)
C13	C14	1.5121(16)
C14	C15	1.3966(18)
C14	C19	1.3985(18)
C15	C16	1.3946(18)
C16	C17	1.388(2)
C17	C18	1.391(2)
C18	C19	1.3904(19)

Table 4: Bond Angles in ° for **3d**.

Atom	Atom	Atom	Angle/°
C1	C2	C3	108.24(11)
C20	C2	C1	108.73(12)
C20	C2	C3	109.43(11)
C20	C2	C21	109.16(12)
C21	C2	C1	109.30(11)
C21	C2	C3	111.92(10)
C4	C3	C2	115.59(10)
O1	C4	C3	111.15(10)
O1	C4	C5	110.16(9)
C3	C4	C5	112.76(10)
C6	C5	C4	108.07(9)
C6	C5	C13	109.94(10)
C13	C5	C4	109.67(10)
O2	C6	C5	119.28(11)
O2	C6	C7	119.22(11)
C7	C6	C5	121.50(10)
C8	C7	C6	122.86(11)
C8	C7	C12	119.33(12)
C12	C7	C6	117.80(11)
C9	C8	C7	120.27(12)
C10	C9	C8	119.76(12)
C9	C10	C11	120.25(12)
C12	C11	C10	120.14(12)
C11	C12	C7	120.25(12)
C14	C13	C5	115.21(10)
C15	C14	C13	119.69(11)
C15	C14	C19	118.73(12)
C19	C14	C13	121.53(11)
C16	C15	C14	120.64(12)
C17	C16	C15	120.13(12)
C16	C17	C18	119.64(12)
C19	C18	C17	120.35(13)
C18	C19	C14	120.52(12)

Table 5: Torsion Angles in ° for **3d**.

Atom	Atom	Atom	Atom	Angle/°
O1	C4	C5	C6	-175.39(9)
O1	C4	C5	C13	-55.55(12)
O2	C6	C7	C8	163.54(12)
O2	C6	C7	C12	-14.82(17)
C1	C2	C3	C4	-169.89(11)
C2	C3	C4	O1	86.95(13)
C2	C3	C4	C5	-148.79(10)
C3	C4	C5	C6	59.80(13)
C3	C4	C5	C13	179.64(10)
C4	C5	C6	O2	58.32(14)
C4	C5	C6	C7	-120.93(11)
C4	C5	C13	C14	165.28(10)
C5	C6	C7	C8	-17.21(17)
C5	C6	C7	C12	164.43(11)
C5	C13	C14	C15	-106.50(13)
C5	C13	C14	C19	76.04(15)
C6	C5	C13	C14	-76.02(13)
C6	C7	C8	C9	-177.60(11)
C6	C7	C12	C11	178.56(11)
C7	C8	C9	C10	-0.95(19)
C8	C7	C12	C11	0.14(19)
C8	C9	C10	C11	0.28(19)
C9	C10	C11	C12	0.6(2)
C10	C11	C12	C7	-0.8(2)
C12	C7	C8	C9	0.74(18)
C13	C5	C6	O2	-61.34(14)
C13	C5	C6	C7	119.41(12)
C13	C14	C15	C16	-

Atom	Atom	Atom	Atom	Angle/°
				177.01(11)
C13	C14	C19	C18	177.02(11)
C14	C15	C16	C17	-0.23(19)
C15	C14	C19	C18	-0.47(19)
C15	C16	C17	C18	-0.14(19)
C16	C17	C18	C19	0.2(2)
C17	C18	C19	C14	0.11(19)
C19	C14	C15	C16	0.53(18)
C20	C2	C3	C4	71.76(15)
C21	C2	C3	C4	-49.38(15)

Table 6: Hydrogen Fractional Atomic Coordinates (×10^4) and Equivalent Isotropic Displacement Parameters (Å^2×10^3) for **3d**. U_{eq} is defined as 1/3 of the trace of the orthogonalised U_{ij}.

Atom	x	y	z	U_{eq}
H1	5044(11)	10010(40)	4318(10)	47(6)
H1A	5837(9)	9110(40)	3142(9)	32(5)
H1B	5522(10)	6720(40)	2793(10)	39(5)
H1C	6218(11)	6830(40)	3158(10)	44(6)
H3A	5105(8)	8970(30)	3505(8)	26(4)
H3B	4859(9)	6380(30)	3278(8)	27(4)
H4	5259(7)	5530(30)	4327(6)	11(3)
H5	4164(8)	7750(30)	3764(7)	17(4)
H8	3341(9)	7460(30)	3076(8)	28(5)
H9	2573(9)	7340(40)	2183(9)	32(5)
H10	2495(10)	4100(40)	1588(9)	37(5)
H11	3184(8)	1080(30)	1877(8)	26(4)
H12	3952(9)	1160(30)	2802(8)	29(5)
H13A	4475(8)	4430(30)	4654(8)	27(4)
H13B	4540(8)	7180(30)	4775(7)	18(4)
H15	3777(8)	9270(30)	4803(8)	24(4)
H16	2823(9)	9580(40)	4738(9)	33(5)
H17	2172(10)	6440(40)	4328(9)	39(5)
H18	2456(9)	3170(40)	3965(9)	31(5)
H19	3409(8)	2890(30)	4047(8)	24(4)
H20A	5715(10)	2980(40)	3963(10)	40(5)
H20B	6152(10)	3200(40)	3659(10)	41(5)
H20C	5444(10)	3150(40)	3295(10)	40(5)
H21A	6271(9)	9100(40)	4226(9)	34(5)
H21B	6254(10)	6700(40)	4546(10)	40(5)
H21C	6623(10)	6780(40)	4204(9)	41(5)

THE UNIVERSITY of EDINBURGH
Crystal Structure Service

Dr Gary S. Nichol
Crystallography Service Manager
Phone: +44 (0)131 650 4806
E–mail: g.s.nichol@ed.ac.uk

3β-acetoxy-17 α-(ethan-1-ol)-5-pregnen-2-one **3x**

Submitted by: **Kieran Nicholson**

Solved by: **Gary S Nichol**

Submitted by: **None**

R_1=7.17%

Compound KN07-055 ab was provided as small colourless crystals suitable for single crystal X-ray diffraction, yielding structure **3x**.

Crystal Data and Experimental

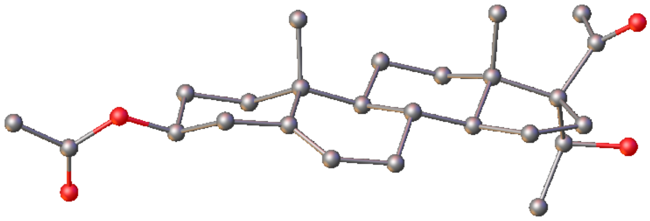

Experimental. Single colourless plate crystals of **3x** recrystallised from a mixture of ethyl acetate, THF and diethyl ether by slow evaporation. A suitable crystal with dimensions 0.13 × 0.08 × 0.05 mm^3 was selected and mounted on a MITIGEN holder in Paratone oil. on a Bruker D8 VENTURE diffractometer. The crystal was kept at a steady T = 100.0 K during data collection. The structure was solved with the ShelXT 2018/2[5] solution program using dual methods and by using Olex2[6] as the graphical interface. The model was refined with ShelXL 2018/3[7] using full matrix least squares minimisation on $\boldsymbol{F}^2$.

Crystal Data. $C_{25}H_{38}O_4$, M_r = 402.55, monoclinic, $C2$ (No. 5), a = 17.573(3) Å, b = 6.6032(11) Å, c = 19.171(3) Å, β = 100.052(6)°, α = γ = 90°, V = 2190.5(6) Å^3, T = 100.0 K, Z = 4, Z' = 1, μ(MoK$_\alpha$) = 0.081, 18134 reflections measured, 3821 unique (R_{int} = 0.0598) which were used in all calculations. The final wR_2 was 0.2236 (all data) and R_1 was 0.0717 (I≥2 σ(I)).

**Compound**	**3x**
Formula	$C_{25}H_{38}O_4$
$D_{calc.}$/ g cm^{-3}	1.221
μ/mm^{-1}	0.081
Formula Weight	402.55
Colour	colourless
Shape	plate
Size/mm^3	0.13×0.08×0.05
T/K	100.0
Crystal System	monoclinic
Flack Parameter	-0.3(8)
Hooft Parameter	-0.2(7)
Space Group	$C2$
a/Å	17.573(3)
b/Å	6.6032(11)
c/Å	19.171(3)
α/°	90
β/°	100.052(6)
γ/°	90
V/Å^3	2190.5(6)
Z	4
Z'	1
Wavelength/Å	0.71073
Radiation type	MoK$_\alpha$
Θ_{min}/°	2.412
Θ_{max}/°	25.349
Measured Refl's.	18134
Indep't Refl's	3821
Refl's I≥2 σ(I)	3154
R_{int}	0.0598
Parameters	271
Restraints	1
Largest Peak	0.483
Deepest Hole	-0.338
GooF	1.107
wR_2 (all data)	0.2236
wR_2	0.2102
R_1 (all data)	0.0840
R_1	0.0717

Structure Quality Indicators

**Reflections:**	d min (Mo) 2θ=50.7° 0.83	I/σ(I) 21.0	Rint 5.98%	Full 50.5° 99.6	
**Refinement:**	Shift 0.000	Max Peak 0.5	Min Peak -0.3	GooF 1.107	Flack -.3(8)

A colourless plate-shaped crystal with dimensions 0.13 × 0.08 × 0.05 mm^3 was mounted on a MITIGEN holder in Paratone oil. Data were collected using a Bruker D8 VENTURE diffractometer equipped with an Oxford Cryosystems Cryostream 800 low-temperature device operating at T = 100.0 K.

Data were measured using ϕ and ω scans using MoK_α radiation. The maximum resolution that was achieved was Θ = 25.349° (0.83 Å).

The unit cell was refined using SAINT[8] on 2936 reflections, 16% of the observed reflections.

Data reduction, scaling and absorption corrections were performed using SAINT[9] (Bruker, V8.40A, after 2013). The final completeness is 99.60 % out to 25.349° in Θ. A multi-scan absorption correction was performed using SADABS-2016/2[10] was used for absorption correction. wR_2(int) was 0.0900 before and 0.0621 after correction. The Ratio of minimum to maximum transmission is 0.8932. The λ/2 correction factor is Not present. The absorption coefficient μ of this material is 0.081 mm^{-1} at this wavelength (λ = 0.71073Å) and the minimum and maximum transmissions are 0.666 and 0.745.

The structure was solved and the space group $C2$ (# 5) determined by the ShelXT[5] 2018/2 structure solution program using using dual methods and refined by full matrix least squares minimisation on $\boldsymbol{F}^2$ using version 2018/3 of ShelXL 2018/3[7]. All non-hydrogen atoms were refined anisotropically. Hydrogen atom positions were calculated geometrically and refined using the riding model. Most hydrogen atom positions were calculated geometrically and refined using the riding model, but some hydrogen atoms were refined freely.

_refine_special_details: The O-bound H atom was identified from a difference map and freely refined.

The Flack parameter was refined to -0.3(8). Determination of absolute structure using Bayesian statistics on Bijvoet differences using the Olex2 results in -0.2(7). Note: The Flack parameter is used to determine chirality of the crystal studied, the value should be near 0, a value of 1 means that the stereochemistry is wrong and the model should be inverted. A value of 0.5 means that the crystal consists of a racemic mixture of the two enantiomers.

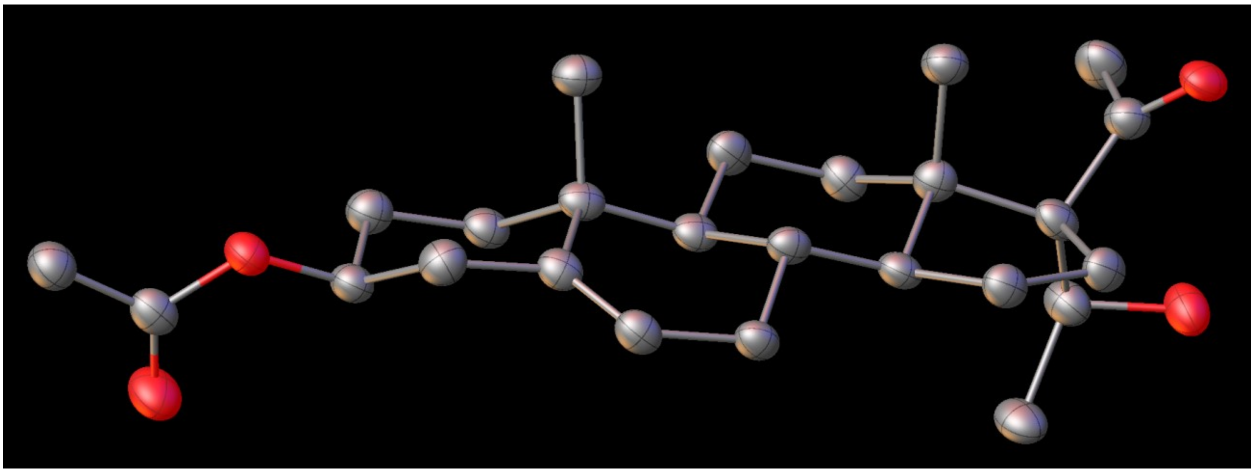

Figure 2: The molecular structure of **3x**. Displacement ellipsoids are at the 50% probability level. C-bound H atoms are not shown.

Data Plots: Diffraction Data

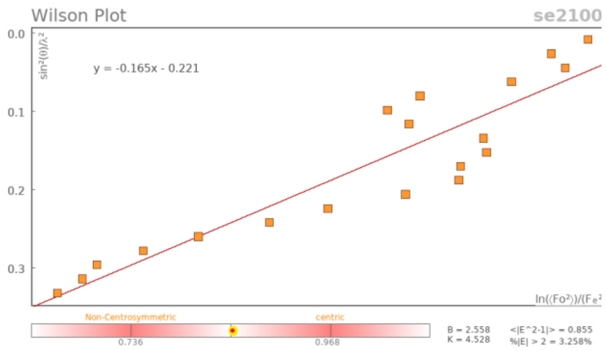

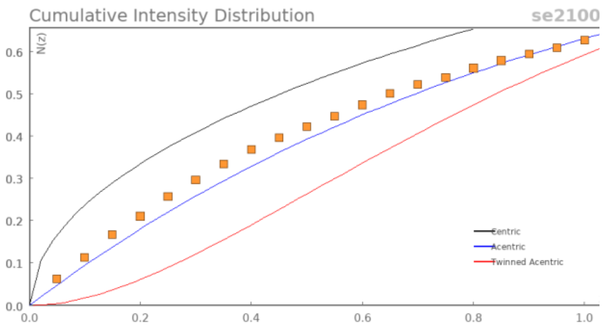

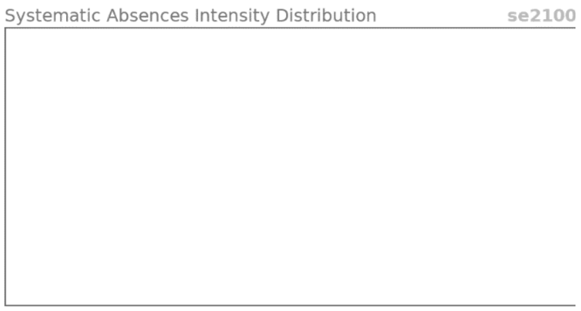

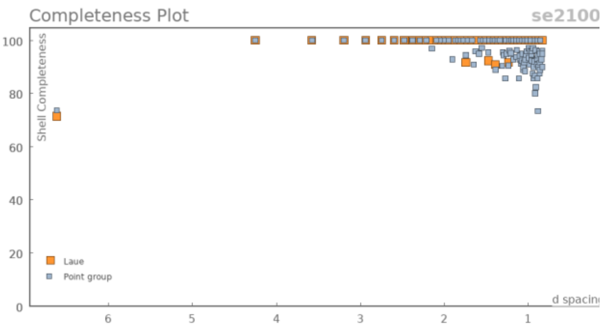

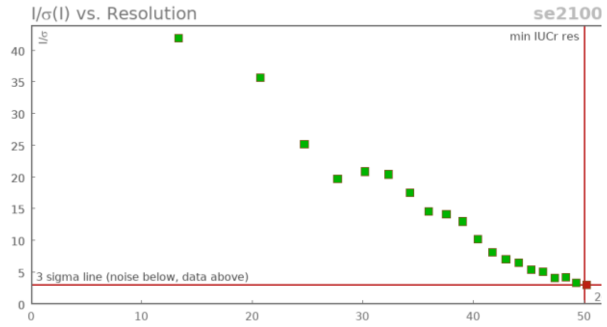

Data Plots: Refinement and Data

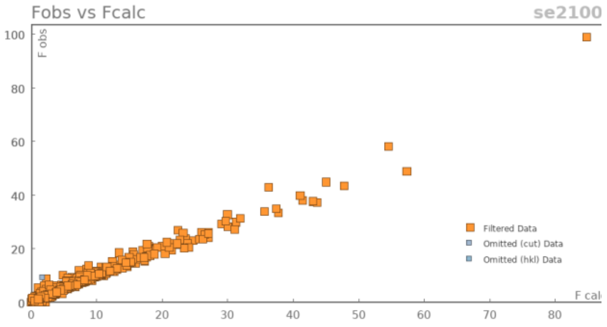

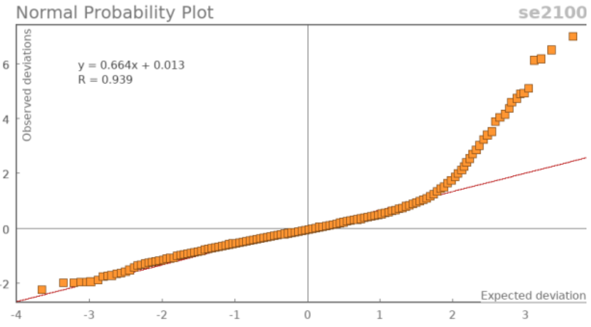

Reflection Statistics

Total reflections (after filtering)	18140	Unique reflections	3821
Completeness	0.955	Mean I/σ	14.18
hkl_{max} collected	(21, 7, 23)	hkl_{min} collected	(-21, -7, -23)
hkl_{max} used	(20, 7, 23)	hkl_{min} used	(-21, -7, 0)
Lim d_{max} collected	100.0	Lim d_{min} collected	0.36
d_{max} used	8.44	d_{min} used	0.83
Friedel pairs	3131	Friedel pairs merged	0

Inconsistent equivalents	0	R_{int}	0.0598
R_{sigma}	0.0476	Intensity transformed	0
Omitted reflections	0	Omitted by user (OMIT hkl)	6
Multiplicity	(1474, 1734, 2883, 552, 353, 96)	Maximum multiplicity	12
Removed systematic absences	0	Filtered off (Shel/OMIT)	0

Table 7: Fractional Atomic Coordinates (×10^4) and Equivalent Isotropic Displacement Parameters (Å^2×10^3) for **3x**. U_{eq} is defined as 1/3 of the trace of the orthogonalised U_{ij}.

Atom	x	y	z	U_{eq}
O1	7888(2)	-653(6)	1267(2)	33.3(9)
O2	7176(3)	2226(7)	7(2)	39.4(11)
O3	3625(2)	7859(6)	4515(2)	32.7(9)
O4	3000(3)	10639(7)	4044(2)	46.7(12)
C1	6866(3)	1833(8)	1135(3)	28.0(12)
C2	6306(3)	22(9)	927(3)	29.4(12)
C3	5658(3)	146(8)	1377(3)	29.5(12)
C4	5748(3)	2264(8)	1709(3)	27.0(11)
C5	5372(3)	2685(9)	2352(3)	25.2(11)
C6	4498(3)	2381(9)	2174(3)	28.1(11)
C7	4089(3)	3198(9)	2732(3)	29.7(12)
C8	4394(3)	4482(9)	3235(3)	27.3(11)
C9	3946(3)	5168(10)	3799(3)	33.4(13)
C10	3946(3)	7476(9)	3880(3)	28.0(12)
C11	4750(3)	8350(9)	3959(3)	32.3(13)
C12	5144(3)	7697(9)	3354(3)	29.1(12)
C13	5203(3)	5349(9)	3280(3)	27.0(12)
C14	5537(3)	4884(8)	2603(3)	26.2(11)
C15	6395(3)	5424(9)	2675(3)	29.9(12)
C16	6756(3)	4902(9)	2024(3)	30.8(12)
C17	6631(3)	2644(9)	1840(3)	27.3(11)
C18	5721(3)	4494(10)	3946(3)	32.5(12)
C19	7041(3)	1327(10)	2451(3)	31.4(12)
C20	7713(3)	1123(9)	1273(3)	31.0(13)
C21	8346(3)	2680(11)	1420(3)	39.4(14)
C22	6837(3)	3349(10)	519(3)	33.2(13)
C23	6055(4)	4166(10)	168(3)	38.4(14)
C24	3187(3)	9511(9)	4545(3)	32.1(12)
C25	2948(3)	9759(10)	5249(3)	37.3(14)

Table 8: Anisotropic Displacement Parameters (×10^4) for **3x**. The anisotropic displacement factor exponent takes the form: $-2\pi^2[h^2a^{*2} \times U_{11} + ... +2hka^* \times b^* \times U_{12}]$

Atom	U_{11}	U_{22}	U_{33}	U_{23}	U_{13}	U_{12}
O1	34(2)	27(2)	36(2)	2.9(17)	-1.6(15)	7.2(17)
O2	44(2)	38(3)	39(2)	5.7(19)	15.0(19)	10.8(19)
O3	32(2)	30(2)	34.8(19)	2.4(17)	4.4(16)	7.4(17)
O4	54(3)	38(3)	48(3)	8(2)	8(2)	14(2)
C1	25(3)	20(3)	38(3)	0(2)	2(2)	2(2)
C2	30(3)	21(3)	35(3)	-4(2)	1(2)	-1(2)
C3	30(3)	22(3)	36(3)	-1(2)	2(2)	-2(2)
C4	22(2)	25(3)	32(3)	2(2)	-1(2)	3(2)
C5	22(2)	22(3)	29(2)	0(2)	-1.5(19)	-3(2)
C6	23(2)	27(3)	33(3)	-1(2)	0(2)	-1(2)
C7	24(2)	25(3)	39(3)	1(2)	4(2)	-2(2)
C8	28(3)	21(3)	33(3)	4(2)	5(2)	-1(2)
C9	35(3)	33(3)	32(3)	1(2)	6(2)	-2(2)
C10	26(3)	27(3)	30(3)	1(2)	2(2)	3(2)

Atom	U_{11}	U_{22}	U_{33}	U_{23}	U_{13}	U_{12}
C11	32(3)	29(3)	35(3)	0(2)	3(2)	-1(2)
C12	28(3)	24(3)	34(3)	3(2)	1(2)	1(2)
C13	27(3)	20(3)	32(3)	4(2)	0(2)	-1(2)
C14	27(3)	19(3)	30(3)	4(2)	0(2)	1(2)
C15	27(3)	23(3)	37(3)	-5(2)	0(2)	-4(2)
C16	26(2)	27(3)	39(3)	1(2)	6(2)	-1(2)
C17	25(3)	26(3)	30(2)	-1(2)	3(2)	-4(2)
C18	32(3)	31(3)	33(3)	3(2)	0(2)	-1(2)
C19	28(3)	32(3)	33(3)	0(2)	0(2)	0(2)
C20	29(3)	31(3)	32(3)	0(2)	1(2)	0(2)
C21	24(3)	41(4)	52(3)	-6(3)	3(2)	-2(3)
C22	31(3)	34(3)	36(3)	5(2)	8(2)	4(2)
C23	40(3)	38(4)	38(3)	12(3)	6(2)	9(3)
C24	27(3)	25(3)	43(3)	0(3)	4(2)	-2(2)
C25	38(3)	32(3)	42(3)	1(3)	6(2)	3(3)

Table 9: Bond Lengths in Å for **3x**.

Atom	Atom	Length/Å
O1	C20	1.213(7)
O2	C22	1.439(7)
O3	C10	1.451(6)
O3	C24	1.342(7)
O4	C24	1.214(7)
C1	C2	1.556(8)
C1	C17	1.575(7)
C1	C20	1.539(7)
C1	C22	1.541(8)
C2	C3	1.547(7)
C3	C4	1.534(8)
C4	C5	1.522(7)
C4	C17	1.548(7)
C5	C6	1.528(7)
C5	C14	1.541(7)
C6	C7	1.491(8)

Atom	Atom	Length/Å
C7	C8	1.324(8)
C8	C9	1.515(7)
C8	C13	1.520(7)
C9	C10	1.532(8)
C10	C11	1.510(8)
C11	C12	1.513(8)
C12	C13	1.562(8)
C13	C14	1.545(7)
C13	C18	1.540(7)
C14	C15	1.532(7)
C15	C16	1.534(7)
C16	C17	1.539(8)
C17	C19	1.535(8)
C20	C21	1.504(8)
C22	C23	1.520(8)
C24	C25	1.490(8)

Table 10: Bond Angles in ° for **3x**.

Atom	Atom	Atom	Angle/°
C24	O3	C10	119.2(4)
C2	C1	C17	103.4(4)
C20	C1	C2	111.1(5)
C20	C1	C17	110.3(4)
C20	C1	C22	103.4(4)
C22	C1	C2	111.6(5)
C22	C1	C17	117.2(5)
C3	C2	C1	108.1(4)
C4	C3	C2	104.3(4)
C3	C4	C17	104.1(4)
C5	C4	C3	118.3(5)
C5	C4	C17	113.8(4)
C4	C5	C6	110.9(4)
C4	C5	C14	109.9(4)
C6	C5	C14	108.7(4)
C7	C6	C5	112.5(4)
C8	C7	C6	124.7(5)
C7	C8	C9	121.1(5)

Atom	Atom	Atom	Angle/°
C7	C8	C13	122.6(5)
C9	C8	C13	116.3(5)
C8	C9	C10	112.2(5)
O3	C10	C9	105.4(4)
O3	C10	C11	110.1(4)
C11	C10	C9	112.0(5)
C10	C11	C12	111.0(5)
C11	C12	C13	113.5(5)
C8	C13	C12	107.6(4)
C8	C13	C14	111.4(4)
C8	C13	C18	108.8(4)
C14	C13	C12	108.3(4)
C18	C13	C12	109.1(5)
C18	C13	C14	111.5(4)
C5	C14	C13	111.8(4)
C15	C14	C5	112.0(4)
C15	C14	C13	113.0(4)
C14	C15	C16	114.6(4)

Atom	Atom	Atom	Angle/°
C15	C16	C17	110.0(4)
C4	C17	C1	102.3(4)
C16	C17	C1	118.6(4)
C16	C17	C4	107.1(4)
C19	C17	C1	107.7(4)
C19	C17	C4	110.9(4)
C19	C17	C16	110.1(4)
O1	C20	C1	122.1(5)
O1	C20	C21	118.9(5)
C21	C20	C1	119.0(5)
O2	C22	C1	103.0(5)
O2	C22	C23	109.3(5)
C23	C22	C1	118.5(5)
O3	C24	C25	112.4(5)
O4	C24	O3	122.9(5)
O4	C24	C25	124.7(6)

Table 11: Torsion Angles in ° for **3x**.

Atom	Atom	Atom	Atom	Angle/°
O3	C10	C11	C12	171.3(4)
C1	C2	C3	C4	12.4(6)
C2	C1	C17	C4	-34.2(5)
C2	C1	C17	C16	-151.6(5)
C2	C1	C17	C19	82.7(5)
C2	C1	C20	O1	-5.1(8)
C2	C1	C20	C21	174.8(5)
C2	C1	C22	O2	-68.3(5)
C2	C1	C22	C23	52.4(7)
C2	C3	C4	C5	-161.8(4)
C2	C3	C4	C17	-34.3(5)
C3	C4	C5	C6	-60.2(6)
C3	C4	C5	C14	179.6(4)
C3	C4	C17	C1	42.9(5)
C3	C4	C17	C16	168.2(4)
C3	C4	C17	C19	-71.6(5)
C4	C5	C6	C7	-167.6(5)
C4	C5	C14	C13	-177.0(4)
C4	C5	C14	C15	-48.9(5)
C5	C4	C17	C1	173.1(4)
C5	C4	C17	C16	-61.6(6)
C5	C4	C17	C19	58.6(6)
C5	C6	C7	C8	16.2(8)
C5	C14	C15	C16	50.1(6)
C6	C5	C14	C13	61.5(5)
C6	C5	C14	C15	-170.4(4)
C6	C7	C8	C9	-177.5(5)
C6	C7	C8	C13	2.6(9)
C7	C8	C9	C10	-129.8(6)
C7	C8	C13	C12	129.8(6)
C7	C8	C13	C14	11.1(7)
C7	C8	C13	C18	-112.1(6)
C8	C9	C10	O3	-169.8(4)
C8	C9	C10	C11	-50.2(6)
C8	C13	C14	C5	-43.1(6)
C8	C13	C14	C15	-170.7(5)
C9	C8	C13	C12	-50.2(6)
C9	C8	C13	C14	-

Atom	Atom	Atom	Atom	Angle/°
				168.8(4)
C9	C8	C13	C18	67.9(6)
C9	C10	C11	C12	54.5(6)
C10	O3	C24	O4	5.3(8)
C10	O3	C24	C25	-176.0(5)
C10	C11	C12	C13	-57.8(6)
C11	C12	C13	C8	53.7(6)
C11	C12	C13	C14	174.3(4)
C11	C12	C13	C18	-64.2(6)
C12	C13	C14	C5	-161.3(4)
C12	C13	C14	C15	71.1(5)
C13	C8	C9	C10	50.1(6)
C13	C14	C15	C16	177.6(4)
C14	C5	C6	C7	-46.7(6)
C14	C15	C16	C17	-55.2(6)
C15	C16	C17	C1	172.7(4)
C15	C16	C17	C4	57.9(5)
C15	C16	C17	C19	-62.7(6)
C17	C1	C2	C3	13.6(5)
C17	C1	C20	O1	108.9(6)
C17	C1	C20	C21	-71.1(6)
C17	C1	C22	O2	172.7(5)
C17	C1	C22	C23	-66.5(7)
C17	C4	C5	C6	177.1(5)
C17	C4	C5	C14	56.9(6)
C18	C13	C14	C5	78.6(6)
C18	C13	C14	C15	-48.9(6)
C20	C1	C2	C3	131.9(5)
C20	C1	C17	C4	-153.0(5)
C20	C1	C17	C16	89.6(6)
C20	C1	C17	C19	-36.2(6)
C20	C1	C22	O2	51.2(5)
C20	C1	C22	C23	171.9(5)
C22	C1	C2	C3	-113.2(5)
C22	C1	C17	C4	89.1(5)
C22	C1	C17	C16	-28.3(7)
C22	C1	C17	C19	-154.1(5)
C22	C1	C20	O1	-125.0(6)
C22	C1	C20	C21	55.0(6)
C24	O3	C10	C9	-145.5(5)
C24	O3	C10	C11	93.6(6)

Table 12: Hydrogen Fractional Atomic Coordinates (×10^4) and Equivalent Isotropic Displacement Parameters (Å^2×10^3) for **3x**. U_{eq} is defined as 1/3 of the trace of the orthogonalised U_{ij}.

Atom	x	y	z	U_{eq}
H2A	6081.74	90.19	416.59	35
H2B	6589.36	-1273.89	1018.49	35
H3A	5142.8	-13.02	1075.81	35
H3B	5723.89	-915.15	1747.33	35
H4	5514.28	3236.99	1332.21	32
H5	5591.92	1738.7	2742.95	30
H6A	4297.41	3061.51	1718.38	34

Atom	x	y	z	U_{eq}
H6B	4385.37	916.33	2112.52	34
H7	3572.32	2766.62	2727.67	36
H9A	4176.41	4549.57	4258.19	40
H9B	3405.82	4685.14	3675.45	40
H10	3607.95	8098.42	3461.22	34
H11A	5059.88	7891.49	4413.4	39
H11B	4721.2	9846.52	3969.95	39
H12A	5671.03	8279.34	3426.59	35
H12B	4853.96	8255.87	2906.43	35
H14	5254.22	5774.65	2222.28	31
H15A	6684.6	4703.42	3090.64	36
H15B	6456.44	6895.24	2767.68	36
H16A	7316.59	5203.05	2123.97	37
H16B	6516.68	5741.64	1616.53	37
H18A	5455.21	4615.87	4352.23	49
H18B	6206.76	5257.41	4039.05	49
H18C	5832.3	3064.59	3868.97	49
H19A	7014	-97.42	2305.32	47
H19B	6788.69	1497.05	2864.13	47
H19C	7583.86	1741.02	2573.44	47
H21A	8798.87	2083.59	1720.21	59
H21B	8164.39	3842.04	1663.79	59
H21C	8485.89	3129.18	972.04	59
H22	7181.54	4519.34	687.29	40
H23A	6116.46	4970.66	-247.97	58
H23B	5841.39	5021.09	504.82	58
H23C	5702.62	3034.34	21.24	58
H25A	2982.41	8449.92	5493.37	56
H25B	2414.83	10253.7	5182.57	56
H25C	3290.79	10734.63	5533.17	56
H2	7300(50)	3050(140)	-340(50)	70(30)

Table 13: Hydrogen Bond information for **3x**.

D	H	A	d(D-H)/Å	d(H-A)/Å	d(D-A)/Å	D-H-A/deg
O2	H2	O1[1]	0.92(10)	1.94(9)	2.802(6)	154(8)

[1]3/2-x,1/2+y,-z

THE UNIVERSITY of EDINBURGH
Crystal Structure Service

Dr Gary S. Nichol
Crystallography Service Manager
Phone: +44 (0)131 650 4806
E–mail: g.s.nichol@ed.ac.uk

R_1=3.01%

3β-acetoxy-17 α-(propan-1-ol)-5-pregnen-2-one **3y**

Submitted by: **Kieran Nicholson**

Solved by: **Gary S Nichol**

Submitted by: **None**

Compound KN07-055 c2 was provided as crystals suitable for single crystal X-ray diffraction, yielding structure **3y**.

Crystal Data and Experimental

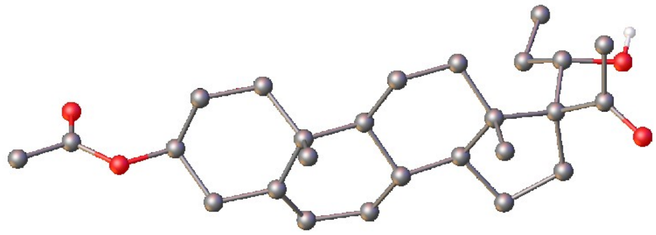

Experimental. Single colourless blade crystals of **3y** recrystallised from hexane by slow evaporation. A suitable crystal with dimensions 0.45 × 0.17 × 0.05 mm^3 was selected and mounted on a MITIGEN holder in Paratone oil. on a Bruker APEX-II CCD diffractometer. The crystal was kept at a steady T = 100.0 K during data collection. The structure was solved with the ShelXT 2018/2[5] solution program using dual methods and by using Olex2[6] as the graphical interface. The model was refined with ShelXL 2018/3[7] using full matrix least squares minimisation on F^2.

Crystal Data. $C_{26}H_{40}O_4$, M_r = 416.58, monoclinic, $C2$ (No. 5), a = 18.0777(9) Å, b = 6.4109(3) Å, c = 19.7176(10) Å, β = 101.499(2)°, α = γ = 90°, V = 2239.29(19) Å^3, T = 100.0 K, Z = 4, Z' = 1, μ(MoK$_\alpha$) = 0.081, 89418 reflections measured, 5584 unique (R_{int} = 0.0827) which were used in all calculations. The final wR_2 was 0.0779 (all data) and R_1 was 0.0301 (I≥2 σ(I)).

**Compound**	**3y**
Formula	$C_{26}H_{40}O_4$
$D_{calc.}$/ g cm^{-3}	1.236
μ/mm^{-1}	0.081
Formula Weight	416.58
Colour	colourless
Shape	blade
Size/mm^3	0.45×0.17×0.05
T/K	100.0
Crystal System	monoclinic
Flack Parameter	-0.03(18)
Hooft Parameter	0.0(2)
Space Group	$C2$
a/Å	18.0777(9)
b/Å	6.4109(3)
c/Å	19.7176(10)
α/°	90
β/°	101.499(2)
γ/°	90
V/Å^3	2239.29(19)
Z	4
Z'	1
Wavelength/Å	0.71073
Radiation type	MoK$_\alpha$
Θ_{min}/°	2.299
Θ_{max}/°	28.304
Measured Refl's.	89418
Indep't Refl's	5584
Refl's I≥2 σ(I)	4940
R_{int}	0.0827
Parameters	431
Restraints	1
Largest Peak	0.246
Deepest Hole	-0.129
GooF	1.057
wR_2 (all data)	0.0779
wR_2	0.0762
R_1 (all data)	0.0471
R_1	0.0301

Structure Quality Indicators

**Reflections:**	d min (Mo) 2θ=56.6° 0.75	I/σ(I) 22.0	Rint 8.27%	Full 50.5° 99.9
**Refinement:**	Shift 0.000	Max Peak 0.2	Min Peak -0.1	GooF 1.057
	Flack -.03(18)			

A colourless blade-shaped crystal with dimensions 0.45 × 0.17 × 0.05 mm^3 was mounted on a MITIGEN holder in Paratone oil. Data were collected using a Bruker APEX-II CCD diffractometer equipped with an Oxford Cryosystems Cryostream 800 low-temperature device operating at T = 100.0 K.

Data were measured using ϕ and ω scans using MoK_α radiation. . The maximum resolution that was achieved was Θ = 28.304° (0.75 Å).

The unit cell was refined using SAINT[9] on 9944 reflections, 11% of the observed reflections.

Data reduction, scaling and absorption corrections were performed using SAINT[9]. The final completeness is 99.90 % out to 28.304° in Θ. A multi-scan absorption correction was performed using SADABS-2016/2[10] was used for absorption correction. wR_2(int) was 0.1242 before and 0.0521 after correction. The Ratio of minimum to maximum transmission is 0.9642. The $\lambda/2$ correction factor is Not present. The absorption coefficient μ of this material is 0.081 mm^{-1} at this wavelength (λ = 0.71073Å) and the minimum and maximum transmissions are 0.719 and 0.746.

The structure was solved and the space group $C2$ (# 5) determined by the ShelXT 2018/2[5] structure solution program using using dual methods and refined by full matrix least squares minimisation on $\boldsymbol{F}^2$ using version 2018/3 of ShelXL 2018/3[7]. All non-hydrogen atoms were refined anisotropically. Hydrogen atom positions were calculated geometrically and refined using the riding model.

_refine_special_details: H atoms were identified from a difference map and freely refined.

The Flack parameter was refined to -0.03(18). Determination of absolute structure using Bayesian statistics on Bijvoet differences using the Olex2 results in 0.0(2). Note: The Flack parameter is used to determine chirality of the crystal studied, the value should be near 0, a value of 1 means that the stereochemistry is wrong and the model should be inverted. A value of 0.5 means that the crystal consists of a racemic mixture of the two enantiomers.

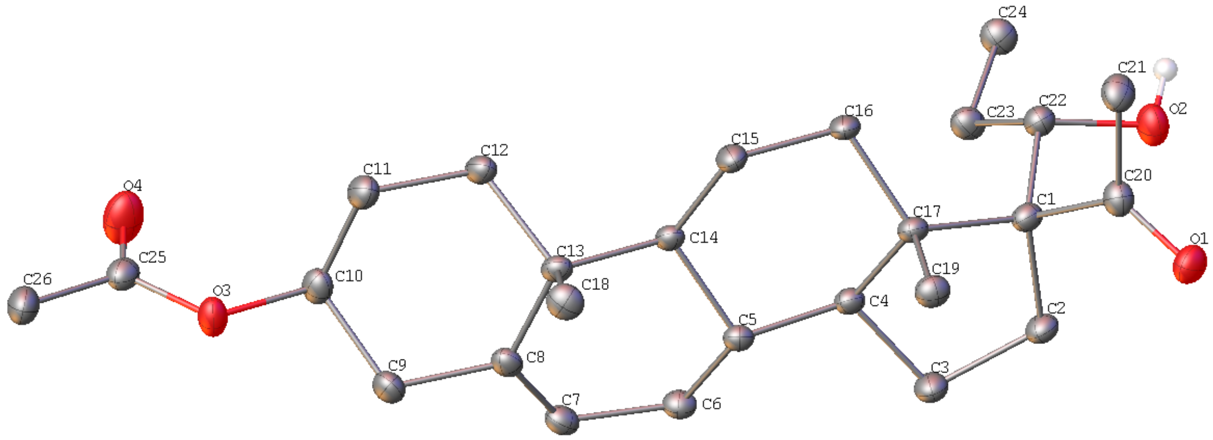

Figure 3: The molecular structure of **3y**. Displacement ellipsoids are at the 50% probability level and C-bound H atoms are not shown.

Data Plots: Diffraction Data

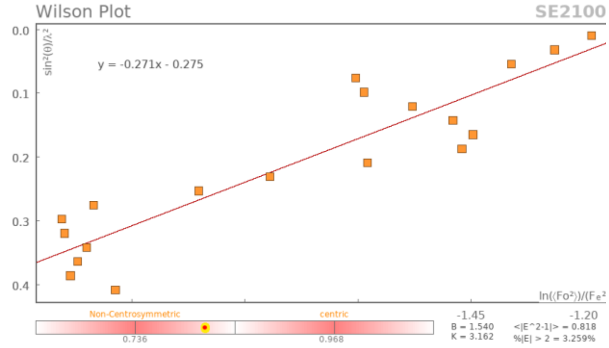

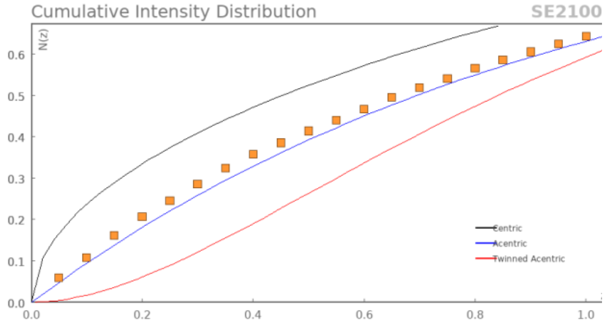

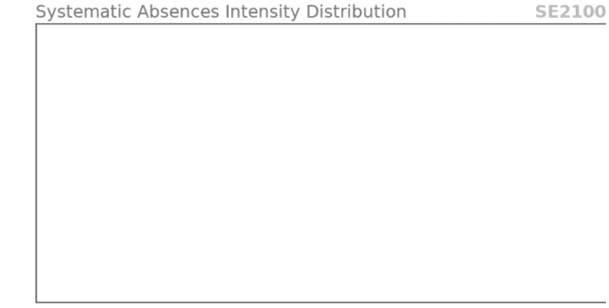

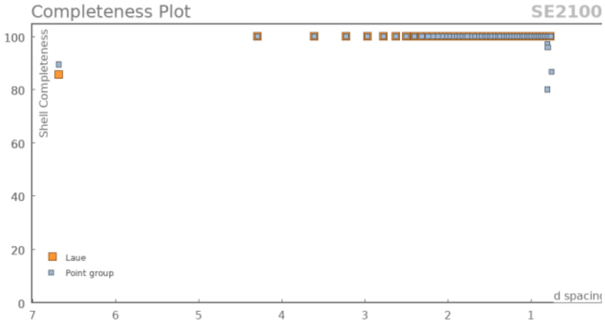

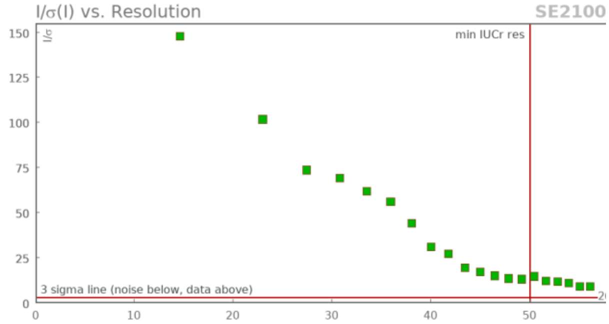

Data Plots: Refinement and Data

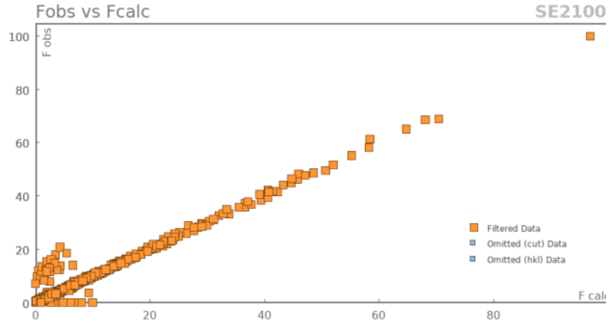

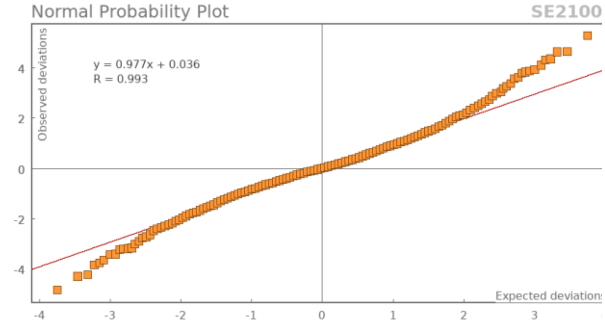

Reflection Statistics

Total reflections (after filtering)	89418	Unique reflections	5584
Completeness	0.999	Mean I/σ	37.61
hkl_{max} collected	(24, 8, 26)	hkl_{min} collected	(-24, -8, -26)
hkl_{max} used	(23, 8, 26)	hkl_{min} used	(-24, -8, 0)
Lim d_{max} collected	100.0	Lim d_{min} collected	0.36
d_{max} used	8.86	d_{min} used	0.75
Friedel pairs	5349	Friedel pairs merged	0

Inconsistent equivalents	412	R_{int}	0.0827
R_{sigma}	0.0454	Intensity transformed	0
Omitted reflections	0	Omitted by user (OMIT hkl)	0
Multiplicity	(163, 153, 246, 437, 944, 1253, 1483, 1409, 1236, 1192, 828, 753, 439, 312, 67, 19)	Maximum multiplicity	30
Removed systematic absences	0	Filtered off (Shel/OMIT)	0

Table 14: Fractional Atomic Coordinates (×10^4) and Equivalent Isotropic Displacement Parameters (Å^2×10^3) for **3y**. U_{eq} is defined as 1/3 of the trace of the orthogonalised U_{ij}.

Atom	x	y	z	U_{eq}
O1	2223.1(7)	-538.0(18)	3733.0(6)	24.2(3)
O2	2930.6(7)	2701.4(19)	4863.5(6)	24.5(3)
O3	6399.2(6)	7715.2(18)	481.7(6)	22.6(2)
O4	7043.4(8)	10535(2)	946.2(7)	36.8(3)
C1	3211.5(8)	2034(2)	3771.2(8)	16.3(3)
C2	3765.5(9)	209(2)	4022.9(8)	18.5(3)
C3	4402.9(9)	269(2)	3604.2(9)	19.6(3)
C4	4302.3(8)	2362(2)	3227.2(8)	15.4(3)
C5	4677.2(8)	2672(2)	2608.0(8)	14.3(3)
C6	5531.0(8)	2362(2)	2811.4(8)	16.8(3)
C7	5937.0(9)	3123(2)	2268.5(8)	18.3(3)
C8	5640.0(8)	4391(2)	1750.7(8)	16.3(3)
C9	6081.0(9)	5012(2)	1204.9(9)	19.6(3)
C10	6092.3(10)	7370(2)	1103.0(8)	19.8(3)
C11	5310.2(9)	8321(2)	996.9(9)	21.0(3)
C12	4913.4(9)	7690(2)	1579.1(8)	18.0(3)
C13	4846.0(8)	5301(2)	1665.0(8)	14.9(3)
C14	4511.0(8)	4893(2)	2318.6(8)	14.2(3)
C15	3665.3(9)	5429(2)	2204.2(9)	18.3(3)
C16	3302.8(9)	5002(2)	2829.5(9)	17.2(3)
C17	3438.2(8)	2730(2)	3068.2(8)	15.2(3)
C18	4346.9(10)	4343(3)	1016.2(9)	20.6(3)
C19	3042.0(9)	1230(2)	2498.9(9)	18.6(3)
C20	2385.6(9)	1261(2)	3643.4(8)	18.9(3)
C21	1763.0(9)	2839(3)	3405.8(10)	22.1(3)
C22	3244.0(9)	3738(2)	4336.8(9)	17.7(3)
C23	4017.9(9)	4646(2)	4637.1(9)	19.3(3)
C24	4014.4(10)	6029(3)	5265.5(9)	21.7(3)
C25	6842.8(9)	9381(2)	461.8(9)	20.4(3)
C26	7049.8(10)	9616(3)	-232.6(10)	25.6(4)

Table 15: Anisotropic Displacement Parameters (×10^4) for **3y**. The anisotropic displacement factor exponent takes the form: $-2\pi^2[h^2a^{*2} \times U_{11} + ... +2hka^* \times b^* \times U_{12}]$

Atom	U_{11}	U_{22}	U_{33}	U_{23}	U_{13}	U_{12}
O1	23.4(6)	19.6(5)	30.2(7)	1.2(5)	7.2(5)	-4.6(4)
O2	28.1(6)	26.8(5)	20.9(6)	-1.7(5)	10.4(5)	-5.1(5)
O3	26.7(6)	22.1(5)	21.6(6)	-2.9(5)	11.3(5)	-7.0(4)
O4	46.2(8)	33.3(6)	32.9(7)	-7.6(6)	12.3(6)	-17.3(6)
C1	16.1(7)	13.1(5)	19.4(7)	0.9(5)	3.1(6)	0.6(5)
C2	19.4(8)	14.5(6)	21.4(8)	3.8(6)	3.9(6)	2.2(6)
C3	19.8(8)	16.4(6)	23.2(8)	4.2(6)	5.4(7)	4.2(6)
C4	16.4(7)	11.5(6)	18.1(7)	-0.5(5)	3.4(6)	0.9(5)
C5	15.0(7)	12.7(5)	15.1(7)	-1.2(6)	2.7(5)	1.3(5)
C6	15.9(7)	16.3(6)	17.5(7)	0.2(6)	1.4(6)	2.8(5)
C7	15.2(7)	18.0(6)	22.1(8)	-2.8(6)	4.7(6)	1.5(5)

Atom	U_{11}	U_{22}	U_{33}	U_{23}	U_{13}	U_{12}
C8	17.8(7)	15.2(5)	16.7(7)	-4.3(6)	5.4(6)	-0.2(5)
C9	21.1(8)	19.7(6)	20.0(8)	-1.5(6)	8.8(6)	1.3(6)
C10	24.8(8)	20.0(7)	16.1(7)	-2.0(6)	7.9(6)	-2.9(6)
C11	27.0(9)	18.2(7)	19.4(8)	2.3(6)	8.5(7)	0.7(6)
C12	23.4(8)	15.0(6)	17.0(7)	0.0(6)	7.4(6)	2.5(6)
C13	18.7(7)	12.6(5)	13.7(7)	-1.5(5)	3.9(6)	1.0(5)
C14	17.4(7)	11.3(5)	14.4(7)	-0.9(5)	4.2(6)	0.9(5)
C15	20.6(8)	14.3(6)	21.2(8)	4.6(6)	7.4(6)	4.3(6)
C16	17.6(8)	12.3(6)	22.6(8)	2.5(6)	6.2(6)	3.8(5)
C17	16.7(7)	11.7(5)	17.5(7)	0.2(5)	4.2(6)	1.6(5)
C18	21.9(8)	22.7(7)	16.3(8)	-2.9(6)	1.9(6)	-0.2(6)
C19	18.5(8)	17.0(6)	19.8(8)	-1.2(6)	2.3(6)	-0.2(6)
C20	19.1(8)	21.2(6)	16.9(8)	0.9(6)	4.8(6)	-1.2(6)
C21	16.3(8)	24.3(7)	25.6(8)	4.1(7)	3.6(7)	-0.3(6)
C22	18.9(7)	15.7(6)	19.1(8)	0.6(5)	5.1(6)	0.7(5)
C23	18.6(7)	19.0(6)	20.6(8)	-3.4(6)	4.2(6)	-1.2(5)
C24	21.7(8)	22.9(7)	20.0(8)	-2.2(6)	3.2(6)	-0.5(6)
C25	16.9(7)	17.6(6)	27.0(8)	2.6(6)	5.0(6)	1.0(6)
C26	25.9(9)	22.3(7)	30.8(10)	5.6(7)	11.5(8)	-0.4(7)

Table 16: Bond Lengths in Å for **3y**.

Atom	Atom	Length/Å
O1	C20	1.212(2)
O2	C22	1.4406(19)
O3	C10	1.4589(18)
O3	C25	1.3410(18)
O4	C25	1.205(2)
C1	C2	1.556(2)
C1	C17	1.586(2)
C1	C20	1.545(2)
C1	C22	1.553(2)
C2	C3	1.546(2)
C3	C4	1.527(2)
C4	C5	1.523(2)
C4	C17	1.549(2)
C5	C6	1.528(2)
C5	C14	1.5410(18)
C6	C7	1.495(2)
C7	C8	1.331(2)
C8	C9	1.515(2)
C8	C13	1.527(2)
C9	C10	1.525(2)
C10	C11	1.516(2)
C11	C12	1.525(2)
C12	C13	1.548(2)
C13	C14	1.552(2)
C13	C18	1.539(2)
C14	C15	1.539(2)
C15	C16	1.532(2)
C16	C17	1.5358(19)
C17	C19	1.542(2)
C20	C21	1.517(2)
C22	C23	1.522(2)
C23	C24	1.525(2)
C25	C26	1.497(2)

Table 17: Bond Angles in ° for **3y**.

Atom	Atom	Atom	Angle/°
C25	O3	C10	118.37(13)
C2	C1	C17	103.18(12)
C20	C1	C2	110.52(11)
C20	C1	C17	110.93(12)
C20	C1	C22	103.87(12)
C22	C1	C2	111.71(13)
C22	C1	C17	116.77(12)
C3	C2	C1	108.19(12)
C4	C3	C2	104.62(12)
C3	C4	C17	104.46(12)
C5	C4	C3	118.43(12)
C5	C4	C17	114.20(12)
C4	C5	C6	111.12(12)
C4	C5	C14	109.42(11)
C6	C5	C14	109.25(12)
C7	C6	C5	112.58(12)
C8	C7	C6	124.58(13)
C7	C8	C9	121.09(13)
C7	C8	C13	122.99(14)
C9	C8	C13	115.91(13)
C8	C9	C10	112.15(12)
O3	C10	C9	106.16(12)
O3	C10	C11	108.87(13)
C11	C10	C9	112.35(14)
C10	C11	C12	110.81(13)
C11	C12	C13	113.83(12)
C8	C13	C12	107.29(12)
C8	C13	C14	110.95(12)

Atom	Atom	Atom	Angle/°
C8	C13	C18	108.79(12)
C12	C13	C14	108.04(11)
C18	C13	C12	110.46(13)
C18	C13	C14	111.23(12)
C5	C14	C13	112.55(11)
C15	C14	C5	112.04(12)
C15	C14	C13	112.43(12)
C16	C15	C14	114.45(13)
C15	C16	C17	110.40(12)
C4	C17	C1	101.90(11)
C16	C17	C1	118.78(12)
C16	C17	C4	107.41(11)
C16	C17	C19	110.24(12)

Atom	Atom	Atom	Angle/°
C19	C17	C1	107.31(11)
C19	C17	C4	110.85(12)
O1	C20	C1	122.41(14)
O1	C20	C21	119.56(15)
C21	C20	C1	118.02(13)
O2	C22	C1	103.26(11)
O2	C22	C23	111.04(13)
C23	C22	C1	116.66(13)
C22	C23	C24	112.73(13)
O3	C25	C26	111.52(14)
O4	C25	O3	123.56(16)
O4	C25	C26	124.92(15)

Table 18: Torsion Angles in ° for **3y**.

Atom	Atom	Atom	Atom	Angle/°
O2	C22	C23	C24	-52.63(17)
O3	C10	C11	C12	170.49(12)
C1	C2	C3	C4	11.28(16)
C1	C22	C23	C24	-170.54(13)
C2	C1	C17	C4	-34.48(13)
C2	C1	C17	C16	-152.16(13)
C2	C1	C17	C19	82.06(13)
C2	C1	C20	O1	-0.2(2)
C2	C1	C20	C21	179.03(14)
C2	C1	C22	O2	-67.86(15)
C2	C1	C22	C23	54.21(17)
C2	C3	C4	C5	-162.01(13)
C2	C3	C4	C17	-33.62(15)
C3	C4	C5	C6	-58.42(17)
C3	C4	C5	C14	-179.13(13)
C3	C4	C17	C1	42.59(14)
C3	C4	C17	C16	168.17(13)
C3	C4	C17	C19	-71.35(15)
C4	C5	C6	C7	-166.80(11)
C4	C5	C14	C13	-177.52(11)
C4	C5	C14	C15	-49.66(16)
C5	C4	C17	C1	173.51(10)
C5	C4	C17	C16	-60.92(15)
C5	C4	C17	C19	59.57(15)
C5	C6	C7	C8	16.1(2)
C5	C14	C15	C16	50.63(17)
C6	C5	C14	C13	60.63(15)
C6	C5	C14	C15	-171.51(12)
C6	C7	C8	C9	-177.37(14)
C6	C7	C8	C13	2.5(2)
C7	C8	C9	C10	-128.82(16)
C7	C8	C13	C12	128.39(15)
C7	C8	C13	C14	10.58(19)
C7	C8	C13	C18	-112.10(16)

Atom	Atom	Atom	Atom	Angle/°
C8	C9	C10	O3	-169.12(12)
C8	C9	C10	C11	-50.22(18)
C8	C13	C14	C5	-42.02(16)
C8	C13	C14	C15	-169.68(11)
C9	C8	C13	C12	-51.74(16)
C9	C8	C13	C14	-169.54(12)
C9	C8	C13	C18	67.78(16)
C9	C10	C11	C12	53.20(18)
C10	O3	C25	O4	5.1(2)
C10	O3	C25	C26	-174.70(13)
C10	C11	C12	C13	-57.06(18)
C11	C12	C13	C8	54.42(17)
C11	C12	C13	C14	174.11(13)
C11	C12	C13	C18	-64.02(17)
C12	C13	C14	C5	-159.37(12)
C12	C13	C14	C15	72.97(15)
C13	C8	C9	C10	51.30(18)
C13	C14	C15	C16	178.56(11)
C14	C5	C6	C7	-45.99(16)
C14	C15	C16	C17	-54.62(17)
C15	C16	C17	C1	171.53(13)
C15	C16	C17	C4	56.77(16)
C15	C16	C17	C19	-64.11(16)
C17	C1	C2	C3	14.64(15)
C17	C1	C20	O1	113.62(17)
C17	C1	C20	C21	-67.15(17)
C17	C1	C22	O2	173.73(12)
C17	C1	C22	C23	-64.20(17)
C17	C4	C5	C6	177.90(11)
C17	C4	C5	C14	57.18(15)
C18	C13	C14	C5	79.23(15)
C18	C13	C14	C15	-48.42(15)
C20	C1	C2	C3	133.29(13)
C20	C1	C17	C4	-152.84(11)
C20	C1	C17	C16	89.48(15)
C20	C1	C17	C19	-36.30(15)
C20	C1	C22	O2	51.27(14)
C20	C1	C22	C23	173.34(13)
C22	C1	C2	C3	-111.60(14)
C22	C1	C17	C4	88.45(14)
C22	C1	C17	C16	-29.23(18)
C22	C1	C17	C19	-155.01(12)
C22	C1	C20	O1	-120.14(17)
C22	C1	C20	C21	59.09(17)
C25	O3	C10	C9	-144.11(14)
C25	O3	C10	C11	94.74(16)

Table 19: Hydrogen Fractional Atomic Coordinates (×10[4]) and Equivalent Isotropic Displacement Parameters (Å[2]×10[3]) for **3y**. U_{eq} is defined as 1/3 of the trace of the orthogonalised U_{ij}.

Atom	x	y	z	U_{eq}
H2	2895(14)	3630(50)	5180(13)	53(7)
H2A	3984(10)	400(30)	4548(11)	20(5)
H2B	3504(11)	-1090(30)	3973(10)	22(5)
H3A	4905(10)	160(30)	3894(9)	16(4)
H3B	4354(11)	-850(40)	3251(11)	31(6)
H4	4526(9)	3540(30)	3542(9)	12(4)
H5	4480(9)	1660(30)	2238(8)	8(4)
H6A	5741(11)	3030(30)	3263(11)	26(5)
H6B	5650(11)	910(30)	2896(10)	25(5)
H7	6451(11)	2690(30)	2300(9)	21(5)
H9A	5857(10)	4410(30)	744(10)	20(4)
H9B	6596(13)	4390(40)	1301(12)	44(6)
H10	6434(11)	8000(30)	1486(10)	25(5)
H11A	5362(12)	9810(40)	1001(12)	41(6)
H11B	5003(10)	7810(30)	540(10)	17(4)
H12A	5206(11)	8300(30)	2029(11)	26(5)
H12B	4387(10)	8340(30)	1501(9)	15(4)
H14	4795(10)	5830(30)	2690(10)	19(5)
H15A	3386(10)	4630(30)	1795(10)	19(5)
H15B	3574(11)	6870(30)	2078(10)	26(5)
H16A	3546(11)	5900(30)	3219(11)	25(5)
H16B	2768(10)	5350(30)	2724(9)	12(4)
H18A	4608(11)	4390(30)	640(11)	30(5)
H18B	4241(10)	2920(30)	1120(10)	21(5)
H18C	3857(11)	4940(30)	891(10)	26(5)
H19A	3094(11)	-220(30)	2666(10)	24(5)
H19B	2476(11)	1490(30)	2350(10)	23(5)
H19C	3240(12)	1300(30)	2085(11)	27(5)
H21A	1663(12)	3560(40)	3802(12)	35(6)
H21B	1297(14)	2160(40)	3186(12)	50(7)
H21C	1861(11)	3850(30)	3066(11)	28(5)
H22	2903(9)	4910(30)	4144(9)	14(4)
H23A	4411(10)	3520(30)	4764(10)	19(4)
H23B	4203(11)	5520(40)	4279(11)	30(5)
H24A	3594(11)	7020(40)	5175(10)	28(5)
H24B	3946(11)	5200(40)	5674(12)	35(6)
H24C	4505(11)	6740(30)	5392(10)	27(5)
H26A	6975(13)	8350(40)	-500(12)	39(6)
H26B	7561(15)	10020(40)	-193(13)	51(7)
H26C	6742(19)	10820(60)	-484(18)	86(10)

S12 References

1) J. E. Laudenschlager, L. A. Combee, M. K. Hilinski, *Org. Biomol. Chem.* **2019**, *17*, 9413-9417.

2) K. Nicholson, T. Langer, S. P. Thomas, *Org. Lett.* **2021**, *23*, 2498-2504.

3) Frost, J. R., Cheong, C. B., Akhtar, W. M., Caputo, D. F. J., Christensen, K. E., Stevenson, N. G., Donohoe, T. J. *Tetrahedron*, **2021**, *86*, 132051-132062.

4) B. H. Lipshutz, W. Chrisman, K. Noson, P. Papa, J. A. Sclafani, R. W. Vivian, J. M. Keith, *Tetrahedron*, **2000**, *56*, 2779-2788.

5) G. M. Sheldrick, SHELXT -Integrated Space-Group and Crystal-Structure Determination. Acta Crystallogr. Sect. A Found. Adv. 2015, 71, 3–8.

6) O. V Dolomanov, L. J. Bourhis, R. J. Gildea, J. Howard, H. Puschmann, OLEX2: A Complete Structure Solution, Refinement and Analysis Program. J. Appl. Crystallogr. 2009, 42, 339–341.

7) G. M. Sheldrick, Crystal Structure Refinement with SHELXL. Acta Crystallogr. Sect. C Struct. Chem. 2015, 71, 3–8.

8) CrysAlisPro Software System, Rigaku Oxford Diffraction, (2021).

9) Software for the Integration of CCD Detector System Bruker Analytical X-ray Systems, Bruker axs, Madison, WI (after 2013).

10) SADABS, Bruker axs, Madison, WI.
